# Systemic Analysis of Heat Shock Response Induced by Heat Shock and a Proteasome Inhibitor MG132

**DOI:** 10.1371/journal.pone.0020252

**Published:** 2011-06-30

**Authors:** Hee-Jung Kim, Hye Joon Joo, Yung Hee Kim, Soyeon Ahn, Jun Chang, Kyu-Baek Hwang, Dong-Hee Lee, Kong-Joo Lee

**Affiliations:** 1 The Center for Cell Signaling & Drug Discovery Research, College of Pharmacy, Ewha Womans University, Seoul, Korea; 2 Department of Life Science, Division of Life & Pharmaceutical Sciences, Department of Bioinspired Science, Ewha Womans University, Seoul, Korea; 3 School of Computer Science and Engineering, Soongsil University, Seoul, Korea; 4 Department of Research and Education, Seoul National University Bundang Hospital, Seongnam, Korea; St. Georges University of London, United Kingdom

## Abstract

The molecular basis of heat shock response (HSR), a cellular defense mechanism against various stresses, is not well understood. In this, the first comprehensive analysis of gene expression changes in response to heat shock and MG132 (a proteasome inhibitor), both of which are known to induce heat shock proteins (Hsps), we compared the responses of normal mouse fibrosarcoma cell line, RIF- 1, and its thermotolerant variant cell line, TR-RIF-1 (TR), to the two stresses. The cellular responses we examined included Hsp expressions, cell viability, total protein synthesis patterns, and accumulation of poly-ubiquitinated proteins. We also compared the mRNA expression profiles and kinetics, in the two cell lines exposed to the two stresses, using microarray analysis. In contrast to RIF-1 cells, TR cells resist heat shock caused changes in cell viability and whole-cell protein synthesis. The patterns of total cellular protein synthesis and accumulation of poly-ubiquitinated proteins in the two cell lines were distinct, depending on the stress and the cell line. Microarray analysis revealed that the gene expression pattern of TR cells was faster and more transient than that of RIF-1 cells, in response to heat shock, while both RIF-1 and TR cells showed similar kinetics of mRNA expression in response to MG132. We also found that 2,208 genes were up-regulated more than 2 fold and could sort them into three groups: 1) genes regulated by both heat shock and MG132, (e.g. chaperones); 2) those regulated only by heat shock (e.g. DNA binding proteins including histones); and 3) those regulated only by MG132 (e.g. innate immunity and defense related molecules). This study shows that heat shock and MG132 share some aspects of HSR signaling pathway, at the same time, inducing distinct stress response signaling pathways, triggered by distinct abnormal proteins.

## Introduction

Heat shock response (HSR) is an evolutionarily conserved defense mechanism against sudden stresses such as elevated temperatures or environmental changes. A major component of HSR is the induction of heat shock proteins (Hsps) which are up-regulated when the transcription factor, heat shock factor (HSF), binds to a DNA sequence motif called the heat shock element (HSE) [1]. Most Hsps are molecular chaperones that play important roles in repair and removal of misfolded and denatured proteins thereby conserving cellular protein homeostasis [2]. Another component of HSR is the induction of thermotolerance in the cells which enables them to resist lethal effects caused by various stresses including oxidative stress, hypoxia and sodium arsenite [3], [4], [5], [6], [7], [8]. It is widely believed that the chaperonic function of Hsps is associated with the development of thermotolerance [9]. Hsps also promote the degradation of abnormal proteins through ubiquitin-proteasome system (UPS) which involves post-translational conjugation of ubiquitins to proteins and degradation by 26S proteasome. Thus heat shock response and ubiquitin-proteasome degradation pathways are closely interconnected [10]. When proteasome function is blocked by inhibitors such as MG132, abnormal proteins accumulate and the expression of Hsps is enhanced. Because MG132 promotes unfolded protein response (UPR), it has recently been called a proteostasis regulator [11], [12], [13], [14], [15].

Both heat shock and MG132 induce accumulation of poly-ubiquitinated proteins [16], [17], but the affected proteins in the two cases are different. Heat shock causes denaturation of *de novo* synthesized proteins, as well as labile proteins [18]. In contrast, MG132 accumulates about 30% of newly synthesized proteins destined to be degraded within minutes of their synthesis, as well as short-lived proteins such as signaling molecules [19], [20]. Thus heat shock mainly produces denatured proteins while MG132 induces accumulation of denatured proteins plus normally-structured proteins. It has been suggested that the accumulated non-native proteins are signaling molecules that activate HSF [16].

We wondered whether accumulation of denatured proteins following heat shock and of poly-ubiquitinated proteins following MG132 treatment induce the same or different signaling pathways. In this, the first comprehensive analysis of gene expression in response to heat shock and MG132, we compared the responses of normal mouse fibrosarcoma cell line, RIF-1, and its thermotolerant variant cell line, TR-RIF-1 (TR), to these two stresses. TR cell line is a heat resistant strain produced following repeated heat shocks of RIF-1 cell line [21], and also resistant to other protein denaturants such as diamide and sodium arsenite [5]. In order to determine whether heat shock and UPR have common pathway, we examined the response of MG132 treatment in heat resistant TR cells.

The cellular responses we examined, included Hsp expression, cell viability, total protein synthesis patterns and accumulation of poly-ubiquitinated proteins. We also compared, using microarray analysis, the mRNA expression profiles and kinetics, in the two cell lines following the two treatments. We found that a total of 2,208 genes were up-regulated more than 2 fold, which could be sorted into three groups: genes up-regulated in common by both of heat shock and MG132; genes up-regulated only by heat shock; and genes up-regulated only by MG132. Also, using gene set enrichment analysis (GSEA) in conjunction with co-regulation gene set, we inferred the possible transcription factors promoting these responses. We also found that the expression of immunity and defense group of genes increased in MG132 treated cells, which might explain the reported anti-viral effect of MG132 [22].

## Results

### Differential responses of RIF-1 and TR cells to heat shock and MG132

**Figure 1:**
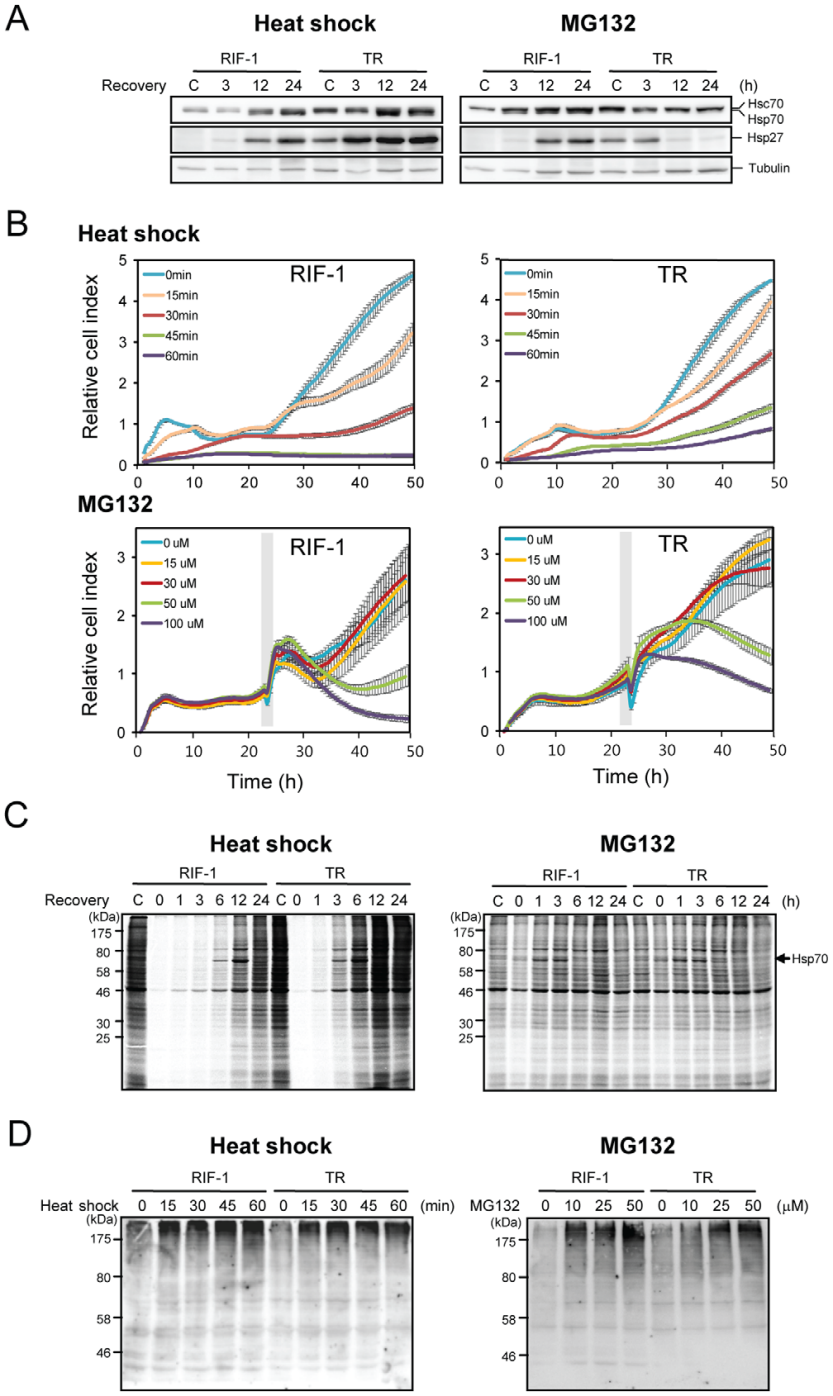
Differential responses of RIF-1 and TR cells to heat shock and MG132 treatment. (A) RIF-1 and TR cells were heat shock treated at 45°C for 30 min or treated with 50 µM MG132 for 1 h, and recovered at the indicated times in fresh media. Hsc70, Hsp70 and Hsp27 were analyzed by Western blot analysis using anti- Hsc70/Hsp70 and anti-Hsp27 antibody. As a loading control, tubulin level was shown. (B, upper panels) RIF-1 and TR cells were heat shock treated at 45°C for the indicated times and recovered at 2 h at 37°C in fresh media. Cells were plated in 96 well E-plate and cell growth was monitored using real-time cell analyzer. Blue lines, untreated cells; orange lines, 15 min; red lines, 30 min; green lines, 45 min; purple lines, 60 min heat shock. (B, lower panels) For MG132 treatment, RIF-1 and TR cells were treated with various concentrations of MG132 for 1 h (grey box) and cell growth was monitored in fresh media. Blue lines, DMSO treated control cells; orange lines, 15 µM; red lines, 30 µM; green lines, 50 µM; purple lines, 100 µM MG132. All samples were triplicated and the average values with standard deviations were represented. (C) RIF-1 and TR cells were heat shock treated at 45°C for 30 min or treated with 50 µM MG132 for 1 h and recovered at the indicated times in fresh media. Cells were metabolically pulse labeled with [^35^S]methionine containing media for 1 h. (D) RIF-1 and TR cells were heat shock treated at 45°C for the indicated times or treated with indicated concentrations of MG132 for 1 h. Cells were collected immediately after each treatment and analyzed by Western blot analysis using anti-ubiquitin antibody.

TR cells resist heat shock-caused changes in cell viability, whole-cell protein synthesis, and activation of signaling molecules involved in cell death, among others [23]. TR cells also exhibit cross resistance to other environmental stresses including diamide and sodium arsenite [5]. To examine molecular differences of RIF-1 and TR cells, we compared the mRNA levels of each gene in RIF-1 and TR cells, using microarray analysis (Table S1, Table S2, Data Set S2). We found that mRNAs of chaperones such as Hspb1, Cryab, Hspca, Dnajb1 and Hsp105 were more than 2 fold up-regulated in TR cells, compared to RIF-1 cells. In addition, immune signaling related genes such as Igtp, AI481100, Ifit3, Isgf3g, Tlr5 and Il15 were up-regulated more than 2 fold in TR cells. Because the Illumina gene chip we used does not contain the probe for inducible forms of Hsp70 genes, we performed Western blot analysis. We found that both Hsc70 and Hsp70 were also up-regulated in TR cells, in contrast to RIF-1 cells (Figure 1A).

We then examined whether the stress responses induced in both cell lines by heat shock or MG132, occurred via the same or distinct signaling pathways. Both heat shock and MG132 induced peak levels of Hsp70 and Hsp27 at 24 h in RIF-1 cells (Figure 1A). Interestingly, the kinetics of induction of Hsp70 and Hsp27 by heat shock in TR cells was similar to that in RIF-1 cells, but for starting from higher basal level than RIF-1 cells. However, MG132 treatment seemed to down-regulate the expression of Hsp70 and Hsp27 in TR cells. Both heat shock and MG132 induced the same isoforms of major chaperons, Hsp70 and Hsp27 as determined by 2D-PAGE and Western blot analysis in RIF-1 cells (Figure S1).

We then compared the effects of heat shock and MG132 stresses on cell growth. RIF-1 and TR cells were exposed to heat shock at 45°C for the indicated times, and to the indicated concentrations of MG132, and cell growth was monitored. Heat shock induced inhibition of cell growth was less in TR cells than RIF-1 cells (Figure 1B upper panels). Even after a lethal heat shock treatment (45 and 60 min at 45°C), a fraction of TR cells survived. Cell growth after MG132 treatment is shown in Figure 1B, lower panels. Because RIF-1 and TR cells showed distinct initial growth patterns following MG132 treatment, it was hard to decide which of these cell lines is more resistant to MG132-induced stress. We applied a linear mixed effects model for the growth index over time as implemented in the nmle R package to examine the growth difference between the samples over time [24], [25]. TR cells were more resistant to heat shock treatment than RIF-1 cells, while this trend is reversed with MG132 treatment (Table S3).

We compared total protein synthesis patterns, respectively following heat shock and MG132 treatments, employing pulse metabolic labeling. RIF-1 and TR cells were exposed to heat shock at 45°C for 30 min or to 50 µM MG132 for 1 h. Cells were incubated and labeled with [^35^S]methionine for 1 h following recovery at 37°C and newly synthesized proteins were estimated based on their labeling. Heat shock totally inhibited cellular protein synthesis immediately (Figure 1C left panel) in agreement with our previous report [23]. Total protein synthesis was recovered more rapidly in TR cells than in RIF-1 cells. However, MG132 treatment caused an immediate, slight inhibition of total cellular protein synthesis with rapid recovery in 1 h (Figure 1C right panel). Synthesis of Hsp70 clearly followed total cellular protein synthesis in both treatments. MG132 induced Hsp70 as effectively as heat shock. There were only slight differences in protein synthesis between RIF-1 and TR cells treated with MG132. This may be because the large amounts of denatured proteins induced by heat shock, other than poly-ubiquitin tagged proteins, affect the total protein synthetic machinery.

MG132 is an inhibitor of proteasome and MG132 treatment results in the accumulation of poly-ubiquitinated proteins destined to be degraded in proteasome. We compared the amounts of poly-ubiquitin tagged proteins accumulated after heat shock and MG132 treatments. RIF-1 and TR cells were exposed to heat shock for various times or incubated with various concentrations of MG132 for 1 h. Immediately after each treatment, poly-ubiquitinated proteins were measured by Western blot analysis using anti-ubiquitin antibody. As shown in Figure 1D left panel, more poly-ubiquitinated proteins were accumulated in RIF-1 cells than in TR cells in response to heat shock. Quantitation of the Western blot is shown in Figure S2A. One possible explanation for this might be that the heat shock-induced denatured proteins are more easily renatured or cleared in TR cells due to the higher levels of chaperones present in TR cells than in RIF-1 cells. These include Hsc70, Hsp70, Hsp27 (19.76 fold in mRNA level), crystallin αB (5.33 fold in mRNA level), Hsp90α (2.17 fold in mRNA level), Hsp40 homolog (2.08 fold in mRNA level) and Hsp110 (2.03 fold in mRNA level) (Table S1, Table S2, Data Set S2, Figure 1A). Another possible explanation may be the presence of higher levels of UPS molecules such as ubiquitin-activating enzyme E1-like (2.39 fold in mRNA level), proteasome subunits and ubiquitin conjugating E2 enzymes in TR cells (unpublished data of protein level). In contrast to the finding with heat shock, no differences were observed between RIF-1 and TR cells in MG132-induced accumulation of poly-ubiquitinated proteins (Figure 1D right panel, Figure S2B). This suggests that the basal degradation rates of proteins by UPS are similar in normal RIF-1 and TR cells; or that the rates of both degradation and poly-ubiquitination of TR cells might be faster than RIF-1 cells, and similar amounts of poly-ubiquitinated proteins exist in RIF-1 and TR cells.

### Global gene expressions in RIF-1 and TR cells exposed to heat shock and MG132

**Figure 2:**
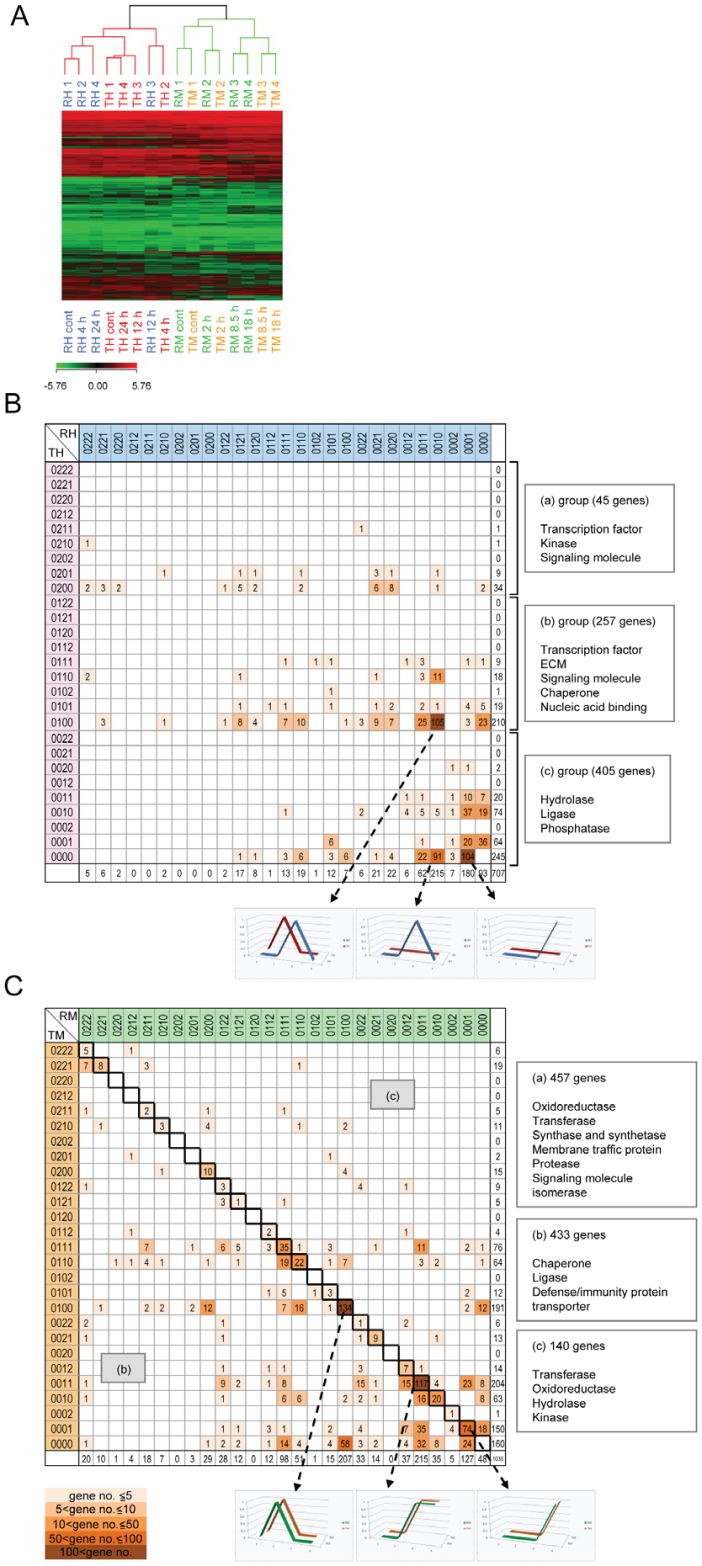
Hierarchical clustering of the gene chip data and recovery kinetics. (A) Cells were recovered at the indicated times after heat shock at 45°C for 30 min or treatment with 50 µM MG132 for 1 h. Total RNAs were analyzed using microarray analysis. Genes that changed are shown in color scales and each sample's expression patterns are analyzed by hierarchical clustering analysis. Each row of colored boxes displays the alteration in mRNA abundance for each gene. Each column displays the alteration in mRNA levels in each sample. The resulting dendrogram presents the relationships between the mRNA expression patterns in given samples. ‘R’ indicates RIF-1 cells, ‘T’ indicates TR cells, ‘H’ indicates heat shock, and ‘M’ indicates MG132 treatment. ‘1’ identifies control samples and ‘2’, ‘3’, and ‘4’ indicate recovery times which are shown under the dendrogram. (B, C) Comparison of heat shock and MG132 induced gene expression kinetics in RIF-1 and TR cells. Heat shock (B) or MG132 (C) induced gene expression kinetics of RIF-1 and TR cells are represented as symbols and shown in matrixes. ‘0’ symbolizes less than 1.5 fold induction of a gene. ‘1’ symbolizes between 1.5 and 3 fold induction and ’2’ symbolizes more than 3 fold induction. The concatenated 4 digit symbols of 4 time points were placed in a matrix horizontally for RIF-1 cells and vertically for TR cells. In the matching cells of RIF-1 and TR symbols, the gene numbers were written. Darker colors are used for cells with higher gene numbers. (B) The genes are grouped into (a), (b), and (c), according to the symbols of TR cells. The molecular functions of the genes are shown on the right side of the matrix. The top ranked 3 combiantions of kinetics of RIF-1 and TR cells are also shown as graphical charts. (C) The genes are grouped according to the location of the cells. The cells on the major diagonal line from top left to down right are group (a). The cells under the line are group (b) and the cells above the line are group (c). The molecular functions of the genes are shown next to the matrix. The top ranked 3 combinations of kinetics of RIF-1 and TR cells are also shown as graphical charts.

Global gene expressions in RIF-1 and TR cells exposed to heat shock and MG132 gleaned from microarray analysis are represented in Data Set S1 and S2. Data Set S1 contains genes that passed initial screening of the microarray data. Among the 12,339 genes in this data set, we selected 2,208 genes which changed more than 2 fold with *p*-value<0.05 (Data Set S2). To test the reliability of microarray data, we chose 3 genes with fold changes around 2 and performed real-time PCR. As shown in Figure S3, all of the gene chip data of the three genes in fold changes were reproduced in real-time PCR data with higher fold changes than those in the gene chip data. We assessed the overall expression patterns of the mRNA levels of these 2,208 genes by analyzing their hierarchical clustering data. The mRNA expression patterns are shown in two sections in Figure 2A, one for heat shock and the other for MG132 treated cells. In the heat shock samples, the mRNA expression patterns of RH3 (recovery for 12 h after heat shock) and TH2 (recovery for 4 h), are distinguishable from other samples, control (RH1, TH1) and recovered samples for 4, 12, and 24 h after heat shock (RH2/4, TH3/4). This indicates that remarkable changes occurred in mRNA profiles in RH3 and TH2 samples in response to heat shock, and that these changes are similar in RH3 and TH2. It appears that TR cells respond to heat shock stress about 8 h faster than RIF-1 cells. As cells recover from heat shock, mRNA profile seems to go back to normal (RH1 and TH1) because RH4 and TH4 are closer to RH1 and TH1 than to RH3 and TH2. When we expressed the fold change and kinetic in a 4 digit number for each gene (detailed in the legend for Figure 2B, C) and compared RIF-1 and TR cells, it appeared that heat shock induced changes in mRNA levels occurred faster and more transiently in TR cells than in RIF-1 cells (Figure 2B). On the other hand, MG132 treated RIF-1 and TR cells showed similar mRNA expression patterns at early time points (RM2 and TM2) but different mRNA expression patterns in later time points, 3 and 4 (Figure 2A). As shown in Figure 2C, 457 genes (44%) out of the 1,030 genes induced by MG132, showed same kinetics, suggesting that the gene expression kinetics induced by MG132 in RIF-1 and TR cells are more similar than those induced by heat shock.

**Figure 3:**
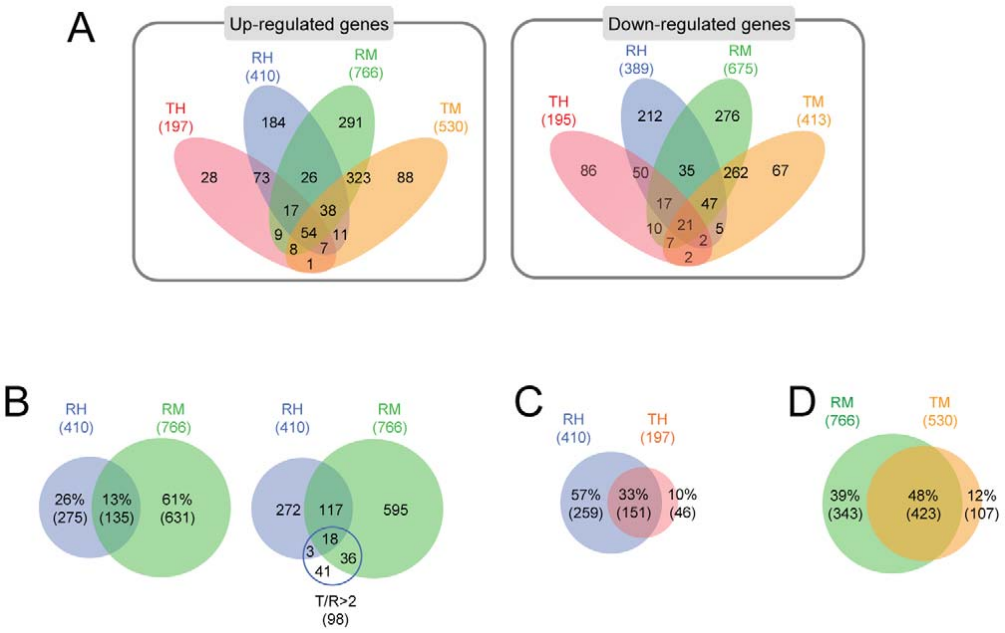
Venn diagram analysis of the gene chip data. (A) Up- or down-regulated genes in Data Set S2 are represented in Venn diagrams. They were grouped based on up- or down-regulations, cell lines, and stresses. ‘R’ indicates RIF-1 cells, ‘T’ indicates TR cells, ‘H’ indicates heat shock, and ‘M’ indicates MG132. Numbers represent gene numbers. (B) Comparison of genes up-regulated by heat shock and MG132 (left Venn diagram). 57 out of 98 genes, which were up-regulated in TR cells compared to RIF-1 cells in basal level more than 2 fold, were up-regulated by heat shock or MG132 in Data Set S2 (right Venn diagram). (C, D) Comparison of up-regulated genes in RIF-1 and TR cells in response to heat shock (C) and MG132 (D) treatment.

The microarray data on gene expression in RIF-1 and TR cells in response to heat shock and MG132, are presented in Venn diagrams. Numbers of genes which are up-regulated more than 2 fold and down-regulated less than 2 fold (Data Set S2) are summarized in Figure 3A. More genes were changed in their expression patterns in RIF-1 (RH and RM) than in TR cells (TH and TM) and more genes changed in their expression in response to MG132 (RM and TM) than to heat shock (RH and TH) treatment. We examined the details of gene expression changes in Data Set S2. We found that after heat shock (RH) and MG132 (RM) treatments, 410 and 766 genes respectively, were activated more than 2 fold at least at one time point during the recovery. Of these, 13% were activated by both heat shock and MG132 treatment (Figure 3B left Venn diagram). Sixty one % of these genes were up-regulated specifically by MG132 treatment. Twenty six % of the genes were heat shock specific genes, clearly unrelated to MG132 signaling pathway. Based on a previous study [26], we expected that the genes up-regulated in normal TR cells in contrast to normal RIF-1 cells, are stress responsive genes. Figure 3B right Venn diagram shows that 98 genes in TR cells were up-regulated more than 2 fold, compared to RIF-1 cells (T/R >2) (Data Set S2), and, of these, 57 genes (58.2%, *p*-value  = 0.000) were responsive to both heat shock and MG132 treatment. This indicates that most of the genes up-regulated in control TR cells are responsive to both heat shock and MG132 stress. We believe they possibly play major roles in cellular stress responses.

We also compared the numbers of genes responsive to both heat shock and MG132 in RIF-1 and TR cells. We found that 410 and 197 genes, respectively, were up-regulated in response to heat shock in RIF-1 and TR cells (Figure 3C). Among these, 151 (33%) genes were up-regulated in both RIF-1 and TR cells. In contrast, 766 genes in RIF-1 cells and 530 genes in TR cells were up-regulated by MG132 treatment. Of these, 423 genes (48%) were up-regulated in both RIF-1 and TR cells (Figure 3D). These results demonstrate that RIF-1 cells more readily respond to heat shock than TR cells. In contrast, relatively few differences were observed between RIF-1 and TR cells in their responses to MG132 treatment and this is more obvious when examined at fold cutoff 1.5 (35% in heat shock and 80% in MG132 treated cells).

Functional analysis using Gene Ontology (GO) revealed that genes with certain specific functions were over-expressed in heat shock or MG132 treated RIF-1 cells. For example, genes related to DNA conformation change, protein-DNA complex assembly, chromatin assembly/disassembly and chromosome organization (biological process group); nucleus and chromosome (cellular component group); and transcription regulator activity (molecular function group) were up-regulated in heat shock treated cells (Table 1). In contrast, genes related to cytokine production (biological process group), proteasome complex and MHC protein complex (cellular component group) were up-regulated in MG132 treated cells (Table 2). Some gene groups, for example, those involved with cell cycle and cell division, were down regulated in both heat shock and MG132 treated cells (Table S4 and S5). This might explain the decrease in cell growth shown in Figure 1B by both stresses. Genes involved in protein-DNA complex assembly, chromatin assembly and disassembly, chromosome organization, nucleus and other cellular components, nucleic acid binding and transcription regulation, were up-regulated in heat shock treated cells but down-regulated in MG132 treated cells, indicating that heat shock and MG132 treatments probably signal to DNA in different ways.

**Table 1:** Over-represented gene ontology categories of the up-regulated genes in heat shock treated RIF-1 cells.

GO ID	Term description	Population count	Study count	Adjusted
				*P*-values
**Biological Process**				
GO:0071103	DNA conformation change	108	22	0.000
GO:0023034	intracellular signaling pathway*	682	31	0.000
GO:0044085	cellular component biogenesis	581	39	0.000
GO:0007242	intracellular signaling cascade*	920	37	0.000
GO:0065004	protein-DNA complex assembly	85	22	0.001
GO:0043933	macromolecular complex subunit organization	420	35	0.002
GO:0006333	chromatin assembly or disassembly	105	22	0.002
GO:0008152	metabolic process*	5252	185	0.004
GO:0051276	chromosome organization	309	28	0.004
GO:0016265	death*	809	43	0.005
**Cellular Component**				
GO:0032993	protein-DNA complex	94	22	0.000
GO:0005622	intracellular*	7149	265	0.000
GO:0043226	organelle*	5933	217	0.000
GO:0005634	nucleus	3093	151	0.001
GO:0005694	chromosome	320	27	0.004
**Molecular Function**				
GO:0003676	nucleic acid binding	1822	99	0.000
GO:0005488	binding*	7994	267	0.000
GO:0030528	transcription regulator activity	892	47	0.002
GO:0033549	MAP kinase phosphatase activity	10	5	0.009

*Common categories of up-regulated genes in heat shock and MG132 treated RIF-1 cells.

**Table 2:** Over-represented gene ontology categories of the up-regulated genes in MG132 treated RIF-1 cells.

GO ID	Term description	Population count	Study count	Adjusted
				*P*-values
**Biological Process**				
GO:0008152	metabolic process*	5252	410	0.000
GO:0023034	intracellular signaling pathway*	682	41	0.000
GO:0007242	intracellular signaling cascade*	920	60	0.000
GO:0051239	regulation of multicellular organismal process	842	63	0.000
GO:0006950	response to stress	1083	109	0.000
GO:0048519	negative regulation of biological process	1277	104	0.000
GO:0016265	death*	809	81	0.000
GO:0001816	cytokine production	143	19	0.000
GO:0009987	cellular process	8567	547	0.003
GO:0009056	catabolic process	603	74	0.005
**Cellular Component**				
GO:0005622	intracellular*	7149	606	0.000
GO:0043226	organelle*	5933	484	0.000
GO:0005623	cell	10521	684	0.000
GO:0000502	proteasome complex	53	19	0.000
GO:0032991	macromolecular complex	1977	164	0.000
GO:0042611	MHC protein complex	26	7	0.007
GO:0005625	soluble fraction	258	34	0.007
**Molecular Function**				
GO:0005488	binding*	7994	564	0.000
GO:0003824	catalytic activity	3617	289	0.000
GO:0022890	inorganic cation transmembrane transporter activity	107	15	0.006

*Common categories of up-regulated genes in heat shock and MG132 treated RIF-1 cells.

### Stress specific genes

**Figure 4:**
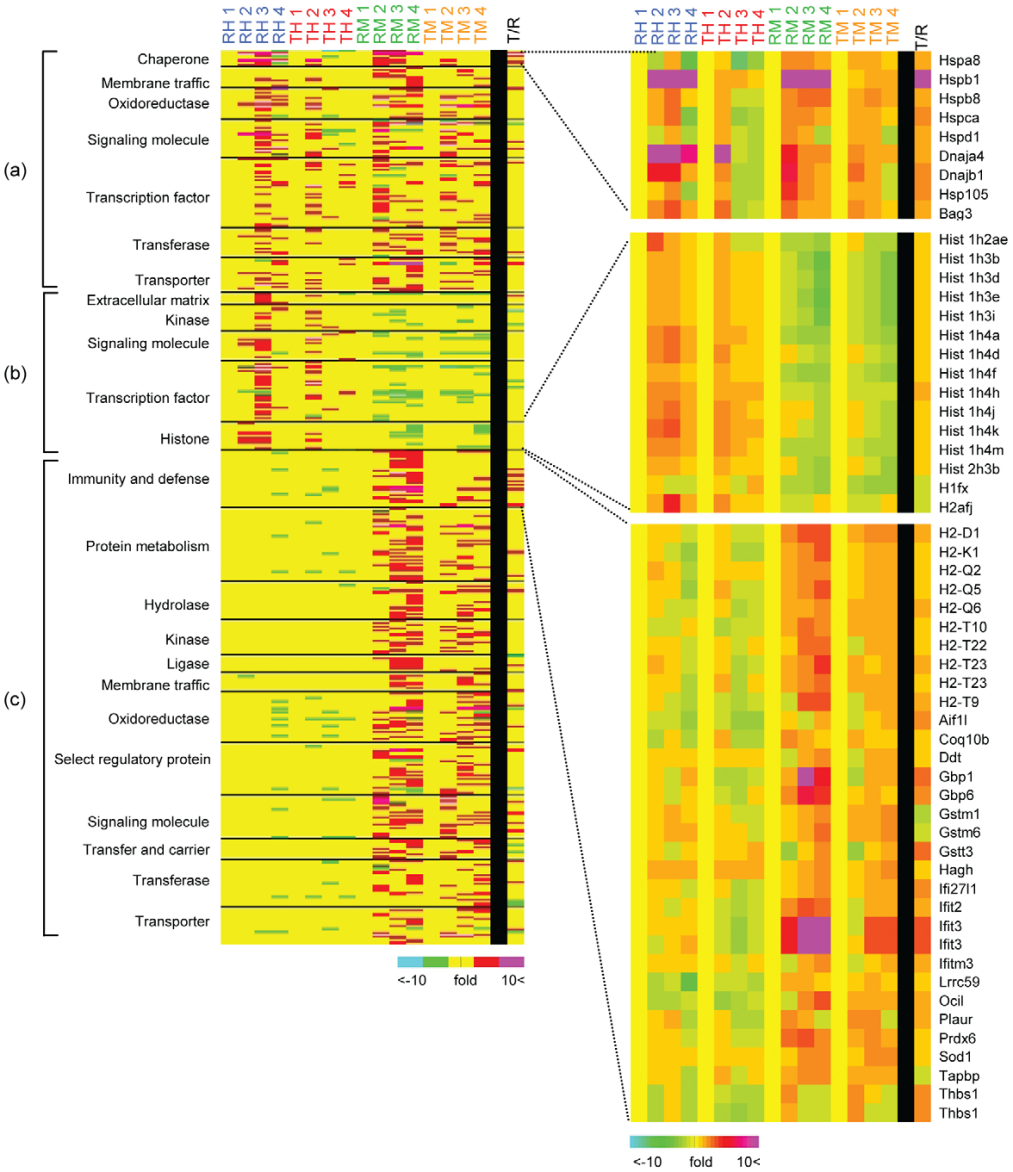
Gene groups up-regulated by both heat shock and MG132, heat shock only or MG132 only in RIF-1 and TR cells. Up-regulated genes are grouped according to their molecular functions and sorted into three groups based on their stress specificity: Genes up-regulated by both heat shock and MG132 (a), genes up-regulated only by heat shock (b), and genes up-regulated only by MG132 (c). The gene groups are subgrouped according to their functions. Chaperone, histone, immunity and defense groups are enlarged and individual gene names are shown. ‘R’ indicates RIF-1 cells, ‘T’ indicates TR cells, ‘H’ indicates heat shock, and ‘M’ indicates MG132. ‘1’ means control samples and ‘2’, ‘3’, and ‘4’ represent indicated recovery times (described in the Materials and Methods). Fold changes are represented by color scale.

We sorted the genes in Data Set S2, into three groups based on their functions: (a) those up-regulated by both heat shock and MG132; (b) those up-regulated only by heat shock, and (c) those up-regulated only by MG132 (Figure 4). The genes in each group are presented in Figure S4 and also listed in Data Set S3. Genes up-regulated by both heat shock and MG132 were found to be chaperone, membrane traffic, oxidoreductase, signaling molecules, transcription factors, transferases and transporter molecules in Figure 4 (a). Among them, the chaperone genes, Hspa8, Hspb1, Hspb8, Hspca, Hspd1, Dnaja4, Dnajb1, Hsp105, and Bag3, were up-regulated by both heat shock and MG132. We found that both heat shock and MG132 treatment activate HSF in agreement with previous reports that most of these genes are controlled by HSF [27], [28]. These genes were up-regulated to a greater degree in RIF-1 than in TR cells. All the genes in the chaperone group showed individual induction patterns in response to heat shock or MG132. A list of all chaperone genes on the gene chip is presented in Table S6. It can be seen that Dnaja4, Hspb1, and Cryab genes are highly up-regulated by both of heat shock and MG132. Among them, Hspb1 and Cryab are up-regulated to a similar extent by both heat shock and MG132, while Dnaja4 was more highly up-regulated in heat shock treated cells than in MG132. Some chaperone genes e, g. Dnajc family members, remained at their basal level after heat shock or MG132 treatment which suggests that, whether or not chaperones are up-regulated, depends on the stress and the cell type.

Heat shock treatment up-regulated mRNAs of extracellular matrix, kinases, signaling molecules, transcription factors and histones in Figure 4 (b). Interestingly, histone genes are up-regulated only by heat shock and the expression of some of their isoforms was decreased by MG132. For example, most of H3 and H4 histone family members are up-regulated in response to heat shock but their expressions decreased in response to MG132 treatment. Histone H3 family members are up-regulated more than 1.5 fold and H4 family members are up-regulated more than 2 fold by heat shock treatment (Table S7). On the other hand, most H2A histone family genes are down regulated by both heat shock and MG132 treatment. It is known that heat shock induces H2AX foci where DNA double strand breaks are recovered [29]. Except for the involvement of histone H2 family in heat shock response, the roles of other histone family members are hitherto unknown. As most histone H4 family members are dramatically up-regulated in response to heat shock, it might be interesting to examine their roles in heat shock signaling pathway. Various transcription factors are also up-regulated by heat shock (Table S8). Egr1, Fos, Jun and Fosb are up-regulated more than 3 fold, as early as 4 h after heat shock (RH2), reaching maximum at 12 h of recovery time point (RH3). Among these, Egr1 and Fos are known to be up-regulated more in HSF1−/− MEF cells than in wild type MEF cells by heat shock [27]. It means that they are stress responsive elements not under the control of HSF.

Gene induction in cells treated with MG132 is interesting. Genes involved in immunity and defense, protein metabolism, hydrolases, kinases, ligases, membrane traffic, oxidoreductases, select regulatory proteins, signaling molecules, transfer and carrier, transferases and transporters were up-regulated by MG132 treatment in Figure 4 (c). Genes related to cellular immunity and defense systems are markedly up-regulated by MG132. As shown in Figure 4, almost half of immunity and defense related genes up-regulated by MG132 are histocompatibility genes. In MHC function, proteasome processing is required for obtaining and loading peptides and facilitating the exit of MHC molecules from ER out into the plasma membrane [30]. Since MG132 inhibits proteasome activity, it is possible that the up-regulation of MHC genes might be necessary to induce MHC molecules in the plasma membrane. In support of this possibility, mRNA of TAP binding protein, which promotes the assembly of MHC class I molecules with peptides, is up-regulated more than 2 fold by MG132 treatment (Data Set S2). Innate immunity related signaling molecules such as TLR2, 4, 6, MyD88, Tbk1, NfkB1, 2, Traf3, 6, Trim25, Tank, Stat1, 2, Irf7, Isg15, Jak2, and Ifnar2 are markedly up-regulated in MG132 treated cells (Table S10). In addition, genes related to interferon activation and response, among others, are also up-regulated in MG132 treated cells. This might be result from the regulation of type I interferon signaling molecules [31], [32] or activation of innate immunity signaling pathway by accumulation of poly-ubiquitinated proteins.

### Antiviral effect of MG132

**Figure 5:**
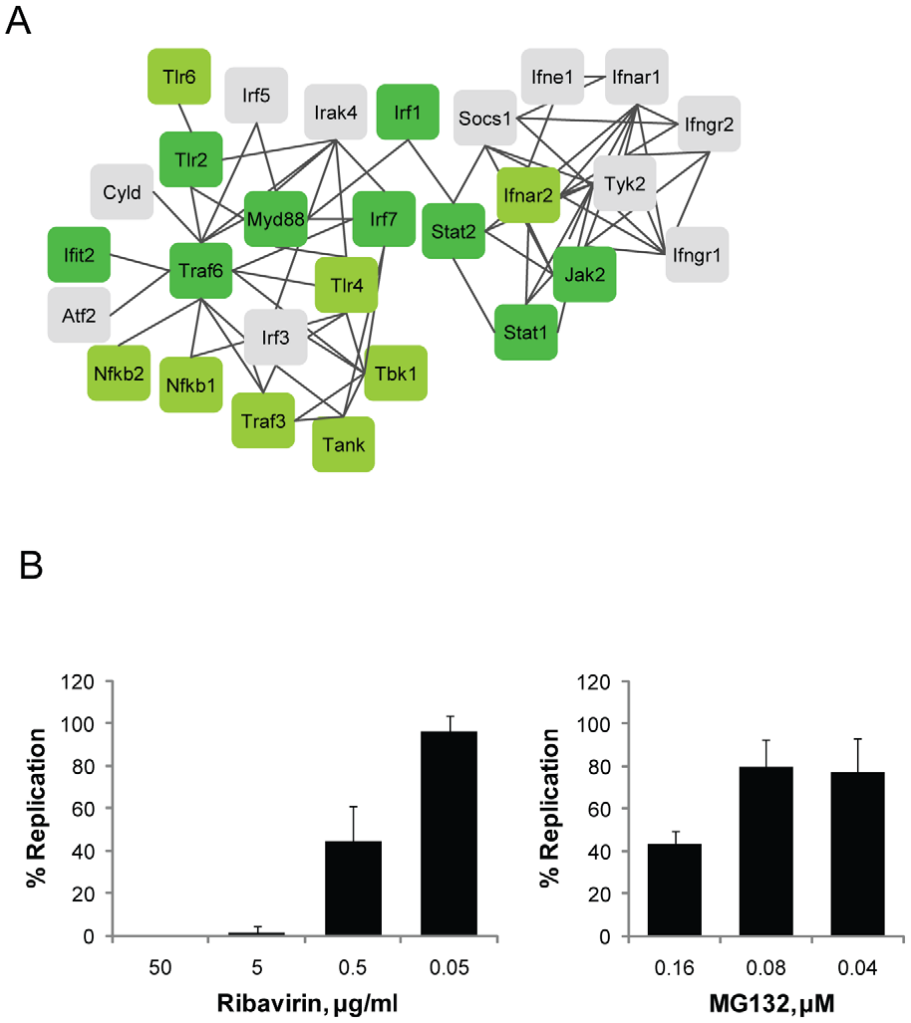
Inhibitory effect of MG132 on RSV replication. (A) Interaction map of the MG132 induced immune signaling molecules. MG132 induced immune signaling related genes were analyzed using protein interaction data retrieved from STRING. Green and yellowish green colored genes were up-regulated by MG132 more than 2 fold and 1.5 fold, respectively. Grey colored genes were up-regulated by MG132 less than 1.5 fold. Black lines indicate protein-protein interactions with confidence coefficient higher than 0.7. (B) The effect of MG132 on RSV replication was measured in plaque formation. Ribavirin was used as a positive control. The replication of RSV was presented as % of DMSO control.

As noted earlier, cytokine production and MHC protein complex related genes, both adaptive and innate immunity related signaling molecules, are up-regulated by MG132 (Table S8 and S10). Previous studies demonstrated the antiviral effect of MG132 and showed that viruses are affected in various steps of viral replication [22], [33], [34], [35], [36]. To understand the relationships among the immune signaling molecules and the antiviral effects, we employed protein interaction analysis software, STRING (http://string-db.org) (Figure 5A). We specifically examined whether MG132 treatment produces antiviral response to respiratory syncytial virus (RSV) by comparing RSV plaque formations after treatment with ribavirin, a known antiviral agent, and MG132 treatment (Figure 5B). We found that MG132 inhibited RSV plaque formation with IC50  = ∼0.15±0.03 µM. This finding is the first to note that MG132 induces anti RSV effects through gene regulation, in addition to the previously known inhibition of ubiquitination dependant protein degradation system [22].

### Prediction of stress activated transcription factors

**Figure 6:**
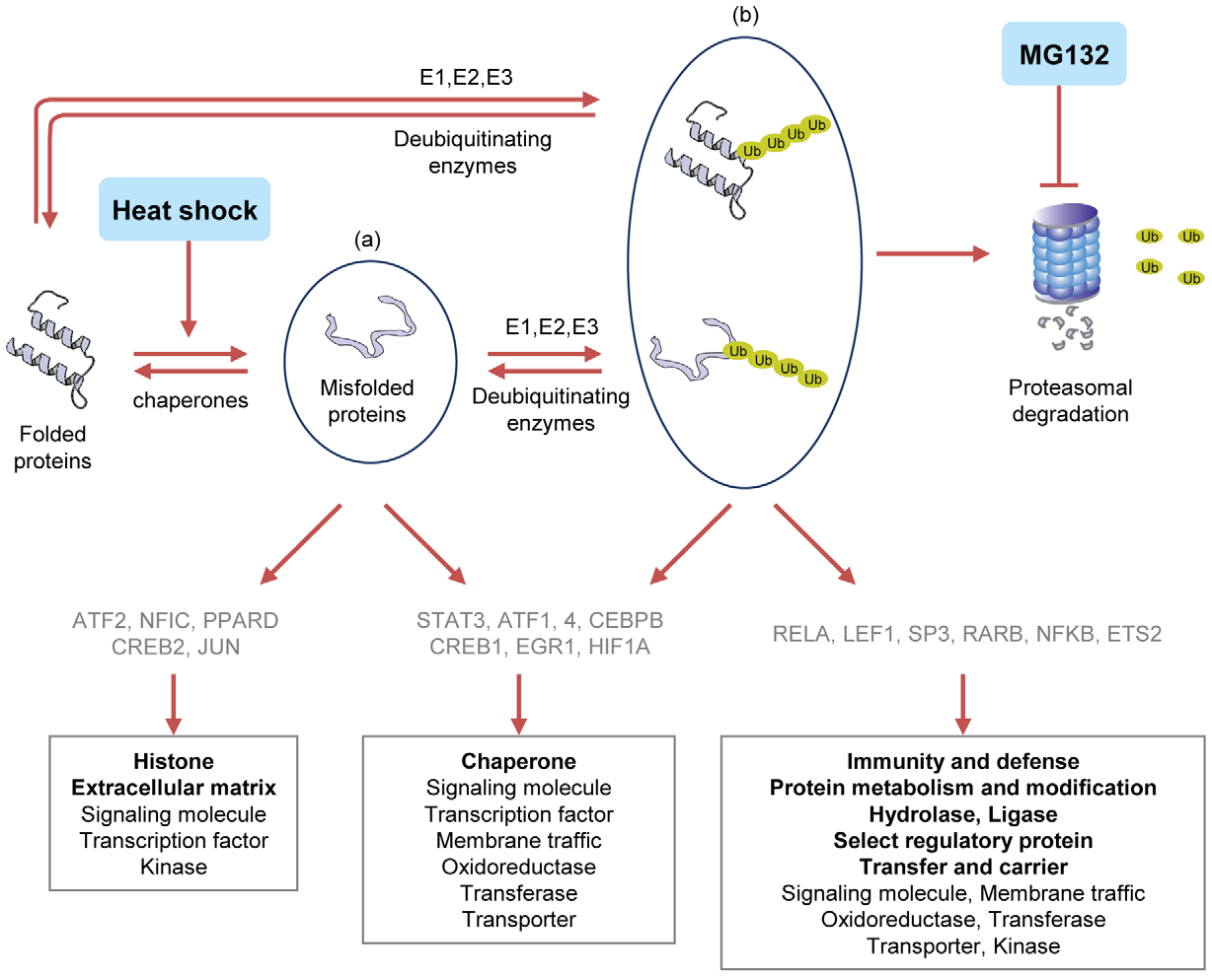
Summary of cellular responses to heat shock and MG132 in RIF-1 and TR cells. In heat shock treated cells, denatured proteins (a) increase which are then either renatured by chaperones or tagged by ubiquitins, followed by degradation in the proteasome. They seem to be the activators of HSR. In MG132 treated cells, ubiquitin tagged proteins (b) accumulate unless degraded in proteasome. They seem to be the initiators of MG132 induced signaling pathways. The possible activated transcription factors are listed, and the activated genes are grouped according to their molecular functions. Each group specific molecular function is indicated in bold.

Previous studies of heat shock signaling pathways have focused on the activation of HSFs. In this study, we looked for possible additional transcription factors activated by heat shock and MG132 signaling pathway. We employed GSEA, using co-regulation gene sets, as described in the Materials and Methods. This approach allowed us to predict that STAT3, ATF1, ATF4, CEBPB, CREB1, EGR1 and HIF1A are possibly activated by both heat shock and MG132, ATF2, NFIC, PPARD, CREB2 and JUN are possibly activated by heat shock, and that ETS2, LEF1, RELA, and SP3 are possibly activated by MG132. These predicted transcription factors are listed in Figure 6 in grey color. The predicted transcription factors with their *p*-values and FDRs are listed in Table S11. We performed luciferase assay to validate the prediction of ATF2 and STAT3 in MG132 treated RIF-1 cells (Figure S5). Supporting our prediction, STAT3 was activated but not ATF2 in MG132 treated RIF-1 cells. In addition, mRNA levels of EGR1, JUN, ATF4 and CEBPB were up-regulated as shown in Data Set S2. Our predictions are in line with previous reports that CEBPB, EGR1, HIF1, and STAT3 are activated in response to heat shock [37], [38], [39] and that ATF4, CEBPB and STAT3 are activated by MG132 treatment [40], [41], [42].

## Discussion

In this study, we tried to provide a genome-wide and kinetic comparison of the heat shock responses of normal mouse fibrosarcoma cell line, RIF- 1, and its thermotolerant variant cell line, TR, to two similar but nonidentical stresses from heat shock and MG132 treatments. Both heat shock and MG132, up-regulated chaperone gene expressions. TR cells were tolerant to heat shock but not to MG132 based on their viability. The accumulation pattern of poly-ubiquitinated proteins and gene expression profiles and kinetics, of heat shock treated RIF-1 and TR cells, were different from those of MG132 treated RIF-1 and TR cells. This means heat shock and MG132 share some features of HSR signaling pathway, but at the same time induce distinct stress response signaling pathways. We summarized the heat shock and MG132 induced cellular responses in Figure 6. In cells exposed to heat shock, there is an increase in denatured proteins, which are recognized and renatured by chaperones, or tagged by ubiquitins and undergo proteasomal degradation. In contrast, in cells exposed to MG132, proteasome function is blocked and poly-ubiquitinated proteins probably including newly synthesized misfolded proteins and normal endogenous short-lived proteins accumulate. The higher levels of chaperone and UPS molecules in TR cells indicate that heat shock induced denatured proteins are removed faster in TR cells than in RIF-1 cells. The amounts of poly-ubiquitinated proteins accumulated by MG132 in RIF-1 and TR cells are similar as these cells probably have less efficient clearing systems for the poly-ubiquitinated proteins in normal conditions. The highly expressed UPS molecules in TR cells seem to degrade poly-ubiquitin tagged proteins in stress conditions. This might explain why the gene expression profiles and kinetics of MG132 treated RIF-1 and TR cells are quite similar.

We found, in the cell lines we studied, that heat shock and MG132 up-regulate a multitude of genes, some by both heat shock and MG132; and some only by heat shock or by MG132. Our systemic analysis helps dissect the signaling pathways induced by two types of stress, and contributes to a better understanding of HSR. HSF1 was the major HSF reportedly activated in heat shock treated cells while both HSF1 and HSF2 were activated in MG132 treated cells [43], [44]. Some of the commonly induced genes by both heat shock and MG132 might be downstream genes of HSF1. Table S6 shows that Hspb1, Hspb8, Hspca, Hsp105, Dnaja4, Dnajb1, Cryab and Cryba4 were up-regulated by both heat shock and MG132. On the other hand, MG132 specifically induced genes such as Hspa5, Hspa8, Hspd1, Dnaja2 and Dnajb10 and their induction might require HSF2.

In addition to the groups of up-regulated genes, we also identified individual genes whose expressions specifically increased or decreased more than 3 fold, in response to heat shock and MG132 treatment. Again we sorted them into the three groups mentioned earlier: genes regulated commonly by both heat shock and MG132; genes regulated only by heat shock and genes regulated only by MG132. These are presented in Table S8 and S9. Arc and Rsad2, respectively, are the most highly up-regulated (more than 80 fold) genes in response to heat shock and MG132 treatment. We confirmed their protein levels by Western blot analysis (Figure S6). Arc which is almost undetectable in normal cells is dramatically increased and then decreased, after heat shock treatment. Rsad2, also known as viperin, is also increased in MG132 treated cells. MyD family members such as MyD116, Gadd45 alpha, MyD118 (Gadd45 beta), and Gadd45 gamma are up-regulated by both heat shock and MG132, in agreement with a previous report [45]. This indicates that cellular differentiation processes are closely related to cellular stress responses. Oxidoreductase genes such as Prdx6 and Npn3 were up-regulated only by MG132, while some others such as Cbr3 and Hmox1 were up-regulated by both the stresses (over 3 fold). Immunity related transcription factors such as Stat1, Stat2, Irf7, Klf2, Ifi203, Ifi205, Isgf3g, and Ifi35 were up-regulated more than 3 fold in MG132 treated cells. This supports the finding from the Ontologizer study that MG132 induces genes concerned with cytokine production. Other MG132 induced genes include UPS genes, E1 (Ube1l?), E2 (Ube2l6), E3 ligase (Trim34, Rnf31), and 26S proteasome subunits (Psmd2, 4, 12, 13, 14, Psmc4, 5). They were up-regulated at later time points, RM3 and TM3, while many genes up-regulated by MG132 were up-regulated in earlier time points RM2 and TM2, and are therefore probably involved in feedback loop of proteasome inhibition by MG132. Nineteen genes were down-regulated by both heat shock and MG132 treatment Table S9). Among them, 6 genes are histone H2 family members. Interestingly, chaperone genes, Hspa8 and Hspd1, were down-regulated in heat shock treated RIF-1 cells. They probably help cellular recovery from heat shock stress. Most of these genes were down-regulated at later time points, e.g. mainly RH4, TH3, and TH4. As was the case with specific heat shock induced genes, down-regulation of genes was also faster in TR cells.

In a previous study, *Trinklein et al.* employed microarrays for studying the heat shock response in MEF cells [27]. This allows a cross-comparison of the gene list obtained with ours. Seventy one genes induced by heat shock in MEF cells were found in our Data Set S1. Among them, 11 and 19 genes were also up-regulated by heat shock in RIF-1 or TR cells more than 2 and 1.5 fold, respectively. Dnajb1, Hsp105, Cryab, Hmox1, Ube2f, Ubqln1, Cyr61, Rbm8, Nupr1, Ddit3, and Tmem66 were up-regulated more than 2 fold and Hspa8, Hspe1, Ubc, Slc20a1, E2f3, Dtr, Klf5, and Prkcabp were up-regulated more than 1.5 fold. These genes are confirmed to be heat shock induced genes by both the Myers group and our group. Except for these genes, the differences between the results of the previous study [27] and ours might be due to the fact that they used cDNA gene chips and we used oligonucleotide gene chips in our study [46].

We postulate that HSR can occur via diverse pathways depending on the inducing agents. Thus far, chaperones have been recognized as major sensors for misfolded proteins [16]. Now our study suggests that other substrate specific sensors and signaling molecules, those recognize and deal with abnormal and ubiquitinated proteins, also might exist inside cells. Identifying such additional sensors and defining how they operate should further advance our understanding of HSR.

## Materials and Methods

### Cell lines and treatments

Radiation induced mouse fibrosarcoma cell line, RIF-1, and its thermotolerant variant, TR cell lines, gifts from Dr. Hahn G. M., were grown in RPMI1640 supplemented with 10% fetal bovine serum, 100 µM/ml of streptomycin and 100 units/ml of penicillin G at 37°C in an atmosphere of 5% CO_2_-95% air. For the heat shock, 5×10^6^ cells grown in 10 cm tissue culture dishes were floated in a 45°C water bath for the indicated times. Immediately after heat shock, the medium was replaced with fresh medium. For the MG132 treatment, the cells were treated with 50 µM MG132 or 0.1% DMSO containing media for 1 h, washed once with media, and left in fresh media. The heat shock and MG132 treated cells were recovered at the indicated times in a 37°C incubator.

### Western blot analysis

Heat shock or MG132 treated cells were resolved in 10% SDS-PAGE and transferred onto PVDF membranes. Hsc70/Hsp70 were visualized using anti-Hsc70/Hsp70 antibody (Stressgen, PA) and Hsp27 were visualized using anti-Hsp27 antibody (Santa Cruz biotechnology, Inc., CA). Anti-α-tubulin antibody was obtained from Santa Cruz biotechnology, Inc. and anti-ubiquitin antibody from Chemicon.

### Cell viability and statistical analysis

RIF-1 and TR cells were respectively exposed to heat shock at 45°C for 0, 15, 30, 45 and 60 min and recovered at 37°C at 2 h in fresh media. The recovering cells (20,000) were plated in 96 well E-plate with 200 µl of media and cell growth was monitored by measuring electrical impedance every 30 minute using real-time cell analyzer, xCELLigence (Roche Applied Science, IN). For MG132 treatment, 20,000 cells were plated in 96 well E-plate and their growth was monitored for 24 h. Cells were treated with 0, 15, 30, 50 and 100 µM of MG132 for 1 h and fresh media was added following washing once with media. The plate was returned to the xCELLigence and cell growth was monitored every 30 minute. We applied a linear mixed effects model for the growth index over time as implemented in the nmle R package to examine the growth difference between the samples over time [24], [25]. Data were fitted with the linear model for repeated measurements, Growth index  =  no intercept + b1*Sample + b2*Treatment + b3*Time + b4*(interaction between Treatment and Time) + b5*(interaction between Sample and Time) + a random intercept for each individual. Detailed method is in Materials and Methods S1.

### Profiling of protein synthesis by [^35^S]methionine pulse labeling

Total cellular protein synthesis after heat shock or MG132 treatment was examined by pulse labeling with [^35^S]methionine (3 µCi/ml) in methionine free RPMI 1640 media supplemented with 10% FBS for 1 h during recovery. The labeled proteins were separated on 10% SDS-PAGE gels and autoradiographed by BAS2500 (Fujifilm, Japan).

### Microarray analysis

Global gene expressions in RIF-1 (designated ‘R’) and TR (designated ‘T’) cells exposed to heat shock and MG132 were evaluated using microarray analysis. Cells exposed to heat shock (45°C for 30 min) were designated ‘H’, and the cells treated with MG132 (50 µM for 1 hr) were designated ‘M’. In both cases, the cell viability was about 30%, 24 h after the starting point of exponential growth. The cells were recovered from the stress in fresh media for 4, 12, and 24 h after heat shock, or for 2, 8.5, and 18 h after MG132 treatment. We selected these recovery time points based on the kinetics of the appearance of Hsp70, 3-6 h after heat shock treatment and 1-3 h after MG132 treatment (Figure 1C). We used the following symbols for the various samples: Control RIF-1 and TR cells (without heat shock or MG132 treatment) were designated ‘1’ (DMSO treatment was used as a control for MG132 treatment): The recovery time points were represented as 2, 3, and 4 for 4, 12, and 24 h recovery after heat shock treatments and 2, 8.5, and 18 h recovery after MG132 treatments. We used the concatenated symbols: RH1, RH2, RH3, RH4, TH1, TH2, TH3, and TH4 for heat shock, and RM1, RM2, RM3, RM4, TM1, TM2, TM3, and TM4 for MG132 treatment. Each sample was prepared in biological duplication for microarray analysis.

Total RNAs of cells harvested at each time point were isolated using Trizol reagent (Invitrogen, CA). For quality control, RNA purity and integrity were evaluated by denaturing gel electrophoresis and OD 260/280 ratio, and analyzed on Agilent 2100 Bioanalyzer (Agilent Technologies, CA). Total RNA was amplified and purified using the Ambion Illumina RNA amplification kit (Ambion, TX) to yield biotinylated cRNA according to the manufacturer's instructions. Briefly, 550 ng of total RNA was reverse-transcribed to cDNA using a T7 oligo(dT) primer. Second-strand cRNA was synthesized, *in vitro* transcribed and labeled with biotin-NTP. After purification, the cRNA was quantified using the ND-1000 Spectrophotometer (NanoDrop, NC). Labeled cRNA samples (750 ng) were hybridized to each mouse-8 expression bead array for 16-18 h at 58°C, according to the manufacturer's instructions (Illumina, Inc., CA). Detection of array signal was carried out using Amersham fluorolink streptavidin-Cy3 (GE Healthcare Bio-Sciences, UK) following the bead array manual. Arrays were scanned with an Illumina bead array reader confocal scanner according to the manufacturer's instructions. Array data export processing and analysis were performed using Illumina BeadStudio v3.1.3 (Gene Expression Module v3.3.8). Data analysis was performed for us by Macrogen INC, Korea (Data Set S1 and S2). Briefly, the array data were filtered by detection ≥0.71 (similar to signal to noise) in at least 75% samples (We applied a filtering criterion for data analysis; higher signal value was required to obtain a detection≥0.71). Selected gene signal values were transformed by logarithm and normalized by quantile method. The significance of the expression data was determined using LPE test and fold change in which the null hypothesis was that no difference exists among 2 groups [47]. False discovery rate (FDR) was controlled by adjusting *p*-value using Benjamini-Hochberg algorithm [48]. Data Set S1 contains 12,339 genes which were filtered from raw data. Data Set S2 contains the genes which were up- or down- regulated more than 2 fold with adjusted *p*-value<0.05 during recovery after heat shock or MG132 treatment at least once. Hierarchical cluster analysis was performed using complete linkage and Euclidean distance as a measure of similarity.

### Gene Ontology analysis

Functional classification and enrichment of differentially expressed genes were performed using parent-child method and Benjamini-Hochberg method implemented by Ontologizer 2.1 program (http://compbio.charite.de/index.php/home.html) [49], [50].

### Inhibition assay on RSV replication

For measuring inhibitory effects on RSV replication, HEp-2 cells were plated in 96-well tissue culture plates, and at 90% confluence, the medium was removed and replaced by virus inoculums (MOI  = 0.01) containing serially diluted amounts of ribavirin or MG132 from 0.16 µM to 0.04 µM. Concentrations of MG132 above 0.2 µM showed intolerable cytotoxicity. The cells were incubated for 24 h and the culture supernatant was removed. The cells then were fixed by adding 95% methanol for 5 min. The cell monolayer was blocked with PBS/10% FBS for 30 min and 1∶100 diluted goat anti-RSV antibody conjugated with HRP was added and incubated for 60 min. The plates were washed 5 times with PBS and the substrate solution (5 mg/ml 3-amino-9-ethylcarbazole in acetate buffer, pH 5.2, 30% H_2_O_2_ (v/v)) was added. The yellowish red spots were counted under a microscope.

### GSEA (Gene set enrichment analysis)

We created a gene set catalogue for the analysis of Illumina expression array. Co-regulation gene sets were built using known information about transcription factors and their known or predicted target genes from Transcriptional Regulatory Element Database (TRED: http://rulai.cshl.edu/cgi-bin/TRED/tred.cgi/?process=home) maintained by Cold Spring Harbor Laboratory. Co-regulation gene sets consist of 88 gene sets. Members in each set are genes commonly controlled by known transcription factors. The gene sets were relatively small because the information available about gene regulatory network inside cell is incomplete. However, they were still quite useful for identifying transcription factors influencing differential expression of genes in particular experimental conditions.

Gene set enrichment test was performed using GSEA-p (version 1.0) desktop application downloaded from http://www.broad.mit.edu/GSEA [51], [52]. In this analysis, we excluded the gene sets that contain fewer than 10 genes. Significance of differentially expressed gene sets was estimated using a tag permutation test because the sample numbers are small. This type of permutation creates 1,000 random gene sets and calculates their enrichment scores for creating null distribution needed to calculate significance of actual gene set. Cutoff value for significance was determined as *p*-value of less than 0.05 and FDR of less than 10%.

## Supporting Information

Materials and Methods S1 Supplemental Materials and Methods.
(DOC)

Figure S1 RIF-1 cells were subjected to heat shock at 45°C for 30 min or treated with 25 µM MG132 for 4 h. Cells were recovered at 6 h for the detection of Hsp70 and at 18 h for Hsp27. Cells were analyzed by 2D-gel electrophoresis and Western blot analysis with anti-Hsp70 and Hsp27 antibody (Santa Cruz Biotechnology, Inc., CA, USA).
(PPT)

Figure S2 Quantification of ubiquitinated proteins using Western blot analysis shown in Figure 1D. Ubiquitinated proteins were detected using anti-ubiquitin antibody (Chemicon, MA), HRP conjugated goat-anti mouse secondary antibody (Bio-Rad, CA) and West-oneTM Western Blot Detection system (iNtRON Biotechnology, Korea). The enhanced chemiluminescence (ECL) signal was captured by LAS300 system (Fujifilm, Japan). Each lane was quantified using Multi Gauge V3.0 software. Solid bars: RIF-1 cells; grey bars: TR cells.
(PPT)

Figure S3 Validation of microarray data by real-time RT-PCR. We chose genes up-regulated around 2 fold by heat shock treatment. Template cDNAs in cells treated with heat shock at 45°C for 30 min and recovered at the indicated times were prepared. mRNA levels of Hspca, Hsp105 and Bag3 genes were analyzed and normalized using control mRNA, GAPDH. We used primers 5′-AGAACATCATCCCTGCATCC-3′ and 5′-CACATTGGGGGTAGGAACAC-3′ for GAPDH, 5′-TCCAAAGTCCCGAGAACAAC-3′ and 5′-CAGAATGTGATTGGGCACTG-3′ for Hspca, 5′-GGTCCCAATGAAAAATGGTG-3′ and 5′-TCAGCAGCATGGCTGTTATC-3′ for Hsp105 and 5′-AAGTCACCTCCTCCTGCTGA-3′ and 5′-TCTGTTCTGCAGCCACATTC-3′ for Bag3. As in microarray data, fold inductions were presented.
(PPT)

Figure S4 Expression kinetics? of genes up-regulated by heat shock and MG132. Expression patterns of genes up-regulated by both heat shock and MG132 in common (A), genes up-regulated by heat shock alone (B), and genes up-regulated by MG132 alone (C). Their fold changes are represented according to the color scale.
(PPT)

Figure S5 Validation of predicted transcription factors by GSEA. The transcription factors possibly activated in heat shock and MG132 treated cells (Figure 6) were examined using luciferase assay. Plasmid DNAs containing luciferase genes under control of the STAT3 (m67-luc) and ATF2 (3X CRE-luc) were transiently transfected in RIF-1 cells. After treatment with 50 µM of MG132 for 1 h and recovery at the indicated times, cells were lysed and luciferase activites were measured using Dual-Luciferase® Reporter Assay System (Promega, WI). We used Renilla luciferase as a control reporter gene.
(PPT)

Figure S6 Confirmation of microarray findings in protein expression levels. Cells treated with heat shock at 45°C for 30 min or with 50 µM MG132 for 1 h were recovered in fresh media at 37°C at the indicated times. Arc and Rsad2, the most up-regulated genes in gene chip data, were analyzed by Western blot analysis using anti-Arc (Santa Cruz, CA) and anti-Rsad2 (Abcam, MA) antibody, respectively.
(PPT)

Table S1 Genes up-regulated more than 3-fold in TR cells compared to RIF-1 cells.
(PPT)

Table S2 Genes down-regulated more than 3-fold in TR cells compared to RIF-1 cells.
(PPT)

Table S3 Summary of the linear mixed effect model of growth index (heat shock: sample size of 3630 from 30 objects, MG132: sample size of 2430 from 30 objects)
(PPT)

Table S4 Over-represented gene ontology categories of the down-regulated genes in heat shock treated RIF-1 cells.
(PPT)

Table S5 Over-represented gene ontology categories of the down-regulated genes in MG132 treated RIF-1 cells.
(PPT)

Table S6 Heat shock protein family members are listed with their mRNA levels in response to heat shock and MG132 treatment. Fold changes more than 2 are colored in red and less than -2 are colored in green.
(PPT)

Table S7 Histone family members are listed with their mRNA levels in response to heat shock and MG132 treatment. Fold changes more than 2 are colored in red and less than -2 are colored in green.
(PPT)

Table S8 Heat shock and MG132 induced genes more than 3 fold in comparison to control cells are listed. Fold changes more than 2 are colored in red and less than -2 are colored in green. T/R means fold differences in TR cells compared to RIF-1 cells.
(PPT)

Table S9 Heat shock and MG132 suppressed genes more than 3 fold in comparison to control cells are listed. Fold changes more than 2 are colored in red and less than -2 are colored in green. T/R means fold differences in TR cells compared to RIF-1 cells.
(PPT)

Table S10 Innate immunity signaling genes are listed with their mRNA levels in response to heat shock and MG132 treatment. Fold changes more than 2 are colored in red and less than -2 are colored in green.
(PPT)

Table S11 Predicted transcription factors possibly activated in heat shock or MG132 treated RIF-1 and TR cells.
(PPT)

Data Set S1 Global gene expressions in RIF-1 and TR cells exposed to heat shock and MG132 obtained from microarray analysis. Fold changes of 12,339 genes that passed initial screening of the microarray data are shown.
(XLSX)

Data Set S2 Among the genes in this Data Set S1, 2,208 genes which changed more than 2 fold with *p*-value<0.05 are listed.
(XLSX)

Data Set S3 Genes in Figure 4 are listed with their fold changes. Genes were grouped into three groups based on their functions: (a) those up-regulated by both heat shock and MG132; (b) those up-regulated only by heat shock, and (c) those up-regulated only by MG132.
(XLSX)
