# Supplementary material for: Systemic Analysis of Heat Shock Response Induced by Heat Shock and a Proteasome Inhibitor MG132
Source: PLoS One. 2011 Jun 30;6(6):e20252. doi: 10.1371/journal.pone.0020252 (PMC3127947; doi:10.1371/journal.pone.0020252)
Supplement: Materials and Methods S1 — Supplemental Materials and Methods. (DOC) [file pone.0020252.s001.doc]

**Supplemental materials and methods**

**2D gel electrophoresis**

The cells was harvested and solubilized in buffer containing 8.4 M urea, 2.4 M thiourea, 5% CHAPS, 1.6% pharmalyte 5-7, 0.4% pharmalyte 3-10, 50 mM DTT, protease inhibitor cocktail, and 5 mM Na_3_VO_4_. Equal amounts of proteins (50 µg) were loaded into the rehydrated dry strip during first dimensional electrophoresis. Immobline Drystrip 4-7 linear gels (7 cm) were used. After electrofocusing, the strips were shaken for 20 min with an equilibration buffer (50 mM tris (pH 8.8), 6 M urea 2% SDS, 30% glycerol), placed onto 11% second dimensional SDS-PAGE slab gels and sealed with a solution of 0.05% agarose.

**Statistical analysis of the effects of heat shock and MG132 on cell growth**

After testing several possible models, we choose the linear mixed effect model with two distinct effects: fixed effects (sample, treatment, time, interaction between treatment and time, and interaction between sample and time) and mixed effect (a random intercept). Growth index = no intercept + b1*Sample + b2*Treatment + b3*Time + b4*(interaction between Treatment and Time) + b5*(interaction between Sample and Time) + a random intercept for each individual. We are mostly interested in the interaction term between sample and time, i.e. b5. After adjusting by covariates, it would explain how the slope differs between sample RIF-1 and TR. The results of our analysis suggest that the slope between samples is statistically different even the magnitude of changes are not large. The detailed explanation of each term is as follows:

Main term :

b1 : overall differences for sample R and T, or the population’s average intercept.

b2 : overall differences between treatments (baseline = treatment 0).

b3 : overall slope. Interestingly, the estimated coefficients are similar, in both samples (R and T) and we may expect the overall trend over time is quite similar (0.079, 0.078) between treatments. It can be interpreted as the growth index is 0.079 positively increased with one time unit change (1 hr).

Interaction term

b4 : how the slope is different across several treatments. Since increasing treatment time is severe to the objects, we easily expect the slope difference is estimated negatively (baseline=Treatment0). For example, at Treatment60 and Sample R in heat-shock case, the final slope is almost zero (0.079 – 0.080 = -0.001).

b5 : It is of our interest. This illustrates how the slope can be different between the samples (cells). We focused on whether a different linear trend exists between the samples even after adjusting by the time trend (b3). If it does, b5 would indicate the difference between the samples.
