# Supplementary figures and images for: Systemic Analysis of Heat Shock Response Induced by Heat Shock and a Proteasome Inhibitor MG132
Source: PLoS One. 2011 Jun 30;6(6):e20252. doi: 10.1371/journal.pone.0020252 (PMC3127947; doi:10.1371/journal.pone.0020252)

## Slide 1
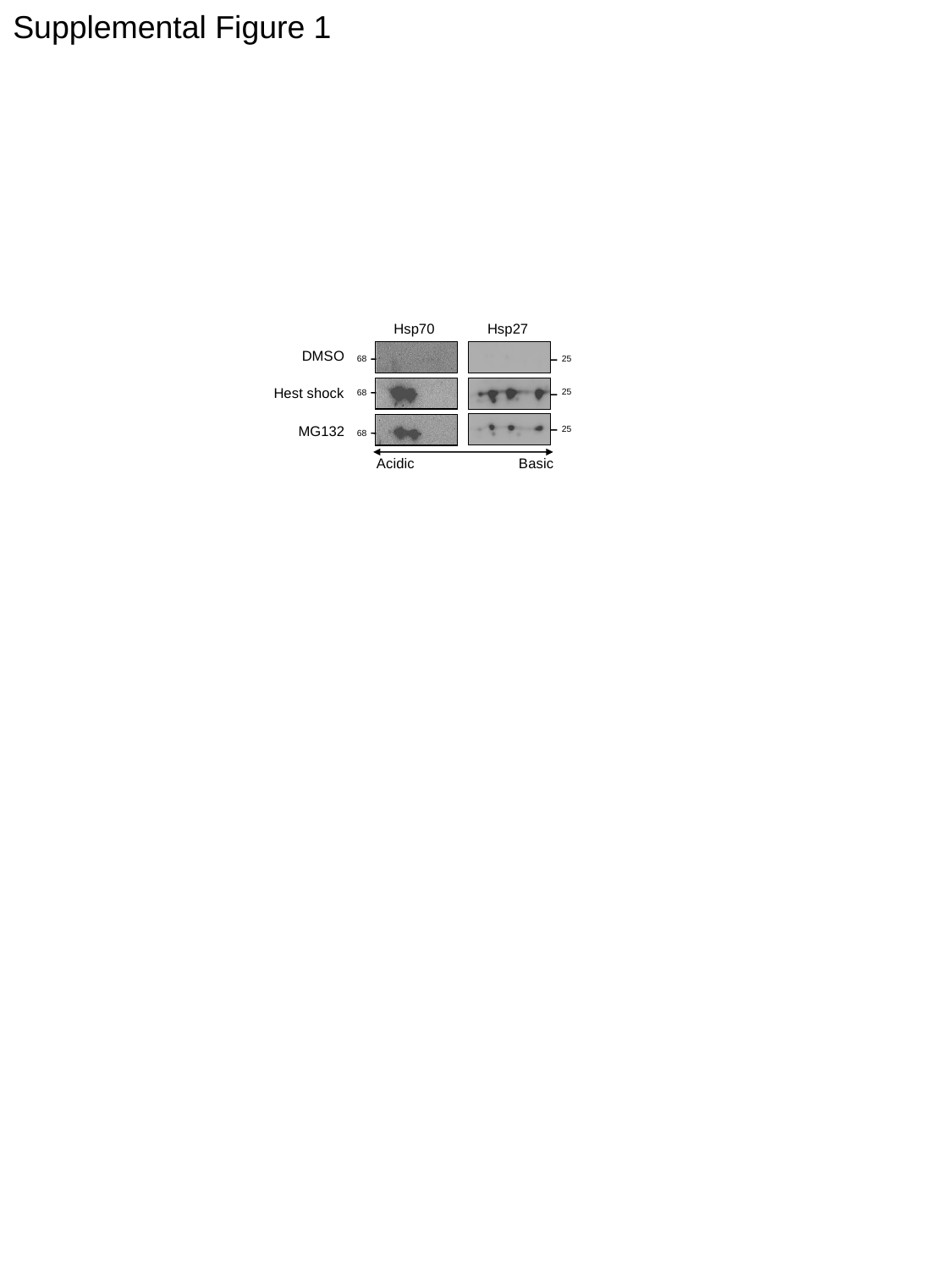

Supplemental Figure 1
Hsp70
Hsp27
DMSO
68
25
Hest shock
25
68
MG132
25
68
Acidic Basic

Supplement: Figure S1 — RIF-1 cells were subjected to heat shock at 45°C for 30 min or treated with 25 µM MG132 for 4 h. Cells were recovered at 6 h for the detection of Hsp70 and at 18 h for Hsp27. Cells were analyzed by 2D-gel electrophoresis and Western blot analysis with anti-Hsp70 and Hsp27 antibody (Santa Cruz Biotechnology, Inc., CA, USA). (PPT) [file pone.0020252.s002.ppt]

## Slide 1
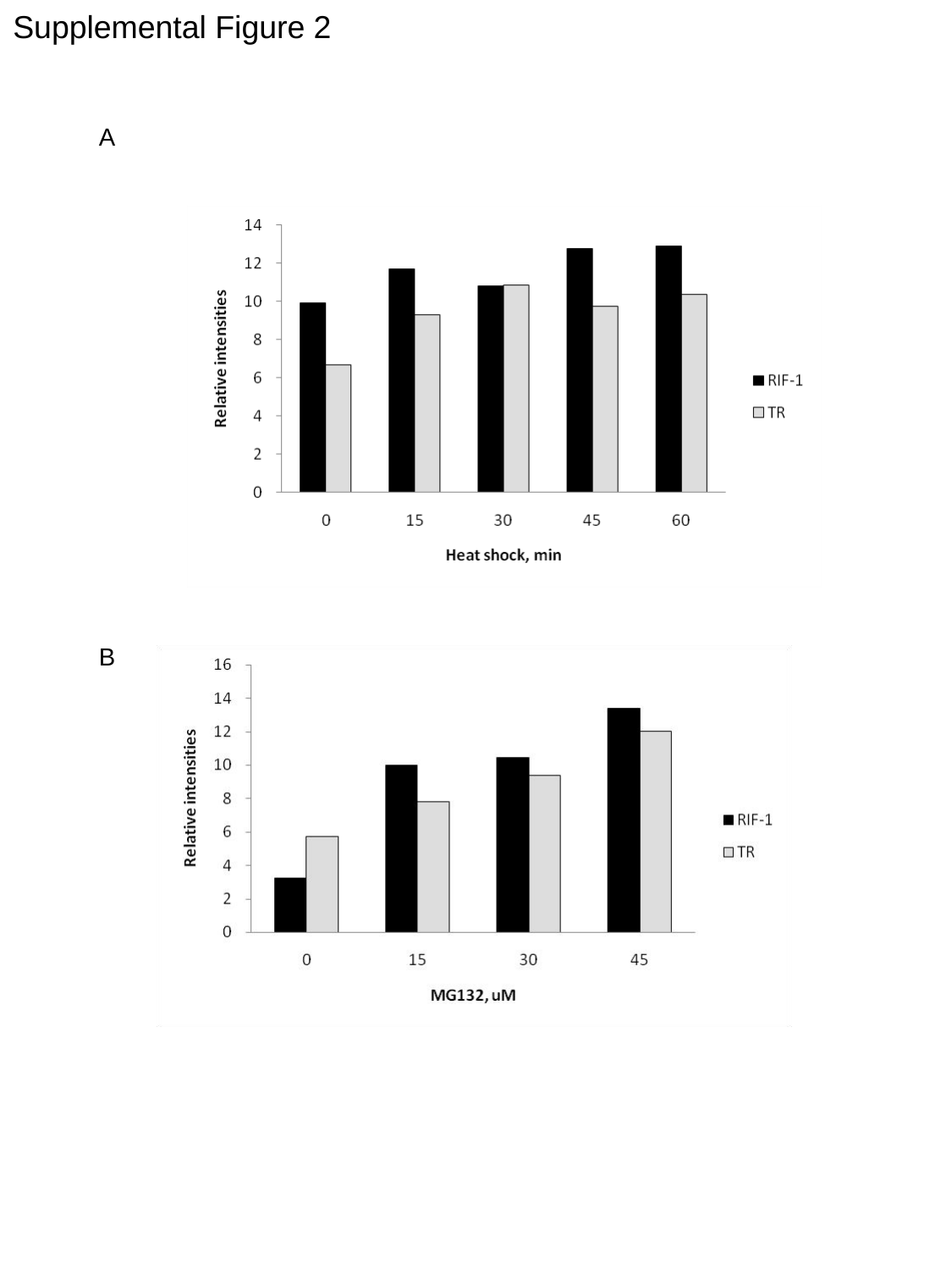

Supplemental Figure 2
A
B

Supplement: Figure S2 — Quantification of ubiquitinated proteins using Western blot analysis shown in Figure 1D. Ubiquitinated proteins were detected using anti-ubiquitin antibody (Chemicon, MA), HRP conjugated goat-anti mouse secondary antibody (Bio-Rad, CA) and West-oneTM Western Blot Detection system (iNtRON Biotechnology, Korea). The enhanced chemiluminescence (ECL) signal was captured by LAS300 system (Fujifilm, Japan). Each lane was quantified using Multi Gauge V3.0 software. Solid bars: RIF-1 cells; grey bars: TR cells. (PPT) [file pone.0020252.s003.ppt]

## Slide 1
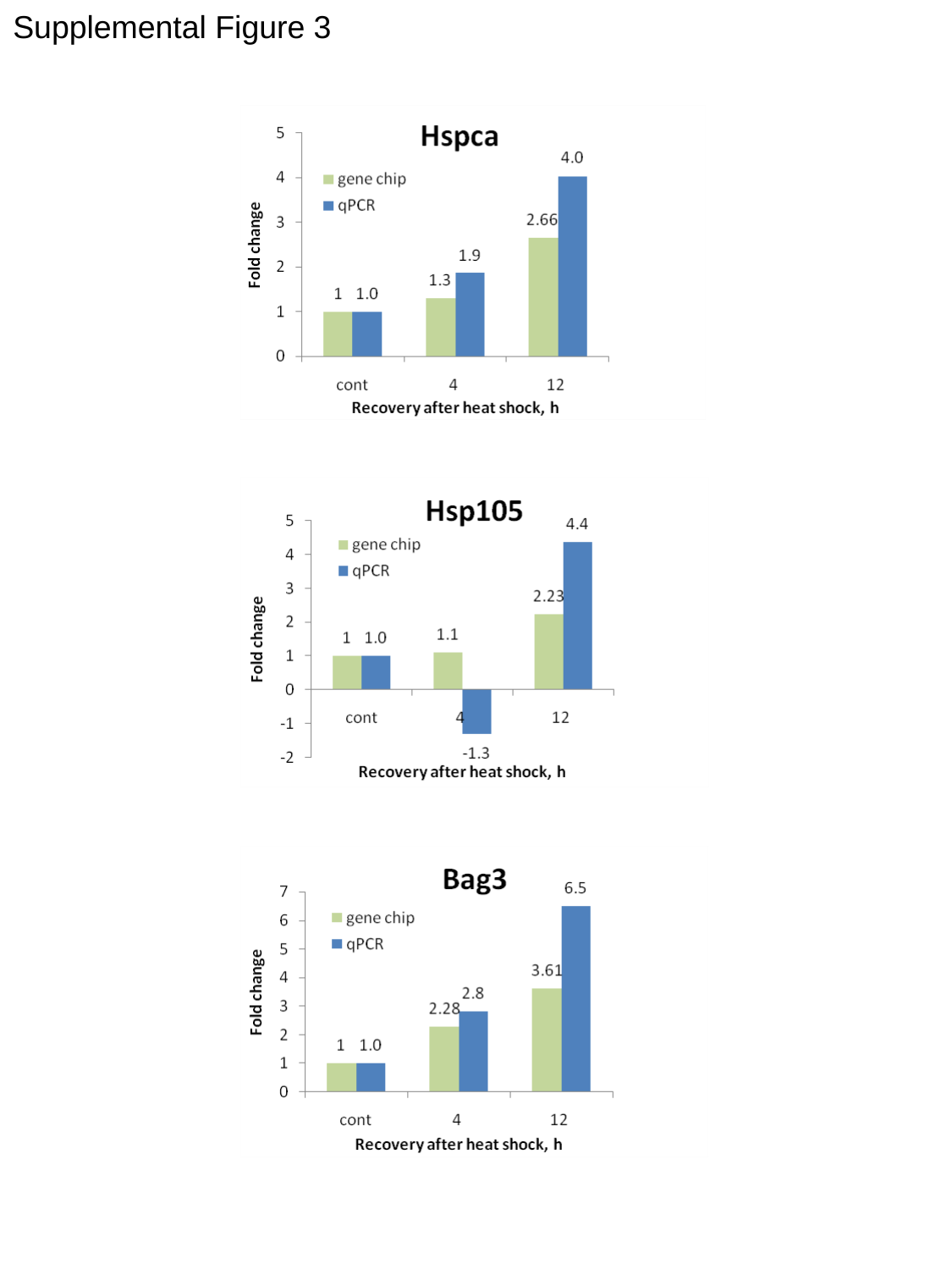

Supplemental Figure 3

Supplement: Figure S3 — Validation of microarray data by real-time RT-PCR. We chose genes up-regulated around 2 fold by heat shock treatment. Template cDNAs in cells treated with heat shock at 45°C for 30 min and recovered at the indicated times were prepared. mRNA levels of Hspca, Hsp105 and Bag3 genes were analyzed and normalized using control mRNA, GAPDH. We used primers 5′-AGAACATCATCCCTGCATCC-3′ and 5′-CACATTGGGGGTAGGAACAC-3′ for GAPDH, 5′-TCCAAAGTCCCGAGAACAAC-3′ and 5′-CAGAATGTGATTGGGCACTG-3′ for Hspca, 5′-GGTCCCAATGAAAAATGGTG-3′ and 5′-TCAGCAGCATGGCTGTTATC-3′ for Hsp105 and 5′-AAGTCACCTCCTCCTGCTGA-3′ and 5′-TCTGTTCTGCAGCCACATTC-3′ for Bag3. As in microarray data, fold inductions were presented. (PPT) [file pone.0020252.s004.ppt]

## Slide 1
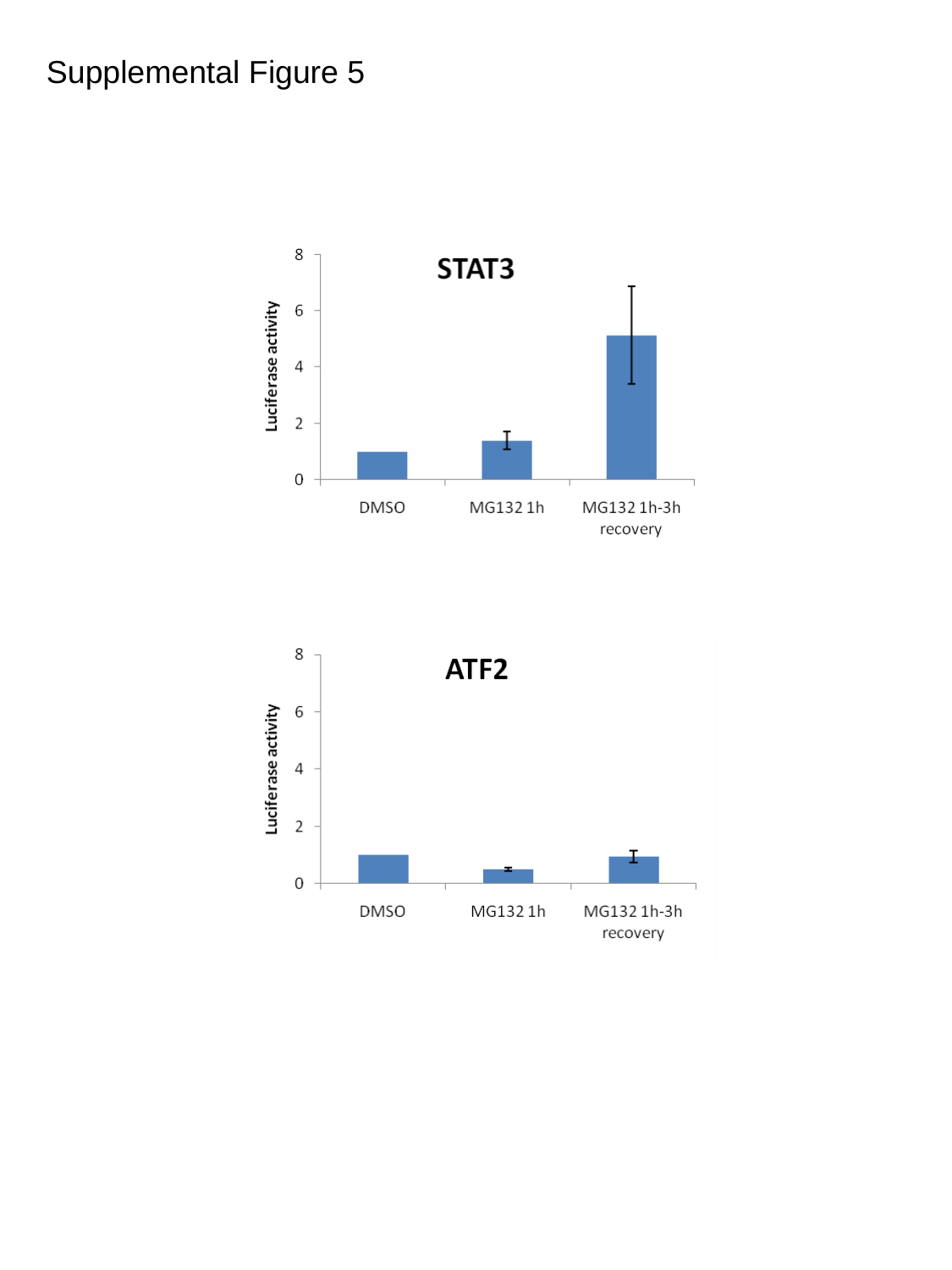

Supplemental Figure 5

Supplement: Figure S5 — Validation of predicted transcription factors by GSEA. The transcription factors possibly activated in heat shock and MG132 treated cells (Figure 6) were examined using luciferase assay. Plasmid DNAs containing luciferase genes under control of the STAT3 (m67-luc) and ATF2 (3X CRE-luc) were transiently transfected in RIF-1 cells. After treatment with 50 µM of MG132 for 1 h and recovery at the indicated times, cells were lysed and luciferase activites were measured using Dual-Luciferase® Reporter Assay System (Promega, WI). We used Renilla luciferase as a control reporter gene. (PPT) [file pone.0020252.s006.ppt]

## Slide 1
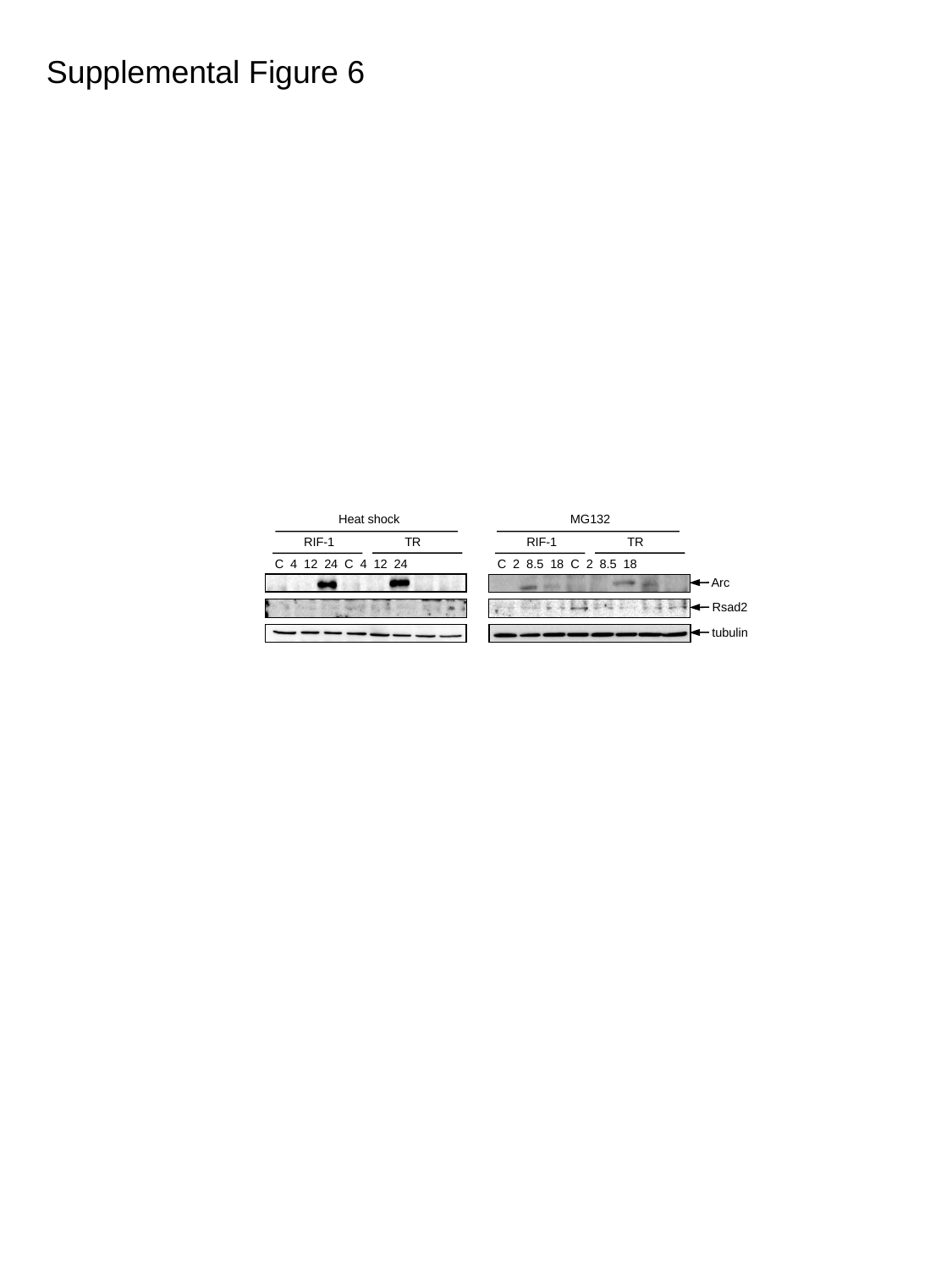

Supplemental Figure 6
Heat shock
MG132
RIF-1
TR
RIF-1
TR
C 4 12 24 C 4 12 24
C 2 8.5 18 C 2 8.5 18
Arc
Rsad2
tubulin

Supplement: Figure S6 — Confirmation of microarray findings in protein expression levels. Cells treated with heat shock at 45°C for 30 min or with 50 µM MG132 for 1 h were recovered in fresh media at 37°C at the indicated times. Arc and Rsad2, the most up-regulated genes in gene chip data, were analyzed by Western blot analysis using anti-Arc (Santa Cruz, CA) and anti-Rsad2 (Abcam, MA) antibody, respectively. (PPT) [file pone.0020252.s007.ppt]
