# Supplementary material for: Systemic Analysis of Heat Shock Response Induced by Heat Shock and a Proteasome Inhibitor MG132
Source: PLoS One. 2011 Jun 30;6(6):e20252. doi: 10.1371/journal.pone.0020252 (PMC3127947; doi:10.1371/journal.pone.0020252)
Supplement: Figure S4 — Expression kinetics? of genes up-regulated by heat shock and MG132. Expression patterns of genes up-regulated by both heat shock and MG132 in common (A), genes up-regulated by heat shock alone (B), and genes up-regulated by MG132 alone (C). Their fold changes are represented according to the color scale. (PPT) [file pone.0020252.s005.ppt]

## Slide 1
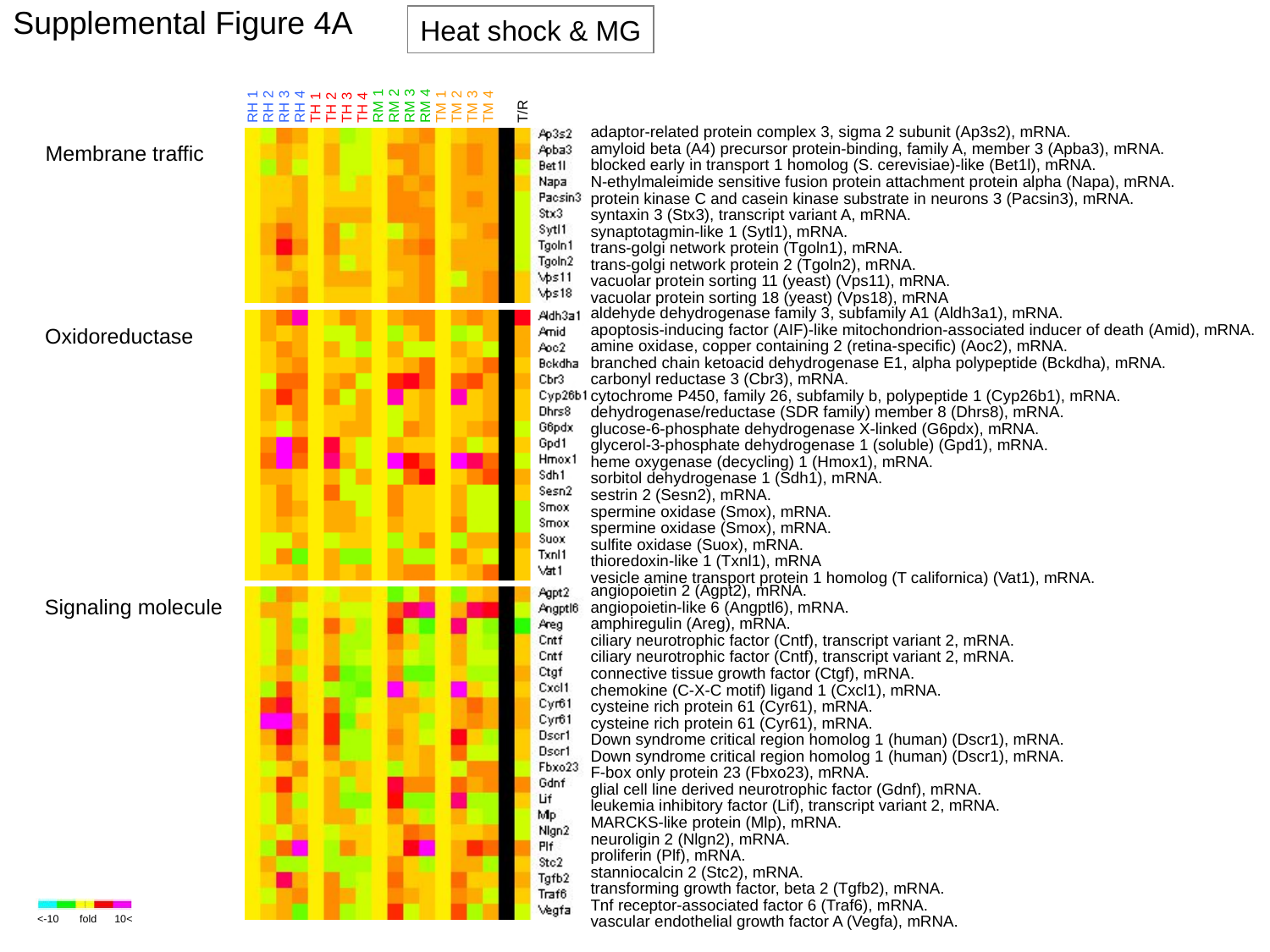

RH 1
RH 2
RH 3
RH 4
TH 1
TH 2
TH 3
TH 4
RM 1
RM 2
RM 3
RM 4
TM 1
TM 2
TM 3
TM 4
Supplemental Figure 4A
Heat shock & MG
T/R
adaptor-related protein complex 3, sigma 2 subunit (Ap3s2), mRNA.
amyloid beta (A4) precursor protein-binding, family A, member 3 (Apba3), mRNA.
blocked early in transport 1 homolog (S. cerevisiae)-like (Bet1l), mRNA.
N-ethylmaleimide sensitive fusion protein attachment protein alpha (Napa), mRNA.
protein kinase C and casein kinase substrate in neurons 3 (Pacsin3), mRNA.
syntaxin 3 (Stx3), transcript variant A, mRNA.
synaptotagmin-like 1 (Sytl1), mRNA.
trans-golgi network protein (Tgoln1), mRNA.
trans-golgi network protein 2 (Tgoln2), mRNA.
vacuolar protein sorting 11 (yeast) (Vps11), mRNA.
vacuolar protein sorting 18 (yeast) (Vps18), mRNA
Membrane traffic
aldehyde dehydrogenase family 3, subfamily A1 (Aldh3a1), mRNA.
apoptosis-inducing factor (AIF)-like mitochondrion-associated inducer of death (Amid), mRNA.
amine oxidase, copper containing 2 (retina-specific) (Aoc2), mRNA.
branched chain ketoacid dehydrogenase E1, alpha polypeptide (Bckdha), mRNA.
carbonyl reductase 3 (Cbr3), mRNA.
cytochrome P450, family 26, subfamily b, polypeptide 1 (Cyp26b1), mRNA.
dehydrogenase/reductase (SDR family) member 8 (Dhrs8), mRNA.
glucose-6-phosphate dehydrogenase X-linked (G6pdx), mRNA.
glycerol-3-phosphate dehydrogenase 1 (soluble) (Gpd1), mRNA.
heme oxygenase (decycling) 1 (Hmox1), mRNA.
sorbitol dehydrogenase 1 (Sdh1), mRNA.
sestrin 2 (Sesn2), mRNA.
spermine oxidase (Smox), mRNA.
spermine oxidase (Smox), mRNA.
sulfite oxidase (Suox), mRNA.
thioredoxin-like 1 (Txnl1), mRNA
vesicle amine transport protein 1 homolog (T californica) (Vat1), mRNA.
Oxidoreductase
angiopoietin 2 (Agpt2), mRNA.
angiopoietin-like 6 (Angptl6), mRNA.
amphiregulin (Areg), mRNA.
ciliary neurotrophic factor (Cntf), transcript variant 2, mRNA.
ciliary neurotrophic factor (Cntf), transcript variant 2, mRNA.
connective tissue growth factor (Ctgf), mRNA.
chemokine (C-X-C motif) ligand 1 (Cxcl1), mRNA.
cysteine rich protein 61 (Cyr61), mRNA.
cysteine rich protein 61 (Cyr61), mRNA.
Down syndrome critical region homolog 1 (human) (Dscr1), mRNA.
Down syndrome critical region homolog 1 (human) (Dscr1), mRNA.
F-box only protein 23 (Fbxo23), mRNA.
glial cell line derived neurotrophic factor (Gdnf), mRNA.
leukemia inhibitory factor (Lif), transcript variant 2, mRNA.
MARCKS-like protein (Mlp), mRNA.
neuroligin 2 (Nlgn2), mRNA.
proliferin (Plf), mRNA.
stanniocalcin 2 (Stc2), mRNA.
transforming growth factor, beta 2 (Tgfb2), mRNA.
Tnf receptor-associated factor 6 (Traf6), mRNA.
vascular endothelial growth factor A (Vegfa), mRNA.
Signaling molecule
<-10 fold 10<

## Slide 2
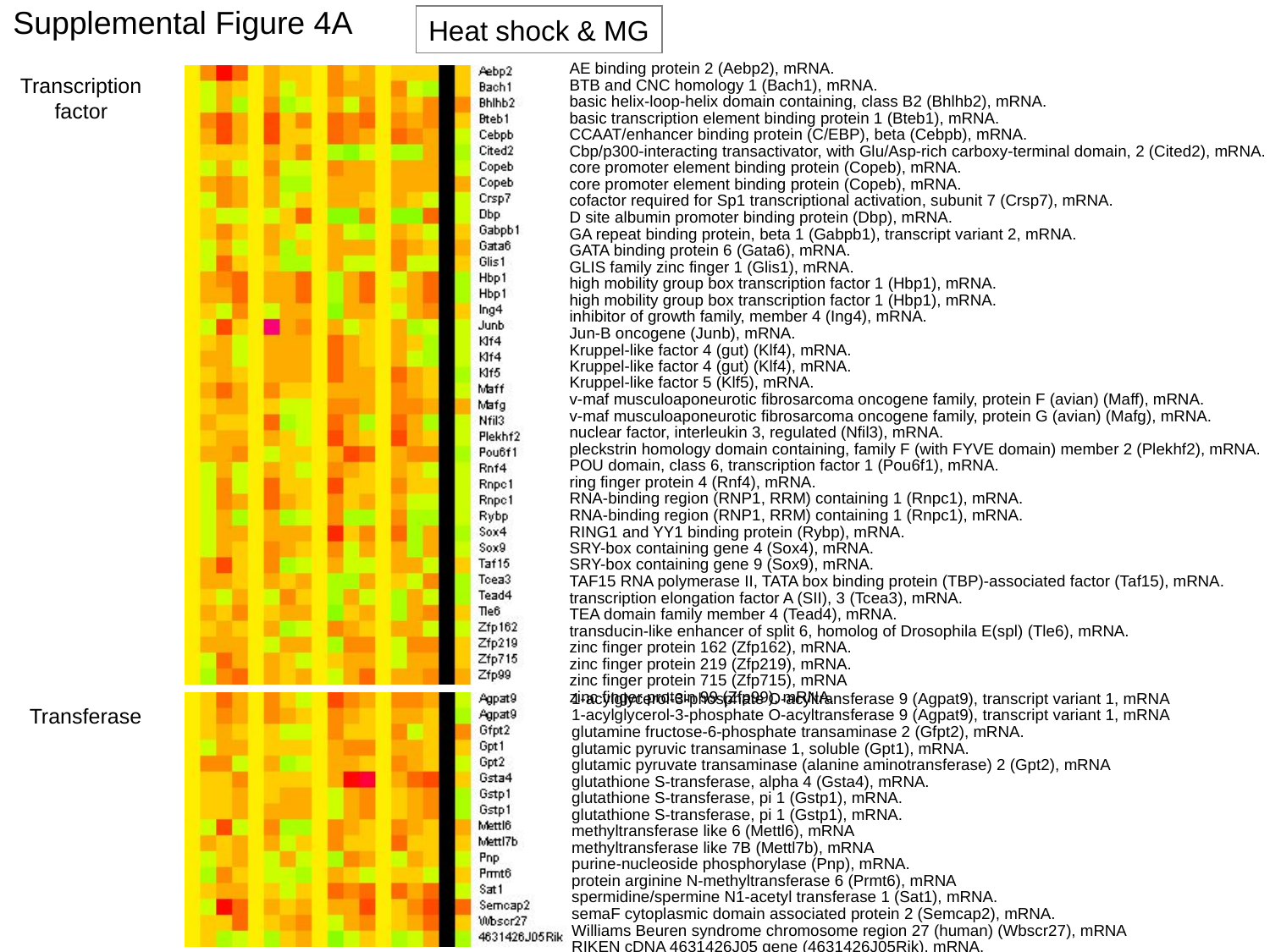

Supplemental Figure 4A
Heat shock & MG
AE binding protein 2 (Aebp2), mRNA.
BTB and CNC homology 1 (Bach1), mRNA.
basic helix-loop-helix domain containing, class B2 (Bhlhb2), mRNA.
basic transcription element binding protein 1 (Bteb1), mRNA.
CCAAT/enhancer binding protein (C/EBP), beta (Cebpb), mRNA.
Cbp/p300-interacting transactivator, with Glu/Asp-rich carboxy-terminal domain, 2 (Cited2), mRNA.
core promoter element binding protein (Copeb), mRNA.
core promoter element binding protein (Copeb), mRNA.
cofactor required for Sp1 transcriptional activation, subunit 7 (Crsp7), mRNA.
D site albumin promoter binding protein (Dbp), mRNA.
GA repeat binding protein, beta 1 (Gabpb1), transcript variant 2, mRNA.
GATA binding protein 6 (Gata6), mRNA.
GLIS family zinc finger 1 (Glis1), mRNA.
high mobility group box transcription factor 1 (Hbp1), mRNA.
high mobility group box transcription factor 1 (Hbp1), mRNA.
inhibitor of growth family, member 4 (Ing4), mRNA.
Jun-B oncogene (Junb), mRNA.
Kruppel-like factor 4 (gut) (Klf4), mRNA.
Kruppel-like factor 4 (gut) (Klf4), mRNA.
Kruppel-like factor 5 (Klf5), mRNA.
v-maf musculoaponeurotic fibrosarcoma oncogene family, protein F (avian) (Maff), mRNA.
v-maf musculoaponeurotic fibrosarcoma oncogene family, protein G (avian) (Mafg), mRNA.
nuclear factor, interleukin 3, regulated (Nfil3), mRNA.
pleckstrin homology domain containing, family F (with FYVE domain) member 2 (Plekhf2), mRNA.
POU domain, class 6, transcription factor 1 (Pou6f1), mRNA.
ring finger protein 4 (Rnf4), mRNA.
RNA-binding region (RNP1, RRM) containing 1 (Rnpc1), mRNA.
RNA-binding region (RNP1, RRM) containing 1 (Rnpc1), mRNA.
RING1 and YY1 binding protein (Rybp), mRNA.
SRY-box containing gene 4 (Sox4), mRNA.
SRY-box containing gene 9 (Sox9), mRNA.
TAF15 RNA polymerase II, TATA box binding protein (TBP)-associated factor (Taf15), mRNA.
transcription elongation factor A (SII), 3 (Tcea3), mRNA.
TEA domain family member 4 (Tead4), mRNA.
transducin-like enhancer of split 6, homolog of Drosophila E(spl) (Tle6), mRNA.
zinc finger protein 162 (Zfp162), mRNA.
zinc finger protein 219 (Zfp219), mRNA.
zinc finger protein 715 (Zfp715), mRNA
zinc finger protein 99 (Zfp99), mRNA.
Transcription
factor
1-acylglycerol-3-phosphate O-acyltransferase 9 (Agpat9), transcript variant 1, mRNA
1-acylglycerol-3-phosphate O-acyltransferase 9 (Agpat9), transcript variant 1, mRNA
glutamine fructose-6-phosphate transaminase 2 (Gfpt2), mRNA.
glutamic pyruvic transaminase 1, soluble (Gpt1), mRNA.
glutamic pyruvate transaminase (alanine aminotransferase) 2 (Gpt2), mRNA
glutathione S-transferase, alpha 4 (Gsta4), mRNA.
glutathione S-transferase, pi 1 (Gstp1), mRNA.
glutathione S-transferase, pi 1 (Gstp1), mRNA.
methyltransferase like 6 (Mettl6), mRNA
methyltransferase like 7B (Mettl7b), mRNA
purine-nucleoside phosphorylase (Pnp), mRNA.
protein arginine N-methyltransferase 6 (Prmt6), mRNA
spermidine/spermine N1-acetyl transferase 1 (Sat1), mRNA.
semaF cytoplasmic domain associated protein 2 (Semcap2), mRNA.
Williams Beuren syndrome chromosome region 27 (human) (Wbscr27), mRNA
RIKEN cDNA 4631426J05 gene (4631426J05Rik), mRNA.
Transferase

## Slide 3
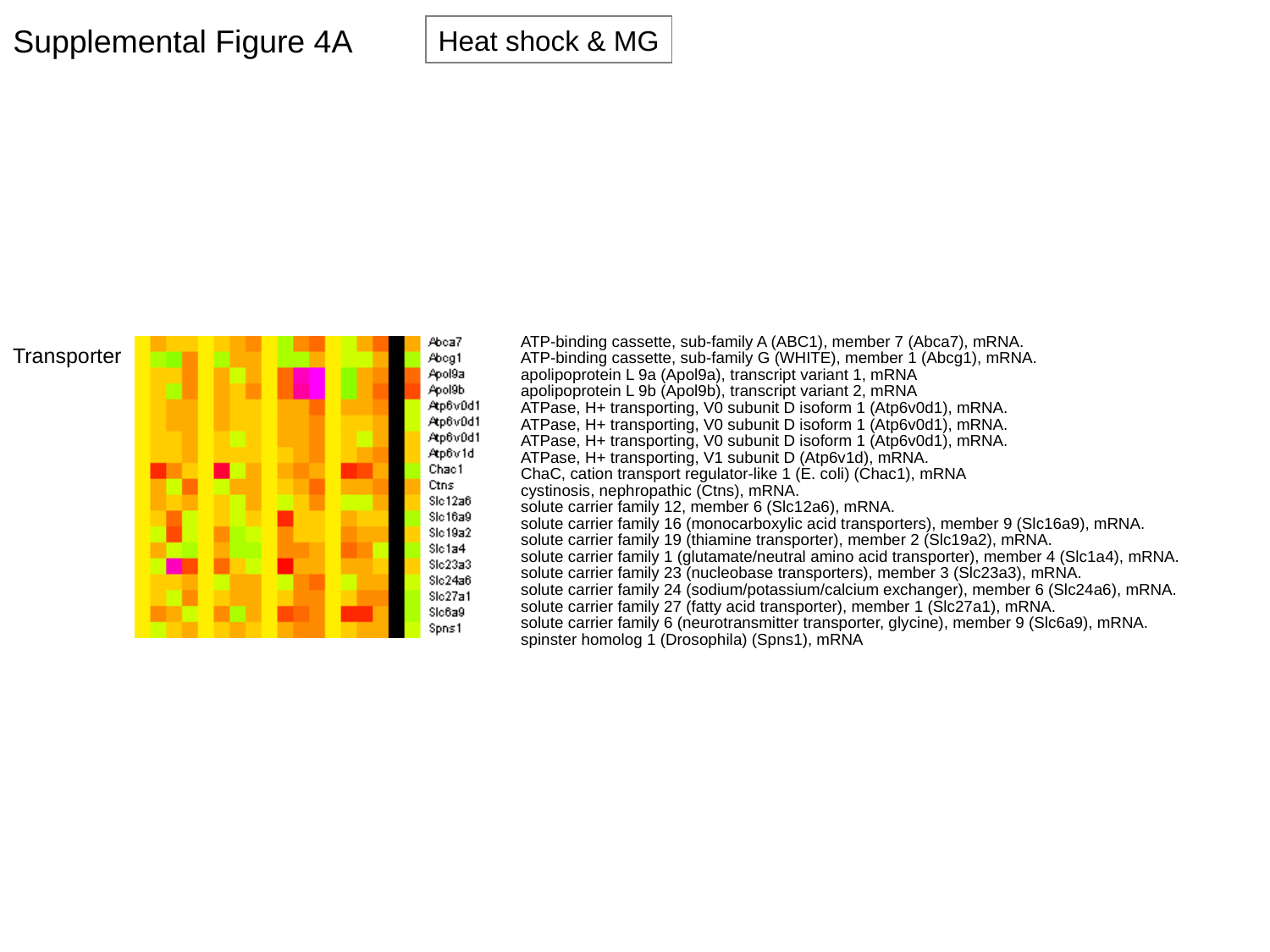

Supplemental Figure 4A
Heat shock & MG
ATP-binding cassette, sub-family A (ABC1), member 7 (Abca7), mRNA.
ATP-binding cassette, sub-family G (WHITE), member 1 (Abcg1), mRNA.
apolipoprotein L 9a (Apol9a), transcript variant 1, mRNA
apolipoprotein L 9b (Apol9b), transcript variant 2, mRNA
ATPase, H+ transporting, V0 subunit D isoform 1 (Atp6v0d1), mRNA.
ATPase, H+ transporting, V0 subunit D isoform 1 (Atp6v0d1), mRNA.
ATPase, H+ transporting, V0 subunit D isoform 1 (Atp6v0d1), mRNA.
ATPase, H+ transporting, V1 subunit D (Atp6v1d), mRNA.
ChaC, cation transport regulator-like 1 (E. coli) (Chac1), mRNA
cystinosis, nephropathic (Ctns), mRNA.
solute carrier family 12, member 6 (Slc12a6), mRNA.
solute carrier family 16 (monocarboxylic acid transporters), member 9 (Slc16a9), mRNA.
solute carrier family 19 (thiamine transporter), member 2 (Slc19a2), mRNA.
solute carrier family 1 (glutamate/neutral amino acid transporter), member 4 (Slc1a4), mRNA.
solute carrier family 23 (nucleobase transporters), member 3 (Slc23a3), mRNA.
solute carrier family 24 (sodium/potassium/calcium exchanger), member 6 (Slc24a6), mRNA.
solute carrier family 27 (fatty acid transporter), member 1 (Slc27a1), mRNA.
solute carrier family 6 (neurotransmitter transporter, glycine), member 9 (Slc6a9), mRNA.
spinster homolog 1 (Drosophila) (Spns1), mRNA
Transporter

## Slide 4
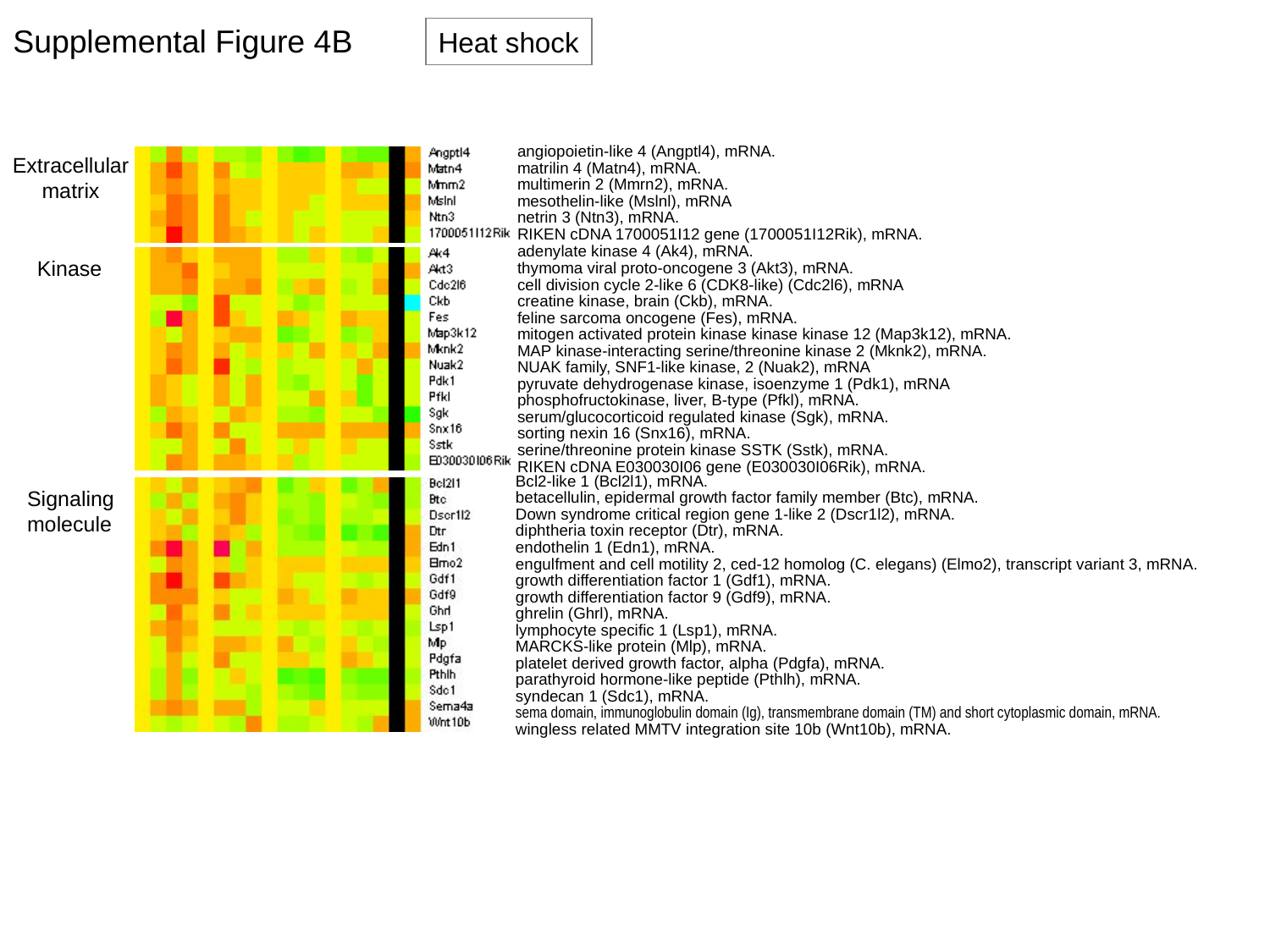

Supplemental Figure 4B
Heat shock
Extracellular
matrix
angiopoietin-like 4 (Angptl4), mRNA.
matrilin 4 (Matn4), mRNA.
multimerin 2 (Mmrn2), mRNA.
mesothelin-like (Mslnl), mRNA
netrin 3 (Ntn3), mRNA.
RIKEN cDNA 1700051I12 gene (1700051I12Rik), mRNA.
adenylate kinase 4 (Ak4), mRNA.
thymoma viral proto-oncogene 3 (Akt3), mRNA.
cell division cycle 2-like 6 (CDK8-like) (Cdc2l6), mRNA
creatine kinase, brain (Ckb), mRNA.
feline sarcoma oncogene (Fes), mRNA.
mitogen activated protein kinase kinase kinase 12 (Map3k12), mRNA.
MAP kinase-interacting serine/threonine kinase 2 (Mknk2), mRNA.
NUAK family, SNF1-like kinase, 2 (Nuak2), mRNA
pyruvate dehydrogenase kinase, isoenzyme 1 (Pdk1), mRNA
phosphofructokinase, liver, B-type (Pfkl), mRNA.
serum/glucocorticoid regulated kinase (Sgk), mRNA.
sorting nexin 16 (Snx16), mRNA.
serine/threonine protein kinase SSTK (Sstk), mRNA.
RIKEN cDNA E030030I06 gene (E030030I06Rik), mRNA.
Kinase
Bcl2-like 1 (Bcl2l1), mRNA.
betacellulin, epidermal growth factor family member (Btc), mRNA.
Down syndrome critical region gene 1-like 2 (Dscr1l2), mRNA.
diphtheria toxin receptor (Dtr), mRNA.
endothelin 1 (Edn1), mRNA.
engulfment and cell motility 2, ced-12 homolog (C. elegans) (Elmo2), transcript variant 3, mRNA.
growth differentiation factor 1 (Gdf1), mRNA.
growth differentiation factor 9 (Gdf9), mRNA.
ghrelin (Ghrl), mRNA.
lymphocyte specific 1 (Lsp1), mRNA.
MARCKS-like protein (Mlp), mRNA.
platelet derived growth factor, alpha (Pdgfa), mRNA.
parathyroid hormone-like peptide (Pthlh), mRNA.
syndecan 1 (Sdc1), mRNA.
sema domain, immunoglobulin domain (Ig), transmembrane domain (TM) and short cytoplasmic domain, mRNA.
wingless related MMTV integration site 10b (Wnt10b), mRNA.
Signaling
molecule

## Slide 5
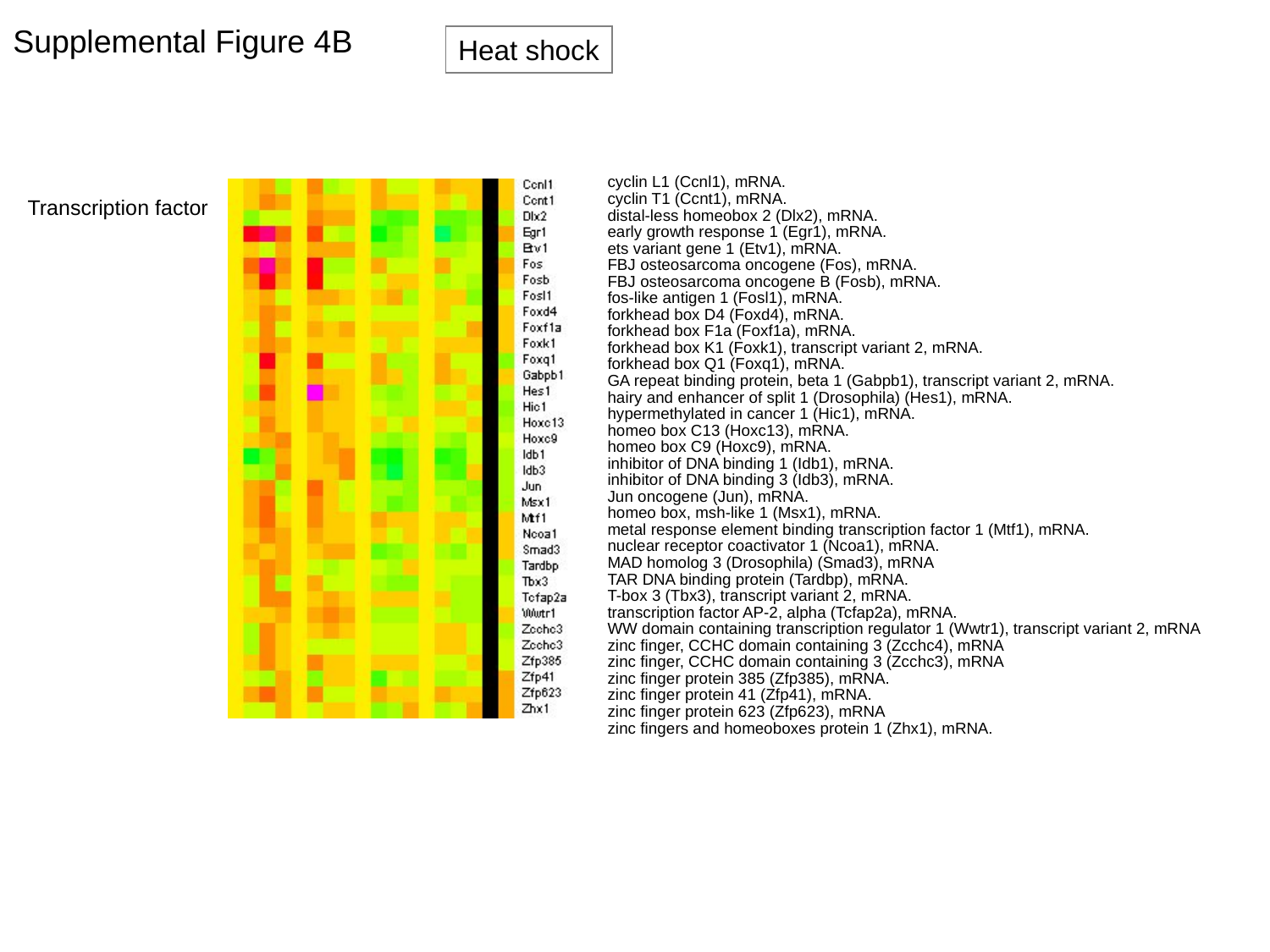

Supplemental Figure 4B
Heat shock
cyclin L1 (Ccnl1), mRNA.
cyclin T1 (Ccnt1), mRNA.
distal-less homeobox 2 (Dlx2), mRNA.
early growth response 1 (Egr1), mRNA.
ets variant gene 1 (Etv1), mRNA.
FBJ osteosarcoma oncogene (Fos), mRNA.
FBJ osteosarcoma oncogene B (Fosb), mRNA.
fos-like antigen 1 (Fosl1), mRNA.
forkhead box D4 (Foxd4), mRNA.
forkhead box F1a (Foxf1a), mRNA.
forkhead box K1 (Foxk1), transcript variant 2, mRNA.
forkhead box Q1 (Foxq1), mRNA.
GA repeat binding protein, beta 1 (Gabpb1), transcript variant 2, mRNA.
hairy and enhancer of split 1 (Drosophila) (Hes1), mRNA.
hypermethylated in cancer 1 (Hic1), mRNA.
homeo box C13 (Hoxc13), mRNA.
homeo box C9 (Hoxc9), mRNA.
inhibitor of DNA binding 1 (Idb1), mRNA.
inhibitor of DNA binding 3 (Idb3), mRNA.
Jun oncogene (Jun), mRNA.
homeo box, msh-like 1 (Msx1), mRNA.
metal response element binding transcription factor 1 (Mtf1), mRNA.
nuclear receptor coactivator 1 (Ncoa1), mRNA.
MAD homolog 3 (Drosophila) (Smad3), mRNA
TAR DNA binding protein (Tardbp), mRNA.
T-box 3 (Tbx3), transcript variant 2, mRNA.
transcription factor AP-2, alpha (Tcfap2a), mRNA.
WW domain containing transcription regulator 1 (Wwtr1), transcript variant 2, mRNA
zinc finger, CCHC domain containing 3 (Zcchc4), mRNA
zinc finger, CCHC domain containing 3 (Zcchc3), mRNA
zinc finger protein 385 (Zfp385), mRNA.
zinc finger protein 41 (Zfp41), mRNA.
zinc finger protein 623 (Zfp623), mRNA
zinc fingers and homeoboxes protein 1 (Zhx1), mRNA.
Transcription factor

## Slide 6
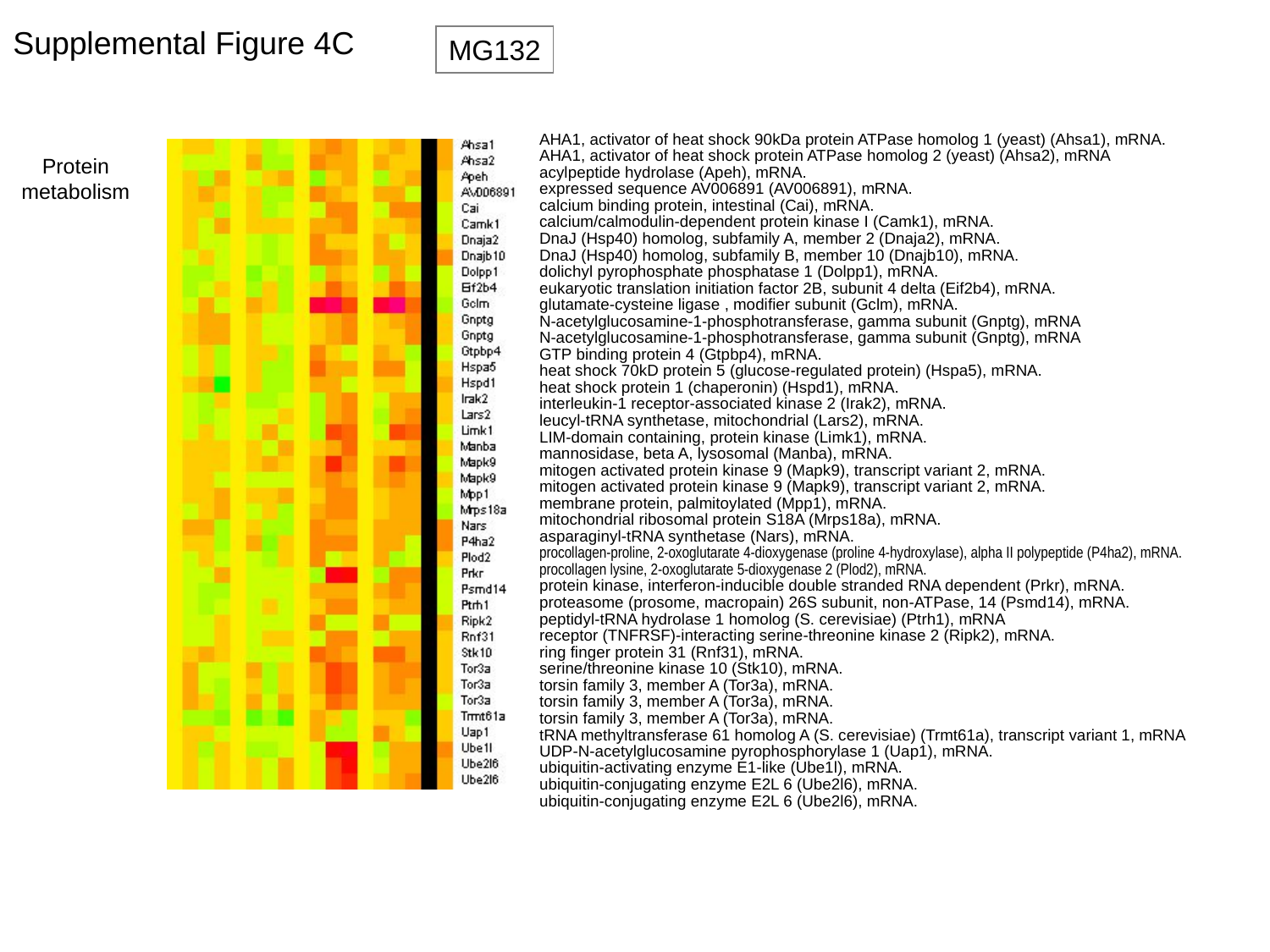

Supplemental Figure 4C
MG132
AHA1, activator of heat shock 90kDa protein ATPase homolog 1 (yeast) (Ahsa1), mRNA.
AHA1, activator of heat shock protein ATPase homolog 2 (yeast) (Ahsa2), mRNA
acylpeptide hydrolase (Apeh), mRNA.
expressed sequence AV006891 (AV006891), mRNA.
calcium binding protein, intestinal (Cai), mRNA.
calcium/calmodulin-dependent protein kinase I (Camk1), mRNA.
DnaJ (Hsp40) homolog, subfamily A, member 2 (Dnaja2), mRNA.
DnaJ (Hsp40) homolog, subfamily B, member 10 (Dnajb10), mRNA.
dolichyl pyrophosphate phosphatase 1 (Dolpp1), mRNA.
eukaryotic translation initiation factor 2B, subunit 4 delta (Eif2b4), mRNA.
glutamate-cysteine ligase , modifier subunit (Gclm), mRNA.
N-acetylglucosamine-1-phosphotransferase, gamma subunit (Gnptg), mRNA
N-acetylglucosamine-1-phosphotransferase, gamma subunit (Gnptg), mRNA
GTP binding protein 4 (Gtpbp4), mRNA.
heat shock 70kD protein 5 (glucose-regulated protein) (Hspa5), mRNA.
heat shock protein 1 (chaperonin) (Hspd1), mRNA.
interleukin-1 receptor-associated kinase 2 (Irak2), mRNA.
leucyl-tRNA synthetase, mitochondrial (Lars2), mRNA.
LIM-domain containing, protein kinase (Limk1), mRNA.
mannosidase, beta A, lysosomal (Manba), mRNA.
mitogen activated protein kinase 9 (Mapk9), transcript variant 2, mRNA.
mitogen activated protein kinase 9 (Mapk9), transcript variant 2, mRNA.
membrane protein, palmitoylated (Mpp1), mRNA.
mitochondrial ribosomal protein S18A (Mrps18a), mRNA.
asparaginyl-tRNA synthetase (Nars), mRNA.
procollagen-proline, 2-oxoglutarate 4-dioxygenase (proline 4-hydroxylase), alpha II polypeptide (P4ha2), mRNA.
procollagen lysine, 2-oxoglutarate 5-dioxygenase 2 (Plod2), mRNA.
protein kinase, interferon-inducible double stranded RNA dependent (Prkr), mRNA.
proteasome (prosome, macropain) 26S subunit, non-ATPase, 14 (Psmd14), mRNA.
peptidyl-tRNA hydrolase 1 homolog (S. cerevisiae) (Ptrh1), mRNA
receptor (TNFRSF)-interacting serine-threonine kinase 2 (Ripk2), mRNA.
ring finger protein 31 (Rnf31), mRNA.
serine/threonine kinase 10 (Stk10), mRNA.
torsin family 3, member A (Tor3a), mRNA.
torsin family 3, member A (Tor3a), mRNA.
torsin family 3, member A (Tor3a), mRNA.
tRNA methyltransferase 61 homolog A (S. cerevisiae) (Trmt61a), transcript variant 1, mRNA
UDP-N-acetylglucosamine pyrophosphorylase 1 (Uap1), mRNA.
ubiquitin-activating enzyme E1-like (Ube1l), mRNA.
ubiquitin-conjugating enzyme E2L 6 (Ube2l6), mRNA.
ubiquitin-conjugating enzyme E2L 6 (Ube2l6), mRNA.
Protein
metabolism

## Slide 7
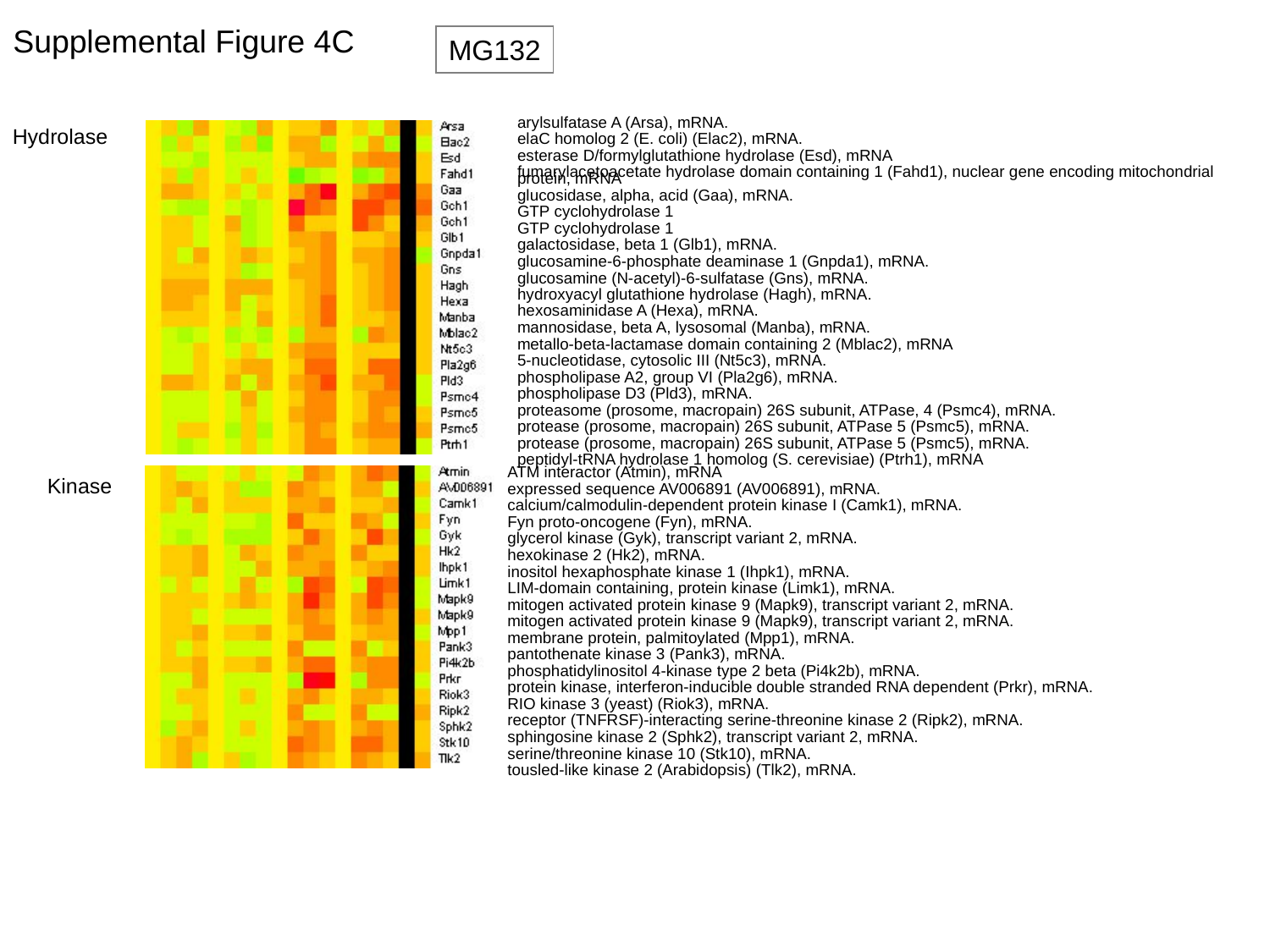

Supplemental Figure 4C
MG132
Hydrolase
arylsulfatase A (Arsa), mRNA.
elaC homolog 2 (E. coli) (Elac2), mRNA.
esterase D/formylglutathione hydrolase (Esd), mRNA
fumarylacetoacetate hydrolase domain containing 1 (Fahd1), nuclear gene encoding mitochondrial protein, mRNA
glucosidase, alpha, acid (Gaa), mRNA.
GTP cyclohydrolase 1
GTP cyclohydrolase 1
galactosidase, beta 1 (Glb1), mRNA.
glucosamine-6-phosphate deaminase 1 (Gnpda1), mRNA.
glucosamine (N-acetyl)-6-sulfatase (Gns), mRNA.
hydroxyacyl glutathione hydrolase (Hagh), mRNA.
hexosaminidase A (Hexa), mRNA.
mannosidase, beta A, lysosomal (Manba), mRNA.
metallo-beta-lactamase domain containing 2 (Mblac2), mRNA
5-nucleotidase, cytosolic III (Nt5c3), mRNA.
phospholipase A2, group VI (Pla2g6), mRNA.
phospholipase D3 (Pld3), mRNA.
proteasome (prosome, macropain) 26S subunit, ATPase, 4 (Psmc4), mRNA.
protease (prosome, macropain) 26S subunit, ATPase 5 (Psmc5), mRNA.
protease (prosome, macropain) 26S subunit, ATPase 5 (Psmc5), mRNA.
peptidyl-tRNA hydrolase 1 homolog (S. cerevisiae) (Ptrh1), mRNA
Kinase
ATM interactor (Atmin), mRNA
expressed sequence AV006891 (AV006891), mRNA.
calcium/calmodulin-dependent protein kinase I (Camk1), mRNA.
Fyn proto-oncogene (Fyn), mRNA.
glycerol kinase (Gyk), transcript variant 2, mRNA.
hexokinase 2 (Hk2), mRNA.
inositol hexaphosphate kinase 1 (Ihpk1), mRNA.
LIM-domain containing, protein kinase (Limk1), mRNA.
mitogen activated protein kinase 9 (Mapk9), transcript variant 2, mRNA.
mitogen activated protein kinase 9 (Mapk9), transcript variant 2, mRNA.
membrane protein, palmitoylated (Mpp1), mRNA.
pantothenate kinase 3 (Pank3), mRNA.
phosphatidylinositol 4-kinase type 2 beta (Pi4k2b), mRNA.
protein kinase, interferon-inducible double stranded RNA dependent (Prkr), mRNA.
RIO kinase 3 (yeast) (Riok3), mRNA.
receptor (TNFRSF)-interacting serine-threonine kinase 2 (Ripk2), mRNA.
sphingosine kinase 2 (Sphk2), transcript variant 2, mRNA.
serine/threonine kinase 10 (Stk10), mRNA.
tousled-like kinase 2 (Arabidopsis) (Tlk2), mRNA.

## Slide 8
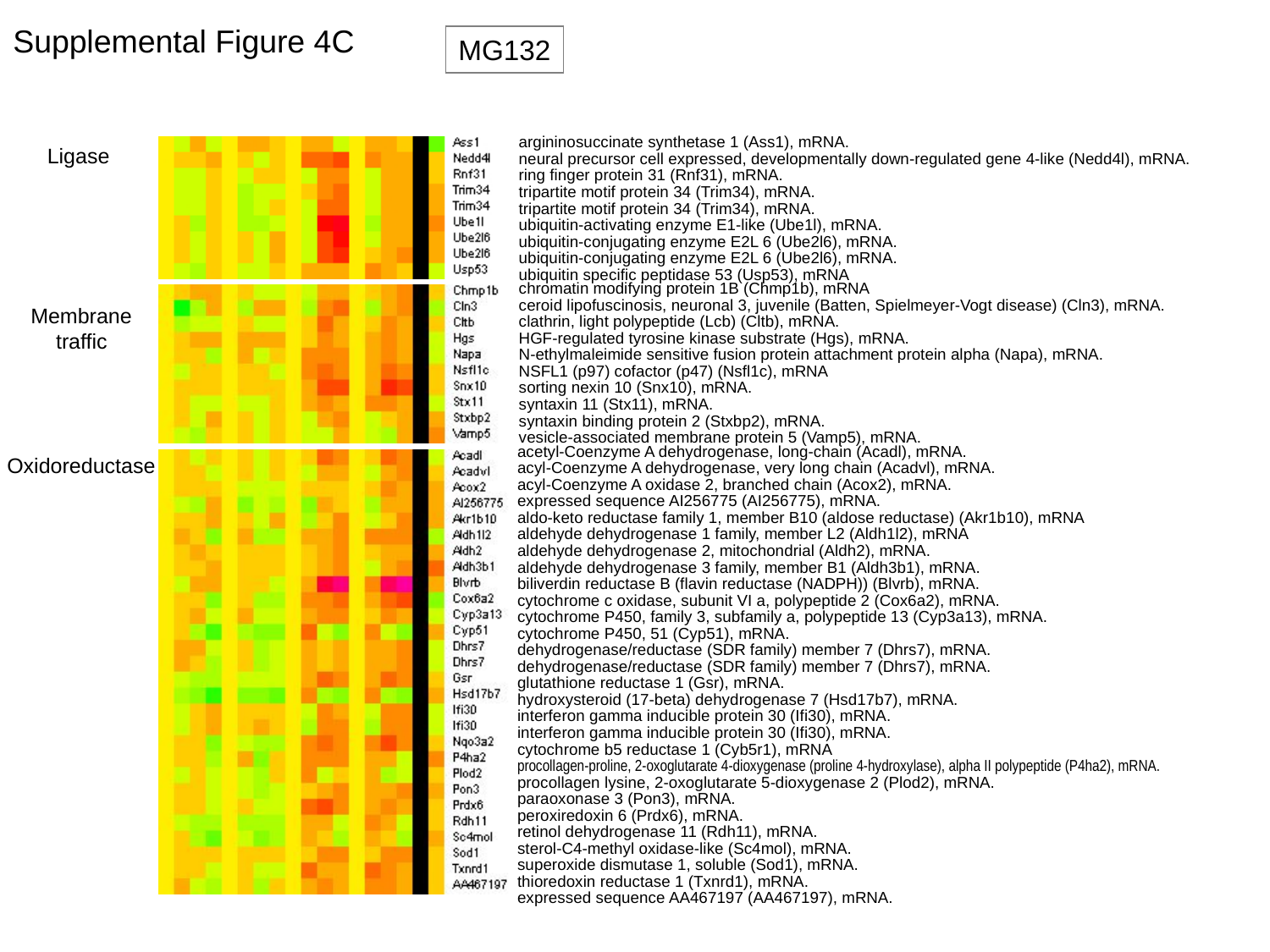

Supplemental Figure 4C
MG132
Ligase
argininosuccinate synthetase 1 (Ass1), mRNA.
neural precursor cell expressed, developmentally down-regulated gene 4-like (Nedd4l), mRNA.
ring finger protein 31 (Rnf31), mRNA.
tripartite motif protein 34 (Trim34), mRNA.
tripartite motif protein 34 (Trim34), mRNA.
ubiquitin-activating enzyme E1-like (Ube1l), mRNA.
ubiquitin-conjugating enzyme E2L 6 (Ube2l6), mRNA.
ubiquitin-conjugating enzyme E2L 6 (Ube2l6), mRNA.
ubiquitin specific peptidase 53 (Usp53), mRNA
chromatin modifying protein 1B (Chmp1b), mRNA
ceroid lipofuscinosis, neuronal 3, juvenile (Batten, Spielmeyer-Vogt disease) (Cln3), mRNA.
clathrin, light polypeptide (Lcb) (Cltb), mRNA.
HGF-regulated tyrosine kinase substrate (Hgs), mRNA.
N-ethylmaleimide sensitive fusion protein attachment protein alpha (Napa), mRNA.
NSFL1 (p97) cofactor (p47) (Nsfl1c), mRNA
sorting nexin 10 (Snx10), mRNA.
syntaxin 11 (Stx11), mRNA.
syntaxin binding protein 2 (Stxbp2), mRNA.
vesicle-associated membrane protein 5 (Vamp5), mRNA.
Membrane
traffic
acetyl-Coenzyme A dehydrogenase, long-chain (Acadl), mRNA.
acyl-Coenzyme A dehydrogenase, very long chain (Acadvl), mRNA.
acyl-Coenzyme A oxidase 2, branched chain (Acox2), mRNA.
expressed sequence AI256775 (AI256775), mRNA.
aldo-keto reductase family 1, member B10 (aldose reductase) (Akr1b10), mRNA
aldehyde dehydrogenase 1 family, member L2 (Aldh1l2), mRNA
aldehyde dehydrogenase 2, mitochondrial (Aldh2), mRNA.
aldehyde dehydrogenase 3 family, member B1 (Aldh3b1), mRNA.
biliverdin reductase B (flavin reductase (NADPH)) (Blvrb), mRNA.
cytochrome c oxidase, subunit VI a, polypeptide 2 (Cox6a2), mRNA.
cytochrome P450, family 3, subfamily a, polypeptide 13 (Cyp3a13), mRNA.
cytochrome P450, 51 (Cyp51), mRNA.
dehydrogenase/reductase (SDR family) member 7 (Dhrs7), mRNA.
dehydrogenase/reductase (SDR family) member 7 (Dhrs7), mRNA.
glutathione reductase 1 (Gsr), mRNA.
hydroxysteroid (17-beta) dehydrogenase 7 (Hsd17b7), mRNA.
interferon gamma inducible protein 30 (Ifi30), mRNA.
interferon gamma inducible protein 30 (Ifi30), mRNA.
cytochrome b5 reductase 1 (Cyb5r1), mRNA
procollagen-proline, 2-oxoglutarate 4-dioxygenase (proline 4-hydroxylase), alpha II polypeptide (P4ha2), mRNA.
procollagen lysine, 2-oxoglutarate 5-dioxygenase 2 (Plod2), mRNA.
paraoxonase 3 (Pon3), mRNA.
peroxiredoxin 6 (Prdx6), mRNA.
retinol dehydrogenase 11 (Rdh11), mRNA.
sterol-C4-methyl oxidase-like (Sc4mol), mRNA.
superoxide dismutase 1, soluble (Sod1), mRNA.
thioredoxin reductase 1 (Txnrd1), mRNA.
expressed sequence AA467197 (AA467197), mRNA.
Oxidoreductase

## Slide 9
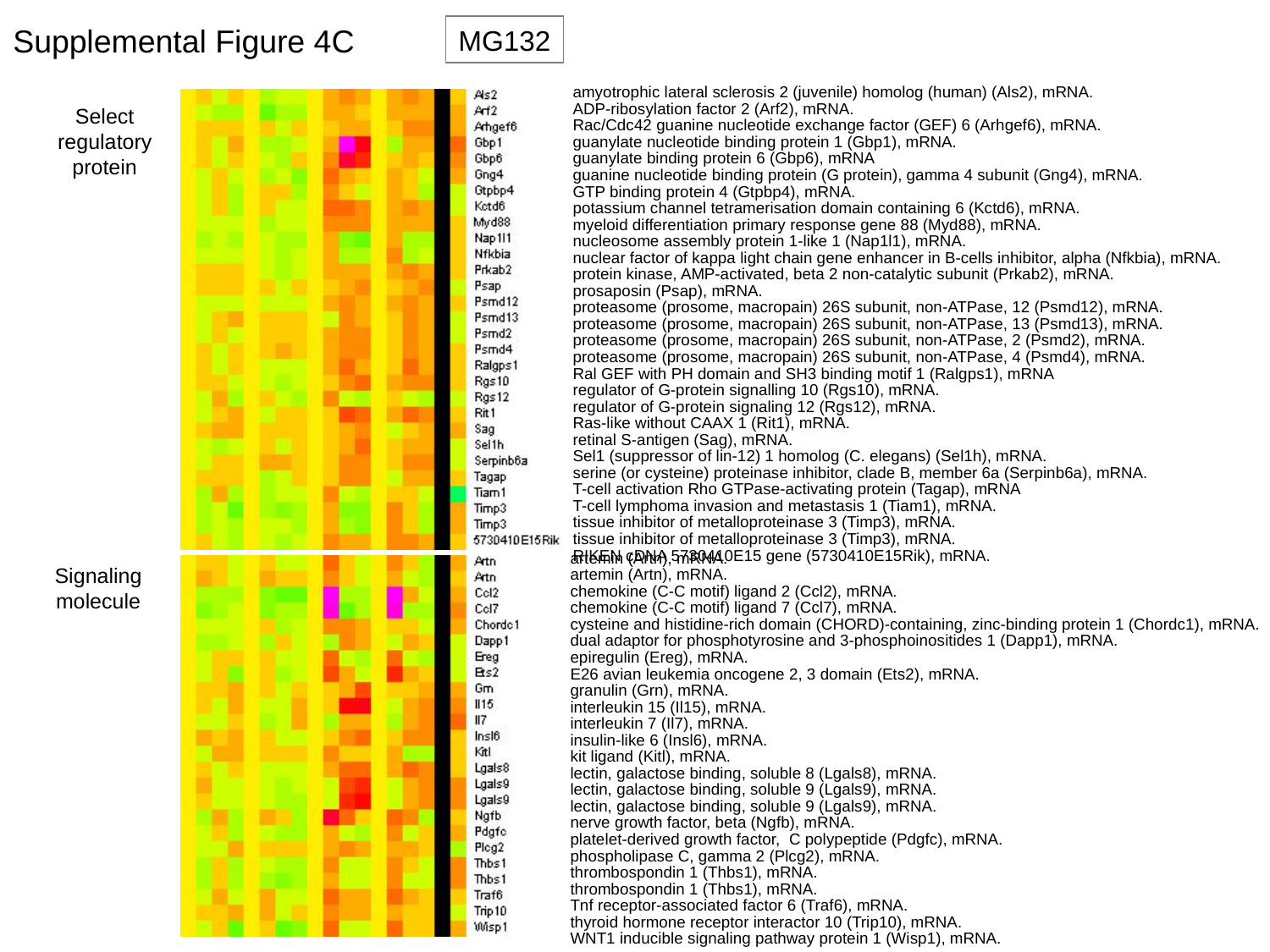

Supplemental Figure 4C
MG132
amyotrophic lateral sclerosis 2 (juvenile) homolog (human) (Als2), mRNA.
ADP-ribosylation factor 2 (Arf2), mRNA.
Rac/Cdc42 guanine nucleotide exchange factor (GEF) 6 (Arhgef6), mRNA.
guanylate nucleotide binding protein 1 (Gbp1), mRNA.
guanylate binding protein 6 (Gbp6), mRNA
guanine nucleotide binding protein (G protein), gamma 4 subunit (Gng4), mRNA.
GTP binding protein 4 (Gtpbp4), mRNA.
potassium channel tetramerisation domain containing 6 (Kctd6), mRNA.
myeloid differentiation primary response gene 88 (Myd88), mRNA.
nucleosome assembly protein 1-like 1 (Nap1l1), mRNA.
nuclear factor of kappa light chain gene enhancer in B-cells inhibitor, alpha (Nfkbia), mRNA.
protein kinase, AMP-activated, beta 2 non-catalytic subunit (Prkab2), mRNA.
prosaposin (Psap), mRNA.
proteasome (prosome, macropain) 26S subunit, non-ATPase, 12 (Psmd12), mRNA.
proteasome (prosome, macropain) 26S subunit, non-ATPase, 13 (Psmd13), mRNA.
proteasome (prosome, macropain) 26S subunit, non-ATPase, 2 (Psmd2), mRNA.
proteasome (prosome, macropain) 26S subunit, non-ATPase, 4 (Psmd4), mRNA.
Ral GEF with PH domain and SH3 binding motif 1 (Ralgps1), mRNA
regulator of G-protein signalling 10 (Rgs10), mRNA.
regulator of G-protein signaling 12 (Rgs12), mRNA.
Ras-like without CAAX 1 (Rit1), mRNA.
retinal S-antigen (Sag), mRNA.
Sel1 (suppressor of lin-12) 1 homolog (C. elegans) (Sel1h), mRNA.
serine (or cysteine) proteinase inhibitor, clade B, member 6a (Serpinb6a), mRNA.
T-cell activation Rho GTPase-activating protein (Tagap), mRNA
T-cell lymphoma invasion and metastasis 1 (Tiam1), mRNA.
tissue inhibitor of metalloproteinase 3 (Timp3), mRNA.
tissue inhibitor of metalloproteinase 3 (Timp3), mRNA.
RIKEN cDNA 5730410E15 gene (5730410E15Rik), mRNA.
Select
regulatory
protein
artemin (Artn), mRNA.
artemin (Artn), mRNA.
chemokine (C-C motif) ligand 2 (Ccl2), mRNA.
chemokine (C-C motif) ligand 7 (Ccl7), mRNA.
cysteine and histidine-rich domain (CHORD)-containing, zinc-binding protein 1 (Chordc1), mRNA.
dual adaptor for phosphotyrosine and 3-phosphoinositides 1 (Dapp1), mRNA.
epiregulin (Ereg), mRNA.
E26 avian leukemia oncogene 2, 3 domain (Ets2), mRNA.
granulin (Grn), mRNA.
interleukin 15 (Il15), mRNA.
interleukin 7 (Il7), mRNA.
insulin-like 6 (Insl6), mRNA.
kit ligand (Kitl), mRNA.
lectin, galactose binding, soluble 8 (Lgals8), mRNA.
lectin, galactose binding, soluble 9 (Lgals9), mRNA.
lectin, galactose binding, soluble 9 (Lgals9), mRNA.
nerve growth factor, beta (Ngfb), mRNA.
platelet-derived growth factor, C polypeptide (Pdgfc), mRNA.
phospholipase C, gamma 2 (Plcg2), mRNA.
thrombospondin 1 (Thbs1), mRNA.
thrombospondin 1 (Thbs1), mRNA.
Tnf receptor-associated factor 6 (Traf6), mRNA.
thyroid hormone receptor interactor 10 (Trip10), mRNA.
WNT1 inducible signaling pathway protein 1 (Wisp1), mRNA.
Signaling
molecule

## Slide 10
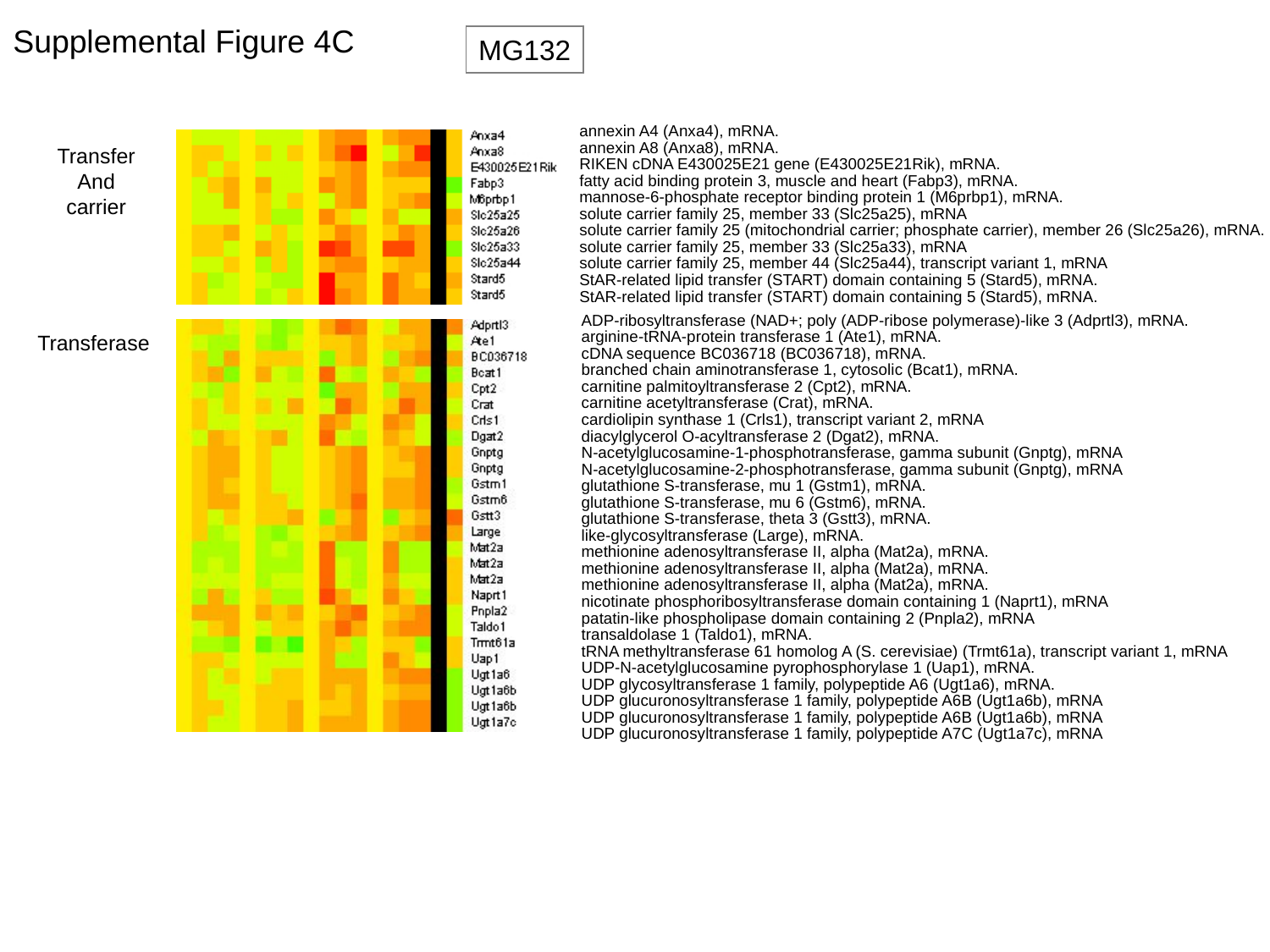

Supplemental Figure 4C
MG132
annexin A4 (Anxa4), mRNA.
annexin A8 (Anxa8), mRNA.
RIKEN cDNA E430025E21 gene (E430025E21Rik), mRNA.
fatty acid binding protein 3, muscle and heart (Fabp3), mRNA.
mannose-6-phosphate receptor binding protein 1 (M6prbp1), mRNA.
solute carrier family 25, member 33 (Slc25a25), mRNA
solute carrier family 25 (mitochondrial carrier; phosphate carrier), member 26 (Slc25a26), mRNA.
solute carrier family 25, member 33 (Slc25a33), mRNA
solute carrier family 25, member 44 (Slc25a44), transcript variant 1, mRNA
StAR-related lipid transfer (START) domain containing 5 (Stard5), mRNA.
StAR-related lipid transfer (START) domain containing 5 (Stard5), mRNA.
Transfer
And
carrier
ADP-ribosyltransferase (NAD+; poly (ADP-ribose polymerase)-like 3 (Adprtl3), mRNA.
arginine-tRNA-protein transferase 1 (Ate1), mRNA.
cDNA sequence BC036718 (BC036718), mRNA.
branched chain aminotransferase 1, cytosolic (Bcat1), mRNA.
carnitine palmitoyltransferase 2 (Cpt2), mRNA.
carnitine acetyltransferase (Crat), mRNA.
cardiolipin synthase 1 (Crls1), transcript variant 2, mRNA
diacylglycerol O-acyltransferase 2 (Dgat2), mRNA.
N-acetylglucosamine-1-phosphotransferase, gamma subunit (Gnptg), mRNA
N-acetylglucosamine-2-phosphotransferase, gamma subunit (Gnptg), mRNA
glutathione S-transferase, mu 1 (Gstm1), mRNA.
glutathione S-transferase, mu 6 (Gstm6), mRNA.
glutathione S-transferase, theta 3 (Gstt3), mRNA.
like-glycosyltransferase (Large), mRNA.
methionine adenosyltransferase II, alpha (Mat2a), mRNA.
methionine adenosyltransferase II, alpha (Mat2a), mRNA.
methionine adenosyltransferase II, alpha (Mat2a), mRNA.
nicotinate phosphoribosyltransferase domain containing 1 (Naprt1), mRNA
patatin-like phospholipase domain containing 2 (Pnpla2), mRNA
transaldolase 1 (Taldo1), mRNA.
tRNA methyltransferase 61 homolog A (S. cerevisiae) (Trmt61a), transcript variant 1, mRNA
UDP-N-acetylglucosamine pyrophosphorylase 1 (Uap1), mRNA.
UDP glycosyltransferase 1 family, polypeptide A6 (Ugt1a6), mRNA.
UDP glucuronosyltransferase 1 family, polypeptide A6B (Ugt1a6b), mRNA
UDP glucuronosyltransferase 1 family, polypeptide A6B (Ugt1a6b), mRNA
UDP glucuronosyltransferase 1 family, polypeptide A7C (Ugt1a7c), mRNA
Transferase

## Slide 11
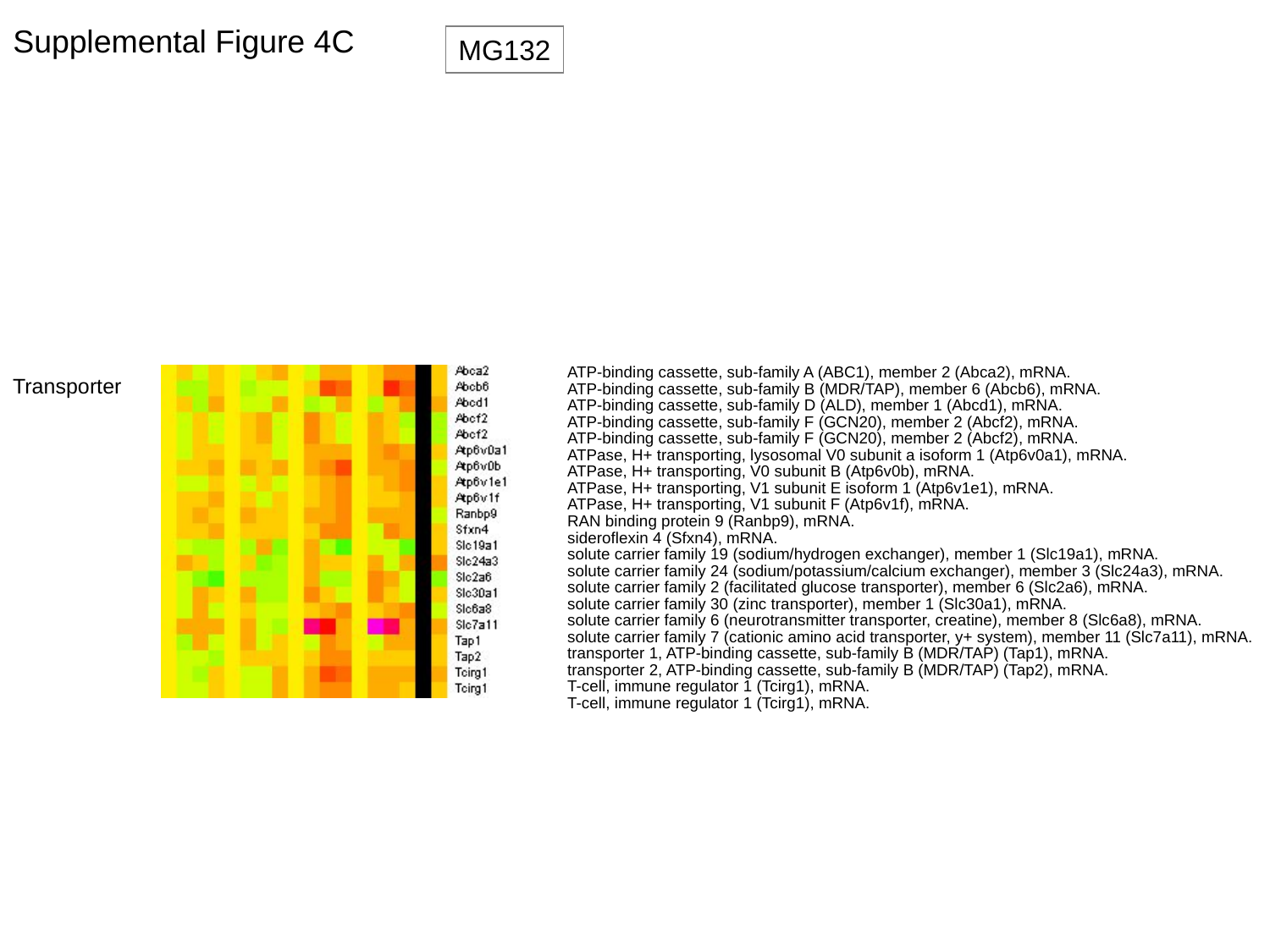

Supplemental Figure 4C
MG132
Transporter
ATP-binding cassette, sub-family A (ABC1), member 2 (Abca2), mRNA.
ATP-binding cassette, sub-family B (MDR/TAP), member 6 (Abcb6), mRNA.
ATP-binding cassette, sub-family D (ALD), member 1 (Abcd1), mRNA.
ATP-binding cassette, sub-family F (GCN20), member 2 (Abcf2), mRNA.
ATP-binding cassette, sub-family F (GCN20), member 2 (Abcf2), mRNA.
ATPase, H+ transporting, lysosomal V0 subunit a isoform 1 (Atp6v0a1), mRNA.
ATPase, H+ transporting, V0 subunit B (Atp6v0b), mRNA.
ATPase, H+ transporting, V1 subunit E isoform 1 (Atp6v1e1), mRNA.
ATPase, H+ transporting, V1 subunit F (Atp6v1f), mRNA.
RAN binding protein 9 (Ranbp9), mRNA.
sideroflexin 4 (Sfxn4), mRNA.
solute carrier family 19 (sodium/hydrogen exchanger), member 1 (Slc19a1), mRNA.
solute carrier family 24 (sodium/potassium/calcium exchanger), member 3 (Slc24a3), mRNA.
solute carrier family 2 (facilitated glucose transporter), member 6 (Slc2a6), mRNA.
solute carrier family 30 (zinc transporter), member 1 (Slc30a1), mRNA.
solute carrier family 6 (neurotransmitter transporter, creatine), member 8 (Slc6a8), mRNA.
solute carrier family 7 (cationic amino acid transporter, y+ system), member 11 (Slc7a11), mRNA.
transporter 1, ATP-binding cassette, sub-family B (MDR/TAP) (Tap1), mRNA.
transporter 2, ATP-binding cassette, sub-family B (MDR/TAP) (Tap2), mRNA.
T-cell, immune regulator 1 (Tcirg1), mRNA.
T-cell, immune regulator 1 (Tcirg1), mRNA.
