# Supplementary material for: Systemic Analysis of Heat Shock Response Induced by Heat Shock and a Proteasome Inhibitor MG132
Source: PLoS One. 2011 Jun 30;6(6):e20252. doi: 10.1371/journal.pone.0020252 (PMC3127947; doi:10.1371/journal.pone.0020252)
Supplement: Table S1 — Genes up-regulated more than 3-fold in TR cells compared to RIF-1 cells. (PPT) [file pone.0020252.s008.ppt]

## Slide 1
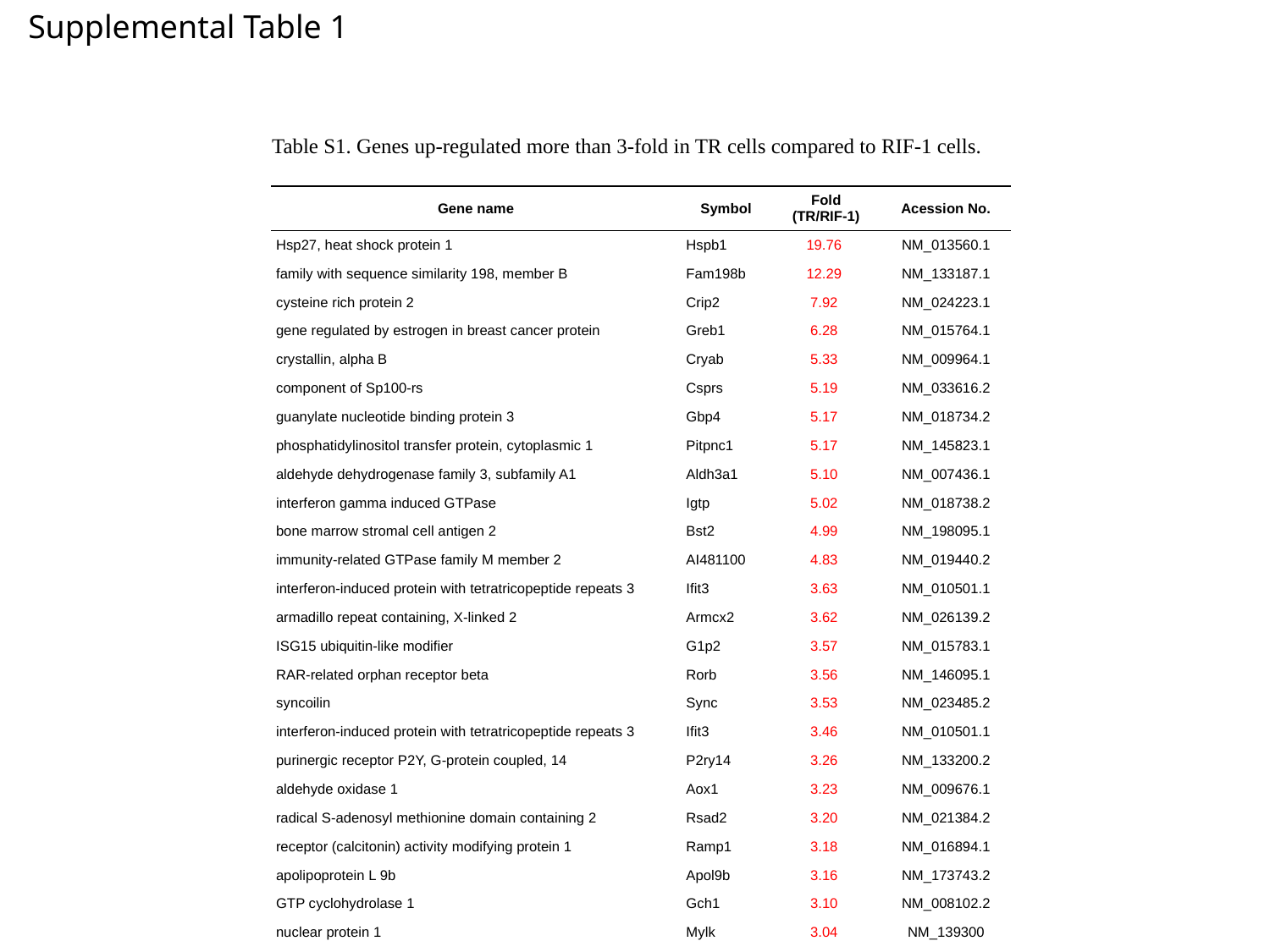

Supplemental Table 1
Table S1. Genes up-regulated more than 3-fold in TR cells compared to RIF-1 cells.
| Gene name | Symbol | Fold (TR/RIF-1) | Acession No. |
| --- | --- | --- | --- |
| Hsp27, heat shock protein 1 | Hspb1 | 19.76 | NM\_013560.1 |
| family with sequence similarity 198, member B | Fam198b | 12.29 | NM\_133187.1 |
| cysteine rich protein 2 | Crip2 | 7.92 | NM\_024223.1 |
| gene regulated by estrogen in breast cancer protein | Greb1 | 6.28 | NM\_015764.1 |
| crystallin, alpha B | Cryab | 5.33 | NM\_009964.1 |
| component of Sp100-rs | Csprs | 5.19 | NM\_033616.2 |
| guanylate nucleotide binding protein 3 | Gbp4 | 5.17 | NM\_018734.2 |
| phosphatidylinositol transfer protein, cytoplasmic 1 | Pitpnc1 | 5.17 | NM\_145823.1 |
| aldehyde dehydrogenase family 3, subfamily A1 | Aldh3a1 | 5.10 | NM\_007436.1 |
| interferon gamma induced GTPase | Igtp | 5.02 | NM\_018738.2 |
| bone marrow stromal cell antigen 2 | Bst2 | 4.99 | NM\_198095.1 |
| immunity-related GTPase family M member 2 | AI481100 | 4.83 | NM\_019440.2 |
| interferon-induced protein with tetratricopeptide repeats 3 | Ifit3 | 3.63 | NM\_010501.1 |
| armadillo repeat containing, X-linked 2 | Armcx2 | 3.62 | NM\_026139.2 |
| ISG15 ubiquitin-like modifier | G1p2 | 3.57 | NM\_015783.1 |
| RAR-related orphan receptor beta | Rorb | 3.56 | NM\_146095.1 |
| syncoilin | Sync | 3.53 | NM\_023485.2 |
| interferon-induced protein with tetratricopeptide repeats 3 | Ifit3 | 3.46 | NM\_010501.1 |
| purinergic receptor P2Y, G-protein coupled, 14 | P2ry14 | 3.26 | NM\_133200.2 |
| aldehyde oxidase 1 | Aox1 | 3.23 | NM\_009676.1 |
| radical S-adenosyl methionine domain containing 2 | Rsad2 | 3.20 | NM\_021384.2 |
| receptor (calcitonin) activity modifying protein 1 | Ramp1 | 3.18 | NM\_016894.1 |
| apolipoprotein L 9b | Apol9b | 3.16 | NM\_173743.2 |
| GTP cyclohydrolase 1 | Gch1 | 3.10 | NM\_008102.2 |
| nuclear protein 1 | Mylk | 3.04 | NM\_139300 |
| meiosis-specific nuclear structural protein 1 | Nupr1 | 3.02 | NM\_019738.1 |
| hypothetical protein LOC223672 | Mns1 | 3.00 | NM\_008613.1 |
