# Supplementary material for: Systemic Analysis of Heat Shock Response Induced by Heat Shock and a Proteasome Inhibitor MG132
Source: PLoS One. 2011 Jun 30;6(6):e20252. doi: 10.1371/journal.pone.0020252 (PMC3127947; doi:10.1371/journal.pone.0020252)
Supplement: Table S2 — Genes down-regulated more than 3-fold in TR cells compared to RIF-1 cells. (PPT) [file pone.0020252.s009.ppt]

## Slide 1
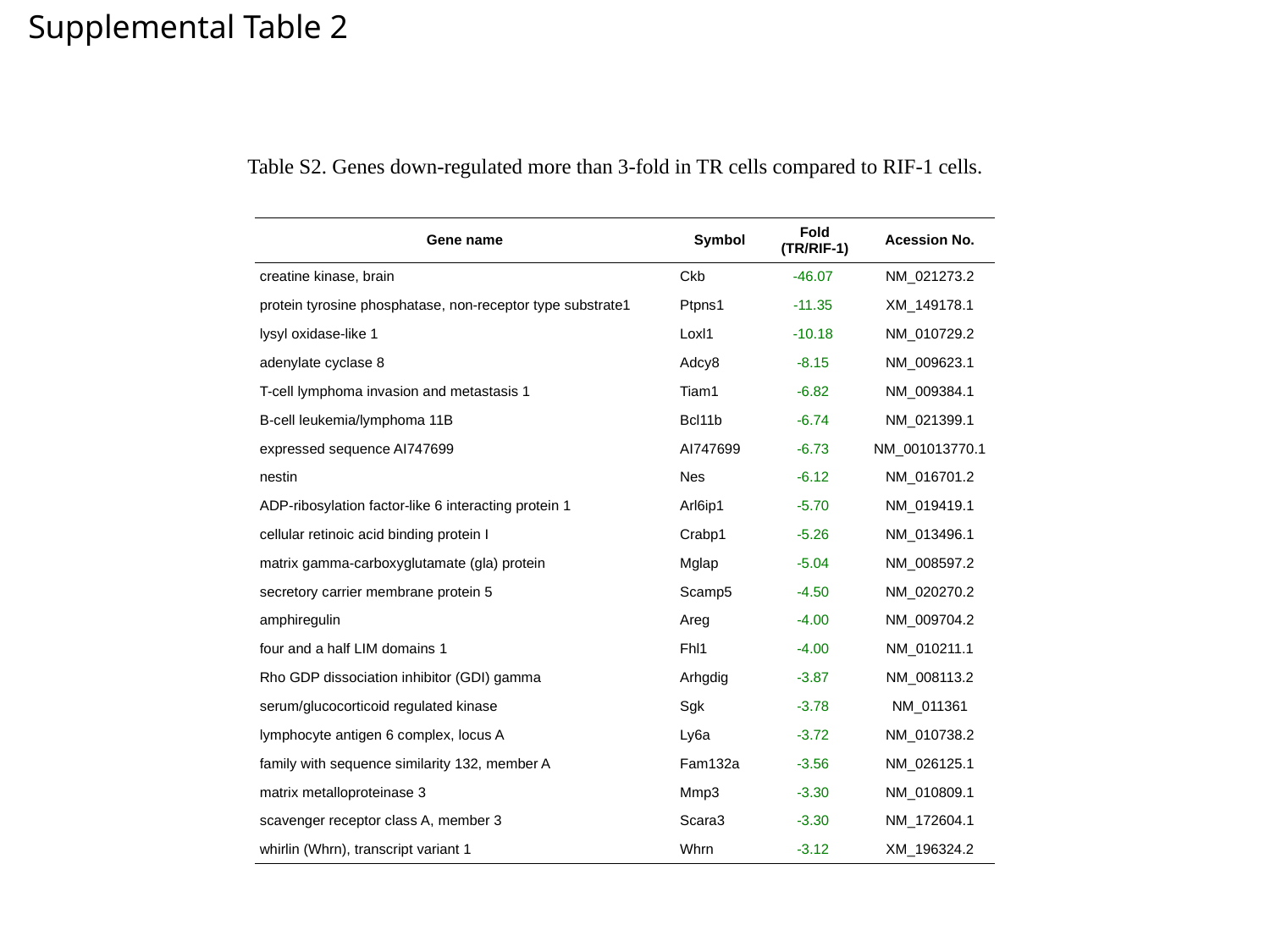

Supplemental Table 2
Table S2. Genes down-regulated more than 3-fold in TR cells compared to RIF-1 cells.
| Gene name | Symbol | Fold (TR/RIF-1) | Acession No. |
| --- | --- | --- | --- |
| creatine kinase, brain | Ckb | -46.07 | NM\_021273.2 |
| protein tyrosine phosphatase, non-receptor type substrate1 | Ptpns1 | -11.35 | XM\_149178.1 |
| lysyl oxidase-like 1 | Loxl1 | -10.18 | NM\_010729.2 |
| adenylate cyclase 8 | Adcy8 | -8.15 | NM\_009623.1 |
| T-cell lymphoma invasion and metastasis 1 | Tiam1 | -6.82 | NM\_009384.1 |
| B-cell leukemia/lymphoma 11B | Bcl11b | -6.74 | NM\_021399.1 |
| expressed sequence AI747699 | AI747699 | -6.73 | NM\_001013770.1 |
| nestin | Nes | -6.12 | NM\_016701.2 |
| ADP-ribosylation factor-like 6 interacting protein 1 | Arl6ip1 | -5.70 | NM\_019419.1 |
| cellular retinoic acid binding protein I | Crabp1 | -5.26 | NM\_013496.1 |
| matrix gamma-carboxyglutamate (gla) protein | Mglap | -5.04 | NM\_008597.2 |
| secretory carrier membrane protein 5 | Scamp5 | -4.50 | NM\_020270.2 |
| amphiregulin | Areg | -4.00 | NM\_009704.2 |
| four and a half LIM domains 1 | Fhl1 | -4.00 | NM\_010211.1 |
| Rho GDP dissociation inhibitor (GDI) gamma | Arhgdig | -3.87 | NM\_008113.2 |
| serum/glucocorticoid regulated kinase | Sgk | -3.78 | NM\_011361 |
| lymphocyte antigen 6 complex, locus A | Ly6a | -3.72 | NM\_010738.2 |
| family with sequence similarity 132, member A | Fam132a | -3.56 | NM\_026125.1 |
| matrix metalloproteinase 3 | Mmp3 | -3.30 | NM\_010809.1 |
| scavenger receptor class A, member 3 | Scara3 | -3.30 | NM\_172604.1 |
| whirlin (Whrn), transcript variant 1 | Whrn | -3.12 | XM\_196324.2 |
