# Supplementary material for: Systemic Analysis of Heat Shock Response Induced by Heat Shock and a Proteasome Inhibitor MG132
Source: PLoS One. 2011 Jun 30;6(6):e20252. doi: 10.1371/journal.pone.0020252 (PMC3127947; doi:10.1371/journal.pone.0020252)
Supplement: Table S3 — Summary of the linear mixed effect model of growth index (heat shock: sample size of 3630 from 30 objects, MG132: sample size of 2430 from 30 objects) (PPT) [file pone.0020252.s010.ppt]

## Slide 1
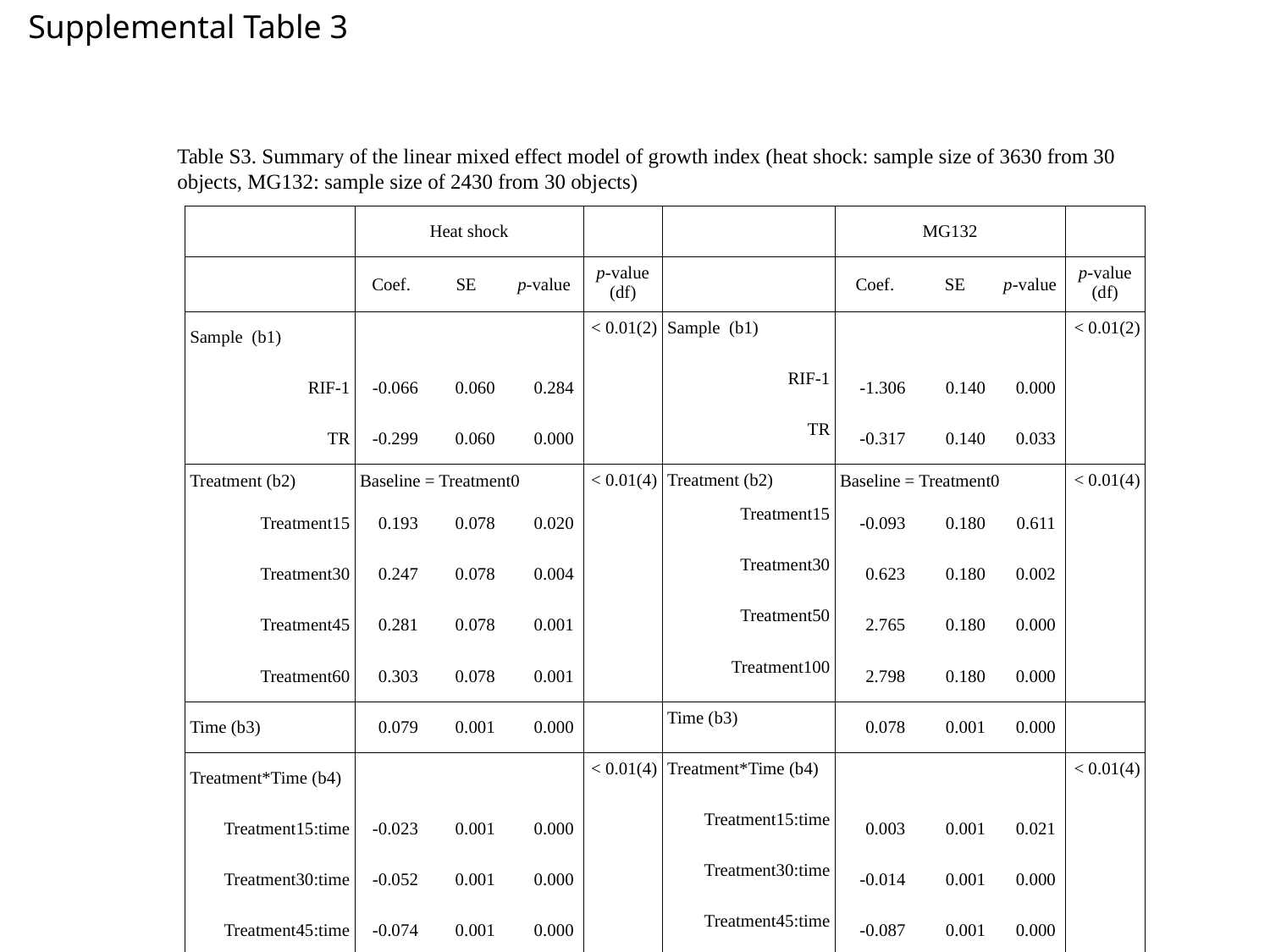

Supplemental Table 3
Table S3. Summary of the linear mixed effect model of growth index (heat shock: sample size of 3630 from 30 objects, MG132: sample size of 2430 from 30 objects)
| | Heat shock | | | | | MG132 | | | |
| --- | --- | --- | --- | --- | --- | --- | --- | --- | --- |
| | Coef. | SE | p-value | p-value (df) | | Coef. | SE | p-value | p-value (df) |
| Sample (b1) | | | | < 0.01(2) | Sample (b1) | | | | < 0.01(2) |
| RIF-1 | -0.066 | 0.060 | 0.284 | | RIF-1 | -1.306 | 0.140 | 0.000 | |
| TR | -0.299 | 0.060 | 0.000 | | TR | -0.317 | 0.140 | 0.033 | |
| Treatment (b2) | Baseline = Treatment0 | | | < 0.01(4) | Treatment (b2) | Baseline = Treatment0 | | | < 0.01(4) |
| Treatment15 | 0.193 | 0.078 | 0.020 | | Treatment15 | -0.093 | 0.180 | 0.611 | |
| Treatment30 | 0.247 | 0.078 | 0.004 | | Treatment30 | 0.623 | 0.180 | 0.002 | |
| Treatment45 | 0.281 | 0.078 | 0.001 | | Treatment50 | 2.765 | 0.180 | 0.000 | |
| Treatment60 | 0.303 | 0.078 | 0.001 | | Treatment100 | 2.798 | 0.180 | 0.000 | |
| Time (b3) | 0.079 | 0.001 | 0.000 | | Time (b3) | 0.078 | 0.001 | 0.000 | |
| Treatment\*Time (b4) | | | | < 0.01(4) | Treatment\*Time (b4) | | | | < 0.01(4) |
| Treatment15:time | -0.023 | 0.001 | 0.000 | | Treatment15:time | 0.003 | 0.001 | 0.021 | |
| Treatment30:time | -0.052 | 0.001 | 0.000 | | Treatment30:time | -0.014 | 0.001 | 0.000 | |
| Treatment45:time | -0.074 | 0.001 | 0.000 | | Treatment45:time | -0.087 | 0.001 | 0.000 | |
| Treatment60:time | -0.080 | 0.001 | 0.000 | | Treatment60:time | -0.100 | 0.001 | 0.000 | |
| Sample\*Time (b5) | 0.017 | 0.001 | 0.000 | | Sample\*Time (b5) | -0.015 | 0.001 | 0.000 | |
