# Supplementary material for: Systemic Analysis of Heat Shock Response Induced by Heat Shock and a Proteasome Inhibitor MG132
Source: PLoS One. 2011 Jun 30;6(6):e20252. doi: 10.1371/journal.pone.0020252 (PMC3127947; doi:10.1371/journal.pone.0020252)
Supplement: Table S4 — Over-represented gene ontology categories of the down-regulated genes in heat shock treated RIF-1 cells. (PPT) [file pone.0020252.s011.ppt]

## Slide 1
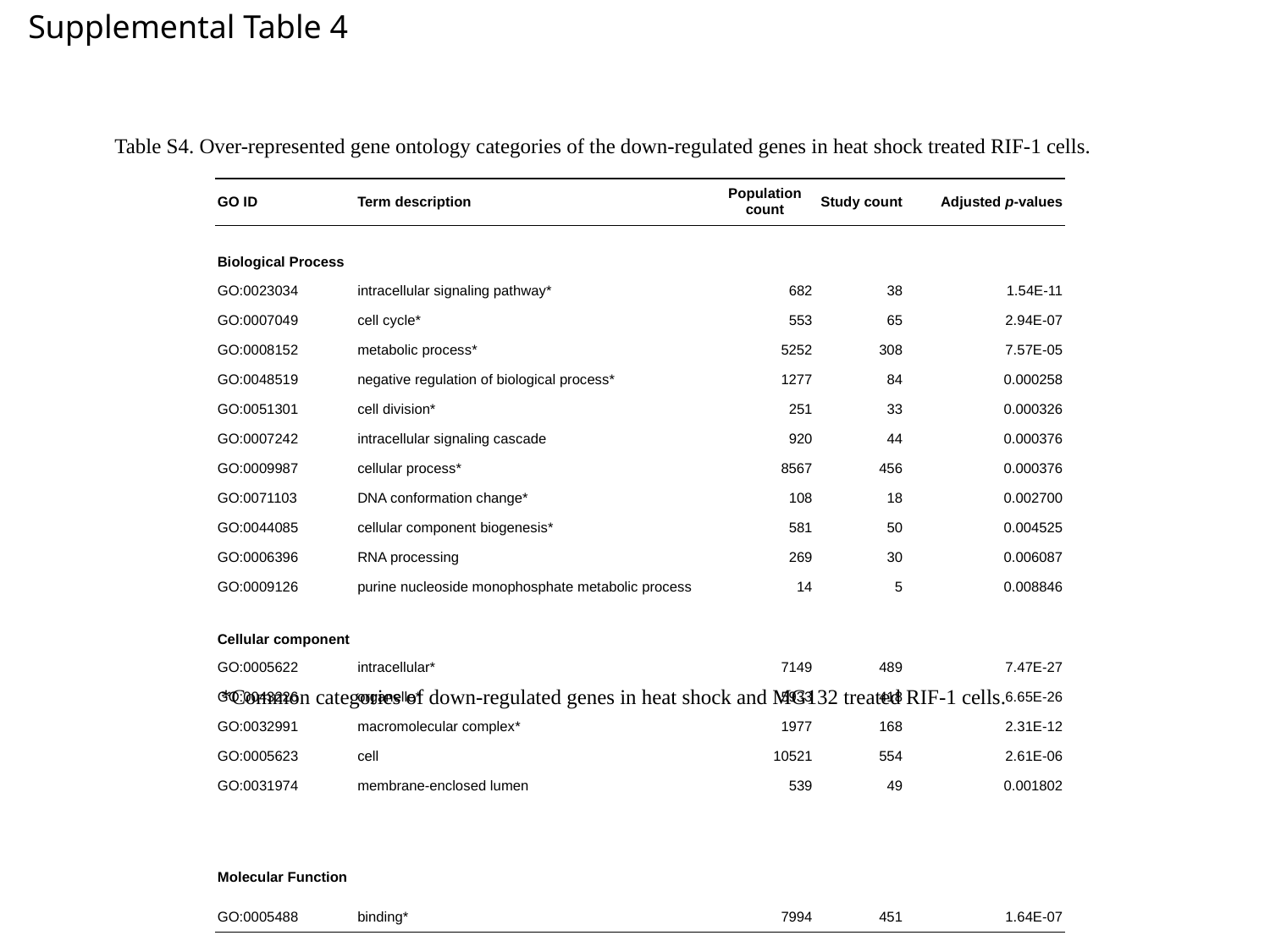

Supplemental Table 4
Table S4. Over-represented gene ontology categories of the down-regulated genes in heat shock treated RIF-1 cells.
| GO ID | Term description | Population count | Study count | Adjusted p-values |
| --- | --- | --- | --- | --- |
| Biological Process | | | | |
| GO:0023034 | intracellular signaling pathway\* | 682 | 38 | 1.54E-11 |
| GO:0007049 | cell cycle\* | 553 | 65 | 2.94E-07 |
| GO:0008152 | metabolic process\* | 5252 | 308 | 7.57E-05 |
| GO:0048519 | negative regulation of biological process\* | 1277 | 84 | 0.000258 |
| GO:0051301 | cell division\* | 251 | 33 | 0.000326 |
| GO:0007242 | intracellular signaling cascade | 920 | 44 | 0.000376 |
| GO:0009987 | cellular process\* | 8567 | 456 | 0.000376 |
| GO:0071103 | DNA conformation change\* | 108 | 18 | 0.002700 |
| GO:0044085 | cellular component biogenesis\* | 581 | 50 | 0.004525 |
| GO:0006396 | RNA processing | 269 | 30 | 0.006087 |
| GO:0009126 | purine nucleoside monophosphate metabolic process | 14 | 5 | 0.008846 |
| Cellular component | | | | |
| GO:0005622 | intracellular\* | 7149 | 489 | 7.47E-27 |
| GO:0043226 | organelle\* | 5933 | 418 | 6.65E-26 |
| GO:0032991 | macromolecular complex\* | 1977 | 168 | 2.31E-12 |
| GO:0005623 | cell | 10521 | 554 | 2.61E-06 |
| GO:0031974 | membrane-enclosed lumen | 539 | 49 | 0.001802 |
| | | | | |
| Molecular Function | | | | |
| GO:0005488 | binding\* | 7994 | 451 | 1.64E-07 |
*Common categories of down-regulated genes in heat shock and MG132 treated RIF-1 cells.
