# Supplementary material for: Systemic Analysis of Heat Shock Response Induced by Heat Shock and a Proteasome Inhibitor MG132
Source: PLoS One. 2011 Jun 30;6(6):e20252. doi: 10.1371/journal.pone.0020252 (PMC3127947; doi:10.1371/journal.pone.0020252)
Supplement: Table S5 — Over-represented gene ontology categories of the down-regulated genes in MG132 treated RIF-1 cells. (PPT) [file pone.0020252.s012.ppt]

## Slide 1
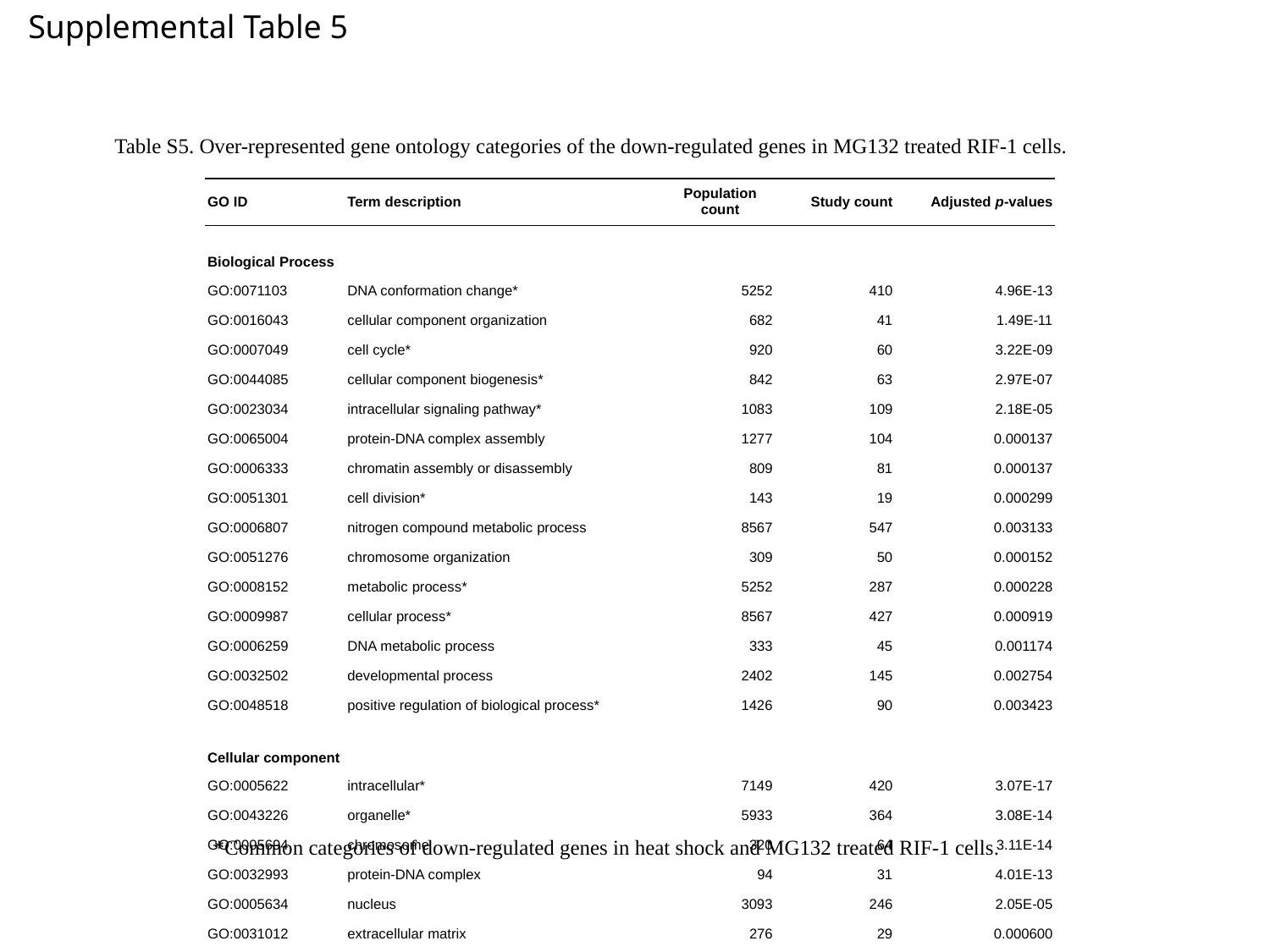

Supplemental Table 5
Table S5. Over-represented gene ontology categories of the down-regulated genes in MG132 treated RIF-1 cells.
| GO ID | Term description | Population count | Study count | Adjusted p-values |
| --- | --- | --- | --- | --- |
| Biological Process | | | | |
| GO:0071103 | DNA conformation change\* | 5252 | 410 | 4.96E-13 |
| GO:0016043 | cellular component organization | 682 | 41 | 1.49E-11 |
| GO:0007049 | cell cycle\* | 920 | 60 | 3.22E-09 |
| GO:0044085 | cellular component biogenesis\* | 842 | 63 | 2.97E-07 |
| GO:0023034 | intracellular signaling pathway\* | 1083 | 109 | 2.18E-05 |
| GO:0065004 | protein-DNA complex assembly | 1277 | 104 | 0.000137 |
| GO:0006333 | chromatin assembly or disassembly | 809 | 81 | 0.000137 |
| GO:0051301 | cell division\* | 143 | 19 | 0.000299 |
| GO:0006807 | nitrogen compound metabolic process | 8567 | 547 | 0.003133 |
| GO:0051276 | chromosome organization | 309 | 50 | 0.000152 |
| GO:0008152 | metabolic process\* | 5252 | 287 | 0.000228 |
| GO:0009987 | cellular process\* | 8567 | 427 | 0.000919 |
| GO:0006259 | DNA metabolic process | 333 | 45 | 0.001174 |
| GO:0032502 | developmental process | 2402 | 145 | 0.002754 |
| GO:0048518 | positive regulation of biological process\* | 1426 | 90 | 0.003423 |
| Cellular component | | | | |
| GO:0005622 | intracellular\* | 7149 | 420 | 3.07E-17 |
| GO:0043226 | organelle\* | 5933 | 364 | 3.08E-14 |
| GO:0005694 | chromosome | 320 | 64 | 3.11E-14 |
| GO:0032993 | protein-DNA complex | 94 | 31 | 4.01E-13 |
| GO:0005634 | nucleus | 3093 | 246 | 2.05E-05 |
| GO:0031012 | extracellular matrix | 276 | 29 | 0.000600 |
| GO:0032991 | macromolecular complex\* | 1977 | 127 | 0.000911 |
| | | | | |
| Molecular Function | | | | |
| GO:0003676 | nucleic acid binding | 1822 | 152 | 6.64E-09 |
| GO:0005488 | binding\* | 7994 | 411 | 4.78E-05 |
| GO:0030528 | transcription regulator activity | 892 | 66 | 0.002553 |
*Common categories of down-regulated genes in heat shock and MG132 treated RIF-1 cells.
