# Supplementary material for: Systemic Analysis of Heat Shock Response Induced by Heat Shock and a Proteasome Inhibitor MG132
Source: PLoS One. 2011 Jun 30;6(6):e20252. doi: 10.1371/journal.pone.0020252 (PMC3127947; doi:10.1371/journal.pone.0020252)
Supplement: Table S6 — Heat shock protein family members are listed with their mRNA levels in response to heat shock and MG132 treatment. Fold changes more than 2 are colored in red and less than -2 are colored in green. (PPT) [file pone.0020252.s013.ppt]

## Slide 1
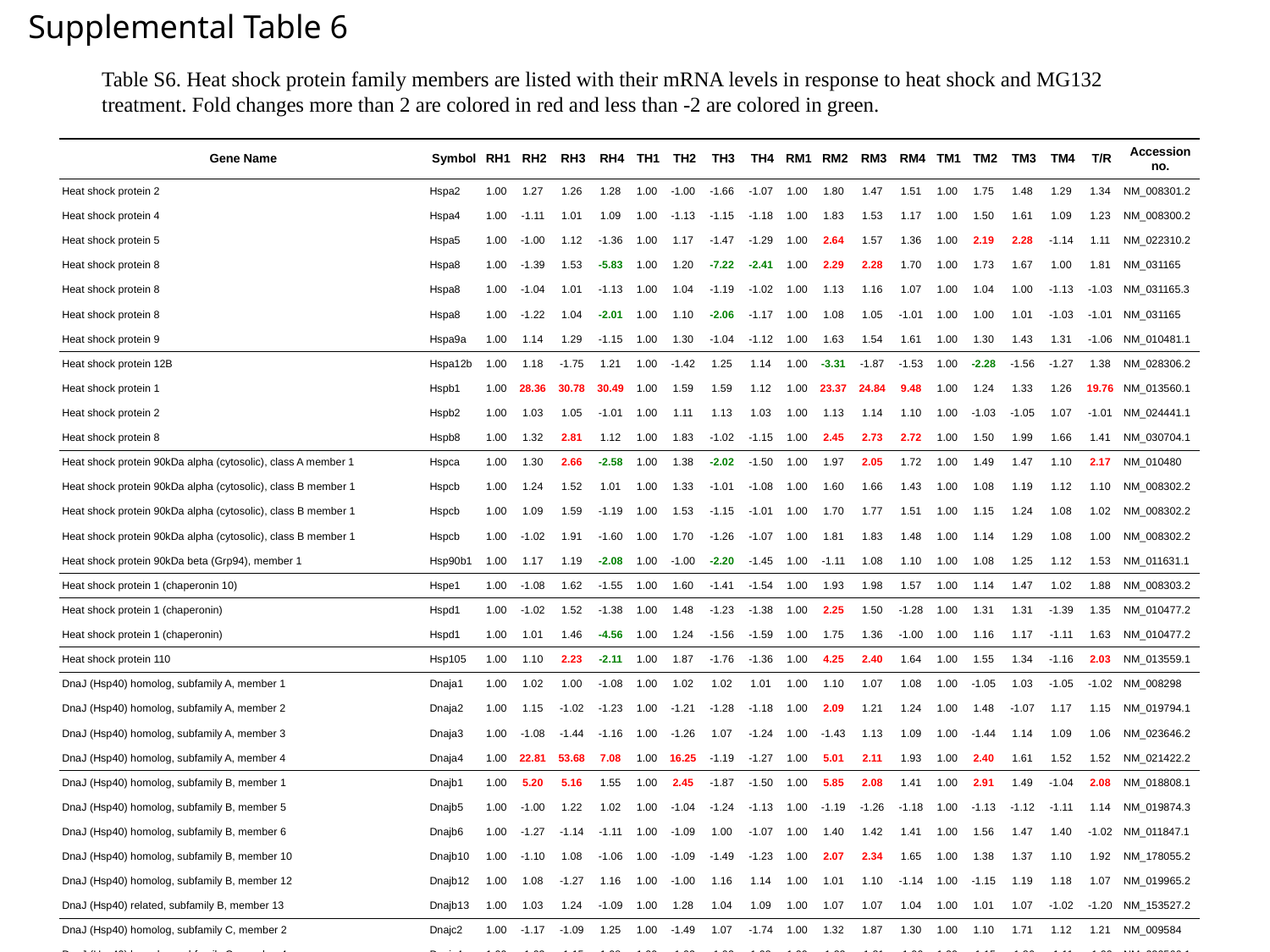

Supplemental Table 6
Table S6. Heat shock protein family members are listed with their mRNA levels in response to heat shock and MG132 treatment. Fold changes more than 2 are colored in red and less than -2 are colored in green.
| Gene Name | Symbol | RH1 | RH2 | RH3 | RH4 | TH1 | TH2 | TH3 | TH4 | RM1 | RM2 | RM3 | RM4 | TM1 | TM2 | TM3 | TM4 | T/R | Accession no. |
| --- | --- | --- | --- | --- | --- | --- | --- | --- | --- | --- | --- | --- | --- | --- | --- | --- | --- | --- | --- |
| Heat shock protein 2 | Hspa2 | 1.00 | 1.27 | 1.26 | 1.28 | 1.00 | -1.00 | -1.66 | -1.07 | 1.00 | 1.80 | 1.47 | 1.51 | 1.00 | 1.75 | 1.48 | 1.29 | 1.34 | NM\_008301.2 |
| Heat shock protein 4 | Hspa4 | 1.00 | -1.11 | 1.01 | 1.09 | 1.00 | -1.13 | -1.15 | -1.18 | 1.00 | 1.83 | 1.53 | 1.17 | 1.00 | 1.50 | 1.61 | 1.09 | 1.23 | NM\_008300.2 |
| Heat shock protein 5 | Hspa5 | 1.00 | -1.00 | 1.12 | -1.36 | 1.00 | 1.17 | -1.47 | -1.29 | 1.00 | 2.64 | 1.57 | 1.36 | 1.00 | 2.19 | 2.28 | -1.14 | 1.11 | NM\_022310.2 |
| Heat shock protein 8 | Hspa8 | 1.00 | -1.39 | 1.53 | -5.83 | 1.00 | 1.20 | -7.22 | -2.41 | 1.00 | 2.29 | 2.28 | 1.70 | 1.00 | 1.73 | 1.67 | 1.00 | 1.81 | NM\_031165 |
| Heat shock protein 8 | Hspa8 | 1.00 | -1.04 | 1.01 | -1.13 | 1.00 | 1.04 | -1.19 | -1.02 | 1.00 | 1.13 | 1.16 | 1.07 | 1.00 | 1.04 | 1.00 | -1.13 | -1.03 | NM\_031165.3 |
| Heat shock protein 8 | Hspa8 | 1.00 | -1.22 | 1.04 | -2.01 | 1.00 | 1.10 | -2.06 | -1.17 | 1.00 | 1.08 | 1.05 | -1.01 | 1.00 | 1.00 | 1.01 | -1.03 | -1.01 | NM\_031165 |
| Heat shock protein 9 | Hspa9a | 1.00 | 1.14 | 1.29 | -1.15 | 1.00 | 1.30 | -1.04 | -1.12 | 1.00 | 1.63 | 1.54 | 1.61 | 1.00 | 1.30 | 1.43 | 1.31 | -1.06 | NM\_010481.1 |
| Heat shock protein 12B | Hspa12b | 1.00 | 1.18 | -1.75 | 1.21 | 1.00 | -1.42 | 1.25 | 1.14 | 1.00 | -3.31 | -1.87 | -1.53 | 1.00 | -2.28 | -1.56 | -1.27 | 1.38 | NM\_028306.2 |
| Heat shock protein 1 | Hspb1 | 1.00 | 28.36 | 30.78 | 30.49 | 1.00 | 1.59 | 1.59 | 1.12 | 1.00 | 23.37 | 24.84 | 9.48 | 1.00 | 1.24 | 1.33 | 1.26 | 19.76 | NM\_013560.1 |
| Heat shock protein 2 | Hspb2 | 1.00 | 1.03 | 1.05 | -1.01 | 1.00 | 1.11 | 1.13 | 1.03 | 1.00 | 1.13 | 1.14 | 1.10 | 1.00 | -1.03 | -1.05 | 1.07 | -1.01 | NM\_024441.1 |
| Heat shock protein 8 | Hspb8 | 1.00 | 1.32 | 2.81 | 1.12 | 1.00 | 1.83 | -1.02 | -1.15 | 1.00 | 2.45 | 2.73 | 2.72 | 1.00 | 1.50 | 1.99 | 1.66 | 1.41 | NM\_030704.1 |
| Heat shock protein 90kDa alpha (cytosolic), class A member 1 | Hspca | 1.00 | 1.30 | 2.66 | -2.58 | 1.00 | 1.38 | -2.02 | -1.50 | 1.00 | 1.97 | 2.05 | 1.72 | 1.00 | 1.49 | 1.47 | 1.10 | 2.17 | NM\_010480 |
| Heat shock protein 90kDa alpha (cytosolic), class B member 1 | Hspcb | 1.00 | 1.24 | 1.52 | 1.01 | 1.00 | 1.33 | -1.01 | -1.08 | 1.00 | 1.60 | 1.66 | 1.43 | 1.00 | 1.08 | 1.19 | 1.12 | 1.10 | NM\_008302.2 |
| Heat shock protein 90kDa alpha (cytosolic), class B member 1 | Hspcb | 1.00 | 1.09 | 1.59 | -1.19 | 1.00 | 1.53 | -1.15 | -1.01 | 1.00 | 1.70 | 1.77 | 1.51 | 1.00 | 1.15 | 1.24 | 1.08 | 1.02 | NM\_008302.2 |
| Heat shock protein 90kDa alpha (cytosolic), class B member 1 | Hspcb | 1.00 | -1.02 | 1.91 | -1.60 | 1.00 | 1.70 | -1.26 | -1.07 | 1.00 | 1.81 | 1.83 | 1.48 | 1.00 | 1.14 | 1.29 | 1.08 | 1.00 | NM\_008302.2 |
| Heat shock protein 90kDa beta (Grp94), member 1 | Hsp90b1 | 1.00 | 1.17 | 1.19 | -2.08 | 1.00 | -1.00 | -2.20 | -1.45 | 1.00 | -1.11 | 1.08 | 1.10 | 1.00 | 1.08 | 1.25 | 1.12 | 1.53 | NM\_011631.1 |
| Heat shock protein 1 (chaperonin 10) | Hspe1 | 1.00 | -1.08 | 1.62 | -1.55 | 1.00 | 1.60 | -1.41 | -1.54 | 1.00 | 1.93 | 1.98 | 1.57 | 1.00 | 1.14 | 1.47 | 1.02 | 1.88 | NM\_008303.2 |
| Heat shock protein 1 (chaperonin) | Hspd1 | 1.00 | -1.02 | 1.52 | -1.38 | 1.00 | 1.48 | -1.23 | -1.38 | 1.00 | 2.25 | 1.50 | -1.28 | 1.00 | 1.31 | 1.31 | -1.39 | 1.35 | NM\_010477.2 |
| Heat shock protein 1 (chaperonin) | Hspd1 | 1.00 | 1.01 | 1.46 | -4.56 | 1.00 | 1.24 | -1.56 | -1.59 | 1.00 | 1.75 | 1.36 | -1.00 | 1.00 | 1.16 | 1.17 | -1.11 | 1.63 | NM\_010477.2 |
| Heat shock protein 110 | Hsp105 | 1.00 | 1.10 | 2.23 | -2.11 | 1.00 | 1.87 | -1.76 | -1.36 | 1.00 | 4.25 | 2.40 | 1.64 | 1.00 | 1.55 | 1.34 | -1.16 | 2.03 | NM\_013559.1 |
| DnaJ (Hsp40) homolog, subfamily A, member 1 | Dnaja1 | 1.00 | 1.02 | 1.00 | -1.08 | 1.00 | 1.02 | 1.02 | 1.01 | 1.00 | 1.10 | 1.07 | 1.08 | 1.00 | -1.05 | 1.03 | -1.05 | -1.02 | NM\_008298 |
| DnaJ (Hsp40) homolog, subfamily A, member 2 | Dnaja2 | 1.00 | 1.15 | -1.02 | -1.23 | 1.00 | -1.21 | -1.28 | -1.18 | 1.00 | 2.09 | 1.21 | 1.24 | 1.00 | 1.48 | -1.07 | 1.17 | 1.15 | NM\_019794.1 |
| DnaJ (Hsp40) homolog, subfamily A, member 3 | Dnaja3 | 1.00 | -1.08 | -1.44 | -1.16 | 1.00 | -1.26 | 1.07 | -1.24 | 1.00 | -1.43 | 1.13 | 1.09 | 1.00 | -1.44 | 1.14 | 1.09 | 1.06 | NM\_023646.2 |
| DnaJ (Hsp40) homolog, subfamily A, member 4 | Dnaja4 | 1.00 | 22.81 | 53.68 | 7.08 | 1.00 | 16.25 | -1.19 | -1.27 | 1.00 | 5.01 | 2.11 | 1.93 | 1.00 | 2.40 | 1.61 | 1.52 | 1.52 | NM\_021422.2 |
| DnaJ (Hsp40) homolog, subfamily B, member 1 | Dnajb1 | 1.00 | 5.20 | 5.16 | 1.55 | 1.00 | 2.45 | -1.87 | -1.50 | 1.00 | 5.85 | 2.08 | 1.41 | 1.00 | 2.91 | 1.49 | -1.04 | 2.08 | NM\_018808.1 |
| DnaJ (Hsp40) homolog, subfamily B, member 5 | Dnajb5 | 1.00 | -1.00 | 1.22 | 1.02 | 1.00 | -1.04 | -1.24 | -1.13 | 1.00 | -1.19 | -1.26 | -1.18 | 1.00 | -1.13 | -1.12 | -1.11 | 1.14 | NM\_019874.3 |
| DnaJ (Hsp40) homolog, subfamily B, member 6 | Dnajb6 | 1.00 | -1.27 | -1.14 | -1.11 | 1.00 | -1.09 | 1.00 | -1.07 | 1.00 | 1.40 | 1.42 | 1.41 | 1.00 | 1.56 | 1.47 | 1.40 | -1.02 | NM\_011847.1 |
| DnaJ (Hsp40) homolog, subfamily B, member 10 | Dnajb10 | 1.00 | -1.10 | 1.08 | -1.06 | 1.00 | -1.09 | -1.49 | -1.23 | 1.00 | 2.07 | 2.34 | 1.65 | 1.00 | 1.38 | 1.37 | 1.10 | 1.92 | NM\_178055.2 |
| DnaJ (Hsp40) homolog, subfamily B, member 12 | Dnajb12 | 1.00 | 1.08 | -1.27 | 1.16 | 1.00 | -1.00 | 1.16 | 1.14 | 1.00 | 1.01 | 1.10 | -1.14 | 1.00 | -1.15 | 1.19 | 1.18 | 1.07 | NM\_019965.2 |
| DnaJ (Hsp40) related, subfamily B, member 13 | Dnajb13 | 1.00 | 1.03 | 1.24 | -1.09 | 1.00 | 1.28 | 1.04 | 1.09 | 1.00 | 1.07 | 1.07 | 1.04 | 1.00 | 1.01 | 1.07 | -1.02 | -1.20 | NM\_153527.2 |
| DnaJ (Hsp40) homolog, subfamily C, member 2 | Dnajc2 | 1.00 | -1.17 | -1.09 | 1.25 | 1.00 | -1.49 | 1.07 | -1.74 | 1.00 | 1.32 | 1.87 | 1.30 | 1.00 | 1.10 | 1.71 | 1.12 | 1.21 | NM\_009584 |
| DnaJ (Hsp40) homolog, subfamily C, member 4 | Dnajc4 | 1.00 | -1.03 | -1.15 | 1.08 | 1.00 | -1.02 | -1.02 | 1.03 | 1.00 | -1.22 | -1.31 | -1.06 | 1.00 | -1.15 | -1.26 | -1.11 | -1.00 | NM\_020566.1 |
| DnaJ (Hsp40) homolog, subfamily C, member 5 | Dnajc5 | 1.00 | -1.03 | -1.04 | -1.08 | 1.00 | 1.06 | 1.09 | 1.22 | 1.00 | 1.05 | -1.15 | 1.02 | 1.00 | 1.06 | -1.36 | -1.12 | -1.29 | NM\_016775.2 |
| DnaJ (Hsp40) homolog, subfamily C, member 5 beta | Dnajc5b | 1.00 | -1.02 | 1.04 | 1.08 | 1.00 | -1.15 | -1.16 | -1.05 | 1.00 | -1.05 | 1.03 | 1.03 | 1.00 | -1.07 | 1.03 | -1.02 | 1.15 | NM\_025489.2 |
| DnaJ (Hsp40) homolog, subfamily C, member 7 | Dnajc7 | 1.00 | -1.22 | 1.16 | -1.13 | 1.00 | -1.22 | 1.34 | 1.08 | 1.00 | -1.11 | 1.04 | -1.00 | 1.00 | -1.19 | 1.01 | -1.20 | 1.26 | NM\_019795.3 |
| DnaJ (Hsp40) homolog, subfamily C, member 8 | Dnajc8 | 1.00 | -1.05 | 1.02 | 1.11 | 1.00 | 1.08 | -1.17 | -1.07 | 1.00 | 1.02 | 1.11 | 1.08 | 1.00 | 1.03 | 1.16 | 1.22 | 1.15 | NM\_172400 |
| DnaJ (Hsp40) homolog, subfamily C, member 11 | Dnajc11 | 1.00 | 1.14 | 1.03 | 1.12 | 1.00 | 1.10 | -1.18 | -1.06 | 1.00 | 1.01 | -1.01 | -1.00 | 1.00 | 1.17 | 1.12 | 1.07 | -1.02 | NM\_172704.1 |
| DnaJ (Hsp40) homolog, subfamily C, member 11 | Dnajc11 | 1.00 | 1.05 | 1.10 | 1.96 | 1.00 | 1.05 | 1.19 | 1.02 | 1.00 | -1.07 | 1.07 | 1.12 | 1.00 | 1.01 | 1.19 | 1.10 | -1.11 | NM\_172704.1 |
| DnaJ (Hsp40) homolog, subfamily C, member 13 | Dnajc13 | 1.00 | 1.16 | 1.50 | 1.08 | 1.00 | 1.26 | 1.06 | 1.14 | 1.00 | 1.15 | 1.35 | 1.49 | 1.00 | 1.16 | 1.12 | 1.23 | -1.12 | XM\_135146.4 |
| DnaJ (Hsp40) homolog, subfamily C, member 14 | Dnajc14 | 1.00 | -1.05 | 1.03 | -1.09 | 1.00 | -1.06 | -1.01 | -1.01 | 1.00 | -1.09 | -1.10 | -1.08 | 1.00 | -1.05 | 1.01 | 1.07 | 1.01 | NM\_028873.2 |
| DnaJ (Hsp40) homolog, subfamily C, member 16 | Dnajc16 | 1.00 | -1.26 | -1.48 | 1.11 | 1.00 | -1.64 | 1.32 | -1.07 | 1.00 | -1.55 | 1.35 | 1.54 | 1.00 | -1.37 | 1.29 | 1.41 | -1.70 | NM\_172338.1 |
| DnaJ (Hsp40) homolog, subfamily C, member 17 | Dnajc17 | 1.00 | -1.01 | -1.41 | -1.29 | 1.00 | -1.32 | -1.20 | -1.10 | 1.00 | -1.05 | 1.14 | -1.02 | 1.00 | -1.08 | 1.00 | -1.11 | -1.34 | NM\_139139.1 |
| DnaJ (Hsp40) homolog, subfamily C, member 18 | Dnajc18 | 1.00 | -1.05 | -1.24 | 1.02 | 1.00 | -1.27 | 1.03 | 1.00 | 1.00 | -1.07 | -1.03 | 1.17 | 1.00 | 1.01 | 1.09 | 1.22 | 1.09 | NM\_029669.2 |
| Crystallin, alpha B | Cryab | 1.00 | 3.66 | 12.77 | 9.18 | 1.00 | 2.58 | 2.41 | 1.19 | 1.00 | 7.43 | 14.43 | 11.39 | 1.00 | 2.06 | 3.47 | 3.34 | 5.33 | NM\_009964.1 |
| Crystallin, beta A4 | Cryba4 | 1.00 | -1.26 | 2.87 | 1.33 | 1.00 | 2.67 | 1.09 | -1.18 | 1.00 | 2.18 | -1.30 | -1.03 | 1.00 | 1.24 | -1.66 | -1.23 | 1.50 | NM\_021351.1 |
| Crystallin, beta B1 | Crybb1 | 1.00 | -1.09 | 1.16 | -1.00 | 1.00 | 1.13 | 1.22 | 1.13 | 1.00 | 1.07 | -1.18 | -1.19 | 1.00 | -1.12 | -1.52 | -1.87 | 2.23 | NM\_023695.1 |
| Crystallin, beta B3 | Crybb3 | 1.00 | 1.00 | 1.13 | 1.02 | 1.00 | 1.17 | -1.04 | -1.13 | 1.00 | -1.05 | 1.04 | 1.16 | 1.00 | -1.01 | 1.01 | 1.01 | -1.03 | NM\_021352.2 |
