# Supplementary material for: Systemic Analysis of Heat Shock Response Induced by Heat Shock and a Proteasome Inhibitor MG132
Source: PLoS One. 2011 Jun 30;6(6):e20252. doi: 10.1371/journal.pone.0020252 (PMC3127947; doi:10.1371/journal.pone.0020252)
Supplement: Table S7 — Histone family members are listed with their mRNA levels in response to heat shock and MG132 treatment. Fold changes more than 2 are colored in red and less than -2 are colored in green. (PPT) [file pone.0020252.s014.ppt]

## Slide 1
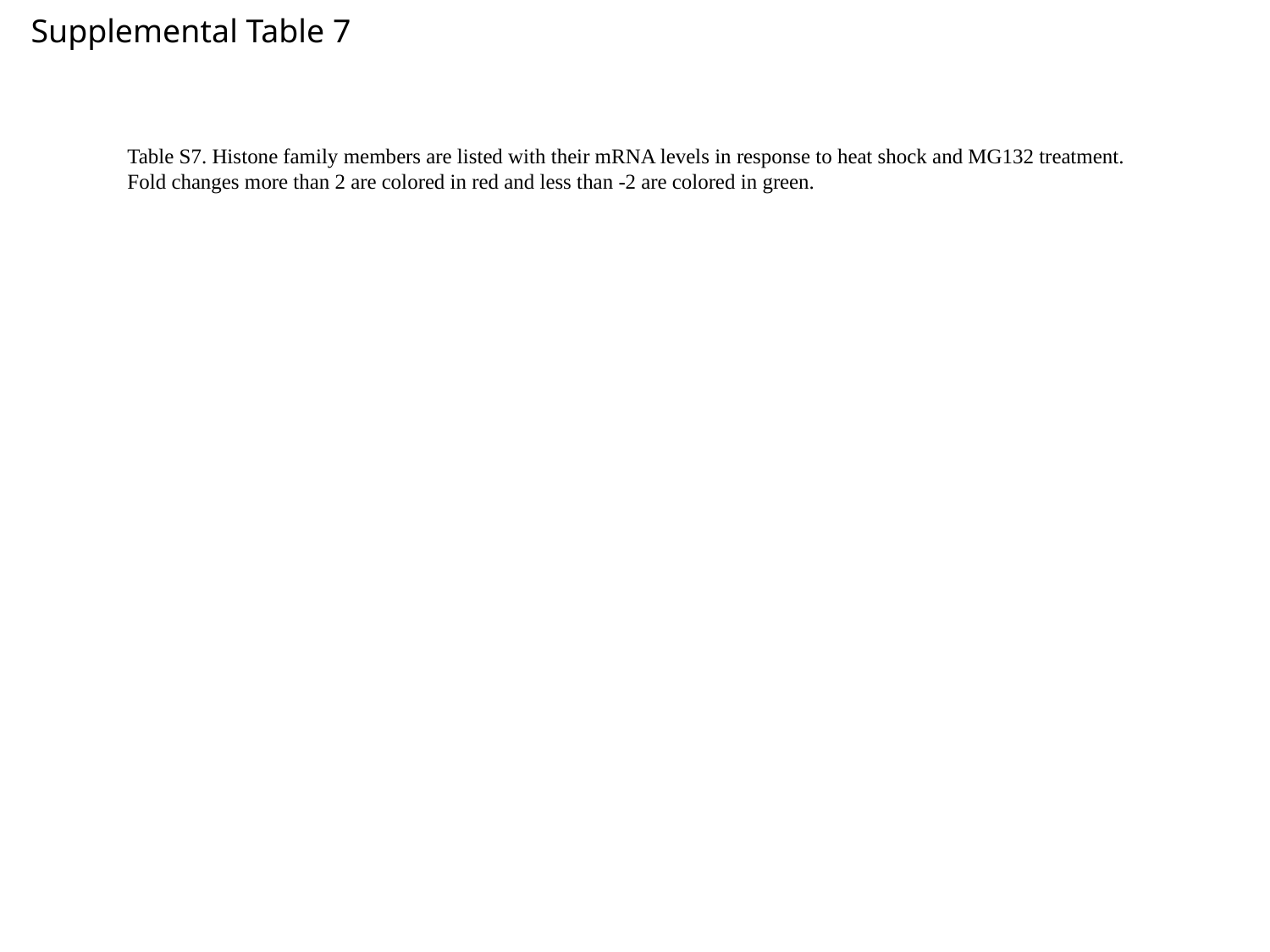

Supplemental Table 7
Table S7. Histone family members are listed with their mRNA levels in response to heat shock and MG132 treatment. Fold changes more than 2 are colored in red and less than -2 are colored in green.

## Slide 2
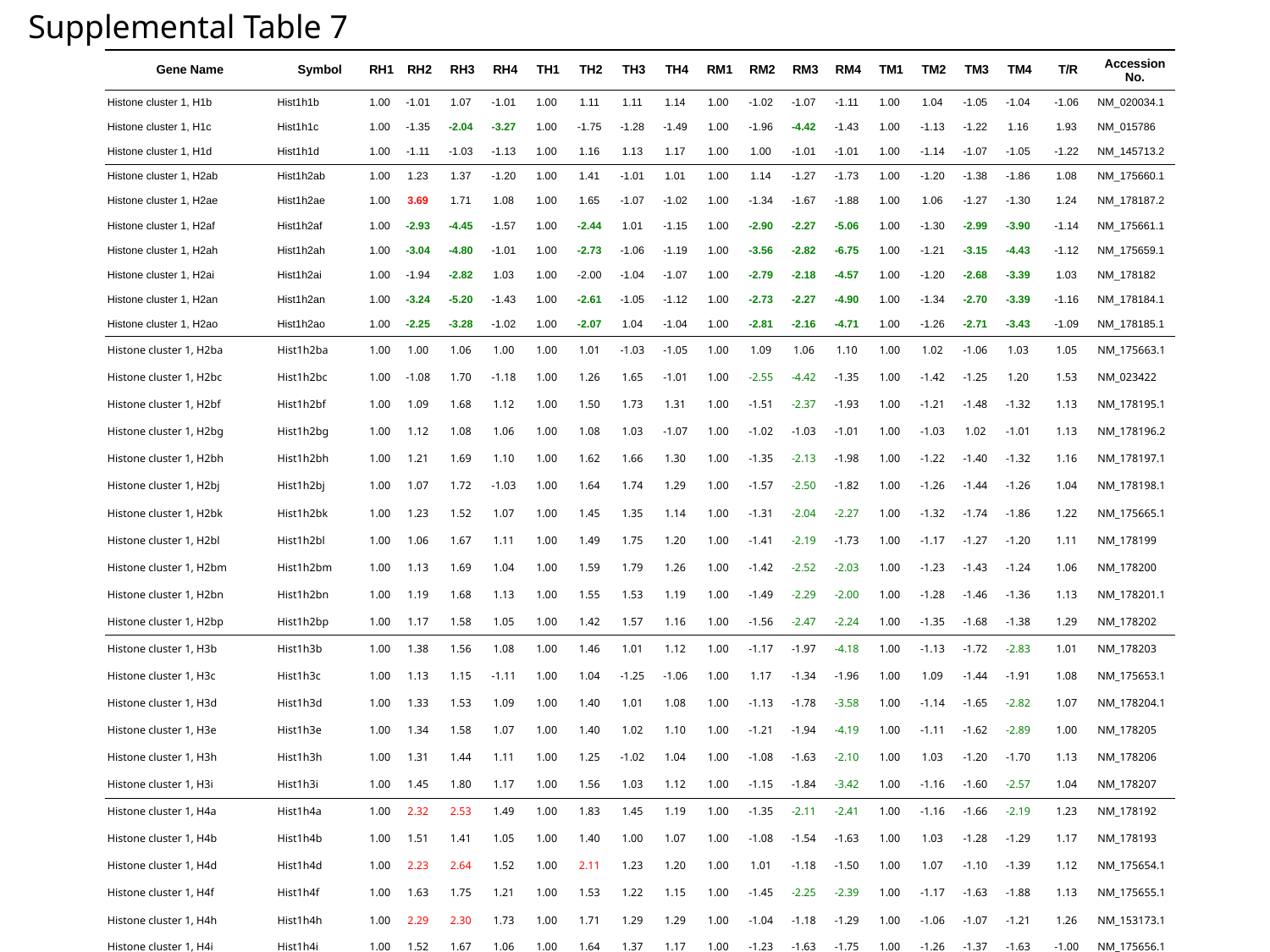

Supplemental Table 7
| Gene Name | Symbol | RH1 | RH2 | RH3 | RH4 | TH1 | TH2 | TH3 | TH4 | RM1 | RM2 | RM3 | RM4 | TM1 | TM2 | TM3 | TM4 | T/R | Accession No. |
| --- | --- | --- | --- | --- | --- | --- | --- | --- | --- | --- | --- | --- | --- | --- | --- | --- | --- | --- | --- |
| Histone cluster 1, H1b | Hist1h1b | 1.00 | -1.01 | 1.07 | -1.01 | 1.00 | 1.11 | 1.11 | 1.14 | 1.00 | -1.02 | -1.07 | -1.11 | 1.00 | 1.04 | -1.05 | -1.04 | -1.06 | NM\_020034.1 |
| Histone cluster 1, H1c | Hist1h1c | 1.00 | -1.35 | -2.04 | -3.27 | 1.00 | -1.75 | -1.28 | -1.49 | 1.00 | -1.96 | -4.42 | -1.43 | 1.00 | -1.13 | -1.22 | 1.16 | 1.93 | NM\_015786 |
| Histone cluster 1, H1d | Hist1h1d | 1.00 | -1.11 | -1.03 | -1.13 | 1.00 | 1.16 | 1.13 | 1.17 | 1.00 | 1.00 | -1.01 | -1.01 | 1.00 | -1.14 | -1.07 | -1.05 | -1.22 | NM\_145713.2 |
| Histone cluster 1, H2ab | Hist1h2ab | 1.00 | 1.23 | 1.37 | -1.20 | 1.00 | 1.41 | -1.01 | 1.01 | 1.00 | 1.14 | -1.27 | -1.73 | 1.00 | -1.20 | -1.38 | -1.86 | 1.08 | NM\_175660.1 |
| Histone cluster 1, H2ae | Hist1h2ae | 1.00 | 3.69 | 1.71 | 1.08 | 1.00 | 1.65 | -1.07 | -1.02 | 1.00 | -1.34 | -1.67 | -1.88 | 1.00 | 1.06 | -1.27 | -1.30 | 1.24 | NM\_178187.2 |
| Histone cluster 1, H2af | Hist1h2af | 1.00 | -2.93 | -4.45 | -1.57 | 1.00 | -2.44 | 1.01 | -1.15 | 1.00 | -2.90 | -2.27 | -5.06 | 1.00 | -1.30 | -2.99 | -3.90 | -1.14 | NM\_175661.1 |
| Histone cluster 1, H2ah | Hist1h2ah | 1.00 | -3.04 | -4.80 | -1.01 | 1.00 | -2.73 | -1.06 | -1.19 | 1.00 | -3.56 | -2.82 | -6.75 | 1.00 | -1.21 | -3.15 | -4.43 | -1.12 | NM\_175659.1 |
| Histone cluster 1, H2ai | Hist1h2ai | 1.00 | -1.94 | -2.82 | 1.03 | 1.00 | -2.00 | -1.04 | -1.07 | 1.00 | -2.79 | -2.18 | -4.57 | 1.00 | -1.20 | -2.68 | -3.39 | 1.03 | NM\_178182 |
| Histone cluster 1, H2an | Hist1h2an | 1.00 | -3.24 | -5.20 | -1.43 | 1.00 | -2.61 | -1.05 | -1.12 | 1.00 | -2.73 | -2.27 | -4.90 | 1.00 | -1.34 | -2.70 | -3.39 | -1.16 | NM\_178184.1 |
| Histone cluster 1, H2ao | Hist1h2ao | 1.00 | -2.25 | -3.28 | -1.02 | 1.00 | -2.07 | 1.04 | -1.04 | 1.00 | -2.81 | -2.16 | -4.71 | 1.00 | -1.26 | -2.71 | -3.43 | -1.09 | NM\_178185.1 |
| Histone cluster 1, H2ba | Hist1h2ba | 1.00 | 1.00 | 1.06 | 1.00 | 1.00 | 1.01 | -1.03 | -1.05 | 1.00 | 1.09 | 1.06 | 1.10 | 1.00 | 1.02 | -1.06 | 1.03 | 1.05 | NM\_175663.1 |
| Histone cluster 1, H2bc | Hist1h2bc | 1.00 | -1.08 | 1.70 | -1.18 | 1.00 | 1.26 | 1.65 | -1.01 | 1.00 | -2.55 | -4.42 | -1.35 | 1.00 | -1.42 | -1.25 | 1.20 | 1.53 | NM\_023422 |
| Histone cluster 1, H2bf | Hist1h2bf | 1.00 | 1.09 | 1.68 | 1.12 | 1.00 | 1.50 | 1.73 | 1.31 | 1.00 | -1.51 | -2.37 | -1.93 | 1.00 | -1.21 | -1.48 | -1.32 | 1.13 | NM\_178195.1 |
| Histone cluster 1, H2bg | Hist1h2bg | 1.00 | 1.12 | 1.08 | 1.06 | 1.00 | 1.08 | 1.03 | -1.07 | 1.00 | -1.02 | -1.03 | -1.01 | 1.00 | -1.03 | 1.02 | -1.01 | 1.13 | NM\_178196.2 |
| Histone cluster 1, H2bh | Hist1h2bh | 1.00 | 1.21 | 1.69 | 1.10 | 1.00 | 1.62 | 1.66 | 1.30 | 1.00 | -1.35 | -2.13 | -1.98 | 1.00 | -1.22 | -1.40 | -1.32 | 1.16 | NM\_178197.1 |
| Histone cluster 1, H2bj | Hist1h2bj | 1.00 | 1.07 | 1.72 | -1.03 | 1.00 | 1.64 | 1.74 | 1.29 | 1.00 | -1.57 | -2.50 | -1.82 | 1.00 | -1.26 | -1.44 | -1.26 | 1.04 | NM\_178198.1 |
| Histone cluster 1, H2bk | Hist1h2bk | 1.00 | 1.23 | 1.52 | 1.07 | 1.00 | 1.45 | 1.35 | 1.14 | 1.00 | -1.31 | -2.04 | -2.27 | 1.00 | -1.32 | -1.74 | -1.86 | 1.22 | NM\_175665.1 |
| Histone cluster 1, H2bl | Hist1h2bl | 1.00 | 1.06 | 1.67 | 1.11 | 1.00 | 1.49 | 1.75 | 1.20 | 1.00 | -1.41 | -2.19 | -1.73 | 1.00 | -1.17 | -1.27 | -1.20 | 1.11 | NM\_178199 |
| Histone cluster 1, H2bm | Hist1h2bm | 1.00 | 1.13 | 1.69 | 1.04 | 1.00 | 1.59 | 1.79 | 1.26 | 1.00 | -1.42 | -2.52 | -2.03 | 1.00 | -1.23 | -1.43 | -1.24 | 1.06 | NM\_178200 |
| Histone cluster 1, H2bn | Hist1h2bn | 1.00 | 1.19 | 1.68 | 1.13 | 1.00 | 1.55 | 1.53 | 1.19 | 1.00 | -1.49 | -2.29 | -2.00 | 1.00 | -1.28 | -1.46 | -1.36 | 1.13 | NM\_178201.1 |
| Histone cluster 1, H2bp | Hist1h2bp | 1.00 | 1.17 | 1.58 | 1.05 | 1.00 | 1.42 | 1.57 | 1.16 | 1.00 | -1.56 | -2.47 | -2.24 | 1.00 | -1.35 | -1.68 | -1.38 | 1.29 | NM\_178202 |
| Histone cluster 1, H3b | Hist1h3b | 1.00 | 1.38 | 1.56 | 1.08 | 1.00 | 1.46 | 1.01 | 1.12 | 1.00 | -1.17 | -1.97 | -4.18 | 1.00 | -1.13 | -1.72 | -2.83 | 1.01 | NM\_178203 |
| Histone cluster 1, H3c | Hist1h3c | 1.00 | 1.13 | 1.15 | -1.11 | 1.00 | 1.04 | -1.25 | -1.06 | 1.00 | 1.17 | -1.34 | -1.96 | 1.00 | 1.09 | -1.44 | -1.91 | 1.08 | NM\_175653.1 |
| Histone cluster 1, H3d | Hist1h3d | 1.00 | 1.33 | 1.53 | 1.09 | 1.00 | 1.40 | 1.01 | 1.08 | 1.00 | -1.13 | -1.78 | -3.58 | 1.00 | -1.14 | -1.65 | -2.82 | 1.07 | NM\_178204.1 |
| Histone cluster 1, H3e | Hist1h3e | 1.00 | 1.34 | 1.58 | 1.07 | 1.00 | 1.40 | 1.02 | 1.10 | 1.00 | -1.21 | -1.94 | -4.19 | 1.00 | -1.11 | -1.62 | -2.89 | 1.00 | NM\_178205 |
| Histone cluster 1, H3h | Hist1h3h | 1.00 | 1.31 | 1.44 | 1.11 | 1.00 | 1.25 | -1.02 | 1.04 | 1.00 | -1.08 | -1.63 | -2.10 | 1.00 | 1.03 | -1.20 | -1.70 | 1.13 | NM\_178206 |
| Histone cluster 1, H3i | Hist1h3i | 1.00 | 1.45 | 1.80 | 1.17 | 1.00 | 1.56 | 1.03 | 1.12 | 1.00 | -1.15 | -1.84 | -3.42 | 1.00 | -1.16 | -1.60 | -2.57 | 1.04 | NM\_178207 |
| Histone cluster 1, H4a | Hist1h4a | 1.00 | 2.32 | 2.53 | 1.49 | 1.00 | 1.83 | 1.45 | 1.19 | 1.00 | -1.35 | -2.11 | -2.41 | 1.00 | -1.16 | -1.66 | -2.19 | 1.23 | NM\_178192 |
| Histone cluster 1, H4b | Hist1h4b | 1.00 | 1.51 | 1.41 | 1.05 | 1.00 | 1.40 | 1.00 | 1.07 | 1.00 | -1.08 | -1.54 | -1.63 | 1.00 | 1.03 | -1.28 | -1.29 | 1.17 | NM\_178193 |
| Histone cluster 1, H4d | Hist1h4d | 1.00 | 2.23 | 2.64 | 1.52 | 1.00 | 2.11 | 1.23 | 1.20 | 1.00 | 1.01 | -1.18 | -1.50 | 1.00 | 1.07 | -1.10 | -1.39 | 1.12 | NM\_175654.1 |
| Histone cluster 1, H4f | Hist1h4f | 1.00 | 1.63 | 1.75 | 1.21 | 1.00 | 1.53 | 1.22 | 1.15 | 1.00 | -1.45 | -2.25 | -2.39 | 1.00 | -1.17 | -1.63 | -1.88 | 1.13 | NM\_175655.1 |
| Histone cluster 1, H4h | Hist1h4h | 1.00 | 2.29 | 2.30 | 1.73 | 1.00 | 1.71 | 1.29 | 1.29 | 1.00 | -1.04 | -1.18 | -1.29 | 1.00 | -1.06 | -1.07 | -1.21 | 1.26 | NM\_153173.1 |
| Histone cluster 1, H4i | Hist1h4i | 1.00 | 1.52 | 1.67 | 1.06 | 1.00 | 1.64 | 1.37 | 1.17 | 1.00 | -1.23 | -1.63 | -1.75 | 1.00 | -1.26 | -1.37 | -1.63 | -1.00 | NM\_175656.1 |
| Histone cluster 1, H4j | Hist1h4j | 1.00 | 2.48 | 2.89 | 1.55 | 1.00 | 2.15 | 1.46 | 1.17 | 1.00 | 1.23 | 1.08 | -1.37 | 1.00 | 1.12 | -1.09 | -1.65 | 1.18 | NM\_178210.1 |
| Histone cluster 1, H4k | Hist1h4k | 1.00 | 2.84 | 3.58 | 1.63 | 1.00 | 2.32 | 1.41 | 1.33 | 1.00 | 1.02 | -1.14 | -1.44 | 1.00 | 1.06 | -1.08 | -1.35 | 1.14 | NM\_178211.1 |
| Histone cluster 1, H4m | Hist1h4m | 1.00 | 2.03 | 2.27 | 1.43 | 1.00 | 1.94 | 1.33 | 1.18 | 1.00 | -1.30 | -1.47 | -1.49 | 1.00 | -1.18 | -1.30 | -1.45 | 1.09 | NM\_175657 |
| Histone cluster 2, H2aa2 | Hist2h2aa1 | 1.00 | 1.06 | 1.02 | 1.11 | 1.00 | -1.09 | 1.09 | 1.10 | 1.00 | -1.01 | -1.06 | -1.01 | 1.00 | -1.05 | 1.03 | 1.06 | 1.25 | NM\_013549.1 |
| Histone cluster 2, H2aa2 | Hist2h2aa2 | 1.00 | 1.34 | 1.87 | 1.55 | 1.00 | 1.29 | 1.37 | 1.19 | 1.00 | -1.10 | -1.20 | -1.24 | 1.00 | -1.02 | -1.08 | -1.01 | 1.45 | NM\_178212 |
| Histone cluster 2, H2ac | Hist2h2ac | 1.00 | -2.22 | -3.36 | -1.04 | 1.00 | -2.12 | -1.04 | -1.09 | 1.00 | -2.90 | -2.39 | -4.62 | 1.00 | -1.38 | -3.11 | -3.45 | -1.04 | NM\_175662 |
| Histone cluster 3, H2ba | Hist3h2ba | 1.00 | 1.12 | 1.17 | -1.01 | 1.00 | 1.12 | 1.05 | -1.04 | 1.00 | -1.00 | -1.11 | 1.05 | 1.00 | -1.06 | -1.04 | 1.00 | -1.07 | NM\_030082.1 |
| Histone cluster 2, H2be | Hist2h2be | 1.00 | 1.48 | 1.92 | 1.19 | 1.00 | 1.71 | 1.13 | 1.18 | 1.00 | -1.06 | -1.66 | -2.94 | 1.00 | -1.05 | -1.39 | -2.21 | -1.03 | NM\_178214.1 |
| Histone cluster 2, H3b | Hist2h3b | 1.00 | 1.42 | 1.78 | 1.10 | 1.00 | 1.49 | 1.05 | 1.07 | 1.00 | 1.04 | -1.41 | -2.21 | 1.00 | 1.08 | -1.20 | -1.95 | 1.08 | NM\_178215 |
| Histone cluster 2, H4 | Hist2h4 | 1.00 | 1.10 | 1.22 | 1.00 | 1.00 | 1.11 | -1.05 | -1.10 | 1.00 | -1.16 | -1.22 | -1.27 | 1.00 | -1.04 | -1.21 | -1.20 | 1.08 | NM\_033596 |
| Histone cluster 4, H4 | Hist4h4 | 1.00 | 1.86 | 1.97 | 1.26 | 1.00 | 1.49 | -1.05 | 1.07 | 1.00 | 1.02 | -1.16 | -1.15 | 1.00 | 1.00 | -1.20 | -1.23 | 1.32 | NM\_175652.1 |
| Centromere protein A | Cenpa | 1.00 | -1.10 | -1.48 | -1.58 | 1.00 | -1.22 | -1.44 | 1.25 | 1.00 | -1.80 | -1.65 | -1.98 | 1.00 | -1.62 | -1.46 | -2.48 | -1.01 | NM\_007681.1 |
| H1 histone family, member X | H1fx | 1.00 | 1.15 | -1.21 | -1.18 | 1.00 | 1.24 | -1.04 | 1.61 | 1.00 | -1.33 | -1.86 | -1.99 | 1.00 | -1.27 | -2.29 | -2.26 | -1.02 | NM\_198622.1 |
| H2A histone family, member J | H2afj | 1.00 | 1.34 | 4.43 | 1.59 | 1.00 | 2.20 | 1.19 | 1.10 | 1.00 | -1.08 | -1.12 | 1.10 | 1.00 | -1.22 | -1.17 | 1.11 | -1.18 | NM\_177688.2 |
| H2A histone family, member X | H2afx | 1.00 | -1.31 | -2.82 | -2.30 | 1.00 | -1.42 | -1.28 | -1.05 | 1.00 | -1.65 | -3.25 | -3.78 | 1.00 | -1.83 | -2.42 | -2.46 | -1.19 | NM\_010436.2 |
| H2A histone family, member Y | H2afy | 1.00 | 1.23 | 1.12 | 1.04 | 1.00 | 1.05 | -1.03 | 1.25 | 1.00 | -1.05 | 1.01 | 1.01 | 1.00 | 1.13 | -1.07 | 1.11 | -1.07 | NM\_012015.1 |
| H2A histone family, member Z | H2afz | 1.00 | -1.06 | -1.23 | -5.35 | 1.00 | -1.08 | -1.64 | -1.09 | 1.00 | 1.15 | -1.50 | -1.64 | 1.00 | 1.22 | -1.27 | -1.56 | 1.05 | NM\_016750.1 |
| H3 histone, family 3A | H3f3a | 1.00 | -1.00 | -1.29 | -1.51 | 1.00 | -1.11 | -1.65 | -1.09 | 1.00 | -1.39 | -1.97 | -1.39 | 1.00 | -1.08 | -1.51 | -1.42 | 1.27 | NM\_008210.2 |
| H3 histone, family 3B | H3f3b | 1.00 | -1.22 | -1.18 | -2.09 | 1.00 | -1.11 | -1.40 | -1.25 | 1.00 | 1.12 | -1.48 | -1.62 | 1.00 | 1.09 | -1.27 | -1.49 | 1.23 | NM\_008211.2 |
| Metastasis associated 1 | Mta1 | 1.00 | 1.04 | -1.11 | -1.02 | 1.00 | 1.22 | 1.21 | 1.36 | 1.00 | 1.14 | -1.28 | -1.20 | 1.00 | -1.14 | -1.34 | -1.18 | -1.21 | NM\_054081.1 |
| Metastasis-associated 2 | Mta2 | 1.00 | -1.12 | 1.06 | 1.03 | 1.00 | -1.00 | 1.10 | -1.10 | 1.00 | 1.03 | -1.32 | -1.14 | 1.00 | -1.06 | -1.28 | -1.06 | -1.14 | NM\_011842.2 |
| Metastasis associated 3 | Mta3 | 1.00 | 1.20 | -1.13 | 1.30 | 1.00 | 1.06 | 1.21 | 1.57 | 1.00 | -1.17 | 1.13 | -1.13 | 1.00 | -1.06 | 1.07 | 1.14 | -1.70 | NM\_054082.1 |
| RIKEN cDNA 1700113O17 gene | 1700113O17Rik | 1.00 | 1.09 | 1.17 | -1.01 | 1.00 | 1.32 | 1.16 | 1.08 | 1.00 | 1.06 | 1.02 | -1.06 | 1.00 | 1.08 | -1.05 | -1.09 | -1.15 | NM\_026627.1 |
