# Supplementary material for: Systemic Analysis of Heat Shock Response Induced by Heat Shock and a Proteasome Inhibitor MG132
Source: PLoS One. 2011 Jun 30;6(6):e20252. doi: 10.1371/journal.pone.0020252 (PMC3127947; doi:10.1371/journal.pone.0020252)
Supplement: Table S8 — Heat shock and MG132 induced genes more than 3 fold in comparison to control cells are listed. Fold changes more than 2 are colored in red and less than -2 are colored in green. T/R means fold differences in TR cells compared to RIF-1 cells. (PPT) [file pone.0020252.s015.ppt]

## Slide 1
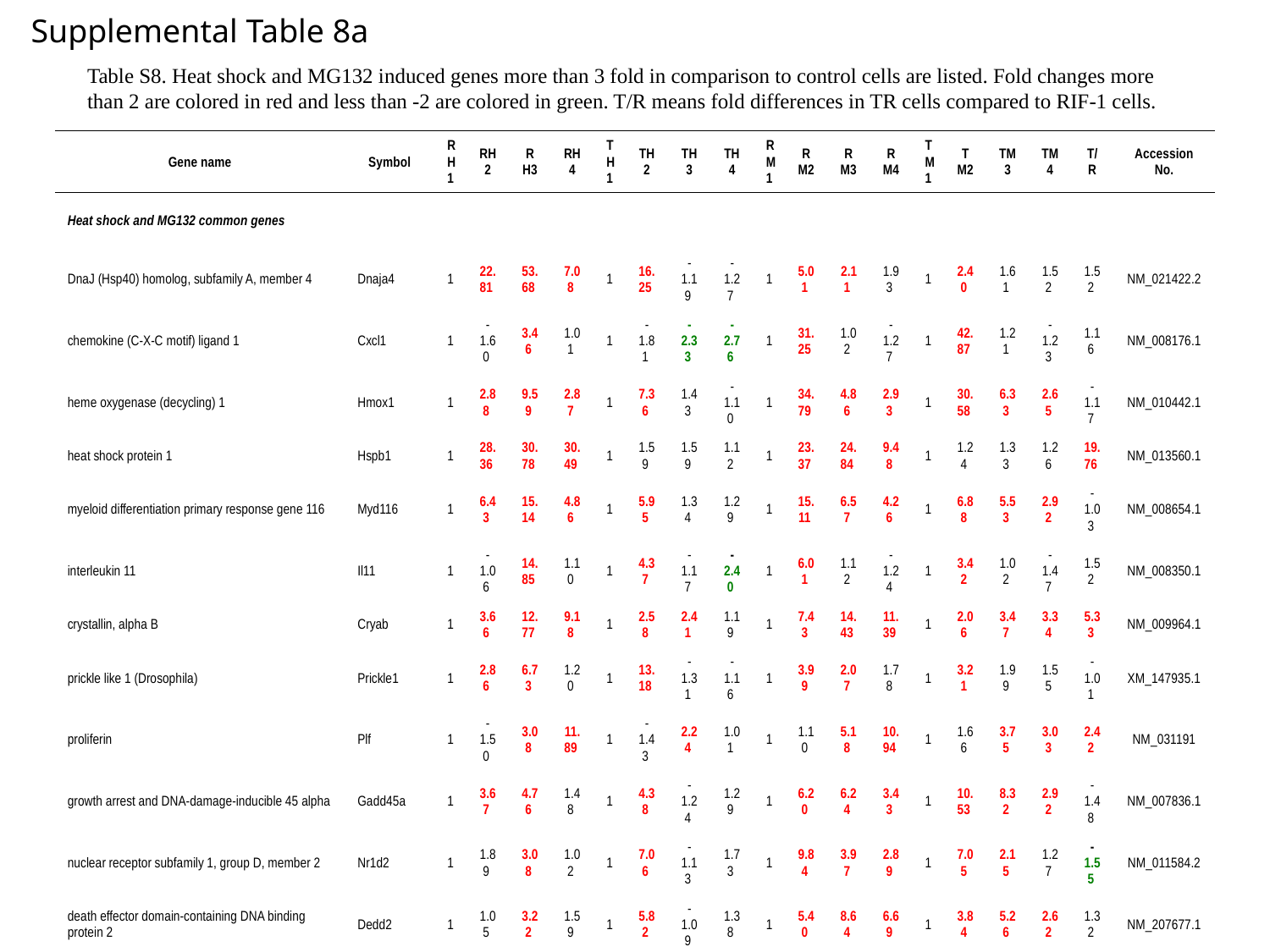

Supplemental Table 8a
Table S8. Heat shock and MG132 induced genes more than 3 fold in comparison to control cells are listed. Fold changes more than 2 are colored in red and less than -2 are colored in green. T/R means fold differences in TR cells compared to RIF-1 cells.
| Gene name | Symbol | RH1 | RH2 | RH3 | RH4 | TH1 | TH2 | TH3 | TH4 | RM1 | RM2 | RM3 | RM4 | TM1 | TM2 | TM3 | TM4 | T/R | Accession No. |
| --- | --- | --- | --- | --- | --- | --- | --- | --- | --- | --- | --- | --- | --- | --- | --- | --- | --- | --- | --- |
| Heat shock and MG132 common genes | | | | | | | | | | | | | | | | | | | |
| DnaJ (Hsp40) homolog, subfamily A, member 4 | Dnaja4 | 1 | 22.81 | 53.68 | 7.08 | 1 | 16.25 | -1.19 | -1.27 | 1 | 5.01 | 2.11 | 1.93 | 1 | 2.40 | 1.61 | 1.52 | 1.52 | NM\_021422.2 |
| chemokine (C-X-C motif) ligand 1 | Cxcl1 | 1 | -1.60 | 3.46 | 1.01 | 1 | -1.81 | -2.33 | -2.76 | 1 | 31.25 | 1.02 | -1.27 | 1 | 42.87 | 1.21 | -1.23 | 1.16 | NM\_008176.1 |
| heme oxygenase (decycling) 1 | Hmox1 | 1 | 2.88 | 9.59 | 2.87 | 1 | 7.36 | 1.43 | -1.10 | 1 | 34.79 | 4.86 | 2.93 | 1 | 30.58 | 6.33 | 2.65 | -1.17 | NM\_010442.1 |
| heat shock protein 1 | Hspb1 | 1 | 28.36 | 30.78 | 30.49 | 1 | 1.59 | 1.59 | 1.12 | 1 | 23.37 | 24.84 | 9.48 | 1 | 1.24 | 1.33 | 1.26 | 19.76 | NM\_013560.1 |
| myeloid differentiation primary response gene 116 | Myd116 | 1 | 6.43 | 15.14 | 4.86 | 1 | 5.95 | 1.34 | 1.29 | 1 | 15.11 | 6.57 | 4.26 | 1 | 6.88 | 5.53 | 2.92 | -1.03 | NM\_008654.1 |
| interleukin 11 | Il11 | 1 | -1.06 | 14.85 | 1.10 | 1 | 4.37 | -1.17 | -2.40 | 1 | 6.01 | 1.12 | -1.24 | 1 | 3.42 | 1.02 | -1.47 | 1.52 | NM\_008350.1 |
| crystallin, alpha B | Cryab | 1 | 3.66 | 12.77 | 9.18 | 1 | 2.58 | 2.41 | 1.19 | 1 | 7.43 | 14.43 | 11.39 | 1 | 2.06 | 3.47 | 3.34 | 5.33 | NM\_009964.1 |
| prickle like 1 (Drosophila) | Prickle1 | 1 | 2.86 | 6.73 | 1.20 | 1 | 13.18 | -1.31 | -1.16 | 1 | 3.99 | 2.07 | 1.78 | 1 | 3.21 | 1.99 | 1.55 | -1.01 | XM\_147935.1 |
| proliferin | Plf | 1 | -1.50 | 3.08 | 11.89 | 1 | -1.43 | 2.24 | 1.01 | 1 | 1.10 | 5.18 | 10.94 | 1 | 1.66 | 3.75 | 3.03 | 2.42 | NM\_031191 |
| growth arrest and DNA-damage-inducible 45 alpha | Gadd45a | 1 | 3.67 | 4.76 | 1.48 | 1 | 4.38 | -1.24 | 1.29 | 1 | 6.20 | 6.24 | 3.43 | 1 | 10.53 | 8.32 | 2.92 | -1.48 | NM\_007836.1 |
| nuclear receptor subfamily 1, group D, member 2 | Nr1d2 | 1 | 1.89 | 3.08 | 1.02 | 1 | 7.06 | -1.13 | 1.73 | 1 | 9.84 | 3.97 | 2.89 | 1 | 7.05 | 2.15 | 1.27 | -1.55 | NM\_011584.2 |
| death effector domain-containing DNA binding protein 2 | Dedd2 | 1 | 1.05 | 3.22 | 1.59 | 1 | 5.82 | -1.09 | 1.38 | 1 | 5.40 | 8.64 | 6.69 | 1 | 3.84 | 5.26 | 2.62 | 1.32 | NM\_207677.1 |
| cytochrome P450, family 26, subfamily b, polypeptide 1 | Cyp26b1 | 1 | 1.83 | 3.81 | 1.97 | 1 | 2.22 | -1.24 | 1.87 | 1 | 8.37 | 1.11 | 1.46 | 1 | 8.41 | 1.07 | 1.41 | 1.17 | NM\_175475.2 |
| oxidative stress induced growth inhibitor 1 | Osgin1 | 1 | 1.05 | 5.10 | 2.27 | 1 | 2.77 | 1.09 | -1.35 | 1 | 7.94 | 6.28 | 2.59 | 1 | 8.35 | 5.69 | 1.71 | -1.06 | NM\_027950.1 |
| solute carrier family 23, member 3 | Slc23a3 | 1 | -1.23 | 8.35 | 3.34 | 1 | 2.96 | 1.19 | -1.05 | 1 | 4.66 | 1.68 | 1.68 | 1 | 1.83 | 1.36 | 1.20 | 1.03 | NM\_194333.2 |
| hyaluronan synthase1 | Has1 | 1 | 1.09 | 3.70 | -1.44 | 1 | 3.34 | -1.15 | -2.45 | 1 | 7.64 | 1.13 | -1.49 | 1 | 4.59 | 1.12 | -1.63 | 2.34 | NM\_008215.1 |
| zinc finger, AN1-type domain 2A | AA407930 | 1 | 1.21 | 4.49 | 2.36 | 1 | 4.01 | 1.42 | -1.18 | 1 | 7.55 | 5.10 | 5.14 | 1 | 5.84 | 4.02 | 2.97 | 1.33 | NM\_133349.2 |
| hyaluronidase 1 | Hyal1 | 1 | 1.83 | 3.16 | 2.67 | 1 | 2.33 | 1.22 | 1.34 | 1 | 1.91 | 3.14 | 6.35 | 1 | 1.52 | 1.87 | 2.52 | 1.08 | NM\_008317.2 |
| immediate early response 3 | Ier3 | 1 | -1.47 | 3.95 | 2.07 | 1 | 3.22 | 1.32 | 1.60 | 1 | 5.45 | 4.04 | 3.97 | 1 | 6.12 | 2.85 | 2.65 | 1.37 | NM\_133662.1 |
| ChaC, cation transport regulator-like 1 (E. coli) | Chac1 | 1 | 3.89 | 2.31 | 1.02 | 1 | 5.89 | -1.18 | 1.63 | 1 | 1.84 | 1.92 | 1.25 | 1 | 3.87 | 3.52 | 1.68 | -1.35 | NM\_026929.2 |
| DnaJ (Hsp40) homolog, subfamily B, member 1 | Dnajb1 | 1 | 5.20 | 5.16 | 1.55 | 1 | 2.45 | -1.87 | -1.50 | 1 | 5.85 | 2.08 | 1.41 | 1 | 2.91 | 1.49 | -1.04 | 2.08 | NM\_018808.1 |
| glial cell line derived neurotrophic factor | Gdnf | 1 | 1.13 | 3.80 | 1.15 | 1 | 1.27 | -1.33 | 1.05 | 1 | 5.66 | 2.18 | 1.91 | 1 | 2.75 | 1.67 | 2.06 | 2.05 | NM\_010275.1 |
| cDNA sequence BC031353 | BC031353 | 1 | 1.28 | 3.58 | 2.68 | 1 | 3.09 | 1.12 | 2.08 | 1 | 1.12 | 3.08 | 5.47 | 1 | 1.86 | 3.34 | 3.66 | 1.21 | NM\_153584.1 |
| Down syndrome critical region homolog 1 (human) | Dscr1 | 1 | 1.29 | 5.45 | 1.67 | 1 | 3.95 | -1.32 | -1.79 | 1 | 2.42 | 1.03 | -1.17 | 1 | 5.13 | 1.01 | -1.30 | 1.21 | NM\_019466.2 |
| AXIN1 up-regulated 1 | Axud1 | 1 | 1.02 | 4.59 | 1.32 | 1 | 5.40 | -1.28 | -1.25 | 1 | 3.16 | 1.35 | 1.38 | 1 | 1.67 | 1.42 | 1.20 | 1.30 | NM\_153287.2 |
| carbonyl reductase 3 | Cbr3 | 1 | -1.10 | 3.07 | 3.08 | 1 | 1.31 | 1.96 | -1.25 | 1 | 3.97 | 5.17 | 2.53 | 1 | 3.03 | 3.40 | 1.83 | 1.58 | NM\_173047.2 |
| tribbles homolog 3 (Drosophila) | Trib3 | 1 | 2.97 | 2.25 | 1.09 | 1 | 5.07 | 1.24 | 1.49 | 1 | 2.40 | 2.53 | 1.42 | 1 | 3.04 | 2.97 | 1.18 | -1.61 | NM\_175093.1 |
| semaF cytoplasmic domain associated protein 2 | Semcap2 | 1 | -1.12 | 4.69 | 2.77 | 1 | 1.70 | 1.10 | 1.53 | 1 | 1.60 | 1.70 | 3.30 | 1 | 1.24 | 2.23 | 3.33 | 2.64 | NM\_016867.1 |
| adenylosuccinate synthetase like 1 | Adssl1 | 1 | 1.28 | 2.46 | 4.57 | 1 | 2.02 | 1.66 | 2.69 | 1 | 1.09 | 1.48 | 3.33 | 1 | -1.09 | 1.40 | 3.58 | 1.30 | NM\_007421.1 |
| homocysteine-inducible, endoplasmic reticulum stress-inducible, ubiquitin-like domain member 1 | Herpud1 | 1 | 1.47 | -1.07 | -1.88 | 1 | 3.45 | -1.25 | 1.39 | 1 | 4.46 | 1.36 | -1.04 | 1 | 4.40 | 1.68 | -1.26 | -1.49 | NM\_022331.1 |
| F-box and WD-40 domain protein 5 | Fbxw5 | 1 | -4.69 | 1.10 | 1.10 | 1 | -2.07 | 2.39 | -8.59 | 1 | -1.53 | -1.09 | -5.07 | 1 | -3.97 | -3.57 | 1.13 | 1.93 | NM\_013908.1 |
| RIKEN cDNA 1200016B10 gene | 1200016B10Rik | 1 | 1.14 | 3.05 | 1.37 | 1 | 4.14 | -1.39 | -1.20 | 1 | 3.44 | 2.11 | 1.67 | 1 | 2.79 | 1.96 | 1.40 | 1.64 | NM\_025819.2 |
| CUB and Sushi multiple domains 3 | Csmd3 | 1 | 1.06 | 3.37 | 1.11 | 1 | 1.09 | -1.13 | -1.08 | 1 | 3.68 | 1.11 | -1.03 | 1 | 1.72 | 1.04 | -1.09 | -1.03 | XM\_139502.3 |
| enolase 2, gamma neuronal | Eno2 | 1 | 1.55 | 1.24 | 2.21 | 1 | 1.61 | 1.46 | 3.54 | 1 | -1.16 | 2.06 | 3.67 | 1 | 1.29 | 1.42 | 2.60 | -1.20 | NM\_013509.2 |
| sphingosine kinase 1 | Sphk1 | 1 | 1.10 | 3.27 | 1.04 | 1 | 3.62 | -1.23 | -1.26 | 1 | 2.55 | -1.20 | 1.87 | 1 | 3.38 | 1.09 | 2.06 | 1.43 | NM\_011451.1 |
| transformation related protein 53 inducible nuclear protein 1 | Trp53inp1 | 1 | 1.18 | 3.60 | 2.70 | 1 | 3.35 | -1.00 | 1.61 | 1 | 3.17 | 1.60 | 3.13 | 1 | 3.21 | 1.44 | 2.85 | 1.03 | NM\_021897.1 |
| transformed mouse 3T3 cell double minute 2 | Mdm2 | 1 | 1.06 | 3.56 | 2.18 | 1 | 3.56 | 1.24 | 1.42 | 1 | 3.12 | 1.48 | 1.63 | 1 | 2.27 | 1.45 | 1.26 | 1.00 | NM\_010786.2 |
| RIKEN cDNA E130102H24 gene (E130102H24Rik) | E130102H24Rik | 1 | -1.16 | 1.16 | 3.07 | 1 | 1.01 | 1.51 | 1.79 | 1 | -1.10 | 1.49 | 3.30 | 1 | -1.15 | 1.25 | 2.23 | -1.02 | XM\_149469.3 |
| cathepsin B | Ctsb | 1 | 1.24 | 3.10 | 1.38 | 1 | 1.47 | -1.29 | -1.21 | 1 | 2.10 | 2.03 | 3.08 | 1 | 1.65 | 1.49 | 1.75 | -1.01 | NM\_007798.1 |
| high mobility group box transcription factor 1 | Hbp1 | 1 | 1.62 | 1.86 | 3.06 | 1 | 1.63 | 1.46 | 2.76 | 1 | -1.27 | 1.66 | 3.07 | 1 | 1.02 | 1.71 | 2.79 | -1.26 | NM\_153198 |
| ChaC, cation transport regulator-like 1 (E. coli) | Chac1 | 1 | 3.89 | 2.31 | 1.02 | 1 | 5.89 | -1.18 | 1.63 | 1 | 1.84 | 1.92 | 1.25 | 1 | 3.87 | 3.52 | 1.68 | -1.35 | NM\_026929.2 |
| | | | | | | | | | | | | | | | | | | | |
| | | | | | | | | | | | | | | | | | | | |

## Slide 2
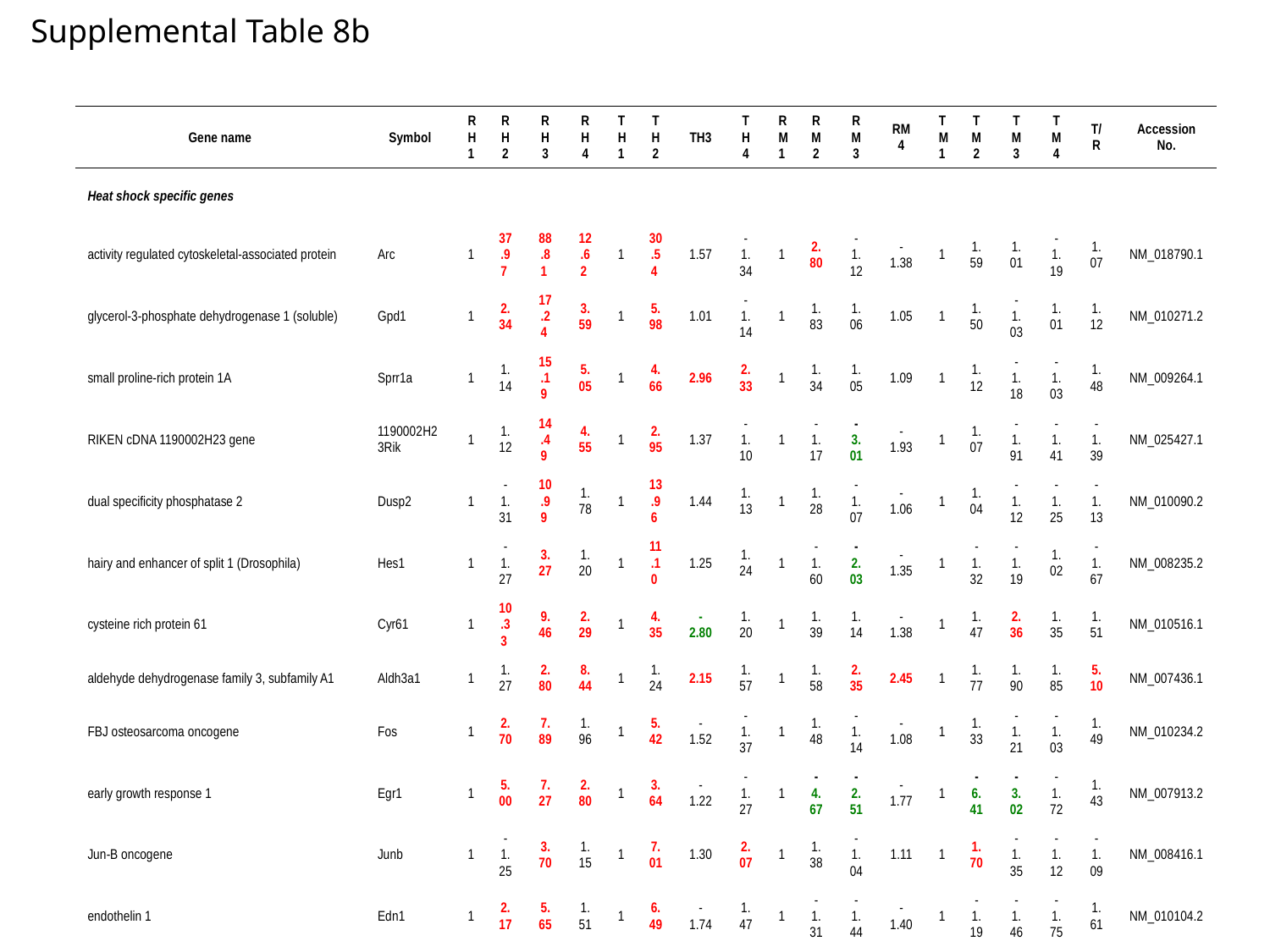

Supplemental Table 8b
| Gene name | Symbol | RH1 | RH2 | RH3 | RH4 | TH1 | TH2 | TH3 | TH4 | RM1 | RM2 | RM3 | RM4 | TM1 | TM2 | TM3 | TM4 | T/R | Accession No. |
| --- | --- | --- | --- | --- | --- | --- | --- | --- | --- | --- | --- | --- | --- | --- | --- | --- | --- | --- | --- |
| Heat shock specific genes | | | | | | | | | | | | | | | | | | | |
| activity regulated cytoskeletal-associated protein | Arc | 1 | 37.97 | 88.81 | 12.62 | 1 | 30.54 | 1.57 | -1.34 | 1 | 2.80 | -1.12 | -1.38 | 1 | 1.59 | 1.01 | -1.19 | 1.07 | NM\_018790.1 |
| glycerol-3-phosphate dehydrogenase 1 (soluble) | Gpd1 | 1 | 2.34 | 17.24 | 3.59 | 1 | 5.98 | 1.01 | -1.14 | 1 | 1.83 | 1.06 | 1.05 | 1 | 1.50 | -1.03 | 1.01 | 1.12 | NM\_010271.2 |
| small proline-rich protein 1A | Sprr1a | 1 | 1.14 | 15.19 | 5.05 | 1 | 4.66 | 2.96 | 2.33 | 1 | 1.34 | 1.05 | 1.09 | 1 | 1.12 | -1.18 | -1.03 | 1.48 | NM\_009264.1 |
| RIKEN cDNA 1190002H23 gene | 1190002H23Rik | 1 | 1.12 | 14.49 | 4.55 | 1 | 2.95 | 1.37 | -1.10 | 1 | -1.17 | -3.01 | -1.93 | 1 | 1.07 | -1.91 | -1.41 | -1.39 | NM\_025427.1 |
| dual specificity phosphatase 2 | Dusp2 | 1 | -1.31 | 10.99 | 1.78 | 1 | 13.96 | 1.44 | 1.13 | 1 | 1.28 | -1.07 | -1.06 | 1 | 1.04 | -1.12 | -1.25 | -1.13 | NM\_010090.2 |
| hairy and enhancer of split 1 (Drosophila) | Hes1 | 1 | -1.27 | 3.27 | 1.20 | 1 | 11.10 | 1.25 | 1.24 | 1 | -1.60 | -2.03 | -1.35 | 1 | -1.32 | -1.19 | 1.02 | -1.67 | NM\_008235.2 |
| cysteine rich protein 61 | Cyr61 | 1 | 10.33 | 9.46 | 2.29 | 1 | 4.35 | -2.80 | 1.20 | 1 | 1.39 | 1.14 | -1.38 | 1 | 1.47 | 2.36 | 1.35 | 1.51 | NM\_010516.1 |
| aldehyde dehydrogenase family 3, subfamily A1 | Aldh3a1 | 1 | 1.27 | 2.80 | 8.44 | 1 | 1.24 | 2.15 | 1.57 | 1 | 1.58 | 2.35 | 2.45 | 1 | 1.77 | 1.90 | 1.85 | 5.10 | NM\_007436.1 |
| FBJ osteosarcoma oncogene | Fos | 1 | 2.70 | 7.89 | 1.96 | 1 | 5.42 | -1.52 | -1.37 | 1 | 1.48 | -1.14 | -1.08 | 1 | 1.33 | -1.21 | -1.03 | 1.49 | NM\_010234.2 |
| early growth response 1 | Egr1 | 1 | 5.00 | 7.27 | 2.80 | 1 | 3.64 | -1.22 | -1.27 | 1 | -4.67 | -2.51 | -1.77 | 1 | -6.41 | -3.02 | -1.72 | 1.43 | NM\_007913.2 |
| Jun-B oncogene | Junb | 1 | -1.25 | 3.70 | 1.15 | 1 | 7.01 | 1.30 | 2.07 | 1 | 1.38 | -1.04 | 1.11 | 1 | 1.70 | -1.35 | -1.12 | -1.09 | NM\_008416.1 |
| endothelin 1 | Edn1 | 1 | 2.17 | 5.65 | 1.51 | 1 | 6.49 | -1.74 | 1.47 | 1 | -1.31 | -1.44 | -1.40 | 1 | -1.19 | -1.46 | -1.75 | 1.61 | NM\_010104.2 |
| transforming growth factor, beta 2 | Tgfb2 | 1 | 1.21 | 6.44 | 1.49 | 1 | 2.38 | -1.39 | -1.22 | 1 | 2.71 | 1.10 | 1.03 | 1 | 2.64 | 1.08 | 1.07 | 1.44 | NM\_009367.1 |
| Solute carrier family 11 (proton-coupled divalent metal ion transporters), member 1 | Slc11a1 | 1 | 6.02 | 5.08 | 1.60 | 1 | 2.23 | -1.20 | -1.13 | 1 | 1.04 | -1.12 | 1.23 | 1 | 1.20 | 1.16 | 1.07 | 1.33 | NM\_013612.1 |
| cysteine rich protein 61 | Cyr61 | 1 | 3.38 | 5.97 | 1.21 | 1 | 3.04 | -1.90 | -1.20 | 1 | 1.86 | 1.43 | 1.01 | 1 | 1.45 | 2.78 | 1.26 | 1.38 | NM\_010516.1 |
| feline sarcoma oncogene | Fes | 1 | -1.28 | 5.70 | 1.73 | 1 | 3.20 | 1.01 | -1.09 | 1 | 1.48 | 1.03 | -1.09 | 1 | 1.32 | 1.05 | 1.02 | -1.10 | NM\_010194.1 |
| forkhead box Q1 | Foxq1 | 1 | -1.08 | 5.38 | 1.07 | 1 | 3.43 | -1.03 | -1.10 | 1 | 1.35 | -1.62 | -1.37 | 1 | 1.37 | -1.17 | -1.02 | -2.18 | NM\_008239.3 |
| trans-golgi network protein | Tgoln1 | 1 | 1.83 | 5.28 | 2.08 | 1 | 2.14 | 1.19 | 1.22 | 1 | 1.80 | 1.97 | 2.95 | 1 | 1.54 | 1.45 | 1.68 | -1.02 | NM\_009443.2 |
| matrix metalloproteinase 3 | Mmp3 | 1 | -1.36 | -1.01 | 5.15 | 1 | -1.19 | 1.19 | 1.17 | 1 | -1.48 | -2.24 | -2.69 | 1 | 1.06 | -1.02 | -1.12 | -3.30 | NM\_010809.1 |
| FBJ osteosarcoma oncogene B | Fosb | 1 | 1.38 | 5.10 | 1.48 | 1 | 4.72 | -1.01 | -1.10 | 1 | -1.13 | 1.02 | 1.11 | 1 | -1.39 | -1.15 | -1.42 | 1.20 | NM\_008036 |
| AE binding protein 2 | Aebp2 | 1 | 2.06 | 4.80 | 2.58 | 1 | 1.73 | 1.05 | 1.12 | 1 | 2.10 | 1.16 | 1.63 | 1 | 1.22 | 1.04 | 1.10 | 1.24 | NM\_009637.1 |
| GRP1 (general receptor for phsphoinositides 1)-associated scaffold protein | Grasp | 1 | 1.24 | 4.67 | 2.47 | 1 | 2.75 | 2.13 | 1.25 | 1 | 1.51 | 1.32 | 1.59 | 1 | 1.20 | 1.03 | 1.10 | 1.23 | NM\_019518.2 |
| docking protein 3 | Dok3 | 1 | -1.18 | 4.66 | 1.54 | 1 | 2.24 | 1.01 | -1.67 | 1 | 2.27 | 1.12 | -1.03 | 1 | 1.93 | 1.02 | -1.27 | 1.28 | NM\_013739.1 |
| Clusterin | Clu | 1 | 2.07 | 4.62 | 2.96 | 1 | 2.30 | 1.72 | 1.38 | 1 | 1.31 | 1.37 | 1.76 | 1 | 1.22 | 1.39 | 1.61 | 1.69 | NM\_013492.1 |
| RIKEN cDNA 1700051I12 gene | 1700051I12Rik | 1 | 1.05 | 4.57 | 2.00 | 1 | 1.88 | 1.32 | 1.04 | 1 | 1.00 | -1.06 | 1.07 | 1 | -1.06 | -1.05 | 1.02 | -1.00 | XM\_181390.2 |
| GCIP-interacting protein p29 | Gcipip | 1 | 1.93 | 4.55 | 3.73 | 1 | 2.19 | 1.44 | 1.44 | 1 | -1.06 | 1.69 | 1.86 | 1 | -1.05 | 1.91 | 2.13 | 1.17 | NM\_026780.1 |
| H2A histone family, member J | H2afj | 1 | 1.34 | 4.43 | 1.59 | 1 | 2.20 | 1.19 | 1.10 | 1 | -1.08 | -1.12 | 1.10 | 1 | -1.22 | -1.17 | 1.11 | -1.18 | NM\_177688.2 |
| growth differentiation factor 1 | Gdf1 | 1 | 2.22 | 4.43 | 1.63 | 1 | 3.46 | 1.30 | 1.16 | 1 | 1.10 | -1.17 | -1.03 | 1 | -1.01 | -1.26 | -1.09 | -1.03 | NM\_008107 |
| growth arrest and DNA-damage-inducible 45 gamma | Gadd45g | 1 | 3.24 | 4.39 | 1.99 | 1 | 4.23 | -1.06 | 1.45 | 1 | 1.31 | 1.67 | 1.12 | 1 | 1.40 | 1.45 | 1.29 | 1.41 | NM\_011817.1 |
| preferentially expressed antigen in melanoma like 4 | Pramel4 | 1 | 4.37 | 3.83 | 1.91 | 1 | 1.75 | -1.16 | -1.26 | 1 | -1.15 | -1.09 | -1.06 | 1 | -1.06 | -1.12 | -1.14 | 1.60 | NM\_178248.2 |
| matrix metalloproteinase 3 | Mmp3 | 1 | -1.23 | 1.10 | 4.34 | 1 | -1.08 | 1.14 | 1.09 | 1 | -1.32 | -1.82 | -2.00 | 1 | 1.01 | -1.14 | -1.08 | -2.40 | NM\_010809.1 |
| purinergic receptor P2X, ligand-gated ion channel, 7 | P2rx7 | 1 | 1.16 | 4.33 | 4.34 | 1 | 2.32 | 1.18 | 1.24 | 1 | -1.02 | -1.13 | 1.16 | 1 | 1.01 | -1.07 | 1.16 | 1.09 | NM\_011027.1 |
| NUAK family, SNF1-like kinase, 2 | 1200013B22Rik | 1 | 1.02 | 2.91 | 1.30 | 1 | 4.28 | -1.11 | -1.33 | 1 | -1.74 | -1.02 | -1.25 | 1 | -1.10 | 1.27 | -1.16 | -1.22 | NM\_028778.2 |
| DNA-damage-inducible transcript 4-like | Ddit4l | 1 | -1.02 | 4.21 | 1.65 | 1 | 2.04 | -1.01 | 1.07 | 1 | 1.83 | 1.29 | 1.25 | 1 | 1.19 | 1.21 | 1.11 | -1.26 | NM\_030143.2 |
| nuclear receptor subfamily 1, group D, member 1 | Nr1d1 | 1 | 1.26 | 4.20 | 1.13 | 1 | 2.12 | -1.15 | 1.97 | 1 | -1.51 | 1.17 | 1.47 | 1 | -1.51 | 1.19 | 1.18 | 1.42 | NM\_145434.1 |
| tumor necrosis factor receptor superfamily, member 25 | Tnfrsf25 | 1 | -1.32 | 4.19 | 1.06 | 1 | 1.52 | -1.31 | -1.44 | 1 | 1.00 | -1.57 | -1.68 | 1 | 1.17 | -1.25 | -1.16 | -1.42 | NM\_033042 |
| RIKEN cDNA 2900001A12 gene | 2900001A12Rik | 1 | 2.30 | 3.89 | 1.29 | 1 | 2.09 | 1.00 | -1.14 | 1 | -1.00 | -1.08 | -1.03 | 1 | 1.19 | 1.03 | 1.25 | 1.16 | NM\_025971.1 |
| abhydrolase domain containing 2 | Abhd2 | 1 | 1.09 | 1.10 | 3.51 | 1 | 1.11 | 3.82 | 1.14 | 1 | 1.00 | 1.15 | -1.31 | 1 | -1.01 | 1.07 | -1.28 | 1.03 | NM\_018811.4 |
| purinergic receptor P2X, ligand-gated ion channel, 7 (P2rx7), transcript variant 4 | P2rx7 | 1 | 1.99 | 3.75 | 2.83 | 1 | 2.73 | 1.21 | -1.00 | 1 | 1.12 | -1.07 | 1.46 | 1 | -1.12 | -1.08 | 1.04 | -1.03 | NM\_001038887.1 |
| Centrosomal protein 97 | Cep97 | 1 | 1.03 | -1.14 | 3.70 | 1 | -1.04 | 1.43 | 1.48 | 1 | -1.27 | 1.20 | 1.43 | 1 | -1.03 | 1.14 | 1.36 | 1.04 | NM\_028815.3 |

## Slide 3
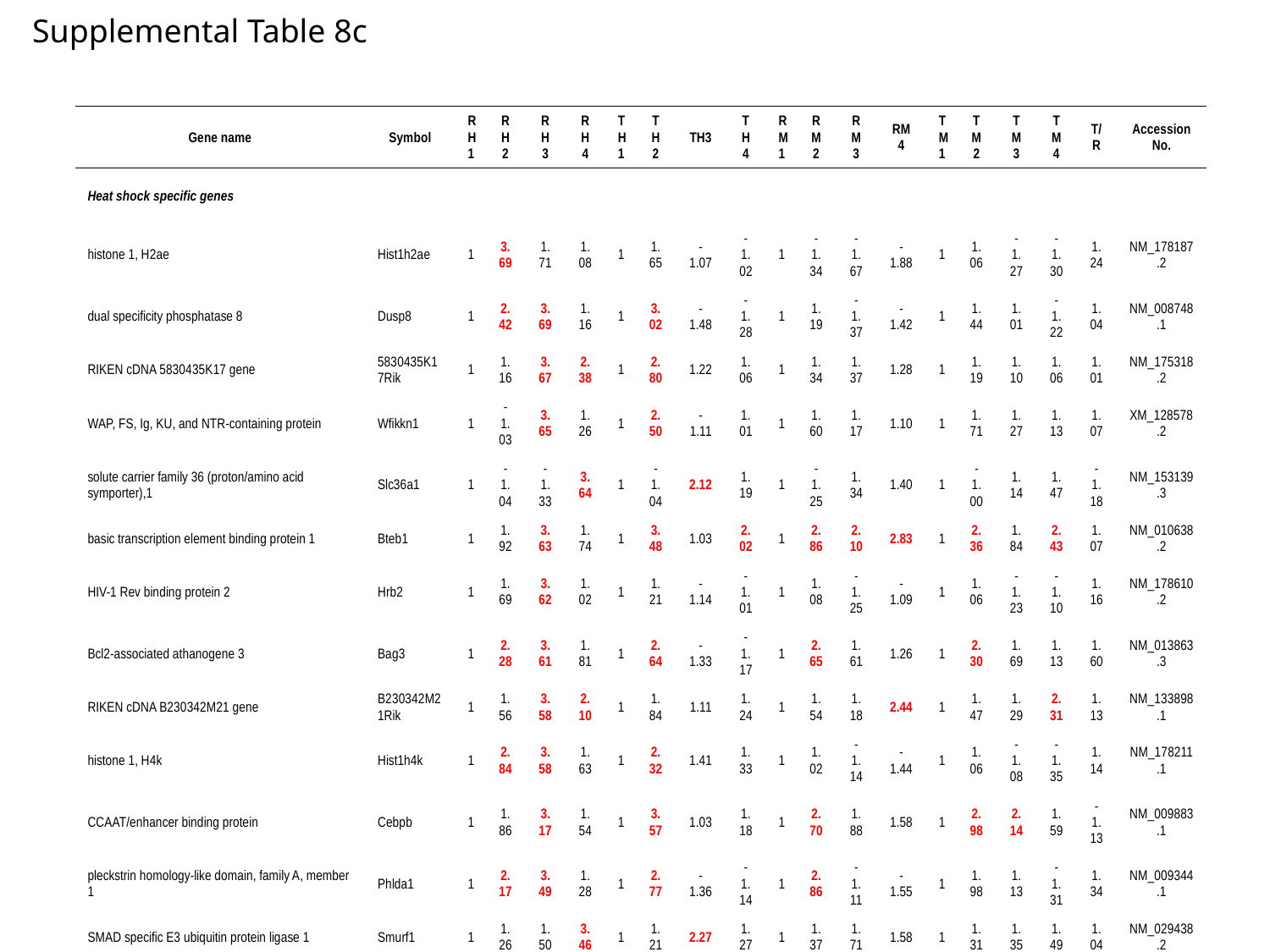

Supplemental Table 8c
| Gene name | Symbol | RH1 | RH2 | RH3 | RH4 | TH1 | TH2 | TH3 | TH4 | RM1 | RM2 | RM3 | RM4 | TM1 | TM2 | TM3 | TM4 | T/R | Accession No. |
| --- | --- | --- | --- | --- | --- | --- | --- | --- | --- | --- | --- | --- | --- | --- | --- | --- | --- | --- | --- |
| Heat shock specific genes | | | | | | | | | | | | | | | | | | | |
| histone 1, H2ae | Hist1h2ae | 1 | 3.69 | 1.71 | 1.08 | 1 | 1.65 | -1.07 | -1.02 | 1 | -1.34 | -1.67 | -1.88 | 1 | 1.06 | -1.27 | -1.30 | 1.24 | NM\_178187.2 |
| dual specificity phosphatase 8 | Dusp8 | 1 | 2.42 | 3.69 | 1.16 | 1 | 3.02 | -1.48 | -1.28 | 1 | 1.19 | -1.37 | -1.42 | 1 | 1.44 | 1.01 | -1.22 | 1.04 | NM\_008748.1 |
| RIKEN cDNA 5830435K17 gene | 5830435K17Rik | 1 | 1.16 | 3.67 | 2.38 | 1 | 2.80 | 1.22 | 1.06 | 1 | 1.34 | 1.37 | 1.28 | 1 | 1.19 | 1.10 | 1.06 | 1.01 | NM\_175318.2 |
| WAP, FS, Ig, KU, and NTR-containing protein | Wfikkn1 | 1 | -1.03 | 3.65 | 1.26 | 1 | 2.50 | -1.11 | 1.01 | 1 | 1.60 | 1.17 | 1.10 | 1 | 1.71 | 1.27 | 1.13 | 1.07 | XM\_128578.2 |
| solute carrier family 36 (proton/amino acid symporter),1 | Slc36a1 | 1 | -1.04 | -1.33 | 3.64 | 1 | -1.04 | 2.12 | 1.19 | 1 | -1.25 | 1.34 | 1.40 | 1 | -1.00 | 1.14 | 1.47 | -1.18 | NM\_153139.3 |
| basic transcription element binding protein 1 | Bteb1 | 1 | 1.92 | 3.63 | 1.74 | 1 | 3.48 | 1.03 | 2.02 | 1 | 2.86 | 2.10 | 2.83 | 1 | 2.36 | 1.84 | 2.43 | 1.07 | NM\_010638.2 |
| HIV-1 Rev binding protein 2 | Hrb2 | 1 | 1.69 | 3.62 | 1.02 | 1 | 1.21 | -1.14 | -1.01 | 1 | 1.08 | -1.25 | -1.09 | 1 | 1.06 | -1.23 | -1.10 | 1.16 | NM\_178610.2 |
| Bcl2-associated athanogene 3 | Bag3 | 1 | 2.28 | 3.61 | 1.81 | 1 | 2.64 | -1.33 | -1.17 | 1 | 2.65 | 1.61 | 1.26 | 1 | 2.30 | 1.69 | 1.13 | 1.60 | NM\_013863.3 |
| RIKEN cDNA B230342M21 gene | B230342M21Rik | 1 | 1.56 | 3.58 | 2.10 | 1 | 1.84 | 1.11 | 1.24 | 1 | 1.54 | 1.18 | 2.44 | 1 | 1.47 | 1.29 | 2.31 | 1.13 | NM\_133898.1 |
| histone 1, H4k | Hist1h4k | 1 | 2.84 | 3.58 | 1.63 | 1 | 2.32 | 1.41 | 1.33 | 1 | 1.02 | -1.14 | -1.44 | 1 | 1.06 | -1.08 | -1.35 | 1.14 | NM\_178211.1 |
| CCAAT/enhancer binding protein | Cebpb | 1 | 1.86 | 3.17 | 1.54 | 1 | 3.57 | 1.03 | 1.18 | 1 | 2.70 | 1.88 | 1.58 | 1 | 2.98 | 2.14 | 1.59 | -1.13 | NM\_009883.1 |
| pleckstrin homology-like domain, family A, member 1 | Phlda1 | 1 | 2.17 | 3.49 | 1.28 | 1 | 2.77 | -1.36 | -1.14 | 1 | 2.86 | -1.11 | -1.55 | 1 | 1.98 | 1.13 | -1.31 | 1.34 | NM\_009344.1 |
| SMAD specific E3 ubiquitin protein ligase 1 | Smurf1 | 1 | 1.26 | 1.50 | 3.46 | 1 | 1.21 | 2.27 | 1.27 | 1 | 1.37 | 1.71 | 1.58 | 1 | 1.31 | 1.35 | 1.49 | 1.04 | NM\_029438.2 |
| solute carrier family 19 (thiamine transporter), member 2 | Slc19a2 | 1 | -1.11 | 3.44 | -1.24 | 1 | 2.27 | -1.38 | -1.20 | 1 | 2.11 | 1.17 | 1.10 | 1 | 1.97 | 1.38 | 1.28 | 1.07 | NM\_054087.1 |
| creatine kinase, brain | Ckb | 1 | -1.02 | -1.06 | -1.90 | 1 | 3.43 | -1.25 | -1.18 | 1 | -1.00 | -2.19 | -1.64 | 1 | -1.08 | -1.11 | -1.06 | -46.07 | NM\_021273.2 |
| protease (prosome, macropain) 26S subunit, ATPase 1 | Psmc1 | 1 | -1.89 | 2.94 | -2.77 | 1 | 3.42 | 1.03 | 2.52 | 1 | 1.30 | 1.60 | 1.34 | 1 | 1.17 | 1.65 | 1.48 | -1.16 | NM\_008947 |
| TAF15 RNA polymerase II, TATA box binding protein (TBP)-associated factor | Taf15 | 1 | 1.87 | 3.40 | 1.10 | 1 | 2.04 | -1.30 | -1.13 | 1 | 1.54 | -1.06 | -1.18 | 1 | 1.57 | 1.40 | -1.13 | 1.16 | NM\_027427.1 |
| matrilin 4 | Matn4 | 1 | 1.85 | 3.40 | 1.36 | 1 | 2.03 | -1.14 | -1.44 | 1 | 1.20 | 1.15 | 1.20 | 1 | 1.30 | 1.38 | 1.15 | 2.48 | NM\_013592.2 |
| dystonin | Dst | 1 | 1.22 | 1.51 | 3.36 | 1 | 1.39 | 2.98 | 1.21 | 1 | -1.11 | -1.08 | -1.24 | 1 | -1.22 | -1.00 | -1.34 | 1.16 | NM\_010081.1 |
| potassium channel tetramerisation domain containing 4 | Kctd4 | 1 | 1.34 | 3.28 | 1.14 | 1 | 1.99 | -1.09 | -1.19 | 1 | -1.01 | 1.08 | 1.01 | 1 | 1.08 | 1.50 | 1.19 | -1.49 | NM\_026214.2 |
| transmembrane protein 80 | Tmem80 | 1 | 2.03 | 3.28 | 1.62 | 1 | 1.40 | 1.09 | -1.10 | 1 | -1.25 | -1.23 | -1.07 | 1 | -1.34 | -1.16 | 1.05 | 1.50 | NM\_027797.1 |
| dead end homolog 1 (zebrafish) | Dnd1 | 1 | 1.04 | 3.26 | 1.19 | 1 | 2.04 | 1.02 | -1.37 | 1 | 1.87 | 1.03 | -1.13 | 1 | 1.82 | 1.12 | -1.11 | 1.27 | NM\_173383.1 |
| C1q and tumor necrosis factor related protein 1 | C1qtnf1 | 1 | 1.07 | -1.00 | 3.25 | 1 | 1.21 | 1.28 | 1.85 | 1 | -1.54 | -2.02 | -1.32 | 1 | -1.42 | -2.41 | 1.01 | 2.04 | NM\_019959.1 |
| CD164 antigen | CD164 | 1 | -1.09 | -1.04 | -4.76 | 1 | -1.05 | -1.23 | -1.07 | 1 | 1.18 | 1.16 | 1.17 | 1 | 1.16 | 1.08 | 1.03 | -1.09 | NM\_016898.1 |
| nuclear receptor subfamily 1, group D, member 1 | Nr1d1 | 1 | 1.08 | 3.22 | 1.05 | 1 | 1.76 | -1.17 | 1.71 | 1 | -1.22 | 1.14 | 1.17 | 1 | -1.23 | 1.01 | 1.05 | 1.34 | NM\_145434.1 |
| Methyltransferase like 6 | Mettl6 | 1 | -1.14 | 3.15 | -1.04 | 1 | 2.04 | -1.30 | -1.36 | 1 | 2.44 | 1.73 | 1.21 | 1 | 2.02 | 1.57 | 1.07 | 1.36 | NM\_025907.2 |
| abhydrolase domain containing 2 | Abhd2 | 1 | 1.05 | 1.04 | 2.44 | 1 | 1.04 | 3.13 | 1.06 | 1 | 1.04 | 1.08 | -1.33 | 1 | 1.21 | 1.13 | -1.25 | 1.12 | NM\_018811.4 |
| zinc finger protein 95 | Zfp95 | 1 | 1.76 | 3.13 | 2.00 | 1 | 2.68 | 1.24 | 1.19 | 1 | -2.10 | 1.17 | 1.49 | 1 | -1.58 | 1.50 | 1.71 | 1.03 | XM\_355663.1 |
| SMAD specific E3 ubiquitin protein ligase 1 | Smurf1 | 1 | 1.21 | 1.53 | 3.11 | 1 | 1.36 | 2.19 | 1.38 | 1 | 1.35 | 1.59 | 1.43 | 1 | 1.12 | 1.35 | 1.38 | -1.07 | NM\_029438 |
| retinol dehydrogenase 5 | Rdh5 | 1 | -1.08 | 1.91 | 3.11 | 1 | 1.17 | 1.85 | 1.39 | 1 | 1.16 | -1.20 | -1.25 | 1 | 1.04 | -1.28 | -1.35 | 1.21 | NM\_134006.3 |
| F-box and leucine-rich repeat protein 20 | Fbxl20 | 1 | 1.74 | 1.25 | 3.02 | 1 | 1.63 | 2.19 | 2.14 | 1 | -1.76 | 1.08 | 1.98 | 1 | -1.67 | -1.32 | 1.76 | 1.33 | XM\_126674.3 |

## Slide 4
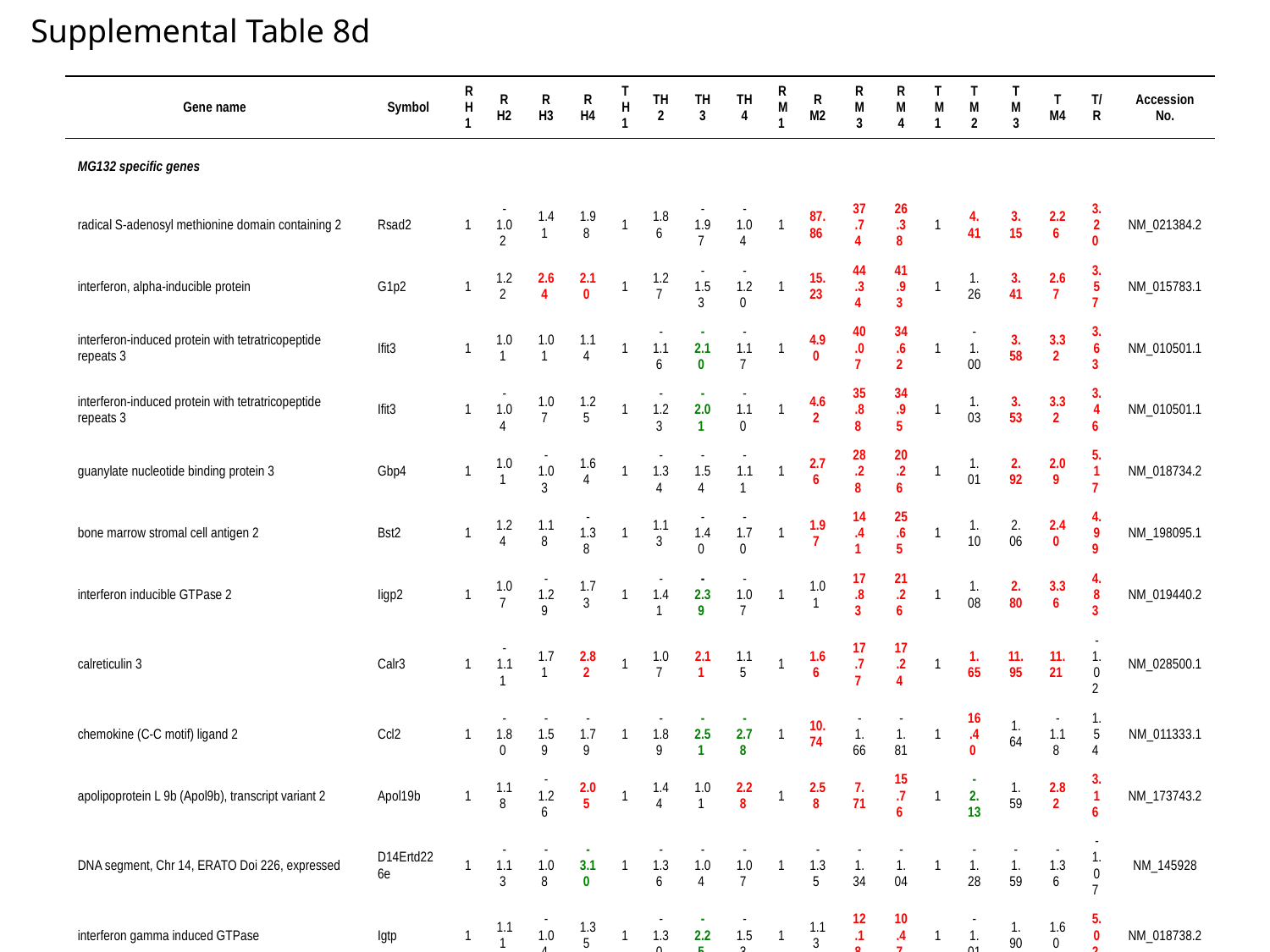

Supplemental Table 8d
| Gene name | Symbol | RH1 | RH2 | RH3 | RH4 | TH1 | TH2 | TH3 | TH4 | RM1 | RM2 | RM3 | RM4 | TM1 | TM2 | TM3 | TM4 | T/R | Accession No. |
| --- | --- | --- | --- | --- | --- | --- | --- | --- | --- | --- | --- | --- | --- | --- | --- | --- | --- | --- | --- |
| MG132 specific genes | | | | | | | | | | | | | | | | | | | |
| radical S-adenosyl methionine domain containing 2 | Rsad2 | 1 | -1.02 | 1.41 | 1.98 | 1 | 1.86 | -1.97 | -1.04 | 1 | 87.86 | 37.74 | 26.38 | 1 | 4.41 | 3.15 | 2.26 | 3.20 | NM\_021384.2 |
| interferon, alpha-inducible protein | G1p2 | 1 | 1.22 | 2.64 | 2.10 | 1 | 1.27 | -1.53 | -1.20 | 1 | 15.23 | 44.34 | 41.93 | 1 | 1.26 | 3.41 | 2.67 | 3.57 | NM\_015783.1 |
| interferon-induced protein with tetratricopeptide repeats 3 | Ifit3 | 1 | 1.01 | 1.01 | 1.14 | 1 | -1.16 | -2.10 | -1.17 | 1 | 4.90 | 40.07 | 34.62 | 1 | -1.00 | 3.58 | 3.32 | 3.63 | NM\_010501.1 |
| interferon-induced protein with tetratricopeptide repeats 3 | Ifit3 | 1 | -1.04 | 1.07 | 1.25 | 1 | -1.23 | -2.01 | -1.10 | 1 | 4.62 | 35.88 | 34.95 | 1 | 1.03 | 3.53 | 3.32 | 3.46 | NM\_010501.1 |
| guanylate nucleotide binding protein 3 | Gbp4 | 1 | 1.01 | -1.03 | 1.64 | 1 | -1.34 | -1.54 | -1.11 | 1 | 2.76 | 28.28 | 20.26 | 1 | 1.01 | 2.92 | 2.09 | 5.17 | NM\_018734.2 |
| bone marrow stromal cell antigen 2 | Bst2 | 1 | 1.24 | 1.18 | -1.38 | 1 | 1.13 | -1.40 | -1.70 | 1 | 1.97 | 14.41 | 25.65 | 1 | 1.10 | 2.06 | 2.40 | 4.99 | NM\_198095.1 |
| interferon inducible GTPase 2 | Iigp2 | 1 | 1.07 | -1.29 | 1.73 | 1 | -1.41 | -2.39 | -1.07 | 1 | 1.01 | 17.83 | 21.26 | 1 | 1.08 | 2.80 | 3.36 | 4.83 | NM\_019440.2 |
| calreticulin 3 | Calr3 | 1 | -1.11 | 1.71 | 2.82 | 1 | 1.07 | 2.11 | 1.15 | 1 | 1.66 | 17.77 | 17.24 | 1 | 1.65 | 11.95 | 11.21 | -1.02 | NM\_028500.1 |
| chemokine (C-C motif) ligand 2 | Ccl2 | 1 | -1.80 | -1.59 | -1.79 | 1 | -1.89 | -2.51 | -2.78 | 1 | 10.74 | -1.66 | -1.81 | 1 | 16.40 | 1.64 | -1.18 | 1.54 | NM\_011333.1 |
| apolipoprotein L 9b (Apol9b), transcript variant 2 | Apol19b | 1 | 1.18 | -1.26 | 2.05 | 1 | 1.44 | 1.01 | 2.28 | 1 | 2.58 | 7.71 | 15.76 | 1 | -2.13 | 1.59 | 2.82 | 3.16 | NM\_173743.2 |
| DNA segment, Chr 14, ERATO Doi 226, expressed | D14Ertd226e | 1 | -1.13 | -1.08 | -3.10 | 1 | -1.36 | -1.04 | -1.07 | 1 | -1.35 | -1.34 | -1.04 | 1 | -1.28 | -1.59 | -1.36 | -1.07 | NM\_145928 |
| interferon gamma induced GTPase | Igtp | 1 | 1.11 | -1.04 | 1.35 | 1 | -1.30 | -2.25 | -1.53 | 1 | 1.13 | 12.18 | 10.47 | 1 | -1.01 | 1.90 | 1.60 | 5.02 | NM\_018738.2 |
| hypothetical protein LOC223672 | LOC223672 | 1 | 1.10 | 1.17 | 2.08 | 1 | 1.39 | -1.02 | 1.78 | 1 | 3.03 | 8.28 | 11.83 | 1 | -2.05 | 1.73 | 2.34 | 3.00 | XM\_128064.4 |
| interferon dependent positive acting transcription factor 3 gamma | Isgf3g | 1 | -1.01 | 1.18 | -1.09 | 1 | -1.37 | -1.87 | -1.22 | 1 | 1.47 | 8.84 | 10.15 | 1 | 1.07 | 2.19 | 2.10 | 2.60 | NM\_008394.2 |
| guanylate nucleotide binding protein 1 | Gbp1 | 1 | 1.05 | -1.13 | 1.28 | 1 | -1.30 | -1.32 | -1.08 | 1 | 1.69 | 9.77 | 5.35 | 1 | -1.26 | 1.71 | 1.29 | 2.58 | NM\_010259.1 |
| interferon regulatory factor 7 | Irf7 | 1 | 1.02 | -1.05 | 1.02 | 1 | -1.10 | -1.40 | 1.00 | 1 | -1.08 | 5.59 | 9.61 | 1 | -1.06 | 2.17 | 2.44 | 1.57 | NM\_016850.1 |
| chemokine (C-C motif) ligand 7 | Ccl7 | 1 | -1.90 | -1.38 | -1.16 | 1 | -1.97 | -1.70 | -1.81 | 1 | 9.36 | -2.86 | -1.56 | 1 | 8.76 | -1.61 | -1.38 | 2.37 | NM\_013654 |
| solute carrier family 7 (cationic amino acid transporter, y+ system), member 11 | Slc7a11 | 1 | 1.25 | 1.27 | 1.34 | 1 | 1.33 | -1.04 | -1.58 | 1 | 6.89 | 4.77 | 1.80 | 1 | 9.32 | 6.25 | 1.66 | 1.25 | NM\_011990.1 |
| signal transducer and activator of transcription 2 | Stat2 | 1 | -1.03 | -1.31 | 1.16 | 1 | -1.19 | -1.56 | 1.15 | 1 | 2.90 | 6.17 | 8.75 | 1 | 1.25 | 1.92 | 2.14 | 1.56 | NM\_019963.1 |
| angiopoietin-like 6 | Angptl6 | 1 | 1.82 | 1.29 | -1.11 | 1 | 1.17 | -1.06 | -1.04 | 1 | 2.67 | 6.52 | 8.73 | 1 | 1.86 | 6.52 | 5.61 | -1.20 | NM\_145154 |
| toll-like receptor 2 | Tlr2 | 1 | 1.38 | -1.72 | 1.03 | 1 | -1.06 | -1.80 | -1.35 | | 5.74 | 2.63 | 2.12 | 1 | 8.73 | 3.04 | 1.57 | 1.91 | NM\_011905.2 |
| lectin, galactoside-binding, soluble, 3 binding protein | Lgals3bp | 1 | 1.14 | -1.92 | -1.01 | 1 | 1.06 | -1.13 | 1.29 | 1 | -1.07 | 4.35 | 8.21 | 1 | -1.13 | 1.57 | 2.49 | 2.27 | NM\_011150.1 |
| DNA-damage inducible transcript 3 | Ddit3 | 1 | 1.90 | 2.00 | 1.88 | 1 | 2.74 | 1.13 | 1.48 | 1 | 7.48 | 7.71 | 7.85 | 1 | 7.99 | 6.32 | 3.29 | -1.24 | NM\_007837.2 |
| biliverdin reductase B (flavin reductase (NADPH)) | Blvrb | 1 | -1.04 | 1.29 | 1.42 | 1 | 1.21 | 1.08 | -1.00 | 1 | 1.65 | 6.22 | 7.06 | 1 | 1.92 | 6.87 | 7.62 | 1.49 | NM\_144923 |
| leukemia inhibitory factor (Lif), transcript variant 2 | Lif | 1 | -1.14 | 2.17 | -1.26 | 1 | 1.82 | -1.97 | -2.07 | 1 | 4.74 | -1.96 | -2.00 | 1 | 7.41 | -1.46 | -1.34 | 1.06 | NM\_001039537.1 |
| abhydrolase domain containing 4 | Abhd4 | 1 | 1.20 | 1.82 | 2.28 | 1 | 1.72 | 1.58 | 1.46 | 1 | 1.31 | 5.69 | 7.27 | 1 | 1.64 | 5.83 | 7.23 | -1.03 | NM\_134076.1 |
| glutamate-cysteine ligase , modifier subunit | Gclm | 1 | -1.19 | 1.34 | -1.05 | 1 | 1.27 | 1.20 | -1.57 | 1 | 6.24 | 6.80 | 3.70 | 1 | 5.70 | 7.19 | 2.62 | -1.32 | NM\_008129.2 |
| amphiregulin | Areg | 1 | -1.23 | 1.58 | -1.95 | 1 | 2.73 | 1.12 | -1.32 | 1 | 3.96 | -1.67 | -3.28 | 1 | 7.09 | -1.07 | -2.48 | -4.00 | NM\_009704.2 |
| interferon inducible protein 1 | Ifi1 | 1 | -1.17 | -1.32 | 1.31 | 1 | -1.29 | -1.79 | -1.33 | 1 | 1.88 | 6.79 | 6.85 | 1 | -1.26 | 1.61 | 1.67 | 2.13 | NM\_008326.1 |
| poly (ADP-ribose) polymerase family, member 14 | Parp14 | | 1.05 | -1.80 | -1.58 | | -1.58 | -1.89 | -1.12 | | 2.76 | 6.79 | 5.36 | | 1.32 | 1.45 | 1.72 | 2.00 | NM\_145481.1 |
| Ras-related GTP binding D | Rragd | 1 | 1.05 | 1.53 | 2.90 | 1 | 1.04 | -1.11 | -1.12 | 1 | 1.42 | 1.93 | 6.68 | 1 | 1.08 | 1.13 | 2.07 | -1.43 | NM\_027491 |
| EST AA175286 | AA175286 | 1 | -1.05 | -1.48 | -1.15 | 1 | -1.20 | -1.65 | -1.17 | 1 | 1.25 | 5.58 | 6.59 | 1 | -1.77 | 1.86 | 2.29 | 1.58 | NM\_010156.2 |
| signal transducer and activator of transcription 1 | Stat1 | 1 | -1.18 | -1.33 | -1.39 | 1 | -1.48 | -1.50 | -1.36 | 1 | -1.40 | 6.44 | 4.13 | 1 | -1.27 | 1.78 | 1.37 | 1.68 | NM\_009283.2 |
| RIKEN cDNA 1200015N20 gene | 1200015N20Rik | 1 | 1.18 | 2.61 | -1.26 | 1 | 2.42 | -1.90 | -1.73 | 1 | 4.41 | 1.50 | 1.08 | 1 | 6.37 | 1.73 | 1.15 | -1.52 | NM\_024244.3 |
| signal transducer and activator of transcription 1 | Stat1 | 1 | -1.11 | -1.22 | 1.01 | 1 | -1.23 | -1.22 | -1.06 | 1 | -1.31 | 6.29 | 3.58 | 1 | -1.38 | 1.79 | 1.33 | 1.61 | NM\_009283.2 |
| sulfiredoxin 1 homolog (S. cerevisiae) | Npn3 | 1 | -1.17 | -1.20 | -1.29 | 1 | 1.30 | 1.04 | -1.53 | 1 | 6.28 | 4.75 | 1.88 | 1 | 5.93 | 5.44 | 2.23 | -1.03 | NM\_029688.2 |
| cDNA sequence BC023741 | BC023741 | 1 | 1.10 | -1.34 | 1.02 | 1 | -1.14 | -1.39 | 1.23 | 1 | 1.17 | 4.89 | 6.26 | 1 | -1.45 | 1.26 | 1.58 | 1.79 | XM\_148582.3 |
| sequestosome 1 | Sqstm1 | 1 | 1.38 | 2.70 | 2.06 | 1 | 2.61 | 1.46 | 1.36 | 1 | 6.23 | 5.67 | 4.76 | 1 | 6.17 | 5.59 | 4.34 | -1.13 | NM\_011018.1 |
| programmed cell death 1 ligand 1 | Pdcd1lg1 | 1 | 1.16 | 1.56 | 1.28 | 1 | 1.01 | -1.13 | -1.14 | 1 | 5.89 | 6.15 | 4.30 | 1 | 1.45 | 1.73 | 1.34 | 2.03 | NM\_021893.2 |
| DEAD (Asp-Glu-Ala-Asp) box polypeptide 58 | Ddx58 | 1 | -1.10 | -1.42 | -1.08 | 1 | -1.01 | -1.17 | 1.14 | 1 | 1.47 | 5.51 | 6.12 | 1 | -1.04 | 1.78 | 1.80 | 1.73 | NM\_172689.2 |
| GTP cyclohydrolase 1 | Gch1 | 1 | 1.05 | 1.14 | -1.11 | 1 | 1.35 | -1.40 | -1.14 | 1 | 2.79 | 1.11 | 1.18 | 1 | 3.58 | 1.95 | 1.21 | 1.47 | NM\_008102.2 |
| nerve growth factor, beta | Ngfb | 1 | -1.30 | 1.43 | -1.35 | 1 | 1.37 | 1.14 | -1.81 | 1 | 5.86 | 3.08 | 1.09 | 1 | 2.61 | 2.25 | -1.60 | 1.20 | NM\_013609.1 |
| cDNA sequence BC022687 | BC022687 | 1 | 1.41 | 1.50 | 2.24 | 1 | 1.77 | 1.77 | 1.28 | 1 | 1.75 | 4.45 | 5.81 | 1 | 1.23 | 3.50 | 3.95 | -1.11 | NM\_145450.2 |
| RIKEN cDNA 9830147J24 gene | 9830147J24Rik | 1 | 1.04 | -1.01 | 1.18 | 1 | -1.02 | -1.33 | 1.05 | 1 | 2.49 | 5.73 | 4.17 | 1 | 1.07 | 1.26 | 1.16 | 2.15 | NM\_145545.2 |

## Slide 5
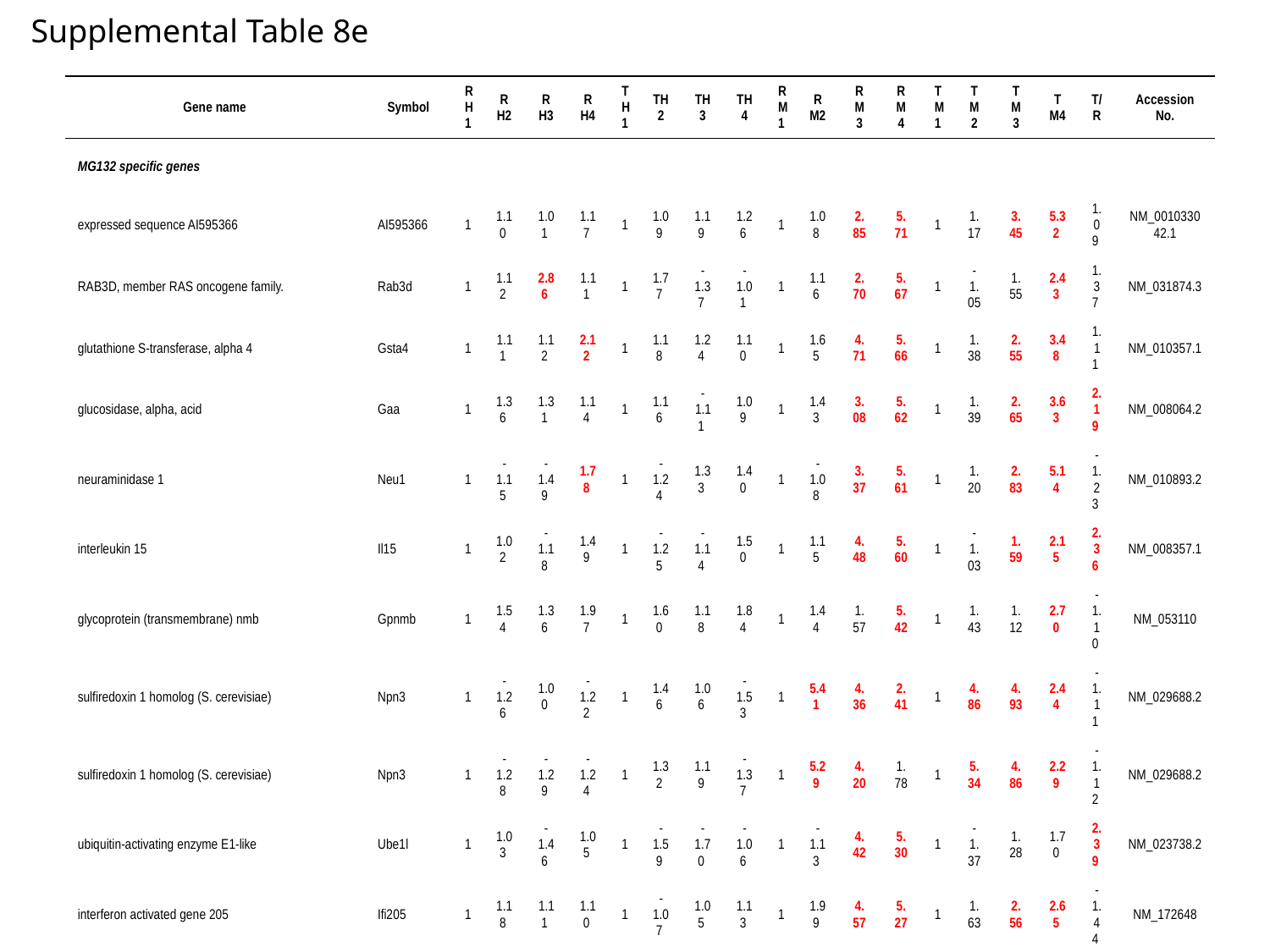

Supplemental Table 8e
| Gene name | Symbol | RH1 | RH2 | RH3 | RH4 | TH1 | TH2 | TH3 | TH4 | RM1 | RM2 | RM3 | RM4 | TM1 | TM2 | TM3 | TM4 | T/R | Accession No. |
| --- | --- | --- | --- | --- | --- | --- | --- | --- | --- | --- | --- | --- | --- | --- | --- | --- | --- | --- | --- |
| MG132 specific genes | | | | | | | | | | | | | | | | | | | |
| expressed sequence AI595366 | AI595366 | 1 | 1.10 | 1.01 | 1.17 | 1 | 1.09 | 1.19 | 1.26 | 1 | 1.08 | 2.85 | 5.71 | 1 | 1.17 | 3.45 | 5.32 | 1.09 | NM\_001033042.1 |
| RAB3D, member RAS oncogene family. | Rab3d | 1 | 1.12 | 2.86 | 1.11 | 1 | 1.77 | -1.37 | -1.01 | 1 | 1.16 | 2.70 | 5.67 | 1 | -1.05 | 1.55 | 2.43 | 1.37 | NM\_031874.3 |
| glutathione S-transferase, alpha 4 | Gsta4 | 1 | 1.11 | 1.12 | 2.12 | 1 | 1.18 | 1.24 | 1.10 | 1 | 1.65 | 4.71 | 5.66 | 1 | 1.38 | 2.55 | 3.48 | 1.11 | NM\_010357.1 |
| glucosidase, alpha, acid | Gaa | 1 | 1.36 | 1.31 | 1.14 | 1 | 1.16 | -1.11 | 1.09 | 1 | 1.43 | 3.08 | 5.62 | 1 | 1.39 | 2.65 | 3.63 | 2.19 | NM\_008064.2 |
| neuraminidase 1 | Neu1 | 1 | -1.15 | -1.49 | 1.78 | 1 | -1.24 | 1.33 | 1.40 | 1 | -1.08 | 3.37 | 5.61 | 1 | 1.20 | 2.83 | 5.14 | -1.23 | NM\_010893.2 |
| interleukin 15 | Il15 | 1 | 1.02 | -1.18 | 1.49 | 1 | -1.25 | -1.14 | 1.50 | 1 | 1.15 | 4.48 | 5.60 | 1 | -1.03 | 1.59 | 2.15 | 2.36 | NM\_008357.1 |
| glycoprotein (transmembrane) nmb | Gpnmb | 1 | 1.54 | 1.36 | 1.97 | 1 | 1.60 | 1.18 | 1.84 | 1 | 1.44 | 1.57 | 5.42 | 1 | 1.43 | 1.12 | 2.70 | -1.10 | NM\_053110 |
| sulfiredoxin 1 homolog (S. cerevisiae) | Npn3 | 1 | -1.26 | 1.00 | -1.22 | 1 | 1.46 | 1.06 | -1.53 | 1 | 5.41 | 4.36 | 2.41 | 1 | 4.86 | 4.93 | 2.44 | -1.11 | NM\_029688.2 |
| sulfiredoxin 1 homolog (S. cerevisiae) | Npn3 | 1 | -1.28 | -1.29 | -1.24 | 1 | 1.32 | 1.19 | -1.37 | 1 | 5.29 | 4.20 | 1.78 | 1 | 5.34 | 4.86 | 2.29 | -1.12 | NM\_029688.2 |
| ubiquitin-activating enzyme E1-like | Ube1l | 1 | 1.03 | -1.46 | 1.05 | 1 | -1.59 | -1.70 | -1.06 | 1 | -1.13 | 4.42 | 5.30 | 1 | -1.37 | 1.28 | 1.70 | 2.39 | NM\_023738.2 |
| interferon activated gene 205 | Ifi205 | 1 | 1.18 | 1.11 | 1.10 | 1 | -1.07 | 1.05 | 1.13 | 1 | 1.99 | 4.57 | 5.27 | 1 | 1.63 | 2.56 | 2.65 | -1.44 | NM\_172648 |
| sorbitol dehydrogenase 1 | Sdh1 | 1 | 1.43 | 1.39 | 1.23 | 1 | 1.57 | -1.19 | 1.62 | 1 | -1.02 | 2.66 | 5.21 | 1 | 1.13 | 2.39 | 3.71 | 1.54 | NM\_146126.1 |
| molybdenum cofactor synthesis 1 | Mocs1 | 1 | -1.06 | 1.35 | 1.63 | 1 | -1.37 | 1.45 | -1.20 | 1 | 1.24 | 5.15 | 2.81 | 1 | 1.38 | 4.57 | 2.58 | -1.60 | NM\_020042.1 |
| component of Sp100-rs | Csprs | 1 | -1.24 | -1.14 | 1.09 | 1 | -1.37 | 2.09 | 2.32 | 1 | -1.78 | 3.77 | 5.12 | 1 | -2.61 | 1.13 | 1.50 | 5.19 | NM\_033616.2 |
| protein kinase, interferon-inducible double stranded RNA dependent | Prkr | 1 | -1.08 | -1.07 | 1.41 | 1 | -1.09 | -1.01 | -1.17 | 1 | -1.67 | 5.05 | 4.82 | 1 | -1.33 | 2.09 | 1.93 | -1.13 | NM\_011163.2 |
| poly (ADP-ribose) polymerase family, member 9 | Parp9 | 1 | 1.07 | -1.22 | -1.06 | 1 | -1.15 | -1.59 | -1.07 | 1 | -1.18 | 4.84 | 5.02 | 1 | -1.17 | 1.91 | 2.39 | 1.81 | NM\_030253.1 |
| Prolyl endopeptidase-like | Prepl | 1 | -1.11 | -1.48 | -1.75 | 1 | -1.40 | -1.87 | -1.52 | 1 | 1.99 | 4.97 | 3.27 | 1 | 1.84 | 4.45 | 2.78 | -1.22 | NM\_145984.2 |
| integrin beta 7 | Itgb7 | 1 | 1.05 | -1.07 | -1.52 | 1 | 1.20 | -1.31 | 1.06 | 1 | 1.18 | 2.21 | 4.96 | 1 | 1.40 | 1.74 | 3.11 | -1.07 | NM\_013566.1 |
| lectin, galactose binding, soluble 9 | Lgals9 | 1 | 1.13 | -1.04 | -1.02 | 1 | -1.02 | -1.50 | -1.30 | 1 | -1.07 | 4.35 | 4.94 | 1 | -1.03 | 1.70 | 2.17 | 2.10 | NM\_010708.1 |
| RIKEN cDNA 1700012H17 gene | 1700012H17Rik | 1 | -1.03 | 2.11 | 2.03 | 1 | 1.98 | 1.23 | 1.19 | 1 | 1.86 | 2.39 | 4.92 | 1 | -1.10 | 1.50 | 2.30 | 1.51 | NM\_173426.1 |
| thrombomodulin | Thbd | 1 | 1.31 | 1.27 | -1.05 | 1 | 1.04 | -1.29 | -1.21 | 1 | 4.90 | 1.20 | 1.14 | 1 | 4.52 | 1.81 | 1.70 | 1.36 | NM\_009378.1 |
| polo-like kinase 3 (Drosophila) | Plk3 | 1 | 1.12 | 1.56 | 1.24 | 1 | 1.95 | 1.09 | -1.18 | 1 | 4.25 | 4.89 | 4.01 | 1 | 4.13 | 3.73 | 1.88 | -1.09 | NM\_013807.1 |
| caspase recruitment domain 4 | Card4 | 1 | 1.43 | 1.20 | 2.58 | 1 | 1.73 | 1.13 | 1.98 | 1 | 1.07 | 1.67 | 4.85 | 1 | 1.26 | 1.82 | 4.18 | -1.01 | NM\_172729.1 |
| StAR-related lipid transfer (START) domain containing 5 | Stard5 | 1 | 1.17 | -1.33 | -1.06 | 1 | -1.13 | -1.34 | -1.38 | 1 | 4.82 | 1.82 | 1.26 | 1 | 2.73 | 1.35 | 1.06 | 1.26 | NM\_023377.4 |
| interferon dependent positive acting transcription factor 3 gamma | Isgf3g | 1 | 1.01 | 1.03 | 1.05 | 1 | -1.06 | -1.46 | -1.05 | 1 | 1.11 | 4.68 | 4.80 | 1 | 1.02 | 1.61 | 1.53 | 2.10 | NM\_008394.2 |
| DNA segment, Chr 5, ERATO Doi 593, expressed | D5Ertd593e | 1 | 1.38 | 1.18 | -1.34 | 1 | 1.07 | -1.93 | -1.34 | 1 | 4.79 | 2.92 | 2.11 | 1 | 3.69 | 2.64 | 1.61 | 1.33 | NM\_175096.2 |
| N-myc downstream regulated gene | Ndrg1 | 1 | 1.61 | 1.54 | -2.14 | 1 | 1.18 | -1.47 | -1.20 | 1 | 3.42 | 2.81 | 4.09 | 1 | 4.78 | 3.21 | 4.14 | 1.09 | NM\_010884.1 |
| annexin A8 | Anxa8 | 1 | 1.00 | 1.17 | -1.24 | 1 | 1.17 | -1.00 | 1.05 | 1 | 1.25 | 2.61 | 4.76 | 1 | -1.03 | 1.76 | 3.92 | 1.12 | NM\_013473.2 |
| nitric oxide synthase 3 antisense | Nos3as | 1 | 1.20 | 2.07 | 1.75 | 1 | 1.90 | 1.61 | 1.59 | 1 | -1.01 | 1.93 | 4.75 | 1 | -1.13 | 2.00 | 2.83 | -1.06 | NM\_001002897.2 |
| ubiquitin-conjugating enzyme E2L 6 | Ube2l6 | 1 | 1.14 | -1.02 | 1.05 | 1 | 1.23 | -1.10 | 1.31 | 1 | -1.09 | 3.26 | 4.73 | 1 | -1.02 | 1.47 | 1.79 | 1.39 | NM\_019949 |
| insulin induced gene 1 | Insig1 | 1 | 1.01 | -1.32 | -1.82 | 1 | 1.18 | -1.67 | 1.01 | 1 | 4.72 | -1.05 | -1.03 | 1 | 3.16 | -1.24 | 1.32 | 1.44 | NM\_153526.2 |
| glycoprotein (transmembrane) nmb | Gpnmb | 1 | 1.26 | 1.30 | 1.88 | 1 | 1.67 | 1.07 | 1.62 | 1 | 1.69 | 1.42 | 4.71 | 1 | 1.70 | 1.20 | 2.38 | -1.09 | NM\_053110.2 |
| zinc finger protein 36 | Zfp36 | 1 | -1.38 | 1.74 | 1.50 | 1 | 1.73 | -1.33 | 1.01 | 1 | 4.70 | 2.37 | 1.86 | 1 | 2.80 | 1.87 | 1.33 | 1.26 | NM\_011756.3 |
| cathepsin D | Ctsd | 1 | 1.27 | 1.70 | 1.54 | 1 | 1.38 | 1.14 | 1.27 | 1 | 1.32 | 2.80 | 4.70 | 1 | 1.22 | 2.13 | 3.38 | 1.06 | NM\_009983.2 |
| interferon activated gene 203 | Ifi203 | 1 | 1.24 | 1.15 | 1.12 | 1 | 1.16 | 1.11 | 1.32 | 1 | 1.93 | 3.78 | 4.63 | 1 | 1.28 | 1.71 | 1.67 | -1.53 | NM\_008328.1 |
| N-myc downstream regulated-like | Ndrl | 1 | 1.68 | 1.59 | 1.13 | 1 | 1.29 | -1.20 | -1.05 | 1 | 3.52 | 3.11 | 4.46 | 1 | 4.53 | 3.24 | 4.23 | -1.05 | NM\_008681 |
| StAR-related lipid transfer (START) domain containing 5 | Stard5 | 1 | 1.18 | -1.12 | -1.02 | 1 | -1.26 | -1.23 | 1.06 | 1 | 4.50 | 2.06 | 1.65 | 1 | 2.84 | 1.60 | 1.25 | 1.08 | NM\_023377.4 |
| solute carrier family 16 (monocarboxylic acid transporters), member 9 | Slc16a9 | 1 | 1.09 | 2.84 | -1.09 | 1 | 1.01 | -1.03 | 1.02 | 1 | 4.35 | 1.09 | 1.10 | 1 | 1.42 | 1.08 | 1.10 | -1.27 | NM\_025807.1 |
| SH3-domain binding protein 2 | Sh3bp2 | 1 | -1.22 | 1.37 | 1.19 | 1 | 1.64 | 1.09 | -1.48 | 1 | 2.11 | 4.08 | 2.70 | 1 | 2.09 | 4.33 | 1.97 | 1.13 | NM\_011893 |
| zinc finger CCCH type domain containing 1 | Zc3hdc1 | 1 | -1.14 | -1.46 | -1.36 | 1 | -1.32 | -1.33 | -1.16 | 1 | -1.05 | 4.30 | 3.88 | 1 | -1.00 | 1.75 | 1.61 | 1.30 | NM\_172893.1 |
| heat shock protein 105 | Hsp105 | 1 | 1.10 | 2.23 | -2.11 | 1 | 1.87 | -1.76 | -1.36 | 1 | 4.25 | 2.40 | 1.64 | 1 | 1.55 | 1.34 | -1.16 | 2.03 | NM\_013559.1 |
| ubiquitin-conjugating enzyme E2L 6 | Ube2l6 | 1 | 1.05 | -1.05 | 1.20 | 1 | 1.01 | -1.21 | 1.26 | 1 | -1.20 | 3.36 | 4.23 | 1 | 1.00 | 1.45 | 2.00 | 1.79 | NM\_019949.1 |
| LIM and senescent cell antigen like domains 2 | Lims2 | 1 | 1.27 | 2.64 | 1.38 | 1 | 1.07 | -2.18 | -1.58 | 1 | 1.34 | 1.96 | 4.21 | 1 | 1.16 | 1.13 | 1.41 | 1.39 | NM\_144862.1 |
| tripartite motif protein 25 | Trim25 | 1 | -1.24 | -1.08 | -1.55 | 1 | -1.26 | -1.40 | 1.13 | 1 | 1.68 | 3.52 | 4.21 | 1 | -1.15 | 1.25 | 1.46 | 1.10 | XM\_126545.2 |

## Slide 6
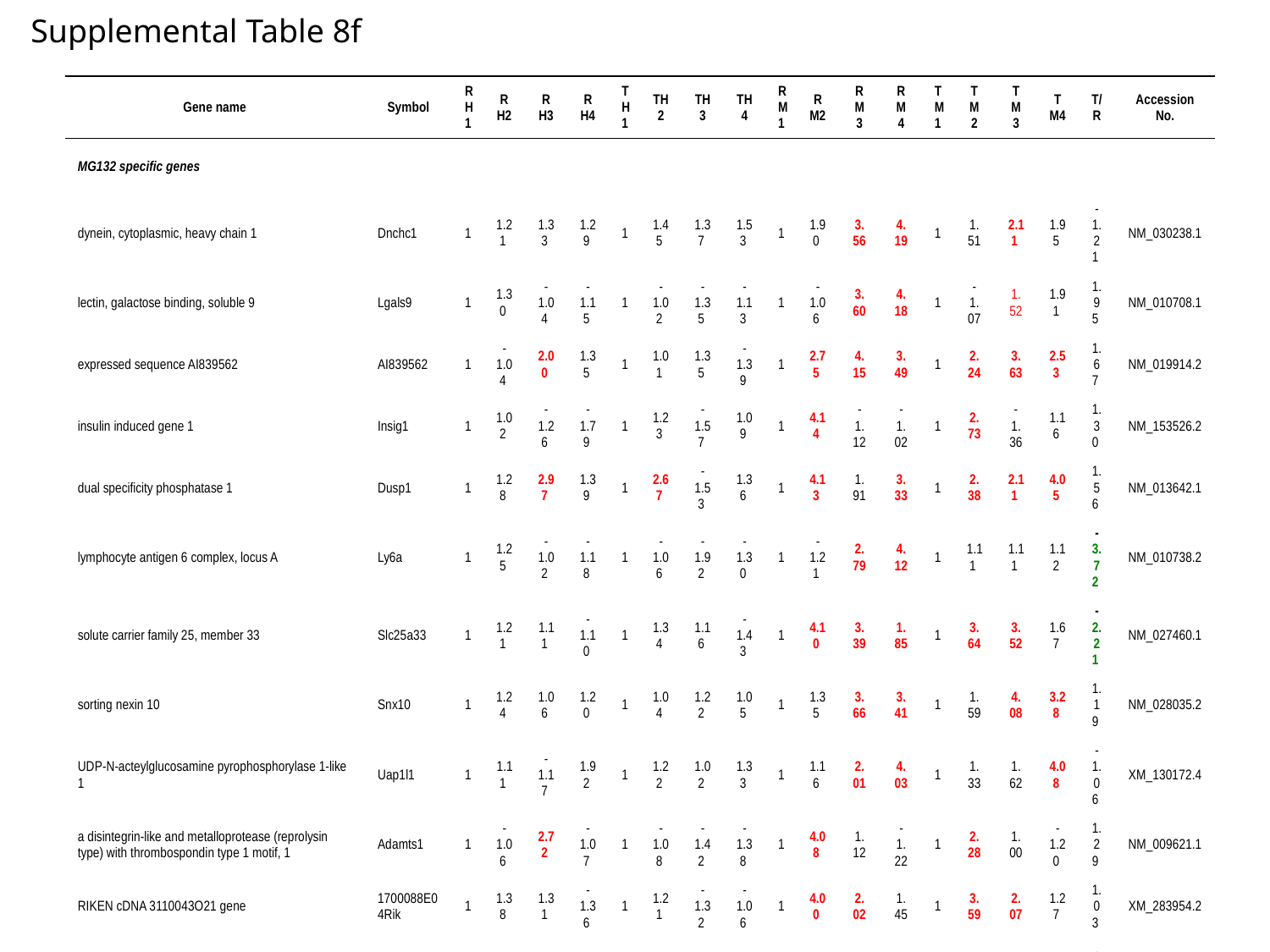

Supplemental Table 8f
| Gene name | Symbol | RH1 | RH2 | RH3 | RH4 | TH1 | TH2 | TH3 | TH4 | RM1 | RM2 | RM3 | RM4 | TM1 | TM2 | TM3 | TM4 | T/R | Accession No. |
| --- | --- | --- | --- | --- | --- | --- | --- | --- | --- | --- | --- | --- | --- | --- | --- | --- | --- | --- | --- |
| MG132 specific genes | | | | | | | | | | | | | | | | | | | |
| dynein, cytoplasmic, heavy chain 1 | Dnchc1 | 1 | 1.21 | 1.33 | 1.29 | 1 | 1.45 | 1.37 | 1.53 | 1 | 1.90 | 3.56 | 4.19 | 1 | 1.51 | 2.11 | 1.95 | -1.21 | NM\_030238.1 |
| lectin, galactose binding, soluble 9 | Lgals9 | 1 | 1.30 | -1.04 | -1.15 | 1 | -1.02 | -1.35 | -1.13 | 1 | -1.06 | 3.60 | 4.18 | 1 | -1.07 | 1.52 | 1.91 | 1.95 | NM\_010708.1 |
| expressed sequence AI839562 | AI839562 | 1 | -1.04 | 2.00 | 1.35 | 1 | 1.01 | 1.35 | -1.39 | 1 | 2.75 | 4.15 | 3.49 | 1 | 2.24 | 3.63 | 2.53 | 1.67 | NM\_019914.2 |
| insulin induced gene 1 | Insig1 | 1 | 1.02 | -1.26 | -1.79 | 1 | 1.23 | -1.57 | 1.09 | 1 | 4.14 | -1.12 | -1.02 | 1 | 2.73 | -1.36 | 1.16 | 1.30 | NM\_153526.2 |
| dual specificity phosphatase 1 | Dusp1 | 1 | 1.28 | 2.97 | 1.39 | 1 | 2.67 | -1.53 | 1.36 | 1 | 4.13 | 1.91 | 3.33 | 1 | 2.38 | 2.11 | 4.05 | 1.56 | NM\_013642.1 |
| lymphocyte antigen 6 complex, locus A | Ly6a | 1 | 1.25 | -1.02 | -1.18 | 1 | -1.06 | -1.92 | -1.30 | 1 | -1.21 | 2.79 | 4.12 | 1 | 1.11 | 1.11 | 1.12 | -3.72 | NM\_010738.2 |
| solute carrier family 25, member 33 | Slc25a33 | 1 | 1.21 | 1.11 | -1.10 | 1 | 1.34 | 1.16 | -1.43 | 1 | 4.10 | 3.39 | 1.85 | 1 | 3.64 | 3.52 | 1.67 | -2.21 | NM\_027460.1 |
| sorting nexin 10 | Snx10 | 1 | 1.24 | 1.06 | 1.20 | 1 | 1.04 | 1.22 | 1.05 | 1 | 1.35 | 3.66 | 3.41 | 1 | 1.59 | 4.08 | 3.28 | 1.19 | NM\_028035.2 |
| UDP-N-acteylglucosamine pyrophosphorylase 1-like 1 | Uap1l1 | 1 | 1.11 | -1.17 | 1.92 | 1 | 1.22 | 1.02 | 1.33 | 1 | 1.16 | 2.01 | 4.03 | 1 | 1.33 | 1.62 | 4.08 | -1.06 | XM\_130172.4 |
| a disintegrin-like and metalloprotease (reprolysin type) with thrombospondin type 1 motif, 1 | Adamts1 | 1 | -1.06 | 2.72 | -1.07 | 1 | -1.08 | -1.42 | -1.38 | 1 | 4.08 | 1.12 | -1.22 | 1 | 2.28 | 1.00 | -1.20 | 1.29 | NM\_009621.1 |
| RIKEN cDNA 3110043O21 gene | 1700088E04Rik | 1 | 1.38 | 1.31 | -1.36 | 1 | 1.21 | -1.32 | -1.06 | 1 | 4.00 | 2.02 | 1.45 | 1 | 3.59 | 2.07 | 1.27 | 1.03 | XM\_283954.2 |
| xanthine dehydrogenase | Xdh | 1 | 1.33 | 1.10 | 1.29 | 1 | 1.34 | -1.49 | 1.49 | 1 | 2.18 | 2.59 | 3.99 | 1 | 2.01 | 2.21 | 3.43 | -1.37 | NM\_011723 |
| N-myc downstream regulated gene 4 | Ndrg4 | 1 | 1.05 | -1.01 | 1.02 | 1 | -1.06 | -1.07 | -1.10 | 1 | 2.46 | 3.82 | 3.85 | 1 | 2.33 | 3.98 | 2.79 | 1.00 | NM\_145602.1 |
| arrestin domain containing 4 | Arrdc4 | 1 | -1.08 | 1.12 | 1.52 | 1 | 1.12 | 1.04 | -1.01 | 1 | 1.46 | 2.53 | 3.17 | 1 | 2.13 | 3.62 | 3.97 | 2.36 | NM\_025549.1 |
| solute carrier family 6 (neurotransmitter transporter, glycine), member 9 | Slc6a9 | 1 | 2.27 | 1.55 | -1.15 | 1 | 2.13 | -1.42 | 1.30 | 1 | 3.41 | 2.88 | 2.24 | 1 | 3.93 | 3.78 | 1.85 | -1.36 | NM\_008135.1 |
| imprinted and ancient | Impact | 1 | -1.31 | 1.67 | 1.35 | 1 | -1.14 | -1.08 | -1.59 | 1 | 3.04 | 3.92 | 3.32 | 1 | 3.01 | 3.78 | 3.03 | 1.05 | NM\_008378.1 |
| RIKEN cDNA B230312A22 gene | B230312A22Rik | 1 | -1.29 | 1.48 | 1.04 | 1 | 1.35 | -1.14 | 1.04 | 1 | 1.04 | 3.41 | 2.55 | 1 | 1.23 | 3.92 | 2.37 | -1.10 | NM\_172691.1 |
| E26 avian leukemia oncogene 2, 3 domain. | Ets2 | 1 | -1.02 | 1.09 | -2.35 | 1 | 1.10 | -1.78 | -1.23 | 1 | 3.42 | 1.25 | -1.24 | 1 | 3.92 | 1.37 | 1.01 | -1.19 | NM\_011809.2 |
| interferon-induced protein 35 | Ifi35 | 1 | -1.03 | -1.17 | -1.17 | 1 | -1.16 | -1.25 | -1.24 | 1 | 1.08 | 3.92 | 3.57 | 1 | 1.05 | 2.21 | 1.88 | 1.81 | NM\_027320.1 |
| three prime repair exonuclease 1 | Trex1 | 1 | -1.37 | -1.04 | -1.00 | 1 | -1.42 | -1.22 | -1.17 | 1 | 3.89 | 3.46 | 2.32 | 1 | 2.23 | 1.50 | 1.14 | 1.06 | NM\_011637.4 |
| SRY-box containing gene 4 | Sox4 | 1 | -1.16 | 1.70 | 1.48 | 1 | 1.58 | 1.71 | 1.28 | 1 | 3.89 | 1.13 | 2.09 | 1 | 2.82 | -1.28 | 1.39 | -1.56 | NM\_009238.1 |
| vascular endothelial growth factor A | Vegfa | 1 | 1.01 | 1.80 | -2.74 | 1 | 2.28 | -1.61 | -1.23 | 1 | 3.88 | -1.11 | 1.03 | 1 | 3.29 | -1.27 | 1.36 | -1.03 | NM\_009505.2 |
| histocompatibility 2, T region locus 23 | H2-T23 | 1 | 1.31 | 1.12 | -1.03 | 1 | 1.01 | -1.48 | -1.04 | 1 | 1.52 | 2.37 | 3.88 | 1 | 1.35 | 1.22 | 1.58 | 1.43 | NM\_010398.1 |
| tumor necrosis factor receptor superfamily, member 6 | Tnfrsf6 | 1 | -1.39 | 1.18 | -1.01 | 1 | -1.41 | -2.25 | -1.90 | 1 | 3.01 | 1.20 | 1.09 | 1 | 3.87 | 1.64 | 1.27 | 1.88 | NM\_007987.1 |
| solute carrier family 20, member 1 | Slc20a1 | 1 | -1.13 | 1.58 | -1.34 | 1 | 1.74 | -1.23 | -1.60 | | 2.11 | -1.48 | -1.67 | 1 | 3.87 | 1.28 | -1.29 | -1.20 | NM\_015747.1 |
| adrenergic receptor, beta 2 | Adrb2 | 1 | -1.24 | 2.24 | 1.07 | 1 | 2.41 | -1.56 | 1.10 | 1 | 3.83 | 1.55 | 1.09 | 1 | 1.72 | 1.56 | 1.12 | 1.66 | NM\_007420.2 |
| dipeptidylpeptidase 7 | Dpp7 | 1 | 1.01 | 1.71 | 1.25 | 1 | 1.51 | -1.14 | -1.11 | 1 | 1.93 | 2.33 | 3.83 | 1 | 1.73 | 2.45 | 3.37 | 1.20 | NM\_031843.2 |
| ATP-binding cassette, sub-family B (MDR/TAP), member 6 | Abcb6 | 1 | -1.28 | -1.36 | 1.00 | 1 | -1.30 | -1.24 | -1.22 | 1 | 1.13 | 3.64 | 2.81 | 1 | 1.12 | 3.81 | 2.55 | 1.07 | NM\_023732.2 |
| adenosine deaminase, RNA-specific | Adar | 1 | 1.02 | -1.08 | 1.14 | 1 | -1.16 | -1.09 | 1.12 | 1 | -1.11 | 3.79 | 3.73 | 1 | -1.21 | 2.16 | 1.94 | 1.21 | NM\_019655.2 |
| CD1d1 antigen | Cd1d1 | 1 | 1.04 | -1.02 | 1.80 | 1 | 1.03 | 1.48 | 1.04 | 1 | 1.02 | 2.78 | 3.52 | 1 | 1.37 | 2.97 | 3.78 | -1.40 | NM\_007639.1 |
| glycoprotein (transmembrane) nmb | Gpnmb | 1 | 1.30 | 1.21 | 1.66 | 1 | 1.11 | -1.16 | 1.15 | 1 | 1.33 | 1.41 | 3.78 | 1 | 1.30 | 1.19 | 2.22 | 1.16 | NM\_053110.2 |
| WD repeat domain 45 | Wdr45 | 1 | 1.51 | 1.28 | 1.80 | 1 | 1.31 | 1.19 | 1.61 | 1 | -1.21 | 2.41 | 3.53 | 1 | 1.11 | 3.13 | 3.78 | 1.15 | NM\_172372.1 |
| hydroxy-delta-5-steroid dehydrogenase, 3 beta- and steroid delta-isomerase 7 | Hsd3b7 | 1 | 1.11 | -1.08 | 1.78 | 1 | -1.21 | 1.18 | 1.03 | 1 | -1.24 | 3.13 | 3.77 | 1 | 1.07 | 3.13 | 3.67 | 1.28 | NM\_133943.1 |
| mitogen activated protein kinase 9 | Mapk9 | 1 | 1.13 | 1.20 | 1.06 | 1 | 1.14 | 1.30 | 1.00 | 1 | 1.31 | 3.76 | 2.46 | 1 | 1.36 | 3.40 | 2.17 | -1.06 | NM\_207692.1 |
| plasma membrane associated protein, S3-12 | S3-12 | 1 | 1.08 | 1.06 | 1.57 | 1 | 1.04 | 1.04 | 1.14 | 1 | 1.13 | 1.78 | 3.75 | 1 | 1.13 | 1.37 | 2.44 | 1.12 | NM\_020568.1 |
| solute carrier family 48 (heme transporter), member 1 | Slc48a1 | 1 | -1.07 | 1.19 | -1.15 | 1 | 1.09 | 1.06 | -1.15 | 1 | 1.99 | 3.72 | 3.06 | 1 | 1.48 | 3.08 | 2.71 | -1.41 | NM\_026353.2 |
| complement component 2 (within H-2S) (C2) | C2 | 1 | 1.22 | 1.23 | -1.04 | 1 | -1.08 | -1.40 | -1.13 | 1 | 1.17 | 1.83 | 3.71 | 1 | 1.17 | 1.18 | 1.55 | -1.10 | NM\_013484.1 |
| torsin family 3, member A | Tor3a | 1 | 1.44 | -1.04 | -1.22 | 1 | 1.27 | -1.61 | 1.21 | 1 | 1.57 | 3.70 | 3.03 | 1 | 1.60 | 2.18 | 2.15 | 1.06 | NM\_023141.1 |
| RUN and SH3 domain containing 1 | Rusc1 | 1 | 1.01 | -1.23 | 1.61 | 1 | -1.12 | 1.20 | 1.10 | 1 | 1.11 | 3.69 | 2.54 | 1 | 1.11 | 3.24 | 2.21 | 1.03 | NM\_028188.1 |
| FLYWCH-type zinc finger 1 | Flywch1 | 1 | -1.07 | 1.11 | 1.25 | 1 | 1.08 | 1.34 | 1.19 | 1 | 1.45 | 3.41 | 1.89 | 1 | 1.48 | 3.68 | 1.93 | -1.09 | NM\_153791.1 |
| platelet-activating factor acetylhydrolase 2 | Pafah2 | 1 | -1.02 | -1.07 | 1.12 | 1 | -1.02 | 1.02 | -1.05 | 1 | 1.34 | 3.67 | 2.89 | 1 | 1.35 | 3.55 | 2.65 | 1.25 | NM\_133880 |

## Slide 7
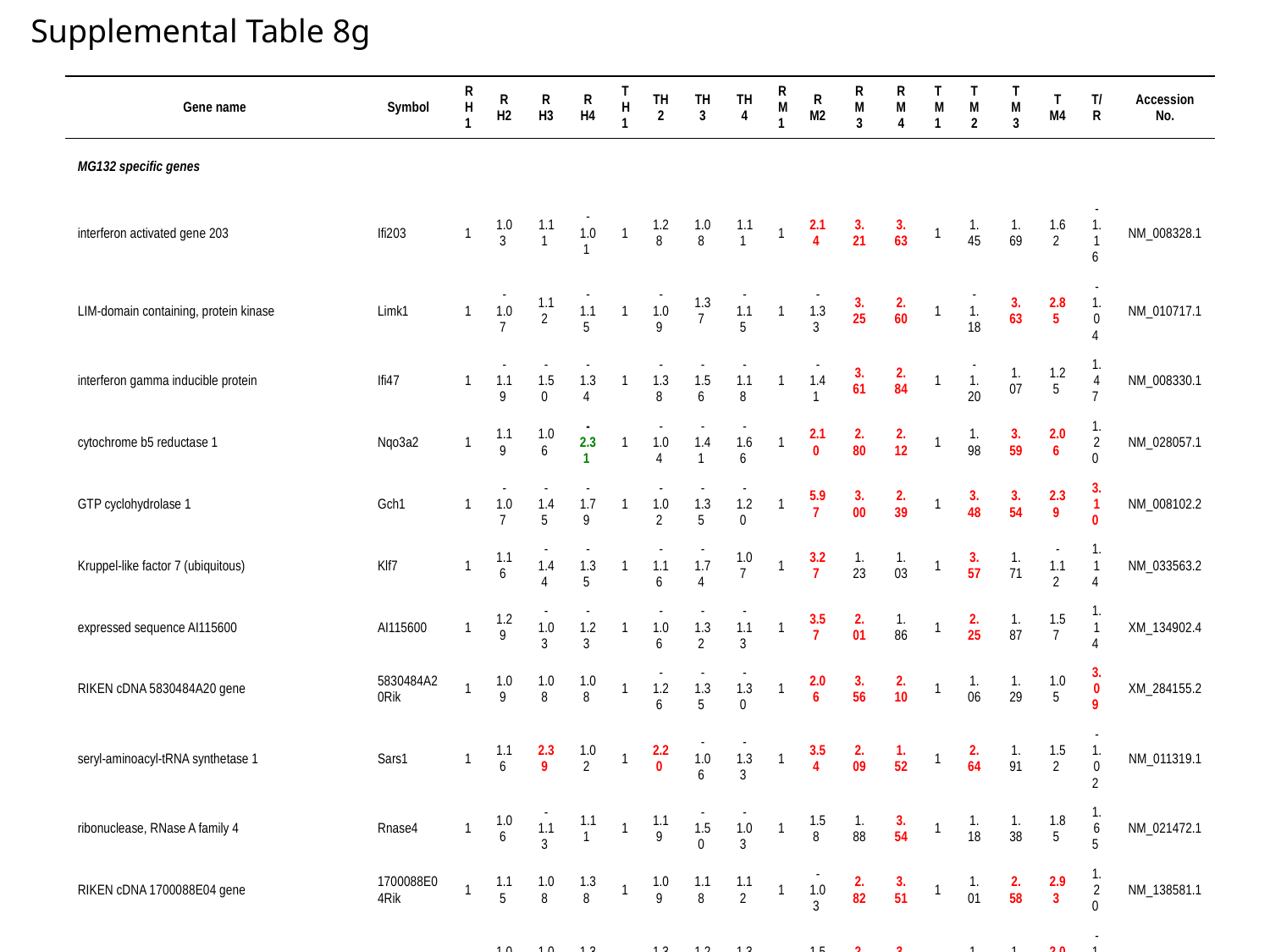

Supplemental Table 8g
| Gene name | Symbol | RH1 | RH2 | RH3 | RH4 | TH1 | TH2 | TH3 | TH4 | RM1 | RM2 | RM3 | RM4 | TM1 | TM2 | TM3 | TM4 | T/R | Accession No. |
| --- | --- | --- | --- | --- | --- | --- | --- | --- | --- | --- | --- | --- | --- | --- | --- | --- | --- | --- | --- |
| MG132 specific genes | | | | | | | | | | | | | | | | | | | |
| interferon activated gene 203 | Ifi203 | 1 | 1.03 | 1.11 | -1.01 | 1 | 1.28 | 1.08 | 1.11 | 1 | 2.14 | 3.21 | 3.63 | 1 | 1.45 | 1.69 | 1.62 | -1.16 | NM\_008328.1 |
| LIM-domain containing, protein kinase | Limk1 | 1 | -1.07 | 1.12 | -1.15 | 1 | -1.09 | 1.37 | -1.15 | 1 | -1.33 | 3.25 | 2.60 | 1 | -1.18 | 3.63 | 2.85 | -1.04 | NM\_010717.1 |
| interferon gamma inducible protein | Ifi47 | 1 | -1.19 | -1.50 | -1.34 | 1 | -1.38 | -1.56 | -1.18 | 1 | -1.41 | 3.61 | 2.84 | 1 | -1.20 | 1.07 | 1.25 | 1.47 | NM\_008330.1 |
| cytochrome b5 reductase 1 | Nqo3a2 | 1 | 1.19 | 1.06 | -2.31 | 1 | -1.04 | -1.41 | -1.66 | 1 | 2.10 | 2.80 | 2.12 | 1 | 1.98 | 3.59 | 2.06 | 1.20 | NM\_028057.1 |
| GTP cyclohydrolase 1 | Gch1 | 1 | -1.07 | -1.45 | -1.79 | 1 | -1.02 | -1.35 | -1.20 | 1 | 5.97 | 3.00 | 2.39 | 1 | 3.48 | 3.54 | 2.39 | 3.10 | NM\_008102.2 |
| Kruppel-like factor 7 (ubiquitous) | Klf7 | 1 | 1.16 | -1.44 | -1.35 | 1 | -1.16 | -1.74 | 1.07 | 1 | 3.27 | 1.23 | 1.03 | 1 | 3.57 | 1.71 | -1.12 | 1.14 | NM\_033563.2 |
| expressed sequence AI115600 | AI115600 | 1 | 1.29 | -1.03 | -1.23 | 1 | -1.06 | -1.32 | -1.13 | 1 | 3.57 | 2.01 | 1.86 | 1 | 2.25 | 1.87 | 1.57 | 1.14 | XM\_134902.4 |
| RIKEN cDNA 5830484A20 gene | 5830484A20Rik | 1 | 1.09 | 1.08 | 1.08 | 1 | -1.26 | -1.35 | -1.30 | 1 | 2.06 | 3.56 | 2.10 | 1 | 1.06 | 1.29 | 1.05 | 3.09 | XM\_284155.2 |
| seryl-aminoacyl-tRNA synthetase 1 | Sars1 | 1 | 1.16 | 2.39 | 1.02 | 1 | 2.20 | -1.06 | -1.33 | 1 | 3.54 | 2.09 | 1.52 | 1 | 2.64 | 1.91 | 1.52 | -1.02 | NM\_011319.1 |
| ribonuclease, RNase A family 4 | Rnase4 | 1 | 1.06 | -1.13 | 1.11 | 1 | 1.19 | -1.50 | -1.03 | 1 | 1.58 | 1.88 | 3.54 | 1 | 1.18 | 1.38 | 1.85 | 1.65 | NM\_021472.1 |
| RIKEN cDNA 1700088E04 gene | 1700088E04Rik | 1 | 1.15 | 1.08 | 1.38 | 1 | 1.09 | 1.18 | 1.12 | 1 | -1.03 | 2.82 | 3.51 | 1 | 1.01 | 2.58 | 2.93 | 1.20 | NM\_138581.1 |
| ATPase, H+ transporting, V0 subunit B | Atp6v0b | 1 | 1.04 | 1.03 | 1.30 | 1 | 1.35 | 1.22 | 1.38 | 1 | 1.51 | 2.40 | 3.50 | 1 | 1.32 | 1.40 | 2.08 | -1.02 | NM\_033617.1 |
| glycerol kinase 2 | Gyk | 1 | -1.16 | -1.33 | -1.09 | 1 | -1.26 | -1.27 | -1.36 | 1 | 1.17 | 2.75 | 1.25 | 1 | 1.18 | 3.49 | 1.51 | -1.01 | NM\_008194 |
| cyclin-dependent kinase 5, regulatory subunit 1 (p35) | Cdk5r1 | 1 | 1.21 | 2.09 | -1.14 | 1 | 1.11 | -1.29 | -1.53 | 1 | 3.48 | 1.22 | -1.10 | 1 | 2.51 | 1.16 | -1.18 | 1.06 | NM\_009871.2 |
| histocompatibility 2, D region locus 1 | H2-D1 | 1 | 1.19 | 1.08 | -1.24 | 1 | 1.09 | -1.02 | -1.04 | 1 | 2.04 | 3.38 | 3.48 | 1 | 1.45 | 2.36 | 2.17 | 1.41 | NM\_010380 |
| phospholipase D3 | Pld3 | 1 | 1.30 | 1.26 | 1.11 | 1 | 1.37 | -1.06 | 1.26 | 1 | 1.26 | 1.91 | 3.46 | 1 | 1.34 | 1.62 | 2.70 | -1.04 | NM\_011116.1 |
| hypothetical protein MGC6357 | MGC6357 | 1 | -1.00 | -1.02 | 1.52 | 1 | -1.30 | -1.05 | -1.38 | 1 | 1.14 | 3.43 | 2.05 | 1 | 1.11 | 2.16 | 1.37 | 1.34 | NM\_144791.1 |
| histocompatibility 2, T region locus 9 | H2-T9 | 1 | 1.02 | -1.06 | -1.30 | 1 | -1.04 | -1.30 | -1.05 | 1 | -1.04 | 3.42 | 3.28 | 1 | -1.21 | 1.34 | 1.43 | 1.25 | NM\_010399 |
| T-cell, immune regulator 1 | Tcirg1 | 1 | -1.11 | -1.34 | 1.20 | 1 | -1.23 | -1.13 | 1.49 | 1 | 1.72 | 3.41 | 2.84 | 1 | 1.34 | 1.41 | 1.38 | 1.32 | NM\_016921.2 |
| pleckstrin homology domain containing, family F (with FYVE domain) member 2 | Plekhf2 | 1 | 1.10 | 1.54 | 1.45 | 1 | 1.51 | 1.06 | -1.15 | 1 | 3.40 | 1.64 | 1.23 | 1 | 3.28 | 1.32 | 1.04 | -1.04 | NM\_175175.3 |
| scotin gene | Scotin | 1 | 1.14 | 1.20 | 1.07 | 1 | 1.14 | -1.11 | 1.02 | 1 | -1.04 | 1.45 | 3.38 | 1 | -1.14 | 1.02 | 1.86 | 1.32 | NM\_026381.1 |
| RIKEN cDNA 2810439F02 gene | 2810439F02Rik | 1 | -1.04 | -1.09 | -1.82 | 1 | 1.02 | -1.22 | -1.29 | 1 | 3.04 | 1.60 | -1.28 | 1 | 3.38 | 1.66 | -1.01 | -1.23 | NM\_028341.1 |
| kinesin family member 3C | Kif3c | 1 | 1.24 | 1.69 | 1.94 | 1 | 1.10 | 1.32 | 1.42 | 1 | 1.95 | 3.38 | 2.53 | 1 | 2.29 | 2.99 | 1.99 | 1.17 | NM\_008445.1 |
| coiled-coil domain containing 117 | Ccdc117 | 1 | -1.05 | 1.94 | -1.80 | 1 | 1.81 | -1.58 | -1.18 | 1 | 3.35 | 2.69 | 1.84 | 1 | 2.09 | 1.66 | 1.04 | 1.57 | NM\_134033.1 |
| ubiquitin specific protease 20 | Usp20 | 1 | -1.01 | -1.12 | 1.20 | 1 | -1.18 | 1.17 | 1.17 | 1 | -1.04 | 2.73 | 3.34 | 1 | -1.13 | 2.65 | 2.53 | 1.23 | NM\_028846.1 |
| calcium modulating ligand | Caml | 1 | -1.26 | 1.21 | 1.31 | 1 | -1.06 | 1.24 | 1.11 | 1 | 1.74 | 3.32 | 2.13 | 1 | 1.42 | 2.78 | 1.52 | -1.12 | NM\_007596.1 |
| cytochrome c oxidase, subunit VI a, polypeptide 2 | Cox6a2 | 1 | 1.03 | 1.03 | -2.17 | 1 | 1.04 | -1.43 | -1.50 | 1 | 1.89 | 2.02 | 2.67 | 1 | 1.47 | 2.91 | 3.31 | -2.29 | NM\_009943.1 |
| osteoclast inhibitory lectin | Ocil | 1 | -1.26 | -1.26 | -1.07 | 1 | -2.13 | -1.79 | -1.22 | 1 | -1.00 | 2.49 | 3.31 | 1 | 1.29 | 1.31 | 1.33 | 1.82 | NM\_053109.1 |
| agrin | Agrn | 1 | -1.05 | 1.04 | -1.35 | 1 | 1.17 | -1.02 | 1.02 | | 1.21 | 1.85 | 3.31 | 1 | -1.09 | -1.02 | 1.37 | -2.70 | NM\_021604.2 |
| granulin | Grn | 1 | 1.20 | 1.07 | 1.43 | 1 | 1.15 | -1.20 | 1.09 | 1 | 1.08 | 1.84 | 3.30 | 1 | 1.12 | 1.07 | 1.72 | 1.37 | NM\_008175.2 |
| family with sequence similarity 110, member C | Fam110c | 1 | -1.02 | -1.10 | -1.29 | 1 | -1.23 | -1.15 | -1.20 | 1 | 3.28 | -1.47 | -1.37 | 1 | 2.28 | -1.13 | -1.20 | -1.03 | NM\_027828.2 |
| 1-acylglycerol-3-phosphatate O-acyltransferase 9 | Agpat9 | 1 | 1.23 | 2.61 | 1.59 | 1 | 2.37 | 1.02 | -1.02 | 1 | 3.26 | 1.99 | 1.66 | 1 | 2.38 | 1.88 | 1.51 | -1.14 | NM\_172715.1 |
| imprinted and ancient | Impact | 1 | -1.16 | 1.65 | 1.04 | 1 | 1.22 | -1.09 | -1.29 | 1 | 2.65 | 3.26 | 2.74 | 1 | 2.35 | 2.61 | 1.75 | -1.09 | NM\_008378 |
| nicotinate phosphoribosyltransferase domain containing 1 | Naprt1 | 1 | -1.57 | 1.30 | -1.28 | 1 | 1.18 | -1.26 | -1.60 | 1 | 3.26 | 1.26 | -1.11 | 1 | 1.99 | 1.18 | -1.30 | 1.08 | NM\_172607.2 |
| GTP binding protein 2 | Gtpbp2 | 1 | 1.73 | 1.02 | 1.50 | 1 | 1.07 | 1.08 | 1.95 | 1 | 1.97 | 2.81 | 3.25 | 1 | 1.58 | 2.20 | 1.91 | -1.60 | NM\_019581.2 |
| POU domain, class 6, transcription factor 1 | Pou6f1 | 1 | 1.49 | 1.28 | 1.99 | 1 | -1.10 | 1.09 | 1.15 | 1 | 1.25 | 3.25 | 2.59 | 1 | 1.30 | 2.17 | 1.88 | -1.38 | NM\_010127.2 |
| gamma-aminobutyric acid (GABA(A)) receptor-associated protein-like 1 | Gabarapl1 | 1 | 1.74 | 2.28 | 1.97 | 1 | 1.33 | 1.50 | 1.20 | 1 | 1.64 | 3.25 | 3.05 | 1 | 1.59 | 2.84 | 2.49 | -1.00 | NM\_020590.3 |
| kinesin family member 1B , 1 | Kif1b | 1 | 1.45 | 1.45 | 1.69 | 1 | 1.09 | -1.31 | 1.42 | 1 | 1.33 | 2.65 | 3.24 | 1 | 1.06 | 2.42 | 2.37 | -1.36 | NM\_207682.1 |
| torsin family 3, member A | Tor3a | 1 | 1.28 | -1.05 | -1.34 | 1 | 1.12 | -1.46 | 1.06 | 1 | 1.41 | 3.24 | 2.51 | 1 | 1.69 | 2.07 | 1.84 | 1.03 | NM\_023141.1 |
| histocompatibility 2, K region locus 1 | H2-K1 | 1 | 1.05 | -1.09 | -2.26 | 1 | 1.07 | -1.67 | -1.26 | 1 | 1.53 | 2.27 | 3.23 | 1 | 1.23 | 1.47 | 1.50 | 1.23 | NM\_001001892 |
| RIKEN cDNA D430039N05 gene | D430039N05Rik | 1 | 1.38 | 1.31 | 1.25 | 1 | -1.04 | -1.03 | 1.09 | 1 | 3.23 | 1.23 | 1.11 | 1 | 1.77 | 1.03 | 1.26 | 1.12 | NM\_175514.1 |
| neural precursor cell expressed, developmentally down-regulated gene 4-like | Nedd4l | 1 | 1.13 | 1.16 | 1.31 | 1 | 1.04 | -1.23 | 1.04 | 1 | 3.08 | 2.87 | 3.20 | 1 | 1.88 | 1.71 | 1.30 | 1.14 | NM\_031881.1 |
| interferon-induced protein with tetratricopeptide repeats 2 | Ifit2 | 1 | 1.12 | -1.23 | 1.14 | 1 | -1.16 | -1.41 | -1.08 | 1 | 2.05 | 3.20 | 2.66 | 1 | -1.10 | 1.40 | 1.82 | 1.59 | NM\_008332.2 |

## Slide 8
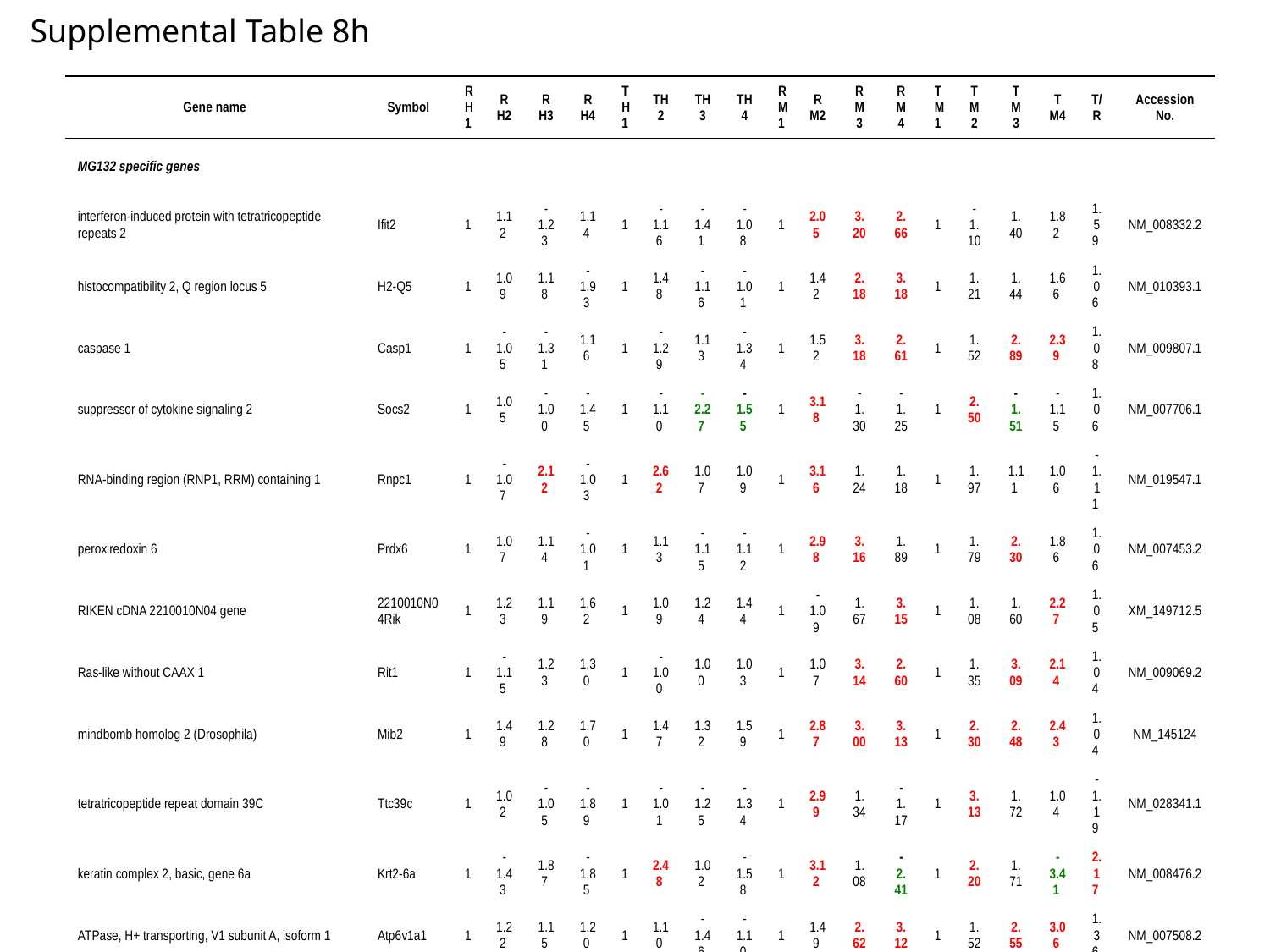

Supplemental Table 8h
| Gene name | Symbol | RH1 | RH2 | RH3 | RH4 | TH1 | TH2 | TH3 | TH4 | RM1 | RM2 | RM3 | RM4 | TM1 | TM2 | TM3 | TM4 | T/R | Accession No. |
| --- | --- | --- | --- | --- | --- | --- | --- | --- | --- | --- | --- | --- | --- | --- | --- | --- | --- | --- | --- |
| MG132 specific genes | | | | | | | | | | | | | | | | | | | |
| interferon-induced protein with tetratricopeptide repeats 2 | Ifit2 | 1 | 1.12 | -1.23 | 1.14 | 1 | -1.16 | -1.41 | -1.08 | 1 | 2.05 | 3.20 | 2.66 | 1 | -1.10 | 1.40 | 1.82 | 1.59 | NM\_008332.2 |
| histocompatibility 2, Q region locus 5 | H2-Q5 | 1 | 1.09 | 1.18 | -1.93 | 1 | 1.48 | -1.16 | -1.01 | 1 | 1.42 | 2.18 | 3.18 | 1 | 1.21 | 1.44 | 1.66 | 1.06 | NM\_010393.1 |
| caspase 1 | Casp1 | 1 | -1.05 | -1.31 | 1.16 | 1 | -1.29 | 1.13 | -1.34 | 1 | 1.52 | 3.18 | 2.61 | 1 | 1.52 | 2.89 | 2.39 | 1.08 | NM\_009807.1 |
| suppressor of cytokine signaling 2 | Socs2 | 1 | 1.05 | -1.00 | -1.45 | 1 | -1.10 | -2.27 | -1.55 | 1 | 3.18 | -1.30 | -1.25 | 1 | 2.50 | -1.51 | -1.15 | 1.06 | NM\_007706.1 |
| RNA-binding region (RNP1, RRM) containing 1 | Rnpc1 | 1 | -1.07 | 2.12 | -1.03 | 1 | 2.62 | 1.07 | 1.09 | 1 | 3.16 | 1.24 | 1.18 | 1 | 1.97 | 1.11 | 1.06 | -1.11 | NM\_019547.1 |
| peroxiredoxin 6 | Prdx6 | 1 | 1.07 | 1.14 | -1.01 | 1 | 1.13 | -1.15 | -1.12 | 1 | 2.98 | 3.16 | 1.89 | 1 | 1.79 | 2.30 | 1.86 | 1.06 | NM\_007453.2 |
| RIKEN cDNA 2210010N04 gene | 2210010N04Rik | 1 | 1.23 | 1.19 | 1.62 | 1 | 1.09 | 1.24 | 1.44 | 1 | -1.09 | 1.67 | 3.15 | 1 | 1.08 | 1.60 | 2.27 | 1.05 | XM\_149712.5 |
| Ras-like without CAAX 1 | Rit1 | 1 | -1.15 | 1.23 | 1.30 | 1 | -1.00 | 1.00 | 1.03 | 1 | 1.07 | 3.14 | 2.60 | 1 | 1.35 | 3.09 | 2.14 | 1.04 | NM\_009069.2 |
| mindbomb homolog 2 (Drosophila) | Mib2 | 1 | 1.49 | 1.28 | 1.70 | 1 | 1.47 | 1.32 | 1.59 | 1 | 2.87 | 3.00 | 3.13 | 1 | 2.30 | 2.48 | 2.43 | 1.04 | NM\_145124 |
| tetratricopeptide repeat domain 39C | Ttc39c | 1 | 1.02 | -1.05 | -1.89 | 1 | -1.01 | -1.25 | -1.34 | 1 | 2.99 | 1.34 | -1.17 | 1 | 3.13 | 1.72 | 1.04 | -1.19 | NM\_028341.1 |
| keratin complex 2, basic, gene 6a | Krt2-6a | 1 | -1.43 | 1.87 | -1.85 | 1 | 2.48 | 1.02 | -1.58 | 1 | 3.12 | 1.08 | -2.41 | 1 | 2.20 | 1.71 | -3.41 | 2.17 | NM\_008476.2 |
| ATPase, H+ transporting, V1 subunit A, isoform 1 | Atp6v1a1 | 1 | 1.22 | 1.15 | 1.20 | 1 | 1.10 | -1.46 | -1.10 | 1 | 1.49 | 2.62 | 3.12 | 1 | 1.52 | 2.55 | 3.06 | 1.36 | NM\_007508.2 |
| high mobility group box transcription factor 1 | Hbp1 | 1 | 1.66 | 1.89 | 2.93 | 1 | 1.62 | 1.56 | 2.61 | 1 | -1.29 | 1.75 | 3.12 | 1 | -1.14 | 1.76 | 2.59 | -1.26 | NM\_153198 |
| histocompatibility 2, T region locus 23 | H2-T23 | 1 | 1.24 | 1.16 | -1.32 | 1 | 1.38 | -1.29 | 1.22 | 1 | 1.49 | 2.36 | 3.12 | 1 | 1.25 | 1.50 | 1.53 | 1.21 | NM\_010398 |
| cell cycle progression 1 | Ccpg1 | 1 | 1.15 | 1.28 | -1.25 | 1 | 1.23 | -1.25 | -1.00 | 1 | 1.05 | 1.12 | 3.09 | 1 | 1.13 | 1.30 | 2.72 | 1.25 | NM\_028181.1 |
| SFT2 domain containing 2 | Sft2d2 | 1 | 1.25 | 1.12 | -1.01 | 1 | 1.09 | -1.21 | 1.30 | 1 | 1.22 | 2.19 | 3.08 | 1 | 1.06 | 2.19 | 2.75 | 1.09 | NM\_145512.2 |
| nuclear receptor subfamily 4, group A, member 2 | Nr4a2 | 1 | 1.16 | 1.49 | -1.37 | 1 | 2.33 | -1.60 | -1.43 | | 3.08 | -1.56 | -1.29 | 1 | 2.66 | -1.72 | -1.11 | 1.15 | NM\_013613.1 |
| nidogen 1 | Nid1 | 1 | -1.04 | 1.45 | 1.58 | 1 | 1.01 | 1.95 | 1.34 | 1 | 1.71 | 3.08 | 2.71 | 1 | 1.97 | 2.83 | 2.59 | 1.03 | NM\_010917.1 |
| huntingtin-associated protein 1 (Hap1), transcript variant 1 | Hap1 | 1 | 1.20 | 1.30 | 1.01 | 1 | 1.18 | -1.08 | -1.05 | 1 | -1.03 | 3.08 | 2.34 | 1 | -1.09 | 1.35 | 1.24 | 1.36 | NM\_177981.1 |
| ras homolog gene family, member B | Rhob | 1 | 1.15 | 2.83 | -1.32 | 1 | 1.75 | -1.50 | -1.22 | 1 | 3.07 | 1.43 | 1.86 | 1 | 2.06 | 2.57 | 2.67 | -1.23 | NM\_007483.2 |
| histocompatibility 2, T region locus 17 | H2-T17 | 1 | 1.04 | -1.10 | -1.32 | 1 | -1.06 | -1.33 | -1.06 | 1 | -1.18 | 3.07 | 3.07 | 1 | -1.19 | 1.43 | 1.44 | 1.31 | NM\_010396 |
| AT rich interactive domain 5A (Mrf1 like) | Arid5a | 1 | 1.60 | -1.14 | -1.23 | 1 | -1.24 | -1.15 | -1.30 | 1 | 2.95 | -1.26 | -1.56 | 1 | 3.07 | -1.13 | -1.39 | 1.14 | NM\_145996.2 |
| N-acetylglucosamine kinase | Nagk | 1 | 1.13 | -1.17 | 1.45 | 1 | 1.02 | -1.09 | 1.15 | 1 | -1.26 | 1.73 | 2.38 | 1 | 1.05 | 2.21 | 3.06 | -1.06 | NM\_019542.1 |
| BSD domain containing 1 | Bsdc1 | 1 | 1.63 | 2.09 | 1.78 | 1 | 1.57 | 1.40 | 1.32 | 1 | -1.27 | 2.96 | 3.06 | 1 | -1.06 | 2.80 | 2.40 | 1.11 | NM\_133889.2 |
| kinesin family member 1B, 1 | Kif1b | 1 | 1.08 | 1.00 | 1.10 | 1 | -1.20 | -1.18 | -1.04 | 1 | 1.37 | 2.28 | 3.06 | 1 | 1.22 | 2.53 | 2.27 | 1.12 | NM\_207682.1 |
| tubulin, beta 2b | Tubb2b | 1 | 1.00 | 1.34 | 1.17 | 1 | 1.32 | -1.01 | -1.96 | 1 | 3.05 | 2.85 | 2.13 | 1 | 2.07 | 2.85 | 1.81 | -1.28 | NM\_023716.1 |
| tumor necrosis factor receptor superfamily, member 10b | Tnfrsf10b | 1 | 1.02 | 1.17 | 1.12 | 1 | 1.23 | -1.26 | -1.13 | 1 | 2.51 | 3.05 | 2.06 | 1 | 2.22 | 2.40 | 1.27 | 1.38 | NM\_020275.3 |
| RUN and SH3 domain containing 2 | Rusc2 | 1 | 1.34 | 1.62 | 1.85 | 1 | 1.70 | 1.05 | 1.32 | 1 | 2.00 | 3.00 | 3.05 | 1 | 1.66 | 2.53 | 2.09 | 1.25 | XM\_131380.3 |
| artemin | Artn | 1 | 1.21 | 1.02 | -1.03 | 1 | 1.08 | -1.15 | -1.03 | 1 | 3.04 | 1.22 | -1.25 | 1 | 2.42 | 1.01 | -1.71 | 1.33 | NM\_009711.2 |
| protease, serine, 15 | Prss15 | 1 | 1.32 | 1.18 | 1.17 | 1 | 1.51 | 1.17 | 1.50 | 1 | 1.93 | 2.29 | 3.04 | 1 | 1.77 | 2.16 | 2.19 | -1.53 | XM\_128721.2 |
| Kruppel-like factor 2 (lung) | Klf2 | 1 | -1.21 | -2.48 | -1.76 | 1 | -1.49 | -1.20 | -1.28 | 1 | 3.04 | 1.58 | 2.03 | 1 | 1.85 | 2.50 | 2.33 | -1.29 | NM\_008452.1 |
| folliculin | Flcn | 1 | 1.00 | 1.42 | 1.50 | 1 | 1.83 | 1.10 | 1.47 | 1 | 1.36 | 1.95 | 3.02 | 1 | 1.44 | 1.94 | 2.72 | -1.05 | NM\_146018.1 |
| activating signal cointegrator 1 complex subunit 2 | Ascc2 | 1 | -1.07 | 1.20 | -1.06 | 1 | 1.26 | 1.19 | 1.16 | 1 | 2.13 | 3.01 | 2.50 | 1 | 1.38 | 2.42 | 1.82 | -1.03 | NM\_029291.1 |
| spermidine/spermine N1-acetyl transferase 1 | Sat1 | 1 | 1.17 | 1.33 | 1.61 | 1 | 1.12 | -1.06 | 1.00 | 1 | 2.92 | 2.02 | 2.80 | 1 | 3.01 | 1.84 | 2.60 | -1.16 | NM\_009121.3 |
| fibronectin leucine rich transmembrane protein 3 | Flrt3 | 1 | -1.61 | 1.37 | -2.18 | 1 | -2.02 | -1.65 | -2.56 | 1 | 2.51 | -1.19 | -1.69 | 1 | 3.00 | -1.19 | -1.46 | -1.34 | NM\_178382.2 |
