# Supplementary material for: Systemic Analysis of Heat Shock Response Induced by Heat Shock and a Proteasome Inhibitor MG132
Source: PLoS One. 2011 Jun 30;6(6):e20252. doi: 10.1371/journal.pone.0020252 (PMC3127947; doi:10.1371/journal.pone.0020252)
Supplement: Table S9 — Heat shock and MG132 suppressed genes more than 3 fold in comparison to control cells are listed. Fold changes more than 2 are colored in red and less than -2 are colored in green. T/R means fold differences in TR cells compared to RIF-1 cells. (PPT) [file pone.0020252.s016.ppt]

## Slide 1
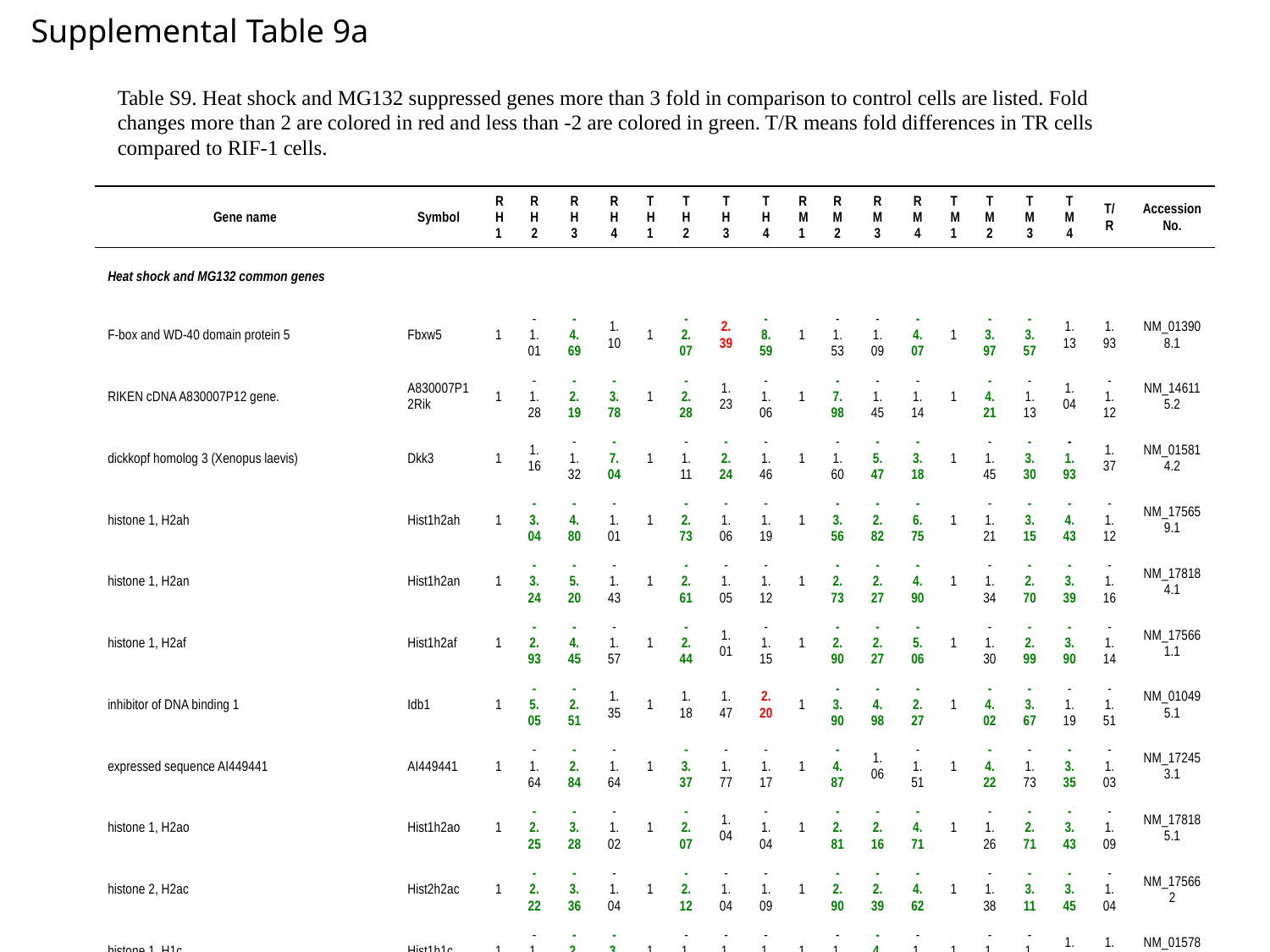

Supplemental Table 9a
Table S9. Heat shock and MG132 suppressed genes more than 3 fold in comparison to control cells are listed. Fold changes more than 2 are colored in red and less than -2 are colored in green. T/R means fold differences in TR cells compared to RIF-1 cells.
| Gene name | Symbol | RH1 | RH2 | RH3 | RH4 | TH1 | TH2 | TH3 | TH4 | RM1 | RM2 | RM3 | RM4 | TM1 | TM2 | TM3 | TM4 | T/R | Accession No. |
| --- | --- | --- | --- | --- | --- | --- | --- | --- | --- | --- | --- | --- | --- | --- | --- | --- | --- | --- | --- |
| Heat shock and MG132 common genes | | | | | | | | | | | | | | | | | | | |
| F-box and WD-40 domain protein 5 | Fbxw5 | 1 | -1.01 | -4.69 | 1.10 | 1 | -2.07 | 2.39 | -8.59 | 1 | -1.53 | -1.09 | -4.07 | 1 | -3.97 | -3.57 | 1.13 | 1.93 | NM\_013908.1 |
| RIKEN cDNA A830007P12 gene. | A830007P12Rik | 1 | -1.28 | -2.19 | -3.78 | 1 | -2.28 | 1.23 | -1.06 | 1 | -7.98 | -1.45 | -1.14 | 1 | -4.21 | -1.13 | 1.04 | -1.12 | NM\_146115.2 |
| dickkopf homolog 3 (Xenopus laevis) | Dkk3 | 1 | 1.16 | -1.32 | -7.04 | 1 | -1.11 | -2.24 | -1.46 | 1 | -1.60 | -5.47 | -3.18 | 1 | -1.45 | -3.30 | -1.93 | 1.37 | NM\_015814.2 |
| histone 1, H2ah | Hist1h2ah | 1 | -3.04 | -4.80 | -1.01 | 1 | -2.73 | -1.06 | -1.19 | 1 | -3.56 | -2.82 | -6.75 | 1 | -1.21 | -3.15 | -4.43 | -1.12 | NM\_175659.1 |
| histone 1, H2an | Hist1h2an | 1 | -3.24 | -5.20 | -1.43 | 1 | -2.61 | -1.05 | -1.12 | 1 | -2.73 | -2.27 | -4.90 | 1 | -1.34 | -2.70 | -3.39 | -1.16 | NM\_178184.1 |
| histone 1, H2af | Hist1h2af | 1 | -2.93 | -4.45 | -1.57 | 1 | -2.44 | 1.01 | -1.15 | 1 | -2.90 | -2.27 | -5.06 | 1 | -1.30 | -2.99 | -3.90 | -1.14 | NM\_175661.1 |
| inhibitor of DNA binding 1 | Idb1 | 1 | -5.05 | -2.51 | 1.35 | 1 | 1.18 | 1.47 | 2.20 | 1 | -3.90 | -4.98 | -2.27 | 1 | -4.02 | -3.67 | -1.19 | -1.51 | NM\_010495.1 |
| expressed sequence AI449441 | AI449441 | 1 | -1.64 | -2.84 | -1.64 | 1 | -3.37 | -1.77 | -1.17 | 1 | -4.87 | 1.06 | -1.51 | 1 | -4.22 | -1.73 | -3.35 | -1.03 | NM\_172453.1 |
| histone 1, H2ao | Hist1h2ao | 1 | -2.25 | -3.28 | -1.02 | 1 | -2.07 | 1.04 | -1.04 | 1 | -2.81 | -2.16 | -4.71 | 1 | -1.26 | -2.71 | -3.43 | -1.09 | NM\_178185.1 |
| histone 2, H2ac | Hist2h2ac | 1 | -2.22 | -3.36 | -1.04 | 1 | -2.12 | -1.04 | -1.09 | 1 | -2.90 | -2.39 | -4.62 | 1 | -1.38 | -3.11 | -3.45 | -1.04 | NM\_175662 |
| histone 1, H1c | Hist1h1c | 1 | -1.35 | -2.04 | -3.27 | 1 | -1.75 | -1.28 | -1.49 | 1 | -1.96 | -4.42 | -1.43 | 1 | -1.13 | -1.22 | 1.16 | 1.93 | NM\_015786 |
| forkhead-like 18 (Drosophila) | Fkhl18 | 1 | -1.38 | -3.51 | -2.20 | 1 | -1.42 | -1.75 | -1.54 | 1 | -2.54 | -4.04 | -3.73 | 1 | -1.50 | -2.19 | -2.60 | -1.61 | NM\_010226.1 |
| dual specificity phosphatase 6 | Dusp6 | 1 | -1.49 | 1.05 | -3.54 | 1 | -1.04 | -1.14 | -1.57 | 1 | -3.98 | -3.66 | -2.72 | 1 | -3.52 | -2.77 | -2.22 | -1.23 | NM\_026268.1 |
| kinesin family member 22 | Kif22 | 1 | -1.39 | -3.28 | -1.21 | 1 | -1.54 | -1.14 | 1.38 | 1 | -3.58 | -1.06 | -1.76 | 1 | -2.82 | -1.41 | -2.49 | -1.18 | NM\_145588.1 |
| cell division cycle associated 2 | Cdca2 | 1 | -1.38 | -3.06 | -1.02 | 1 | -1.78 | -1.00 | 1.31 | 1 | -3.56 | -1.15 | -1.99 | 1 | -2.15 | -1.31 | -2.46 | -1.09 | NM\_175384.3 |
| cyclin E1 | Ccne1 | 1 | -1.46 | -3.29 | -1.03 | 1 | -2.49 | 1.35 | -1.49 | 1 | -3.49 | -2.93 | -3.25 | 1 | -2.83 | -2.94 | -2.21 | -1.17 | NM\_007633.1 |
| latent transforming growth factor beta binding protein 2 | Ltbp2 | 1 | -1.04 | -1.27 | -3.47 | 1 | -1.44 | -2.47 | -1.49 | 1 | 1.11 | -1.78 | -3.04 | 1 | 1.08 | -1.53 | -2.44 | -1.42 | NM\_013589.1 |
| COMM domain containing 5 | Commd5 | 1 | -1.31 | -3.02 | 1.05 | 1 | -1.94 | 1.35 | -1.01 | 1 | -3.27 | -1.35 | -1.08 | 1 | -2.49 | -1.21 | 1.07 | -1.11 | NM\_025536.1 |
| RIKEN cDNA 1700018O18 gene | 1700018O18Rik | 1 | -1.37 | -1.92 | -3.18 | 1 | -3.22 | -1.53 | -2.83 | 1 | 2.31 | -1.39 | -3.25 | 1 | 1.39 | -1.63 | -3.11 | 1.07 | XM\_131683.2 |

## Slide 2
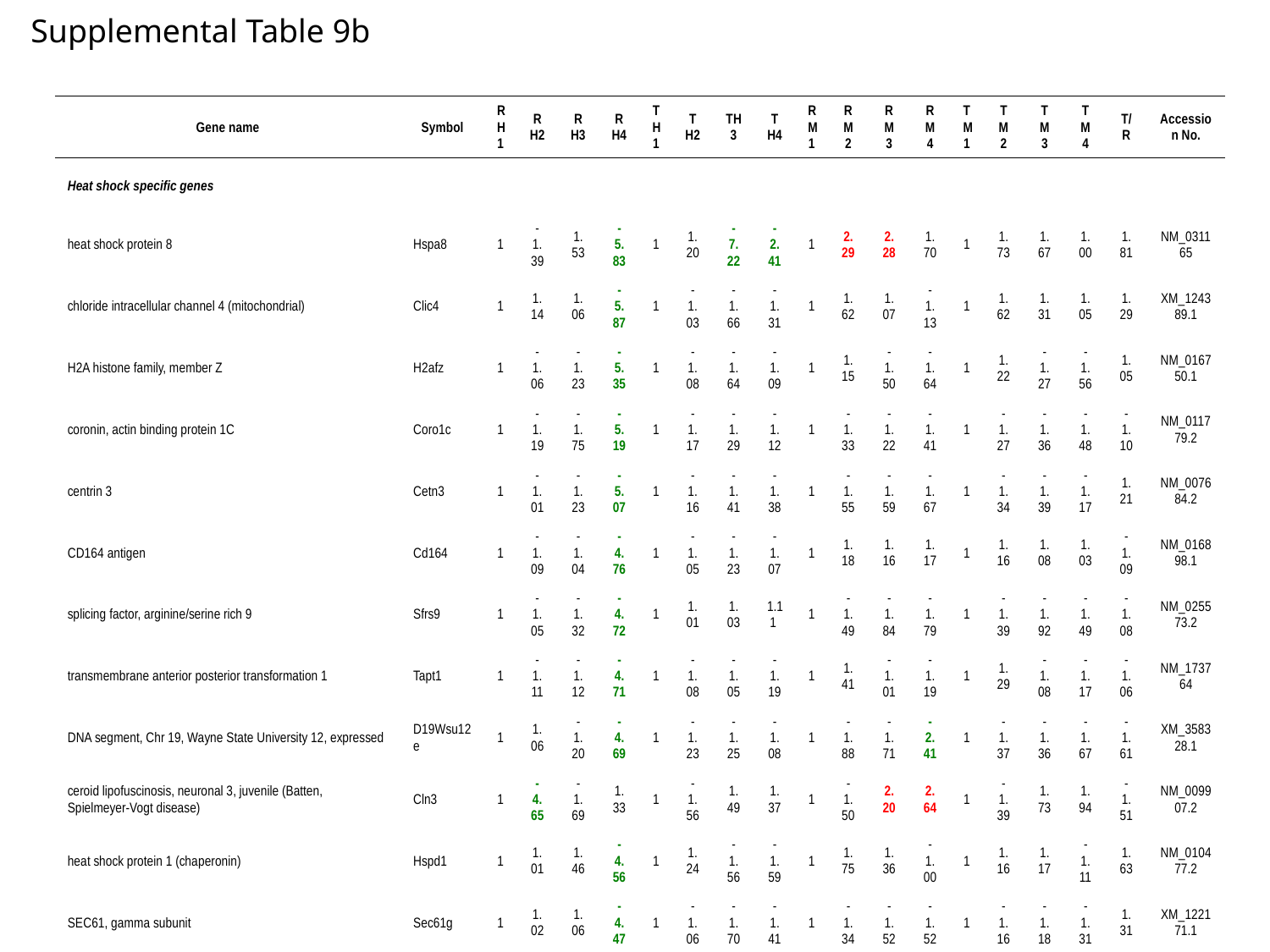

Supplemental Table 9b
| Gene name | Symbol | RH1 | RH2 | RH3 | RH4 | TH1 | TH2 | TH3 | TH4 | RM1 | RM2 | RM3 | RM4 | TM1 | TM2 | TM3 | TM4 | T/R | Accession No. |
| --- | --- | --- | --- | --- | --- | --- | --- | --- | --- | --- | --- | --- | --- | --- | --- | --- | --- | --- | --- |
| Heat shock specific genes | | | | | | | | | | | | | | | | | | | |
| heat shock protein 8 | Hspa8 | 1 | -1.39 | 1.53 | -5.83 | 1 | 1.20 | -7.22 | -2.41 | 1 | 2.29 | 2.28 | 1.70 | 1 | 1.73 | 1.67 | 1.00 | 1.81 | NM\_031165 |
| chloride intracellular channel 4 (mitochondrial) | Clic4 | 1 | 1.14 | 1.06 | -5.87 | 1 | -1.03 | -1.66 | -1.31 | 1 | 1.62 | 1.07 | -1.13 | 1 | 1.62 | 1.31 | 1.05 | 1.29 | XM\_124389.1 |
| H2A histone family, member Z | H2afz | 1 | -1.06 | -1.23 | -5.35 | 1 | -1.08 | -1.64 | -1.09 | 1 | 1.15 | -1.50 | -1.64 | 1 | 1.22 | -1.27 | -1.56 | 1.05 | NM\_016750.1 |
| coronin, actin binding protein 1C | Coro1c | 1 | -1.19 | -1.75 | -5.19 | 1 | -1.17 | -1.29 | -1.12 | 1 | -1.33 | -1.22 | -1.41 | 1 | -1.27 | -1.36 | -1.48 | -1.10 | NM\_011779.2 |
| centrin 3 | Cetn3 | 1 | -1.01 | -1.23 | -5.07 | 1 | -1.16 | -1.41 | -1.38 | 1 | -1.55 | -1.59 | -1.67 | 1 | -1.34 | -1.39 | -1.17 | 1.21 | NM\_007684.2 |
| CD164 antigen | Cd164 | 1 | -1.09 | -1.04 | -4.76 | 1 | -1.05 | -1.23 | -1.07 | 1 | 1.18 | 1.16 | 1.17 | 1 | 1.16 | 1.08 | 1.03 | -1.09 | NM\_016898.1 |
| splicing factor, arginine/serine rich 9 | Sfrs9 | 1 | -1.05 | -1.32 | -4.72 | 1 | 1.01 | 1.03 | 1.11 | 1 | -1.49 | -1.84 | -1.79 | 1 | -1.39 | -1.92 | -1.49 | -1.08 | NM\_025573.2 |
| transmembrane anterior posterior transformation 1 | Tapt1 | 1 | -1.11 | -1.12 | -4.71 | 1 | -1.08 | -1.05 | -1.19 | 1 | 1.41 | -1.01 | -1.19 | 1 | 1.29 | -1.08 | -1.17 | -1.06 | NM\_173764 |
| DNA segment, Chr 19, Wayne State University 12, expressed | D19Wsu12e | 1 | 1.06 | -1.20 | -4.69 | 1 | -1.23 | -1.25 | -1.08 | 1 | -1.88 | -1.71 | -2.41 | 1 | -1.37 | -1.36 | -1.67 | -1.61 | XM\_358328.1 |
| ceroid lipofuscinosis, neuronal 3, juvenile (Batten, Spielmeyer-Vogt disease) | Cln3 | 1 | -4.65 | -1.69 | 1.33 | 1 | -1.56 | 1.49 | 1.37 | 1 | -1.50 | 2.20 | 2.64 | 1 | -1.39 | 1.73 | 1.94 | -1.51 | NM\_009907.2 |
| heat shock protein 1 (chaperonin) | Hspd1 | 1 | 1.01 | 1.46 | -4.56 | 1 | 1.24 | -1.56 | -1.59 | 1 | 1.75 | 1.36 | -1.00 | 1 | 1.16 | 1.17 | -1.11 | 1.63 | NM\_010477.2 |
| SEC61, gamma subunit | Sec61g | 1 | 1.02 | 1.06 | -4.47 | 1 | -1.06 | -1.70 | -1.41 | 1 | -1.34 | -1.52 | -1.52 | 1 | -1.16 | -1.18 | -1.31 | 1.31 | XM\_122171.1 |
| actin, alpha 2, smooth muscle, aorta | Acta2 | 1 | -1.23 | -1.71 | -3.70 | 1 | -1.79 | -4.47 | -4.25 | 1 | 1.93 | -1.20 | -1.70 | 1 | 1.34 | -1.06 | -1.91 | 1.08 | NM\_007392.2 |
| leucine rich repeat containing 59 | Lrrc59 | 1 | -1.39 | -1.05 | -4.41 | 1 | -1.06 | -1.03 | -1.42 | 1 | 1.28 | 1.51 | 1.09 | 1 | 1.12 | 1.42 | 1.01 | 1.13 | NM\_133807.1 |
| small inducible cytokine subfamily E, member 1 | Scye1 | 1 | -1.03 | 1.00 | -4.28 | 1 | -1.13 | -1.03 | -1.08 | 1 | -1.12 | -1.16 | -1.09 | 1 | -1.11 | -1.02 | 1.01 | 1.04 | NM\_007926.1 |
| serum response factor | Srf | 1 | -1.30 | -1.28 | -1.57 | 1 | -1.70 | -4.16 | -1.71 | 1 | 1.71 | -1.41 | -1.34 | 1 | 1.77 | -1.01 | -1.19 | 1.13 | NM\_020493 |
| actin, alpha 2, smooth muscle, aorta | Acta2 | 1 | 1.11 | -1.20 | -4.12 | 1 | -1.23 | -3.16 | -3.23 | 1 | 1.48 | -1.13 | -1.49 | 1 | 1.05 | 1.02 | -1.26 | -1.25 | NM\_007392.2 |
| proteolipid protein 2 | Plp2 | 1 | -1.13 | -1.18 | -4.08 | 1 | 1.03 | -1.57 | -1.14 | 1 | -1.28 | -1.50 | -1.31 | 1 | -1.06 | -1.14 | -1.32 | -1.04 | NM\_019755.2 |
| WASP family 1 | Wasf1 | 1 | 1.04 | -1.23 | -4.08 | 1 | -1.20 | -1.25 | 1.02 | 1 | -1.08 | -1.50 | -1.31 | 1 | 1.05 | -1.30 | -1.22 | -1.05 | NM\_031877.2 |
| DEAH (Asp-Glu-Ala-His) box polypeptide 15 | Dhx15 | 1 | -1.19 | 1.14 | -4.01 | 1 | 1.20 | -1.48 | -1.35 | 1 | 1.38 | -1.07 | -1.32 | 1 | 1.45 | 1.01 | -1.32 | -1.21 | NM\_007839.1 |
| uridine-cytidine kinase 2 | Uck2 | 1 | -1.16 | -1.61 | -3.99 | 1 | -1.11 | 1.14 | -1.25 | 1 | -1.04 | -1.26 | -1.94 | 1 | 1.07 | -1.03 | -1.61 | 1.05 | NM\_030724.1 |
| secreted phosphoprotein 1 | Spp1 | 1 | 1.23 | 1.12 | -3.98 | 1 | 1.10 | -1.81 | -1.06 | 1 | -1.16 | -2.35 | -1.68 | 1 | 1.16 | -1.41 | -1.31 | -1.45 | NM\_009263.1 |
| peripheral myelin protein | Pmp22 | 1 | -1.10 | -1.20 | -3.95 | 1 | -1.26 | -1.46 | -1.38 | 1 | -1.37 | -1.25 | 1.04 | 1 | 1.25 | 1.03 | 1.43 | -1.20 | NM\_008885.1 |
| hydroxysteroid (17-beta) dehydrogenase 7 | Hsd17b7 | 1 | -1.44 | -1.99 | -3.90 | 1 | -2.18 | -2.41 | -2.57 | 1 | 2.05 | -1.50 | -2.20 | 1 | 1.99 | -1.55 | -2.03 | 1.58 | NM\_010476.2 |
| fatty acid synthase | Fasn | 1 | -1.33 | -1.33 | -3.84 | 1 | -1.14 | -1.32 | -1.09 | 1 | 1.26 | -1.21 | -1.56 | 1 | 1.09 | -1.26 | -1.60 | 1.52 | NM\_007988.1 |
| cyclin B1 | Ccnb1 | 1 | -1.26 | -1.82 | -3.75 | 1 | -1.35 | -1.86 | 1.00 | 1 | -2.03 | -1.18 | -1.57 | 1 | -1.70 | -1.17 | -1.97 | 1.06 | NM\_172301.2 |
| SEC5-like 1 (S. cerevisiae) | Sec5l1 | 1 | 1.11 | 1.09 | -3.74 | 1 | 1.12 | -1.07 | 1.12 | 1 | 1.03 | 1.10 | 1.21 | 1 | -1.07 | -1.12 | 1.07 | -1.06 | NM\_025588.2 |
| actin, gamma 2, smooth muscle, enteric | Actg2 | 1 | -1.10 | -1.36 | -3.67 | 1 | -1.64 | -2.39 | -2.76 | 1 | 1.54 | -1.11 | -1.78 | 1 | -1.20 | -1.05 | -1.44 | -1.85 | NM\_009610.1 |
| S100 calcium binding protein A4 | S100a4 | 1 | 1.11 | 1.01 | -3.65 | 1 | -1.06 | -1.50 | -1.10 | 1 | -1.28 | -1.47 | -1.37 | 1 | -1.16 | -1.22 | -1.33 | 1.50 | NM\_011311.1 |
| cyclin B1 | Ccnb1 | 1 | -1.18 | -1.81 | -3.65 | 1 | -1.36 | -2.12 | -1.02 | 1 | -2.10 | -1.18 | -1.55 | 1 | -1.51 | -1.24 | -2.00 | 1.11 | NM\_172301.2 |
| nucleoporin 37 | Nup37 | 1 | -1.23 | -1.50 | -3.65 | 1 | -1.30 | -1.37 | -1.34 | 1 | -1.48 | -1.40 | -1.72 | 1 | -1.46 | -1.43 | -1.81 | 1.07 | NM\_028334.1 |
| trophinin associated protein | Troap | 1 | -1.66 | -3.63 | -1.31 | 1 | -2.38 | -1.03 | 1.27 | 1 | -2.28 | 1.09 | -1.64 | 1 | -1.84 | -1.41 | -2.85 | -1.08 | XM\_283286.2 |
| DNA segment, Chr 4, Brigham & Womens Genetics 0951 expressed | D4Bwg0951e | 1 | -1.26 | -3.13 | -3.60 | 1 | -3.51 | -1.91 | -1.03 | 1 | 1.31 | -1.12 | -1.03 | 1 | -1.08 | 1.00 | -1.22 | 1.56 | NM\_026821.2 |
| transcription elongation factor B (SIII), polypeptide 2 | Tceb2 | 1 | 1.03 | 1.13 | -3.58 | 1 | 1.08 | -1.25 | 1.00 | 1 | -1.06 | -1.18 | -1.11 | 1 | -1.01 | -1.06 | -1.04 | 1.02 | NM\_026305.1 |
| lysyl oxidase | Lox | 1 | -1.05 | -1.05 | -3.58 | 1 | -1.33 | -2.11 | -2.08 | 1 | 1.31 | -1.86 | -1.55 | 1 | 1.17 | -1.01 | 1.09 | 1.95 | NM\_010728.1 |
| cofactor required for Sp1 transcriptional activation, subunit 9 | Crsp9 | 1 | -1.30 | -1.02 | -3.56 | 1 | 1.08 | -1.02 | -1.22 | 1 | 1.16 | 1.19 | 1.10 | 1 | 1.21 | 1.26 | 1.05 | -1.14 | NM\_025426.2 |
| eukaryotic translation elongation factor 1 beta 2 | Eef1b2 | 1 | -1.05 | 1.05 | -3.55 | 1 | -1.01 | -1.31 | -1.04 | 1 | -1.08 | -1.08 | -1.10 | 1 | 1.09 | 1.10 | 1.02 | 1.04 | NM\_018796.2 |
| actin, alpha 2, smooth muscle, aorta | Acta2 | 1 | -1.00 | -1.29 | -3.54 | 1 | -1.25 | -2.32 | -2.79 | 1 | 1.15 | -1.19 | -1.40 | 1 | -1.15 | -1.02 | -1.28 | -1.16 | NM\_183274 |
| lysosomal membrane glycoprotein 2 | Lamp2 | 1 | 1.04 | 1.07 | -3.52 | 1 | 1.01 | -1.37 | -1.08 | 1 | 1.20 | 1.40 | 1.59 | 1 | 1.48 | 1.31 | 1.43 | 1.08 | NM\_010685.2 |
| protein kinase, lysine deficient 1 | Prkwnk1 | 1 | -3.50 | -1.62 | -1.09 | 1 | -1.61 | -1.09 | -1.03 | 1 | -1.42 | -1.13 | -1.04 | 1 | -1.60 | -1.32 | -1.18 | -1.02 | NM\_198703.1 |
| S100 calcium binding protein A10 (calpactin) | S100a10 | 1 | 1.03 | -1.00 | -3.50 | 1 | 1.04 | -1.74 | -1.00 | 1 | -1.41 | -1.24 | -1.04 | 1 | -1.01 | -1.05 | -1.10 | 1.78 | NM\_009112.1 |
| RIKEN cDNA 2300008B03 gene | 2300008B03Rik | 1 | -1.26 | -3.49 | 1.04 | 1 | -3.27 | 1.08 | -1.03 | 1 | -1.77 | 1.24 | 1.29 | 1 | -1.35 | 1.49 | 1.30 | 1.05 | NM\_175106.2 |
| RIKEN cDNA 6720458F09 gene | 6720458F09Rik | 1 | -1.64 | -1.73 | -2.40 | 1 | -2.91 | -1.46 | -3.40 | 1 | 1.59 | 1.12 | -1.67 | 1 | 1.24 | 1.04 | -1.99 | 1.18 | NM\_177374.2 |

## Slide 3
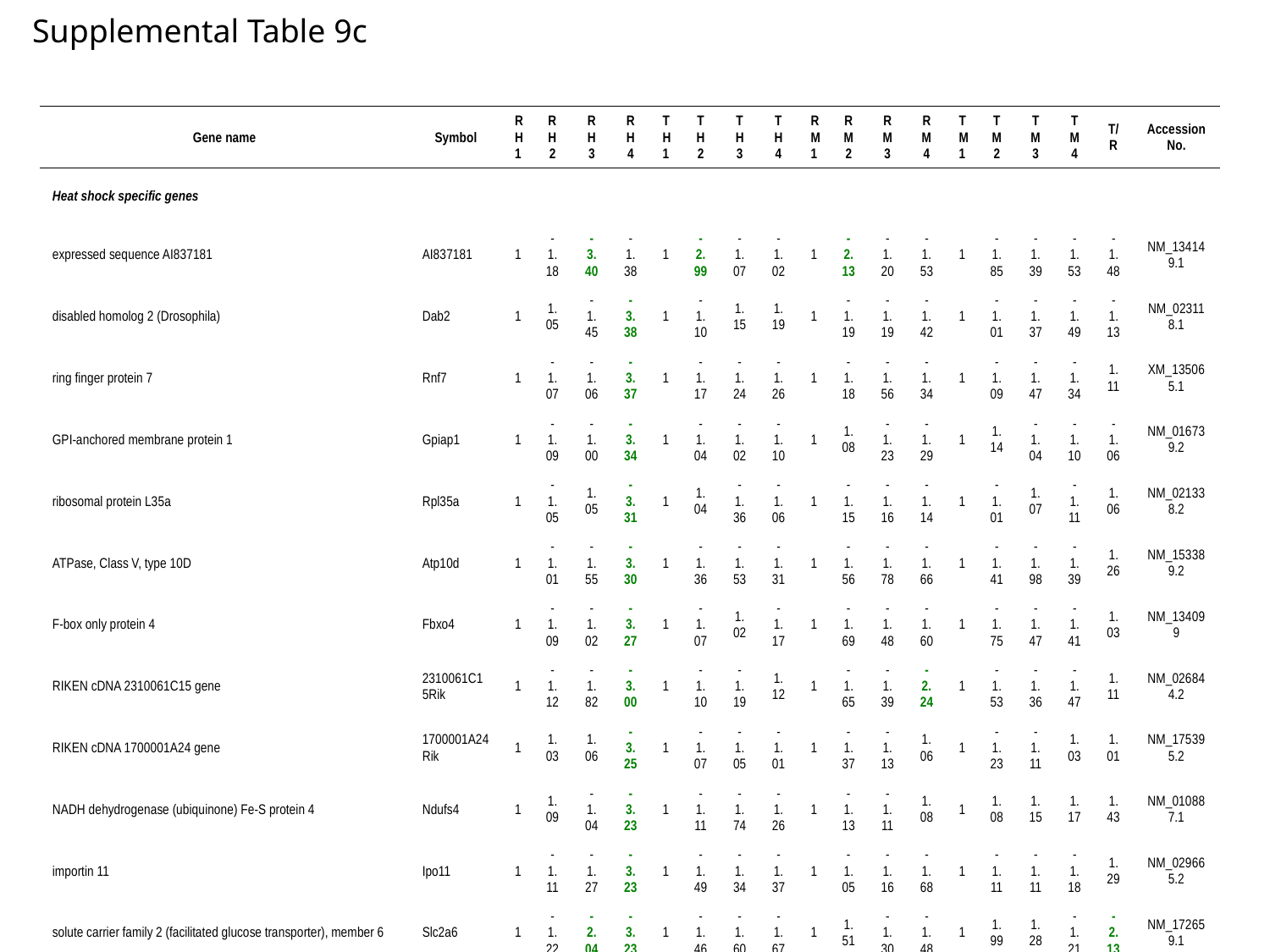

Supplemental Table 9c
| Gene name | Symbol | RH1 | RH2 | RH3 | RH4 | TH1 | TH2 | TH3 | TH4 | RM1 | RM2 | RM3 | RM4 | TM1 | TM2 | TM3 | TM4 | T/R | Accession No. |
| --- | --- | --- | --- | --- | --- | --- | --- | --- | --- | --- | --- | --- | --- | --- | --- | --- | --- | --- | --- |
| Heat shock specific genes | | | | | | | | | | | | | | | | | | | |
| expressed sequence AI837181 | AI837181 | 1 | -1.18 | -3.40 | -1.38 | 1 | -2.99 | -1.07 | -1.02 | 1 | -2.13 | -1.20 | -1.53 | 1 | -1.85 | -1.39 | -1.53 | -1.48 | NM\_134149.1 |
| disabled homolog 2 (Drosophila) | Dab2 | 1 | 1.05 | -1.45 | -3.38 | 1 | -1.10 | 1.15 | 1.19 | 1 | -1.19 | -1.19 | -1.42 | 1 | -1.01 | -1.37 | -1.49 | -1.13 | NM\_023118.1 |
| ring finger protein 7 | Rnf7 | 1 | -1.07 | -1.06 | -3.37 | 1 | -1.17 | -1.24 | -1.26 | 1 | -1.18 | -1.56 | -1.34 | 1 | -1.09 | -1.47 | -1.34 | 1.11 | XM\_135065.1 |
| GPI-anchored membrane protein 1 | Gpiap1 | 1 | -1.09 | -1.00 | -3.34 | 1 | -1.04 | -1.02 | -1.10 | 1 | 1.08 | -1.23 | -1.29 | 1 | 1.14 | -1.04 | -1.10 | -1.06 | NM\_016739.2 |
| ribosomal protein L35a | Rpl35a | 1 | -1.05 | 1.05 | -3.31 | 1 | 1.04 | -1.36 | -1.06 | 1 | -1.15 | -1.16 | -1.14 | 1 | -1.01 | 1.07 | -1.11 | 1.06 | NM\_021338.2 |
| ATPase, Class V, type 10D | Atp10d | 1 | -1.01 | -1.55 | -3.30 | 1 | -1.36 | -1.53 | -1.31 | 1 | -1.56 | -1.78 | -1.66 | 1 | -1.41 | -1.98 | -1.39 | 1.26 | NM\_153389.2 |
| F-box only protein 4 | Fbxo4 | 1 | -1.09 | -1.02 | -3.27 | 1 | -1.07 | 1.02 | -1.17 | 1 | -1.69 | -1.48 | -1.60 | 1 | -1.75 | -1.47 | -1.41 | 1.03 | NM\_134099 |
| RIKEN cDNA 2310061C15 gene | 2310061C15Rik | 1 | -1.12 | -1.82 | -3.00 | 1 | -1.10 | -1.19 | 1.12 | 1 | -1.65 | -1.39 | -2.24 | 1 | -1.53 | -1.36 | -1.47 | 1.11 | NM\_026844.2 |
| RIKEN cDNA 1700001A24 gene | 1700001A24Rik | 1 | 1.03 | 1.06 | -3.25 | 1 | -1.07 | -1.05 | -1.01 | 1 | -1.37 | -1.13 | 1.06 | 1 | -1.23 | -1.11 | 1.03 | 1.01 | NM\_175395.2 |
| NADH dehydrogenase (ubiquinone) Fe-S protein 4 | Ndufs4 | 1 | 1.09 | -1.04 | -3.23 | 1 | -1.11 | -1.74 | -1.26 | 1 | -1.13 | -1.11 | 1.08 | 1 | 1.08 | 1.15 | 1.17 | 1.43 | NM\_010887.1 |
| importin 11 | Ipo11 | 1 | -1.11 | -1.27 | -3.23 | 1 | -1.49 | -1.34 | -1.37 | 1 | -1.05 | -1.16 | -1.68 | 1 | -1.11 | -1.11 | -1.18 | 1.29 | NM\_029665.2 |
| solute carrier family 2 (facilitated glucose transporter), member 6 | Slc2a6 | 1 | -1.22 | -2.04 | -3.23 | 1 | -1.46 | -1.60 | -1.67 | 1 | 1.51 | -1.30 | -1.48 | 1 | 1.99 | 1.28 | -1.21 | -2.13 | NM\_172659.1 |
| transmembrane protein 109 | Tmem109 | 1 | 1.05 | -1.02 | -3.23 | 1 | 1.00 | -1.43 | -1.19 | 1 | -1.13 | -1.40 | -1.77 | 1 | 1.07 | -1.22 | -1.54 | 1.07 | NM\_134142.1 |
| cytochrome P450, 51 | Cyp51 | 1 | 1.05 | -1.48 | -3.22 | 1 | -1.31 | -2.07 | -1.89 | 1 | 2.73 | -1.17 | -2.16 | 1 | 2.37 | -1.16 | -2.34 | 1.27 | NM\_020010.1 |
| chaperonin subunit 8 (theta) | Cct8 | 1 | -1.10 | 1.15 | -3.19 | 1 | 1.09 | -1.26 | -1.29 | 1 | 1.24 | 1.27 | 1.12 | 1 | 1.07 | 1.25 | 1.08 | 1.30 | NM\_009840.2 |
| RIKEN cDNA 2610018I03 gene | 2610018I03Rik | 1 | -1.42 | -1.50 | -3.17 | 1 | -1.35 | -1.32 | -2.29 | 1 | 2.44 | 1.39 | -1.60 | 1 | 1.50 | 1.11 | -1.60 | -1.25 | XM\_135023.2 |
| cell division cycle 5-like (S. pombe) | Cdc5l | 1 | 1.04 | -1.10 | -3.16 | 1 | -1.14 | -1.14 | -1.16 | 1 | 1.05 | -1.31 | -1.25 | 1 | 1.13 | -1.04 | -1.16 | -1.20 | NM\_152810.1 |
| transmembrane protein 186 | Tmem186 | 1 | -1.43 | -2.41 | -1.08 | 1 | -3.15 | 1.01 | -1.11 | 1 | -1.62 | -1.05 | 1.08 | 1 | -1.55 | -1.11 | -1.05 | 1.12 | NM\_025708.1 |
| activating transcription factor 4 | Atf4 | 1 | 1.46 | 1.09 | -3.14 | 1 | 1.11 | -1.59 | -1.42 | 1 | 1.41 | 1.50 | 1.13 | 1 | 1.77 | 1.75 | -1.03 | 1.14 | XM\_139474.1 |
| ribosomal protein L6 | Rpl6 | 1 | -1.03 | 1.02 | -3.13 | 1 | 1.06 | -1.23 | 1.10 | 1 | -1.10 | -1.21 | -1.08 | 1 | 1.23 | 1.11 | -1.07 | -1.19 | NM\_011290 |
| zinc finger protein 672 | Zfp672 | 1 | -1.63 | -2.74 | -3.12 | 1 | -1.37 | -1.28 | -1.49 | 1 | 1.14 | -1.65 | -1.36 | 1 | 1.28 | -1.16 | 1.20 | 1.02 | NM\_178761.2 |
| t-complex protein 1 | Tcp1 | 1 | -1.07 | -1.04 | -3.12 | 1 | -1.15 | -1.27 | -1.22 | 1 | -1.04 | -1.09 | -1.28 | 1 | -1.02 | -1.01 | -1.16 | 1.26 | NM\_013686.1 |
| ribosomal protein S9 | Rps9 | 1 | 1.04 | 1.07 | -3.12 | 1 | -1.12 | -1.68 | -1.16 | 1 | 1.01 | -1.16 | 1.02 | 1 | -1.03 | 1.02 | -1.07 | 1.11 | NM\_029767.1 |
| poly A binding protein, cytoplasmic 1 | Pabpc1 | 1 | 1.09 | 1.16 | -3.11 | 1 | 1.11 | -1.45 | 1.04 | 1 | -1.07 | -1.11 | -1.13 | 1 | 1.05 | -1.07 | -1.17 | -1.13 | NM\_008774.2 |
| Tetraspanin 14 | Tspan14 | 1 | -1.13 | -1.08 | -3.10 | 1 | -1.36 | -1.04 | -1.07 | 1 | -1.35 | -1.34 | -1.04 | 1 | -1.28 | -1.59 | -1.36 | -1.07 | NM\_145928 |
| centromere protein O | Cenpo | 1 | -1.21 | -3.09 | -1.08 | 1 | -2.44 | -1.11 | -1.18 | 1 | -1.14 | 1.07 | -1.37 | 1 | 1.03 | 1.05 | -1.17 | -1.01 | NM\_134046.3 |
| transmembrane protein 15 | Tmem15 | 1 | -1.79 | -3.06 | -2.24 | 1 | -2.53 | -1.07 | -1.47 | 1 | -1.97 | 1.00 | 1.04 | 1 | -1.30 | 1.15 | 1.31 | 1.24 | NM\_177648.2 |
| sarcolemma associated protein | Slmap | 1 | 1.12 | -1.11 | -3.05 | 1 | -1.15 | -1.05 | 1.07 | 1 | -1.38 | 1.11 | 1.16 | 1 | -1.27 | 1.19 | 1.27 | 1.02 | NM\_032008.2 |
| latent transforming growth factor beta binding protein 1 | Ltbp1 | 1 | 1.05 | 1.02 | -3.05 | 1 | -1.06 | -2.55 | -1.37 | 1 | 1.14 | -2.05 | -1.99 | 1 | 1.13 | -1.24 | -1.27 | -1.43 | NM\_019919.2 |
| gene rich cluster, C2f gene | Grcc2f | 1 | -1.10 | -1.33 | -3.03 | 1 | -1.10 | -1.10 | -1.32 | 1 | -1.10 | -1.23 | -1.37 | 1 | -1.09 | -1.05 | -1.26 | 1.05 | NM\_013536.1 |
| RIKEN cDNA 2310002L13 gene | 2310002L13Rik | 1 | -1.78 | -3.26 | -1.17 | 1 | -2.18 | 1.22 | -1.25 | 1 | -1.81 | -1.24 | 1.52 | 1 | -2.20 | -1.23 | -1.13 | 1.15 | XM\_128949.1 |

## Slide 4
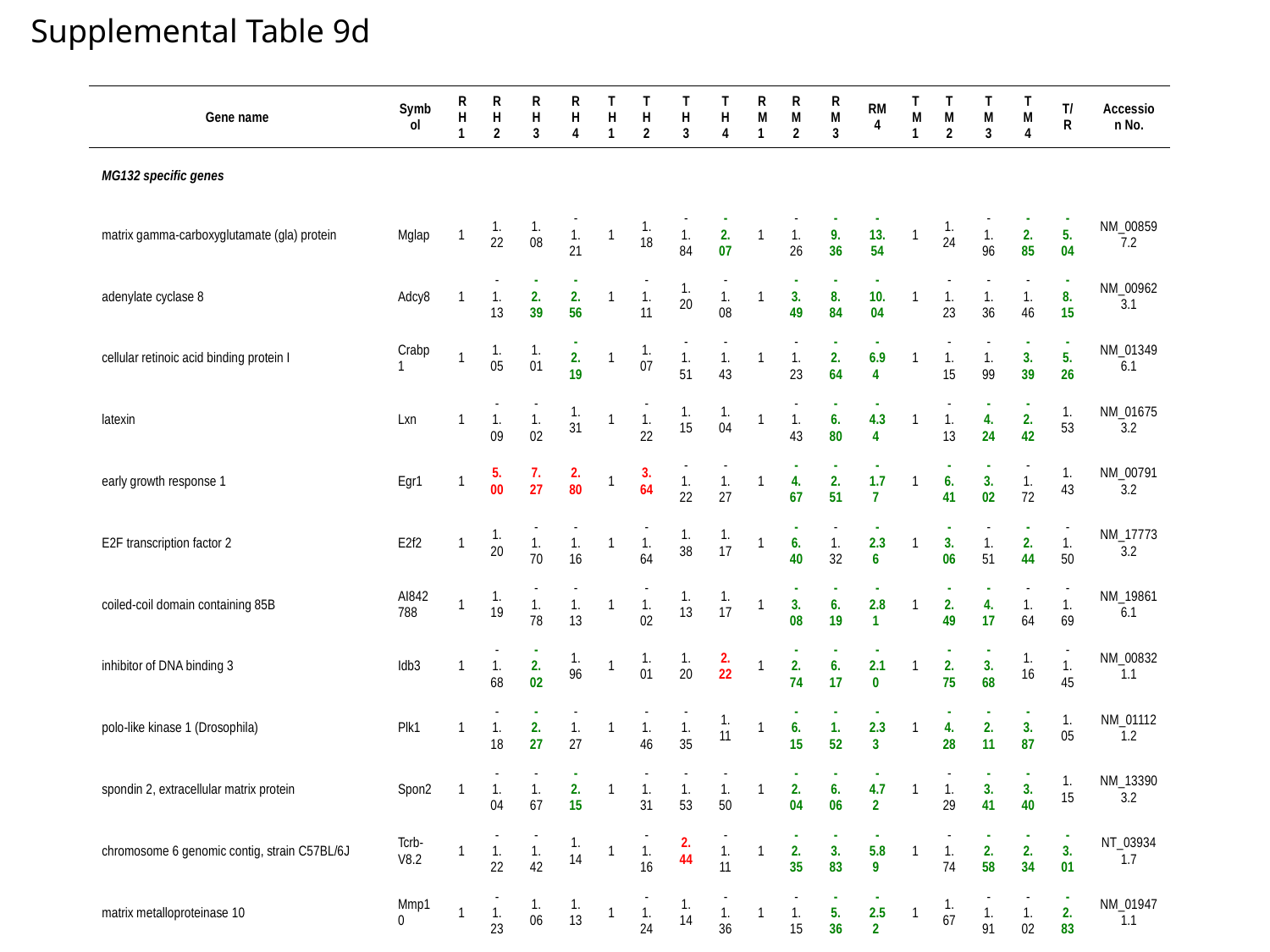

Supplemental Table 9d
| Gene name | Symbol | RH1 | RH2 | RH3 | RH4 | TH1 | TH2 | TH3 | TH4 | RM1 | RM2 | RM3 | RM4 | TM1 | TM2 | TM3 | TM4 | T/R | Accession No. |
| --- | --- | --- | --- | --- | --- | --- | --- | --- | --- | --- | --- | --- | --- | --- | --- | --- | --- | --- | --- |
| MG132 specific genes | | | | | | | | | | | | | | | | | | | |
| matrix gamma-carboxyglutamate (gla) protein | Mglap | 1 | 1.22 | 1.08 | -1.21 | 1 | 1.18 | -1.84 | -2.07 | 1 | -1.26 | -9.36 | -13.54 | 1 | 1.24 | -1.96 | -2.85 | -5.04 | NM\_008597.2 |
| adenylate cyclase 8 | Adcy8 | 1 | -1.13 | -2.39 | -2.56 | 1 | -1.11 | 1.20 | -1.08 | 1 | -3.49 | -8.84 | -10.04 | 1 | -1.23 | -1.36 | -1.46 | -8.15 | NM\_009623.1 |
| cellular retinoic acid binding protein I | Crabp1 | 1 | 1.05 | 1.01 | -2.19 | 1 | 1.07 | -1.51 | -1.43 | 1 | -1.23 | -2.64 | -6.94 | 1 | -1.15 | -1.99 | -3.39 | -5.26 | NM\_013496.1 |
| latexin | Lxn | 1 | -1.09 | -1.02 | 1.31 | 1 | -1.22 | 1.15 | 1.04 | 1 | -1.43 | -6.80 | -4.34 | 1 | -1.13 | -4.24 | -2.42 | 1.53 | NM\_016753.2 |
| early growth response 1 | Egr1 | 1 | 5.00 | 7.27 | 2.80 | 1 | 3.64 | -1.22 | -1.27 | 1 | -4.67 | -2.51 | -1.77 | 1 | -6.41 | -3.02 | -1.72 | 1.43 | NM\_007913.2 |
| E2F transcription factor 2 | E2f2 | 1 | 1.20 | -1.70 | -1.16 | 1 | -1.64 | 1.38 | 1.17 | 1 | -6.40 | -1.32 | -2.36 | 1 | -3.06 | -1.51 | -2.44 | -1.50 | NM\_177733.2 |
| coiled-coil domain containing 85B | AI842788 | 1 | 1.19 | -1.78 | -1.13 | 1 | -1.02 | 1.13 | 1.17 | 1 | -3.08 | -6.19 | -2.81 | 1 | -2.49 | -4.17 | -1.64 | -1.69 | NM\_198616.1 |
| inhibitor of DNA binding 3 | Idb3 | 1 | -1.68 | -2.02 | 1.96 | 1 | 1.01 | 1.20 | 2.22 | 1 | -2.74 | -6.17 | -2.10 | 1 | -2.75 | -3.68 | 1.16 | -1.45 | NM\_008321.1 |
| polo-like kinase 1 (Drosophila) | Plk1 | 1 | -1.18 | -2.27 | -1.27 | 1 | -1.46 | -1.35 | 1.11 | 1 | -6.15 | -1.52 | -2.33 | 1 | -4.28 | -2.11 | -3.87 | 1.05 | NM\_011121.2 |
| spondin 2, extracellular matrix protein | Spon2 | 1 | -1.04 | -1.67 | -2.15 | 1 | -1.31 | -1.53 | -1.50 | 1 | -2.04 | -6.06 | -4.72 | 1 | -1.29 | -3.41 | -3.40 | 1.15 | NM\_133903.2 |
| chromosome 6 genomic contig, strain C57BL/6J | Tcrb-V8.2 | 1 | -1.22 | -1.42 | 1.14 | 1 | -1.16 | 2.44 | -1.11 | 1 | -2.35 | -3.83 | -5.89 | 1 | -1.74 | -2.58 | -2.34 | -3.01 | NT\_039341.7 |
| matrix metalloproteinase 10 | Mmp10 | 1 | -1.23 | 1.06 | 1.13 | 1 | -1.24 | 1.14 | -1.36 | 1 | -1.15 | -5.36 | -2.52 | 1 | 1.67 | -1.91 | -1.02 | -2.83 | NM\_019471.1 |
| progressive ankylosis | Ank | 1 | -1.12 | -1.40 | -1.53 | 1 | -1.34 | 1.32 | -1.26 | 1 | -1.87 | -5.23 | -4.06 | 1 | -1.41 | -3.12 | -2.31 | -2.19 | NM\_020332.2 |
| procollagen, type I, alpha 1 | Col1a1 | 1 | 1.17 | 1.00 | -2.35 | 1 | 1.27 | -1.02 | -1.03 | 1 | -1.23 | -2.58 | -5.19 | 1 | -1.44 | -2.76 | -3.62 | -1.45 | NM\_007742.2 |
| expressed sequence AI415330 | AI415330 | 1 | 1.02 | -1.58 | -1.01 | 1 | -1.43 | -1.05 | -1.12 | 1 | -5.19 | -2.49 | -1.23 | 1 | -4.30 | -1.75 | 1.09 | 1.79 | NM\_178114.2 |
| chemokine (C-X3-C motif) ligand 1 | Cx3cl1 | 1 | -1.32 | -1.40 | -1.88 | 1 | -1.25 | -1.47 | -1.46 | 1 | -1.05 | -5.04 | -3.91 | 1 | 1.09 | -3.03 | -2.75 | -1.85 | NM\_009142.2 |
| homeo box, msh-like 3 | Msx3 | 1 | -1.26 | -2.11 | -1.16 | 1 | -1.63 | 1.41 | 1.12 | 1 | -4.99 | -3.83 | -2.11 | 1 | -2.39 | -2.73 | -1.60 | 1.08 | NM\_010836 |
| plasminogen activator, urokinase | Plau | 1 | -1.38 | -1.75 | 1.00 | 1 | -2.00 | -1.12 | -1.92 | 1 | -1.03 | -4.56 | -4.83 | 1 | 2.12 | -4.01 | -3.13 | 1.05 | NM\_008873.2 |
| inhibitor of DNA binding 2 | Idb2 | 1 | -1.48 | -1.53 | -1.07 | 1 | 1.15 | -1.26 | 1.22 | 1 | -2.38 | -4.82 | -2.10 | 1 | -1.88 | -2.64 | 1.26 | -1.24 | NM\_010496.2 |
| thymidine kinase 1 | Tk1 | 1 | -1.05 | -2.89 | -1.16 | 1 | -1.51 | -1.18 | -1.01 | 1 | -4.80 | -3.27 | -4.41 | 1 | -2.73 | -3.34 | -2.89 | 1.74 | NM\_009387.1 |
| vascular cell adhesion molecule 1 | Vcam1 | 1 | -1.04 | -1.60 | -1.32 | 1 | -1.69 | -1.33 | -1.14 | 1 | -1.48 | -4.63 | -3.09 | 1 | -1.16 | -3.43 | -1.95 | 1.64 | NM\_011693.2 |
| thymidine kinase 1 | Tk1 | 1 | -1.05 | -2.64 | 1.20 | 1 | -1.27 | 1.02 | 1.22 | 1 | -4.21 | -3.30 | -4.62 | 1 | -2.73 | -3.34 | -3.24 | 1.54 | NM\_009387 |
| immediate early response 5-like | Ier5l | 1 | 1.15 | -1.82 | -2.01 | 1 | 1.11 | -2.18 | 1.20 | 1 | -1.82 | -4.58 | -2.67 | 1 | -1.10 | -3.31 | -1.61 | -1.39 | NM\_030244 |
| histone 1, H2ai | Hist1h2ai | 1 | -1.94 | -2.82 | 1.03 | 1 | -2.00 | -1.04 | -1.07 | 1 | -2.79 | -2.18 | -4.57 | 1 | -1.20 | -2.68 | -3.39 | 1.03 | NM\_178182 |
| cell division cycle associated 7 | Cdca7 | 1 | -1.00 | -1.59 | -1.38 | 1 | -1.85 | -1.10 | -1.32 | 1 | -1.97 | -3.54 | -4.50 | 1 | -1.80 | -2.96 | -2.62 | 1.13 | NM\_025866.1 |
| sema domain, immunoglobulin domain (Ig), short basic domain, secreted, (semaphorin) 3A | Sema3a | 1 | 1.13 | -1.49 | -2.70 | 1 | -1.75 | -1.57 | -1.42 | 1 | -2.03 | -4.49 | -2.79 | 1 | -1.70 | -2.68 | -1.54 | 1.17 | NM\_009152.2 |
| histone 1, H2bc | Hist1h2bc | 1 | -1.08 | 1.70 | -1.18 | 1 | 1.26 | 1.65 | -1.01 | 1 | -2.55 | -4.42 | -1.35 | 1 | -1.42 | -1.25 | 1.20 | 1.53 | NM\_023422 |
| cell division cycle 6 homolog (S. cerevisiae) | Cdc6 | 1 | -1.17 | -2.25 | -1.25 | 1 | -1.67 | 1.10 | -1.19 | 1 | -2.01 | -3.17 | -4.40 | 1 | -1.15 | -3.03 | -2.61 | 1.54 | NM\_011799.1 |
| Notch-regulated ankyrin repeat protein | Nrarp | 1 | 1.03 | -1.16 | -1.36 | 1 | 1.07 | 1.68 | -1.02 | 1 | -2.66 | -4.38 | -3.61 | 1 | -2.10 | -2.87 | -2.23 | -2.89 | NM\_025980.1 |
| cysteine and glycine-rich protein 2 | Csrp2 | 1 | -1.23 | -1.06 | -1.26 | 1 | 1.06 | 1.17 | 1.01 | 1 | -1.43 | -4.38 | -3.55 | 1 | -1.36 | -3.61 | -2.38 | -1.14 | NM\_007792.2 |
| promyelocytic leukemia | Pml | 1 | 1.03 | -1.34 | 1.44 | 1 | 1.15 | 1.50 | 2.06 | 1 | -4.28 | 1.90 | 2.09 | 1 | -3.26 | -1.12 | 1.13 | -1.13 | NM\_008884.2 |
| ribonucleotide reductase M2 | Rrm2 | 1 | -1.19 | -1.38 | 1.03 | 1 | -1.44 | -1.17 | -1.31 | 1 | -1.66 | -3.09 | -4.26 | 1 | -1.36 | -2.75 | -2.78 | 1.31 | NM\_009104.1 |
| cDNA sequence BC060615 | BC060615 | 1 | -1.21 | -1.62 | -1.41 | 1 | -1.54 | -1.24 | 1.18 | 1 | -4.22 | -2.75 | -2.12 | 1 | -3.51 | -3.13 | -2.17 | -1.23 | NM\_198423.1 |
| kinesin family member 2C | Kif2c | 1 | -1.00 | -1.92 | -1.08 | 1 | -1.28 | -1.31 | 1.44 | 1 | -4.20 | 1.01 | -1.75 | 1 | -2.84 | -1.36 | -2.44 | 1.01 | NM\_134471.2 |
| regulator of G-protein signaling 16 | Rgs16 | 1 | -1.11 | 1.53 | -1.67 | 1 | 1.69 | 1.67 | -1.25 | 1 | 2.33 | -2.82 | -3.33 | 1 | 1.19 | -2.74 | -4.20 | -1.13 | NM\_011267.1 |
| procollagen, type VI, alpha 1 | Col6a1 | 1 | 1.10 | -1.09 | -1.78 | 1 | 1.03 | -1.23 | -1.06 | 1 | -1.52 | -2.93 | -4.19 | 1 | -1.17 | -1.90 | -2.35 | -1.74 | NM\_009933.1 |
| histone 1, H3e | Hist1h3e | 1 | 1.34 | 1.58 | 1.07 | 1 | 1.40 | 1.02 | 1.10 | 1 | -1.21 | -1.94 | -4.19 | 1 | -1.11 | -1.62 | -2.89 | 1.00 | NM\_178205 |
| catenin alpha-like 1 | Catnal1 | 1 | 1.06 | -1.19 | -1.12 | 1 | 1.11 | -1.14 | 1.33 | 1 | -2.07 | -4.18 | -1.72 | 1 | -1.52 | -3.30 | -1.10 | -1.31 | NM\_018761.2 |
| histone 1, H3b | Hist1h3b | 1 | 1.38 | 1.56 | 1.08 | 1 | 1.46 | 1.01 | 1.12 | 1 | -1.17 | -1.97 | -4.18 | 1 | -1.13 | -1.72 | -2.83 | 1.01 | NM\_178203 |
| arylsulfatase J | Arsj | 1 | -1.43 | -2.65 | -1.99 | 1 | -2.42 | -1.08 | -1.98 | 1 | -3.87 | -2.89 | -4.17 | 1 | -2.49 | -2.36 | -3.52 | 1.02 | NM\_173451.1 |
| ELOVL family member 6, elongation of long chain fatty acids (yeast) | Elovl6 | 1 | 1.08 | -1.25 | -2.20 | 1 | -1.32 | -1.81 | -1.67 | 1 | -1.40 | -3.03 | -3.11 | 1 | -1.59 | -4.16 | -3.65 | 1.27 | NM\_130450.1 |
| helicase (DNA) B | Helb | 1 | 1.20 | -1.31 | 1.64 | 1 | -1.08 | 1.09 | 1.49 | 1 | -4.15 | -1.49 | 1.01 | 1 | -2.05 | -1.26 | 1.38 | 1.08 | NM\_080446.1 |

## Slide 5
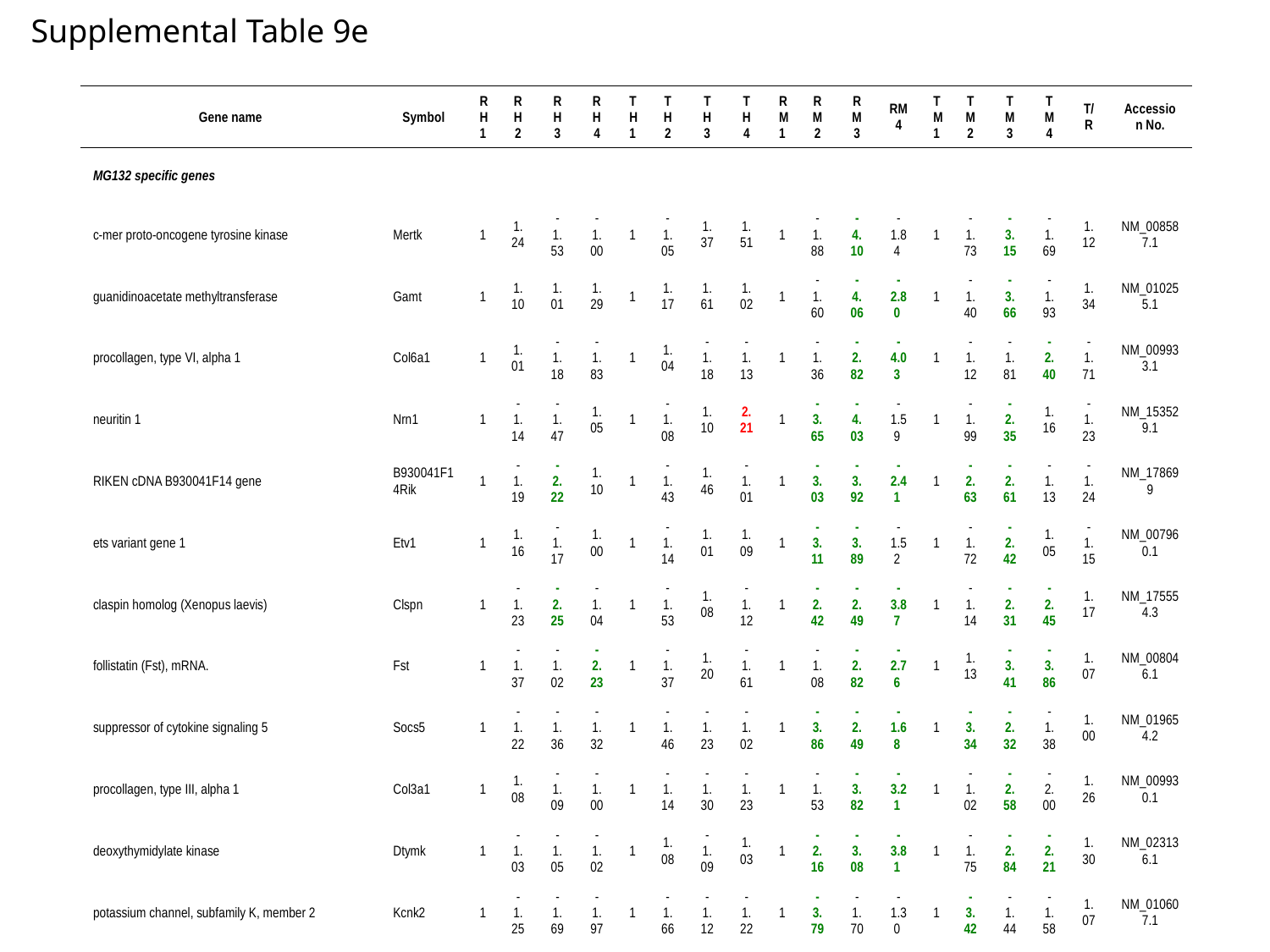

Supplemental Table 9e
| Gene name | Symbol | RH1 | RH2 | RH3 | RH4 | TH1 | TH2 | TH3 | TH4 | RM1 | RM2 | RM3 | RM4 | TM1 | TM2 | TM3 | TM4 | T/R | Accession No. |
| --- | --- | --- | --- | --- | --- | --- | --- | --- | --- | --- | --- | --- | --- | --- | --- | --- | --- | --- | --- |
| MG132 specific genes | | | | | | | | | | | | | | | | | | | |
| c-mer proto-oncogene tyrosine kinase | Mertk | 1 | 1.24 | -1.53 | -1.00 | 1 | -1.05 | 1.37 | 1.51 | 1 | -1.88 | -4.10 | -1.84 | 1 | -1.73 | -3.15 | -1.69 | 1.12 | NM\_008587.1 |
| guanidinoacetate methyltransferase | Gamt | 1 | 1.10 | 1.01 | 1.29 | 1 | 1.17 | 1.61 | 1.02 | 1 | -1.60 | -4.06 | -2.80 | 1 | -1.40 | -3.66 | -1.93 | 1.34 | NM\_010255.1 |
| procollagen, type VI, alpha 1 | Col6a1 | 1 | 1.01 | -1.18 | -1.83 | 1 | 1.04 | -1.18 | -1.13 | 1 | -1.36 | -2.82 | -4.03 | 1 | -1.12 | -1.81 | -2.40 | -1.71 | NM\_009933.1 |
| neuritin 1 | Nrn1 | 1 | -1.14 | -1.47 | 1.05 | 1 | -1.08 | 1.10 | 2.21 | 1 | -3.65 | -4.03 | -1.59 | 1 | -1.99 | -2.35 | 1.16 | -1.23 | NM\_153529.1 |
| RIKEN cDNA B930041F14 gene | B930041F14Rik | 1 | -1.19 | -2.22 | 1.10 | 1 | -1.43 | 1.46 | -1.01 | 1 | -3.03 | -3.92 | -2.41 | 1 | -2.63 | -2.61 | -1.13 | -1.24 | NM\_178699 |
| ets variant gene 1 | Etv1 | 1 | 1.16 | -1.17 | 1.00 | 1 | -1.14 | 1.01 | 1.09 | 1 | -3.11 | -3.89 | -1.52 | 1 | -1.72 | -2.42 | 1.05 | -1.15 | NM\_007960.1 |
| claspin homolog (Xenopus laevis) | Clspn | 1 | -1.23 | -2.25 | -1.04 | 1 | -1.53 | 1.08 | -1.12 | 1 | -2.42 | -2.49 | -3.87 | 1 | -1.14 | -2.31 | -2.45 | 1.17 | NM\_175554.3 |
| follistatin (Fst), mRNA. | Fst | 1 | -1.37 | -1.02 | -2.23 | 1 | -1.37 | 1.20 | -1.61 | 1 | -1.08 | -2.82 | -2.76 | 1 | 1.13 | -3.41 | -3.86 | 1.07 | NM\_008046.1 |
| suppressor of cytokine signaling 5 | Socs5 | 1 | -1.22 | -1.36 | -1.32 | 1 | -1.46 | -1.23 | -1.02 | 1 | -3.86 | -2.49 | -1.68 | 1 | -3.34 | -2.32 | -1.38 | 1.00 | NM\_019654.2 |
| procollagen, type III, alpha 1 | Col3a1 | 1 | 1.08 | -1.09 | -1.00 | 1 | -1.14 | -1.30 | -1.23 | 1 | -1.53 | -3.82 | -3.21 | 1 | -1.02 | -2.58 | -2.00 | 1.26 | NM\_009930.1 |
| deoxythymidylate kinase | Dtymk | 1 | -1.03 | -1.05 | -1.02 | 1 | 1.08 | -1.09 | 1.03 | 1 | -2.16 | -3.08 | -3.81 | 1 | -1.75 | -2.84 | -2.21 | 1.30 | NM\_023136.1 |
| potassium channel, subfamily K, member 2 | Kcnk2 | 1 | -1.25 | -1.69 | -1.97 | 1 | -1.66 | -1.12 | -1.22 | 1 | -3.79 | -1.70 | -1.30 | 1 | -3.42 | -1.44 | -1.58 | 1.07 | NM\_010607.1 |
| H2A histone family, member X | H2afx | 1 | -1.31 | -2.82 | -2.30 | 1 | -1.42 | -1.28 | -1.05 | 1 | -1.65 | -3.25 | -3.78 | 1 | -1.83 | -2.42 | -2.46 | -1.19 | NM\_010436.2 |
| EGF-like-domain, multiple 9 | Egfl9 | 1 | -1.05 | -2.05 | -1.58 | 1 | -1.21 | -1.33 | -1.01 | 1 | -2.19 | -3.78 | -2.17 | 1 | -2.46 | -2.64 | -1.45 | 1.57 | NM\_207666.1 |
| RIKEN cDNA 2510015F01 gene | 2510015F01Rik | 1 | 1.09 | -1.20 | -1.50 | 1 | 1.16 | 1.01 | 1.26 | 1 | -1.14 | -2.63 | -3.76 | 1 | -1.15 | -2.11 | -2.93 | -1.96 | XM\_354801.1 |
| baculoviral IAP repeat-containing 5 | Birc5 | 1 | -1.06 | -1.41 | -1.15 | 1 | -1.09 | -1.41 | 1.19 | 1 | -1.72 | -1.92 | -3.73 | 1 | -1.48 | -1.85 | -3.39 | 1.36 | NM\_009689.1 |
| acid phosphatase-like 2 | C130099A20Rik | 1 | -1.03 | -1.53 | 2.01 | 1 | -1.47 | -1.09 | 1.14 | 1 | -2.44 | -3.73 | -2.20 | 1 | -1.67 | -3.45 | -1.20 | 1.41 | NM\_153420.1 |
| Ras and Rab interactor 1 | Rin1 | 1 | 1.11 | -1.66 | -1.00 | 1 | -2.10 | 1.43 | -1.14 | 1 | -3.70 | -1.61 | -1.51 | 1 | -3.59 | -1.45 | -1.79 | -1.13 | NM\_145495.1 |
| angiopoietin-like 4 | Angptl4 | 1 | -1.79 | 2.04 | -1.58 | 1 | -1.61 | -1.35 | -2.14 | 1 | -2.43 | -3.69 | -2.59 | 1 | -2.23 | -3.06 | -2.57 | 1.64 | NM\_020581.1 |
| troponin T2, cardiac | Tnnt2 | 1 | -1.51 | 1.73 | 2.05 | 1 | 2.00 | 2.57 | 1.70 | 1 | -1.40 | -3.52 | -3.69 | 1 | -1.22 | -2.19 | -2.21 | -1.19 | NM\_011619.1 |
| plasminogen activator, tissue | Plat | 1 | -1.14 | 2.27 | 1.10 | 1 | 1.08 | 1.03 | -1.48 | 1 | 1.07 | -3.30 | -3.68 | 1 | -1.41 | -2.39 | -3.56 | -1.27 | NM\_008872 |
| G protein-coupled receptor, family C, group 5, member B | Gprc5b | 1 | 1.31 | -1.19 | -1.08 | 1 | -1.13 | -1.44 | -1.06 | 1 | -1.49 | -2.50 | -1.29 | 1 | -1.03 | -3.68 | -2.32 | -1.72 | NM\_022420.1 |
| cadherin-like 26 | Gm1010 | 1 | 1.06 | -1.18 | -1.35 | 1 | -1.38 | -1.36 | -1.30 | 1 | -1.18 | -3.17 | -3.15 | 1 | -1.31 | -3.67 | -2.47 | 1.84 | NM\_198656.1 |
| procollagen, type V, alpha 1 | Col5a1 | 1 | 1.11 | 1.01 | -1.77 | 1 | -1.19 | -1.58 | -1.43 | 1 | -1.27 | -2.60 | -3.62 | 1 | -1.17 | -1.92 | -2.56 | 1.14 | NM\_015734.1 |
| zinc finger, DHHC domain containing 12 | Zdhhc12 | 1 | -1.20 | -2.59 | 1.15 | 1 | -1.81 | 1.23 | -1.00 | 1 | -3.62 | -1.17 | -1.11 | 1 | -2.32 | -1.11 | 1.10 | -1.37 | NM\_025428.1 |
| phosphatidic acid phosphatase type 2B | Ppap2b | 1 | -1.47 | 2.29 | -1.28 | 1 | -1.19 | -1.12 | -2.16 | 1 | 2.85 | -1.23 | -1.96 | 1 | 1.05 | -2.01 | -3.61 | 2.50 | NM\_080555.1 |
| diphtheria toxin receptor | Dtr | 1 | 1.06 | 1.51 | -1.32 | 1 | 1.51 | 1.01 | -1.57 | 1 | -1.83 | -1.93 | -3.01 | 1 | -3.34 | -2.12 | -3.60 | 1.33 | NM\_010415.1 |
| claspin homolog (Xenopus laevis) | Clspn | 1 | -1.02 | -2.20 | 1.01 | 1 | -1.51 | 1.06 | -1.02 | 1 | -2.29 | -2.54 | -3.60 | 1 | -1.31 | -2.27 | -2.22 | 1.08 | NM\_175554.3 |
| histone1, H3d | Hist1h3d | 1 | 1.33 | 1.53 | 1.09 | 1 | 1.40 | 1.01 | 1.08 | 1 | -1.13 | -1.78 | -3.58 | 1 | -1.14 | -1.65 | -2.82 | 1.07 | NM\_178204.1 |
| cysteine-rich protein 1 (intestinal) | Crip1 | 1 | -1.14 | -1.58 | -1.76 | 1 | 1.03 | -1.45 | 1.11 | 1 | -1.74 | -3.57 | -2.41 | 1 | -1.30 | -2.54 | -2.23 | 1.32 | NM\_007763 |
| follistatin-like 1 | Fstl1 | 1 | 1.10 | -1.55 | -2.57 | 1 | -1.69 | -2.35 | -1.53 | 1 | -1.40 | -2.06 | -3.57 | 1 | -1.38 | -1.72 | -2.87 | -1.08 | NM\_008047.2 |
| paired related homeobox 1 | Prrx1 | 1 | -1.24 | -1.17 | -1.73 | 1 | -1.44 | 1.33 | -1.14 | 1 | -1.43 | -1.48 | -3.16 | 1 | -1.40 | -2.27 | -3.56 | -1.58 | NM\_011127.1 |
| parathyroid hormone-like peptide | Pthlh | 1 | -1.62 | 1.56 | -2.15 | 1 | -1.15 | 1.17 | -1.18 | 1 | -3.22 | -3.03 | -3.54 | 1 | -2.32 | -2.57 | -2.83 | -1.54 | NM\_008970.1 |
| caveolin, caveolae protein 1 | Cav1 | 1 | 1.05 | -1.04 | -1.04 | 1 | -1.12 | -1.40 | -1.01 | 1 | -1.69 | -3.52 | -1.68 | 1 | -1.29 | -2.00 | -1.11 | 2.34 | NM\_007616.2 |
| kinesin family member 23 | Kif23 | 1 | -1.15 | -1.55 | -2.01 | 1 | -1.29 | -1.87 | -1.04 | 1 | -1.62 | -1.78 | -3.20 | 1 | -1.22 | -2.00 | -3.51 | 1.12 | NM\_024245 |
| minichromosome maintenance deficient 5, cell division cycle 46 (S. cerevisiae) | Mcm5 | 1 | -1.04 | -1.56 | -1.10 | 1 | -1.14 | 1.14 | 1.09 | 1 | -1.30 | -1.85 | -3.51 | 1 | -1.12 | -1.83 | -2.23 | 1.09 | NM\_008566.1 |
| limb-bud and heart | Lbh | 1 | 1.15 | -1.19 | 1.11 | 1 | -1.13 | 1.26 | 1.31 | 1 | -3.19 | -3.50 | -1.67 | 1 | -2.91 | -2.79 | -1.51 | -1.50 | NM\_029999.3 |
| nurim (nuclear envelope membrane protein) | Nrm | 1 | 1.15 | -1.10 | 1.11 | 1 | -1.01 | 1.22 | 1.21 | 1 | -3.48 | -2.59 | -3.33 | 1 | -2.77 | -2.46 | -2.29 | 1.11 | NM\_134122.1 |
| RIKEN cDNA 2810417H13 gene | 2810417H13Rik | 1 | 1.02 | -1.17 | 1.16 | 1 | -1.03 | -1.20 | 1.33 | 1 | -1.50 | -2.70 | -3.48 | 1 | -1.33 | -2.41 | -2.36 | 1.36 | NM\_026515.1 |
| glucosaminyl (N-acetyl) transferase 1, core 2 | Gcnt1 | 1 | -1.44 | -1.50 | -1.49 | 1 | -1.54 | -1.30 | -1.15 | 1 | -2.26 | -3.47 | 1.03 | 1 | -1.12 | -2.67 | 1.27 | 1.10 | NM\_173442.1 |

## Slide 6
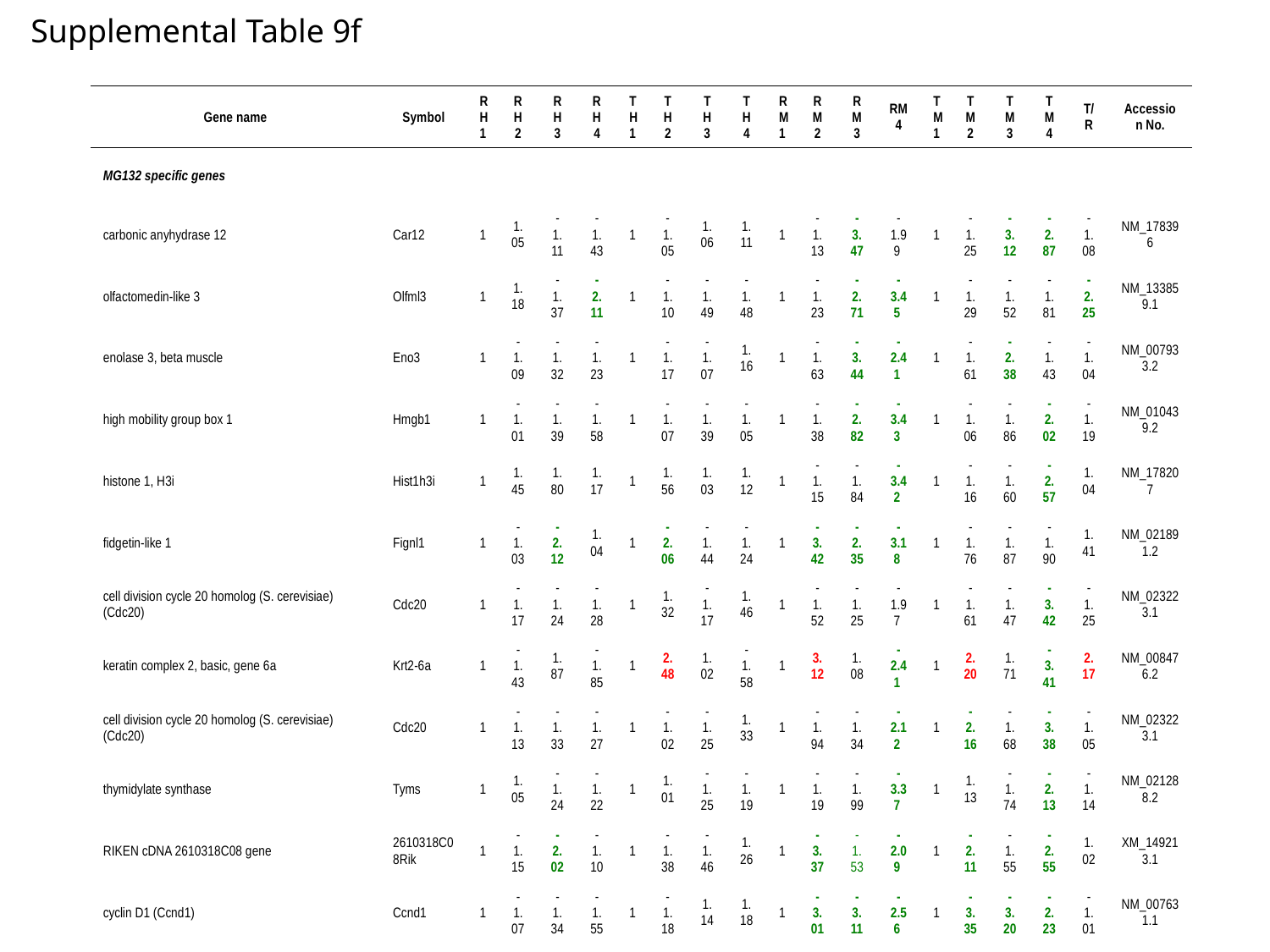

Supplemental Table 9f
| Gene name | Symbol | RH1 | RH2 | RH3 | RH4 | TH1 | TH2 | TH3 | TH4 | RM1 | RM2 | RM3 | RM4 | TM1 | TM2 | TM3 | TM4 | T/R | Accession No. |
| --- | --- | --- | --- | --- | --- | --- | --- | --- | --- | --- | --- | --- | --- | --- | --- | --- | --- | --- | --- |
| MG132 specific genes | | | | | | | | | | | | | | | | | | | |
| carbonic anyhydrase 12 | Car12 | 1 | 1.05 | -1.11 | -1.43 | 1 | -1.05 | 1.06 | 1.11 | 1 | -1.13 | -3.47 | -1.99 | 1 | -1.25 | -3.12 | -2.87 | -1.08 | NM\_178396 |
| olfactomedin-like 3 | Olfml3 | 1 | 1.18 | -1.37 | -2.11 | 1 | -1.10 | -1.49 | -1.48 | 1 | -1.23 | -2.71 | -3.45 | 1 | -1.29 | -1.52 | -1.81 | -2.25 | NM\_133859.1 |
| enolase 3, beta muscle | Eno3 | 1 | -1.09 | -1.32 | -1.23 | 1 | -1.17 | -1.07 | 1.16 | 1 | -1.63 | -3.44 | -2.41 | 1 | -1.61 | -2.38 | -1.43 | -1.04 | NM\_007933.2 |
| high mobility group box 1 | Hmgb1 | 1 | -1.01 | -1.39 | -1.58 | 1 | -1.07 | -1.39 | -1.05 | 1 | -1.38 | -2.82 | -3.43 | 1 | -1.06 | -1.86 | -2.02 | -1.19 | NM\_010439.2 |
| histone 1, H3i | Hist1h3i | 1 | 1.45 | 1.80 | 1.17 | 1 | 1.56 | 1.03 | 1.12 | 1 | -1.15 | -1.84 | -3.42 | 1 | -1.16 | -1.60 | -2.57 | 1.04 | NM\_178207 |
| fidgetin-like 1 | Fignl1 | 1 | -1.03 | -2.12 | 1.04 | 1 | -2.06 | -1.44 | -1.24 | 1 | -3.42 | -2.35 | -3.18 | 1 | -1.76 | -1.87 | -1.90 | 1.41 | NM\_021891.2 |
| cell division cycle 20 homolog (S. cerevisiae) (Cdc20) | Cdc20 | 1 | -1.17 | -1.24 | -1.28 | 1 | 1.32 | -1.17 | 1.46 | 1 | -1.52 | -1.25 | -1.97 | 1 | -1.61 | -1.47 | -3.42 | -1.25 | NM\_023223.1 |
| keratin complex 2, basic, gene 6a | Krt2-6a | 1 | -1.43 | 1.87 | -1.85 | 1 | 2.48 | 1.02 | -1.58 | 1 | 3.12 | 1.08 | -2.41 | 1 | 2.20 | 1.71 | -3.41 | 2.17 | NM\_008476.2 |
| cell division cycle 20 homolog (S. cerevisiae) (Cdc20) | Cdc20 | 1 | -1.13 | -1.33 | -1.27 | 1 | -1.02 | -1.25 | 1.33 | 1 | -1.94 | -1.34 | -2.12 | 1 | -2.16 | -1.68 | -3.38 | -1.05 | NM\_023223.1 |
| thymidylate synthase | Tyms | 1 | 1.05 | -1.24 | -1.22 | 1 | 1.01 | -1.25 | -1.19 | 1 | -1.19 | -1.99 | -3.37 | 1 | 1.13 | -1.74 | -2.13 | -1.14 | NM\_021288.2 |
| RIKEN cDNA 2610318C08 gene | 2610318C08Rik | 1 | -1.15 | -2.02 | -1.10 | 1 | -1.38 | -1.46 | 1.26 | 1 | -3.37 | -1.53 | -2.09 | 1 | -2.11 | -1.55 | -2.55 | 1.02 | XM\_149213.1 |
| cyclin D1 (Ccnd1) | Ccnd1 | 1 | -1.07 | -1.34 | -1.55 | 1 | -1.18 | 1.14 | 1.18 | 1 | -3.01 | -3.11 | -2.56 | 1 | -3.35 | -3.20 | -2.23 | -1.01 | NM\_007631.1 |
| chemokine (C-X-C motif) ligand 12 (Cxcl12), transcript variant 2 | Cxcl12 | 1 | 1.19 | -1.10 | -1.62 | 1 | 1.38 | -1.14 | 1.43 | 1 | -1.69 | -3.34 | -2.06 | 1 | -1.63 | -2.69 | -1.42 | -1.27 | NM\_021704.1 |
| scavenger receptor class A, member 3 | Scara3 | 1 | -1.04 | -1.57 | 1.16 | 1 | -1.47 | 1.03 | 1.10 | 1 | -2.06 | -3.34 | -2.69 | 1 | -1.21 | -2.49 | -1.70 | -3.30 | NM\_172604.1 |
| gap junction membrane channel protein beta 3 | Gjb3 | 1 | -1.68 | 1.04 | -1.20 | 1 | 1.32 | 1.04 | -1.72 | 1 | -1.57 | -2.23 | -2.12 | 1 | -2.01 | -3.33 | -3.00 | 2.30 | NM\_008126.1 |
| ERBB receptor feedback inhibitor 1 | Errfi1 | 1 | -1.09 | -1.14 | -1.13 | 1 | -1.18 | -1.15 | -1.58 | 1 | -3.32 | -2.70 | -2.21 | 1 | -2.03 | -2.11 | -2.03 | -1.42 | NM\_133753.1 |
| protein tyrosine phosphatase, receptor type, V | Ptprv | 1 | -1.05 | 1.23 | -1.56 | 1 | 1.20 | -1.02 | -1.06 | 1 | -1.33 | -3.08 | -3.31 | 1 | -1.35 | -2.18 | -1.65 | -1.30 | NM\_007955.2 |
| heat shock protein 12B | Hspa12b | 1 | 1.18 | -1.75 | 1.21 | 1 | -1.42 | 1.25 | 1.14 | 1 | -3.31 | -1.87 | -1.53 | 1 | -2.28 | -1.56 | -1.27 | 1.38 | NM\_028306.2 |
| baculoviral IAP repeat-containing 5 | Birc5 | 1 | -1.09 | -1.41 | -1.18 | 1 | -1.08 | -1.24 | 1.24 | 1 | -1.78 | -1.78 | -2.94 | 1 | -1.81 | -2.10 | -3.31 | 1.35 | NM\_009689.1 |
| zinc finger protein 41 | Zfp41 | 1 | -1.08 | -1.33 | 1.77 | 1 | -2.19 | 1.12 | 1.06 | 1 | -3.31 | -1.18 | -1.43 | 1 | -1.68 | -1.33 | -1.23 | 1.37 | NM\_011759.1 |
| thymidylate synthase | Tyms | 1 | -1.04 | -1.25 | -2.58 | 1 | -1.09 | -1.37 | -1.21 | 1 | -1.15 | -1.86 | -3.30 | 1 | -1.02 | -2.01 | -2.47 | -1.06 | NM\_021288.2 |
| amphiregulin | Areg | 1 | -1.23 | 1.58 | -1.95 | 1 | 2.73 | 1.12 | -1.32 | 1 | 3.96 | -1.67 | -3.28 | 1 | 7.09 | -1.07 | -2.48 | -4.00 | NM\_009704.2 |
| RIKEN cDNA 2310016C16 gene | 2310016C16Rik | 1 | -1.33 | -1.55 | 1.10 | 1 | -1.18 | 1.16 | 1.25 | 1 | -3.28 | -1.67 | -1.09 | 1 | -2.02 | -1.50 | -1.16 | 1.17 | NM\_027127.1 |
| distal-less homeobox 2 | Dlx2 | 1 | -2.01 | -1.04 | -1.16 | 1 | 2.40 | 1.30 | 1.36 | 1 | -3.08 | -3.27 | -3.04 | 1 | -2.75 | -2.96 | -2.16 | -1.72 | NM\_010054.1 |
| baculoviral IAP repeat-containing 5 | Birc5 | 1 | -1.06 | -1.43 | -1.27 | 1 | -1.08 | -1.43 | 1.17 | 1 | -1.59 | -1.93 | -3.27 | 1 | -1.54 | -1.75 | -3.12 | 1.39 | NM\_009689.1 |
| RIKEN cDNA 1600012H06 gene | 1600012H06Rik | 1 | -1.40 | -1.23 | -1.04 | 1 | -1.41 | -1.18 | -1.22 | 1 | -3.27 | -1.49 | 1.18 | 1 | -2.65 | -1.25 | 1.07 | 1.10 | NM\_026451.1 |
| transmembrane 4 superfamily member 7 | Tm4sf7 | 1 | 1.04 | -1.14 | -1.11 | 1 | 1.08 | -1.19 | 1.05 | 1 | -1.11 | -3.09 | -1.75 | 1 | -1.17 | -3.24 | -1.46 | 1.14 | NM\_053082.1 |
| arrestin domain containing 3 | Arrdc3 | 1 | -1.18 | 1.13 | 1.06 | 1 | 1.05 | -1.19 | -1.09 | 1 | -2.32 | -3.24 | -1.43 | 1 | -1.89 | -2.07 | 1.25 | 1.06 | NM\_178917.2 |
| DNA segment, Chr 17, human D6S56E 5 | D17H6S56E-5 | 1 | -1.01 | -1.76 | -1.61 | 1 | -1.27 | -1.55 | 1.36 | 1 | -3.23 | -2.09 | -2.32 | 1 | -1.89 | -1.75 | -2.56 | 1.21 | NM\_033075.2 |
| nuclear factor I/X | Nfix | 1 | 1.14 | -1.03 | -1.18 | 1 | 1.15 | 1.05 | 1.38 | 1 | -1.65 | -3.23 | -2.40 | 1 | -1.53 | -3.00 | -1.50 | -1.42 | NM\_010906.1 |
| RIKEN cDNA 1110012D08 gene | 1110012D08Rik | 1 | -1.01 | -1.58 | 1.60 | 1 | -1.45 | 1.44 | 1.51 | 1 | -3.22 | -1.84 | -1.04 | 1 | -2.65 | -1.67 | 1.02 | -1.15 | NM\_178066.2 |
| caveolin 2 | Cav2 | 1 | 1.01 | -1.08 | 1.15 | 1 | 1.32 | 1.36 | 1.41 | 1 | -2.15 | -3.22 | -1.83 | 1 | -1.74 | -2.36 | -1.17 | 1.41 | NM\_016900.2 |
| carnitine palmitoyltransferase 2 | Cpt2 | 1 | -1.08 | -1.06 | -1.83 | 1 | -1.25 | 1.07 | -1.01 | 1 | -3.21 | 1.17 | 1.39 | 1 | -2.03 | 1.19 | 1.47 | 1.08 | NM\_009949 |
| homeo box A7 | Hoxa7 | 1 | -1.14 | -1.13 | 1.02 | 1 | 1.20 | 1.36 | 1.34 | 1 | -3.21 | -1.75 | 1.06 | 1 | -2.62 | -2.05 | 1.05 | -1.66 | NM\_010455.1 |
| homeo box B7 | Hoxb7 | 1 | -1.06 | -1.36 | 1.14 | 1 | -1.49 | 1.18 | -1.06 | 1 | -3.20 | -1.69 | -1.24 | 1 | -2.42 | -1.60 | -1.08 | 1.68 | NM\_010460.2 |
| cell division cycle 7 (S. cerevisiae) | Cdc7 | 1 | -1.03 | -2.22 | 1.04 | 1 | -2.04 | -1.02 | -1.03 | 1 | -3.16 | -1.70 | -2.16 | 1 | -1.92 | -1.71 | -1.78 | -1.16 | NM\_009863.1 |
| cyclin E2 | Ccne2 | 1 | -1.27 | 1.60 | 1.03 | 1 | 1.10 | 1.16 | -1.32 | 1 | -1.98 | -2.33 | -3.16 | 1 | -1.14 | -2.04 | -1.97 | -1.15 | NM\_009830.1 |
| homeo box A5 | Hoxa5 | 1 | -1.10 | -1.09 | 1.45 | 1 | -1.15 | 1.15 | 1.40 | 1 | -3.15 | -2.59 | -1.31 | 1 | -1.99 | -1.78 | 1.09 | -1.28 | NM\_010453.2 |

## Slide 7
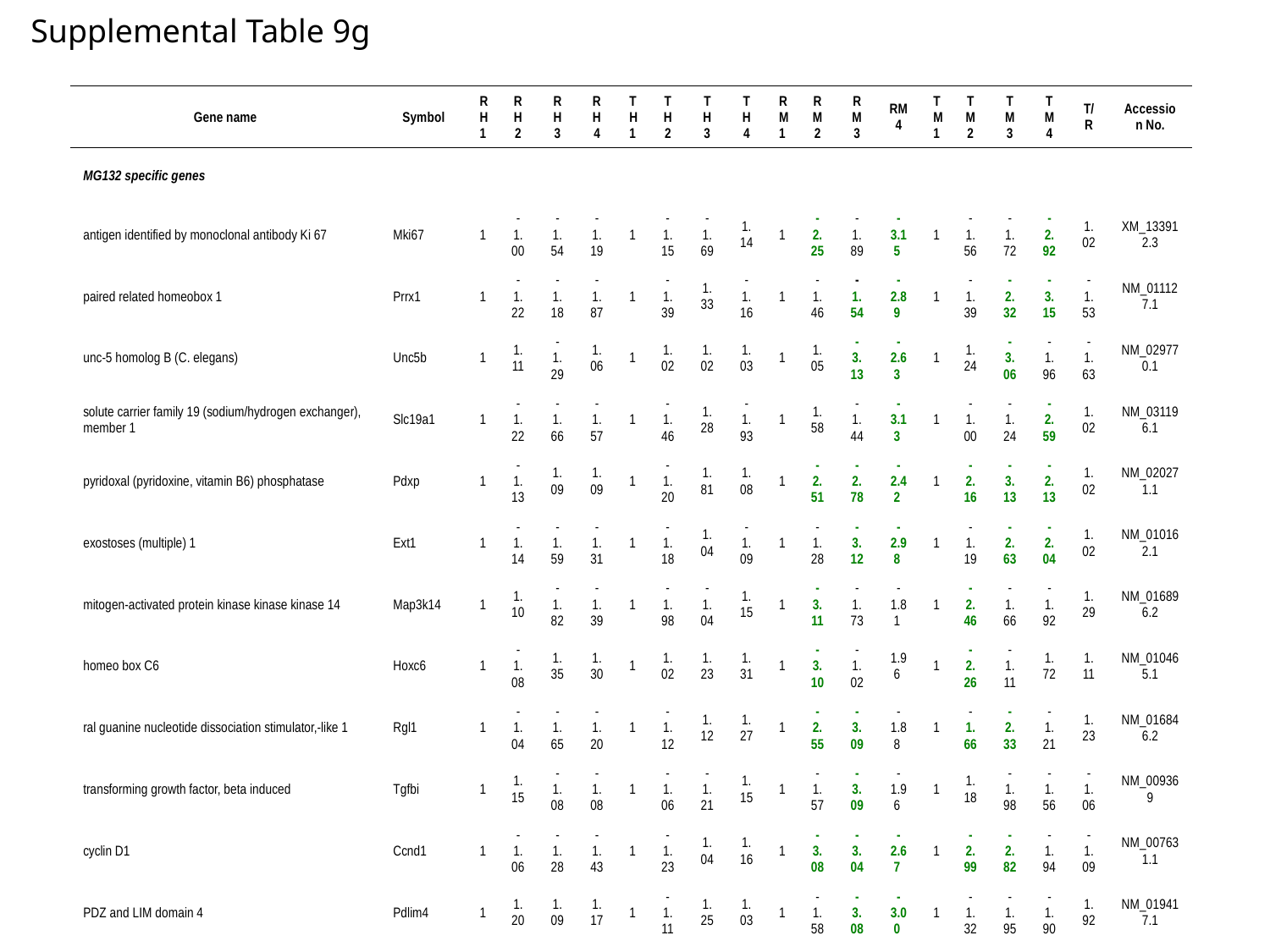

Supplemental Table 9g
| Gene name | Symbol | RH1 | RH2 | RH3 | RH4 | TH1 | TH2 | TH3 | TH4 | RM1 | RM2 | RM3 | RM4 | TM1 | TM2 | TM3 | TM4 | T/R | Accession No. |
| --- | --- | --- | --- | --- | --- | --- | --- | --- | --- | --- | --- | --- | --- | --- | --- | --- | --- | --- | --- |
| MG132 specific genes | | | | | | | | | | | | | | | | | | | |
| antigen identified by monoclonal antibody Ki 67 | Mki67 | 1 | -1.00 | -1.54 | -1.19 | 1 | -1.15 | -1.69 | 1.14 | 1 | -2.25 | -1.89 | -3.15 | 1 | -1.56 | -1.72 | -2.92 | 1.02 | XM\_133912.3 |
| paired related homeobox 1 | Prrx1 | 1 | -1.22 | -1.18 | -1.87 | 1 | -1.39 | 1.33 | -1.16 | 1 | -1.46 | -1.54 | -2.89 | 1 | -1.39 | -2.32 | -3.15 | -1.53 | NM\_011127.1 |
| unc-5 homolog B (C. elegans) | Unc5b | 1 | 1.11 | -1.29 | 1.06 | 1 | 1.02 | 1.02 | 1.03 | 1 | 1.05 | -3.13 | -2.63 | 1 | 1.24 | -3.06 | -1.96 | -1.63 | NM\_029770.1 |
| solute carrier family 19 (sodium/hydrogen exchanger), member 1 | Slc19a1 | 1 | -1.22 | -1.66 | -1.57 | 1 | -1.46 | 1.28 | -1.93 | 1 | 1.58 | -1.44 | -3.13 | 1 | -1.00 | -1.24 | -2.59 | 1.02 | NM\_031196.1 |
| pyridoxal (pyridoxine, vitamin B6) phosphatase | Pdxp | 1 | -1.13 | 1.09 | 1.09 | 1 | -1.20 | 1.81 | 1.08 | 1 | -2.51 | -2.78 | -2.42 | 1 | -2.16 | -3.13 | -2.13 | 1.02 | NM\_020271.1 |
| exostoses (multiple) 1 | Ext1 | 1 | -1.14 | -1.59 | -1.31 | 1 | -1.18 | 1.04 | -1.09 | 1 | -1.28 | -3.12 | -2.98 | 1 | -1.19 | -2.63 | -2.04 | 1.02 | NM\_010162.1 |
| mitogen-activated protein kinase kinase kinase 14 | Map3k14 | 1 | 1.10 | -1.82 | -1.39 | 1 | -1.98 | -1.04 | 1.15 | 1 | -3.11 | -1.73 | -1.81 | 1 | -2.46 | -1.66 | -1.92 | 1.29 | NM\_016896.2 |
| homeo box C6 | Hoxc6 | 1 | -1.08 | 1.35 | 1.30 | 1 | 1.02 | 1.23 | 1.31 | 1 | -3.10 | -1.02 | 1.96 | 1 | -2.26 | -1.11 | 1.72 | 1.11 | NM\_010465.1 |
| ral guanine nucleotide dissociation stimulator,-like 1 | Rgl1 | 1 | -1.04 | -1.65 | -1.20 | 1 | -1.12 | 1.12 | 1.27 | 1 | -2.55 | -3.09 | -1.88 | 1 | -1.66 | -2.33 | -1.21 | 1.23 | NM\_016846.2 |
| transforming growth factor, beta induced | Tgfbi | 1 | 1.15 | -1.08 | -1.08 | 1 | -1.06 | -1.21 | 1.15 | 1 | -1.57 | -3.09 | -1.96 | 1 | 1.18 | -1.98 | -1.56 | -1.06 | NM\_009369 |
| cyclin D1 | Ccnd1 | 1 | -1.06 | -1.28 | -1.43 | 1 | -1.23 | 1.04 | 1.16 | 1 | -3.08 | -3.04 | -2.67 | 1 | -2.99 | -2.82 | -1.94 | -1.09 | NM\_007631.1 |
| PDZ and LIM domain 4 | Pdlim4 | 1 | 1.20 | 1.09 | 1.17 | 1 | -1.11 | 1.25 | 1.03 | 1 | -1.58 | -3.08 | -3.00 | 1 | -1.32 | -1.95 | -1.90 | 1.92 | NM\_019417.1 |
| MAD homolog 3 (Drosophila) | Smad3 | 1 | 1.69 | 1.20 | 1.62 | 1 | 1.06 | 1.45 | 1.71 | 1 | -3.08 | -2.41 | -1.53 | 1 | -1.81 | -2.44 | -1.16 | 1.02 | NM\_016769 |
| a disintegrin-like and metalloprotease (reprolysin type) with thrombospondin type 1 motif, 7 | Adamts7 | 1 | -1.14 | -1.57 | -1.02 | 1 | -1.11 | 1.44 | 1.71 | 1 | -2.30 | -3.07 | -2.35 | 1 | -1.48 | -1.99 | -1.56 | -2.33 | XM\_135041.5 |
| replication protein A2 | Rpa2 | 1 | 1.09 | -1.75 | 1.24 | 1 | -1.26 | 1.47 | 1.19 | 1 | -1.31 | -1.78 | -3.07 | 1 | -1.22 | -2.10 | -2.15 | 1.04 | NM\_011284.2 |
| cyclin D1 | Ccnd1 | 1 | 1.01 | -1.25 | -1.69 | 1 | -1.14 | -1.20 | -1.01 | 1 | -3.00 | -2.86 | -2.41 | 1 | -3.07 | -2.97 | -2.06 | 1.05 | NM\_007631.1 |
| aurora kinase B | Aurkb | 1 | 1.02 | -1.62 | -1.41 | 1 | -1.10 | -1.27 | 1.16 | 1 | -2.40 | -1.49 | -3.06 | 1 | -2.19 | -2.01 | -2.95 | -1.04 | XM\_181344.3 |
| DNA segment, Chr 6, Wayne State University 176, expressed | D6Wsu176e | 1 | -1.11 | -1.17 | -2.02 | 1 | -1.20 | -1.50 | -1.43 | 1 | -1.31 | -2.42 | -3.06 | 1 | -1.50 | -2.80 | -2.30 | -1.41 | NM\_138587.3 |
| left-right determination, factor B | Leftb | 1 | -1.13 | -1.49 | -1.10 | 1 | -1.59 | 1.39 | 1.13 | 1 | -3.05 | -1.94 | -1.63 | 1 | -2.47 | -1.53 | -1.35 | 1.18 | NM\_010094.2 |
| tribbles homolog 2 (Drosophila) | Trib2 | 1 | -1.08 | -1.54 | -1.10 | 1 | -1.44 | 1.09 | 1.05 | 1 | -2.13 | -3.05 | -1.93 | 1 | -2.02 | -2.25 | -1.34 | 1.04 | NM\_144551.3 |
| RIKEN cDNA 4732435N03 gene | 4732435N03Rik | 1 | 1.11 | -1.55 | -2.03 | 1 | -1.27 | 1.07 | -1.06 | 1 | -1.67 | -3.05 | -2.70 | 1 | -1.78 | -2.74 | -2.19 | -1.74 | NM\_172753.2 |
| nudix (nucleoside diphosphate linked moiety X)-type motif 16 | Nudt16 | 1 | 1.04 | -1.15 | 2.21 | 1 | -1.27 | 1.66 | 1.97 | 1 | -3.04 | -1.45 | 1.53 | 1 | -2.38 | 1.08 | 2.44 | 1.02 | NM\_029385.1 |
| RIKEN cDNA A030007L17 gene | A030007L17Rik | 1 | -1.54 | -1.46 | 1.01 | 1 | -1.10 | 1.41 | -1.29 | 1 | -1.10 | -2.21 | -3.03 | 1 | 1.05 | -1.14 | -1.40 | -2.48 | NM\_026637.2 |
| procollagen, type XVI, alpha 1 | Col16a1 | 1 | 1.03 | -1.17 | -1.56 | 1 | -1.03 | -1.28 | 1.04 | 1 | -1.28 | -2.53 | -3.03 | 1 | -1.17 | -1.75 | -1.91 | -1.14 | NM\_028266.3 |
| RIKEN cDNA 2310003H01 gene | 2310003H01Rik | 1 | -1.32 | -1.08 | -1.13 | 1 | -1.29 | 1.20 | -1.26 | 1 | -1.24 | -2.76 | -3.03 | 1 | -1.61 | -2.39 | -2.34 | 1.15 | XM\_126658.3 |
| cDNA sequence BC025076 | BC025076 | 1 | 1.04 | -1.44 | 1.11 | 1 | -1.16 | 1.25 | 1.07 | 1 | -3.02 | -2.29 | -1.48 | 1 | -2.50 | -2.05 | -1.22 | -1.21 | NM\_175002.1 |
| ubiquitin-like, containing PHD and RING finger domains, 1 | Uhrf1 | 1 | -1.02 | -1.47 | -1.19 | 1 | -1.21 | -1.07 | -1.06 | 1 | -1.70 | -1.97 | -3.02 | 1 | -1.48 | -2.02 | -2.22 | -1.02 | NM\_010931.2 |
| RIKEN cDNA 6720460F02 gene | 6720460F02Rik | 1 | -1.06 | -1.83 | -1.32 | 1 | -1.95 | -1.56 | -1.14 | 1 | -2.29 | -1.39 | -2.37 | 1 | -2.28 | -1.70 | -3.02 | 1.17 | NM\_144526.2 |
| RIKEN cDNA 1190002H23 gene | 1190002H23Rik | 1 | 1.12 | 14.49 | 4.55 | 1 | 2.95 | 1.37 | -1.10 | 1 | -1.17 | -3.01 | -1.93 | 1 | 1.07 | -1.91 | -1.41 | -1.39 | NM\_025427.1 |
| O-acyltransferase (membrane bound) domain containing 1 | Oact1 | 1 | 1.03 | -1.16 | -1.39 | 1 | 1.01 | -1.37 | -1.08 | 1 | -1.37 | -1.86 | -3.01 | 1 | -1.06 | -1.95 | -1.81 | -1.02 | NM\_153546.1 |
| SERTA domain containing 4 | Sertad4 | 1 | -1.17 | -1.49 | -1.79 | 1 | -1.65 | -1.79 | -1.36 | 1 | -2.48 | -1.35 | -1.72 | 1 | -3.01 | -1.73 | -1.64 | 1.46 | NM\_198247.1 |
| ELK3, member of ETS oncogene family (Elk3), transcript variant 2 | Elk3 | 1 | 1.02 | -1.43 | -1.29 | 1 | -1.35 | -1.35 | -1.16 | 1 | -2.36 | -3.01 | -2.42 | 1 | -1.71 | -2.33 | -1.65 | -1.10 | NM\_205536.1 |
| pyruvate dehydrogenase kinase, isoenzyme 1 | Pdk1 | 1 | 1.35 | 1.19 | -1.15 | 1 | 1.47 | -1.00 | 1.54 | 1 | -1.27 | -2.17 | -1.21 | 1 | -1.20 | -3.01 | -1.21 | -1.10 | NM\_172665.1 |
| RIKEN cDNA 9030425E11 gene | 9030425E11Rik | 1 | 1.04 | -1.18 | -1.18 | 1 | -1.04 | -1.07 | 1.02 | 1 | -1.43 | -2.89 | -3.00 | 1 | -1.21 | -2.17 | -1.96 | -1.43 | NM\_133733.2 |
