# Supplementary material for: Systemic Analysis of Heat Shock Response Induced by Heat Shock and a Proteasome Inhibitor MG132
Source: PLoS One. 2011 Jun 30;6(6):e20252. doi: 10.1371/journal.pone.0020252 (PMC3127947; doi:10.1371/journal.pone.0020252)
Supplement: Table S10 — Innate immunity signaling genes are listed with their mRNA levels in response to heat shock and MG132 treatment. Fold changes more than 2 are colored in red and less than -2 are colored in green. (PPT) [file pone.0020252.s017.ppt]

## Slide 1
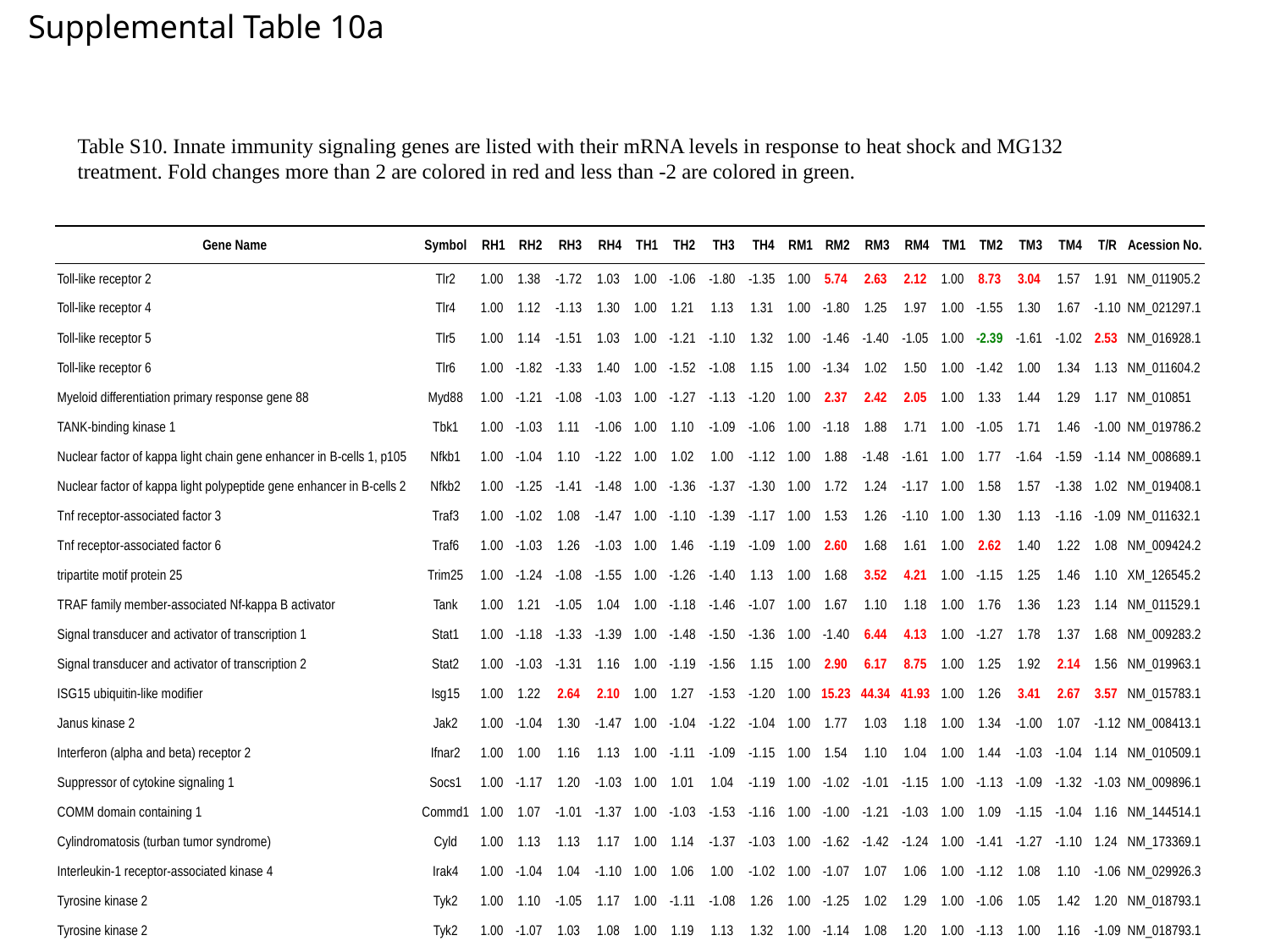

Supplemental Table 10a
Table S10. Innate immunity signaling genes are listed with their mRNA levels in response to heat shock and MG132 treatment. Fold changes more than 2 are colored in red and less than -2 are colored in green.
| Gene Name | Symbol | RH1 | RH2 | RH3 | RH4 | TH1 | TH2 | TH3 | TH4 | RM1 | RM2 | RM3 | RM4 | TM1 | TM2 | TM3 | TM4 | T/R | Acession No. |
| --- | --- | --- | --- | --- | --- | --- | --- | --- | --- | --- | --- | --- | --- | --- | --- | --- | --- | --- | --- |
| Toll-like receptor 2 | Tlr2 | 1.00 | 1.38 | -1.72 | 1.03 | 1.00 | -1.06 | -1.80 | -1.35 | 1.00 | 5.74 | 2.63 | 2.12 | 1.00 | 8.73 | 3.04 | 1.57 | 1.91 | NM\_011905.2 |
| Toll-like receptor 4 | Tlr4 | 1.00 | 1.12 | -1.13 | 1.30 | 1.00 | 1.21 | 1.13 | 1.31 | 1.00 | -1.80 | 1.25 | 1.97 | 1.00 | -1.55 | 1.30 | 1.67 | -1.10 | NM\_021297.1 |
| Toll-like receptor 5 | Tlr5 | 1.00 | 1.14 | -1.51 | 1.03 | 1.00 | -1.21 | -1.10 | 1.32 | 1.00 | -1.46 | -1.40 | -1.05 | 1.00 | -2.39 | -1.61 | -1.02 | 2.53 | NM\_016928.1 |
| Toll-like receptor 6 | Tlr6 | 1.00 | -1.82 | -1.33 | 1.40 | 1.00 | -1.52 | -1.08 | 1.15 | 1.00 | -1.34 | 1.02 | 1.50 | 1.00 | -1.42 | 1.00 | 1.34 | 1.13 | NM\_011604.2 |
| Myeloid differentiation primary response gene 88 | Myd88 | 1.00 | -1.21 | -1.08 | -1.03 | 1.00 | -1.27 | -1.13 | -1.20 | 1.00 | 2.37 | 2.42 | 2.05 | 1.00 | 1.33 | 1.44 | 1.29 | 1.17 | NM\_010851 |
| TANK-binding kinase 1 | Tbk1 | 1.00 | -1.03 | 1.11 | -1.06 | 1.00 | 1.10 | -1.09 | -1.06 | 1.00 | -1.18 | 1.88 | 1.71 | 1.00 | -1.05 | 1.71 | 1.46 | -1.00 | NM\_019786.2 |
| Nuclear factor of kappa light chain gene enhancer in B-cells 1, p105 | Nfkb1 | 1.00 | -1.04 | 1.10 | -1.22 | 1.00 | 1.02 | 1.00 | -1.12 | 1.00 | 1.88 | -1.48 | -1.61 | 1.00 | 1.77 | -1.64 | -1.59 | -1.14 | NM\_008689.1 |
| Nuclear factor of kappa light polypeptide gene enhancer in B-cells 2 | Nfkb2 | 1.00 | -1.25 | -1.41 | -1.48 | 1.00 | -1.36 | -1.37 | -1.30 | 1.00 | 1.72 | 1.24 | -1.17 | 1.00 | 1.58 | 1.57 | -1.38 | 1.02 | NM\_019408.1 |
| Tnf receptor-associated factor 3 | Traf3 | 1.00 | -1.02 | 1.08 | -1.47 | 1.00 | -1.10 | -1.39 | -1.17 | 1.00 | 1.53 | 1.26 | -1.10 | 1.00 | 1.30 | 1.13 | -1.16 | -1.09 | NM\_011632.1 |
| Tnf receptor-associated factor 6 | Traf6 | 1.00 | -1.03 | 1.26 | -1.03 | 1.00 | 1.46 | -1.19 | -1.09 | 1.00 | 2.60 | 1.68 | 1.61 | 1.00 | 2.62 | 1.40 | 1.22 | 1.08 | NM\_009424.2 |
| tripartite motif protein 25 | Trim25 | 1.00 | -1.24 | -1.08 | -1.55 | 1.00 | -1.26 | -1.40 | 1.13 | 1.00 | 1.68 | 3.52 | 4.21 | 1.00 | -1.15 | 1.25 | 1.46 | 1.10 | XM\_126545.2 |
| TRAF family member-associated Nf-kappa B activator | Tank | 1.00 | 1.21 | -1.05 | 1.04 | 1.00 | -1.18 | -1.46 | -1.07 | 1.00 | 1.67 | 1.10 | 1.18 | 1.00 | 1.76 | 1.36 | 1.23 | 1.14 | NM\_011529.1 |
| Signal transducer and activator of transcription 1 | Stat1 | 1.00 | -1.18 | -1.33 | -1.39 | 1.00 | -1.48 | -1.50 | -1.36 | 1.00 | -1.40 | 6.44 | 4.13 | 1.00 | -1.27 | 1.78 | 1.37 | 1.68 | NM\_009283.2 |
| Signal transducer and activator of transcription 2 | Stat2 | 1.00 | -1.03 | -1.31 | 1.16 | 1.00 | -1.19 | -1.56 | 1.15 | 1.00 | 2.90 | 6.17 | 8.75 | 1.00 | 1.25 | 1.92 | 2.14 | 1.56 | NM\_019963.1 |
| ISG15 ubiquitin-like modifier | Isg15 | 1.00 | 1.22 | 2.64 | 2.10 | 1.00 | 1.27 | -1.53 | -1.20 | 1.00 | 15.23 | 44.34 | 41.93 | 1.00 | 1.26 | 3.41 | 2.67 | 3.57 | NM\_015783.1 |
| Janus kinase 2 | Jak2 | 1.00 | -1.04 | 1.30 | -1.47 | 1.00 | -1.04 | -1.22 | -1.04 | 1.00 | 1.77 | 1.03 | 1.18 | 1.00 | 1.34 | -1.00 | 1.07 | -1.12 | NM\_008413.1 |
| Interferon (alpha and beta) receptor 2 | Ifnar2 | 1.00 | 1.00 | 1.16 | 1.13 | 1.00 | -1.11 | -1.09 | -1.15 | 1.00 | 1.54 | 1.10 | 1.04 | 1.00 | 1.44 | -1.03 | -1.04 | 1.14 | NM\_010509.1 |
| Suppressor of cytokine signaling 1 | Socs1 | 1.00 | -1.17 | 1.20 | -1.03 | 1.00 | 1.01 | 1.04 | -1.19 | 1.00 | -1.02 | -1.01 | -1.15 | 1.00 | -1.13 | -1.09 | -1.32 | -1.03 | NM\_009896.1 |
| COMM domain containing 1 | Commd1 | 1.00 | 1.07 | -1.01 | -1.37 | 1.00 | -1.03 | -1.53 | -1.16 | 1.00 | -1.00 | -1.21 | -1.03 | 1.00 | 1.09 | -1.15 | -1.04 | 1.16 | NM\_144514.1 |
| Cylindromatosis (turban tumor syndrome) | Cyld | 1.00 | 1.13 | 1.13 | 1.17 | 1.00 | 1.14 | -1.37 | -1.03 | 1.00 | -1.62 | -1.42 | -1.24 | 1.00 | -1.41 | -1.27 | -1.10 | 1.24 | NM\_173369.1 |
| Interleukin-1 receptor-associated kinase 4 | Irak4 | 1.00 | -1.04 | 1.04 | -1.10 | 1.00 | 1.06 | 1.00 | -1.02 | 1.00 | -1.07 | 1.07 | 1.06 | 1.00 | -1.12 | 1.08 | 1.10 | -1.06 | NM\_029926.3 |
| Tyrosine kinase 2 | Tyk2 | 1.00 | 1.10 | -1.05 | 1.17 | 1.00 | -1.11 | -1.08 | 1.26 | 1.00 | -1.25 | 1.02 | 1.29 | 1.00 | -1.06 | 1.05 | 1.42 | 1.20 | NM\_018793.1 |
| Tyrosine kinase 2 | Tyk2 | 1.00 | -1.07 | 1.03 | 1.08 | 1.00 | 1.19 | 1.13 | 1.32 | 1.00 | -1.14 | 1.08 | 1.20 | 1.00 | -1.13 | 1.00 | 1.16 | -1.09 | NM\_018793.1 |
| PDZ and LIM domain 2 | Pdlim2 | 1.00 | 1.56 | -1.05 | 1.89 | 1.00 | 1.62 | 1.74 | 1.76 | 1.00 | -1.89 | -2.23 | -1.24 | 1.00 | -1.54 | -1.79 | -1.12 | -1.47 | NM\_145978.1 |
| Activating transcription factor 2 | Atf2 | 1.00 | 1.22 | 1.41 | 1.76 | 1.00 | 1.11 | 1.32 | 1.19 | 1.00 | 1.09 | 1.24 | 1.36 | 1.00 | 1.02 | 1.14 | 1.09 | -1.04 | NM\_009715.1 |

## Slide 2
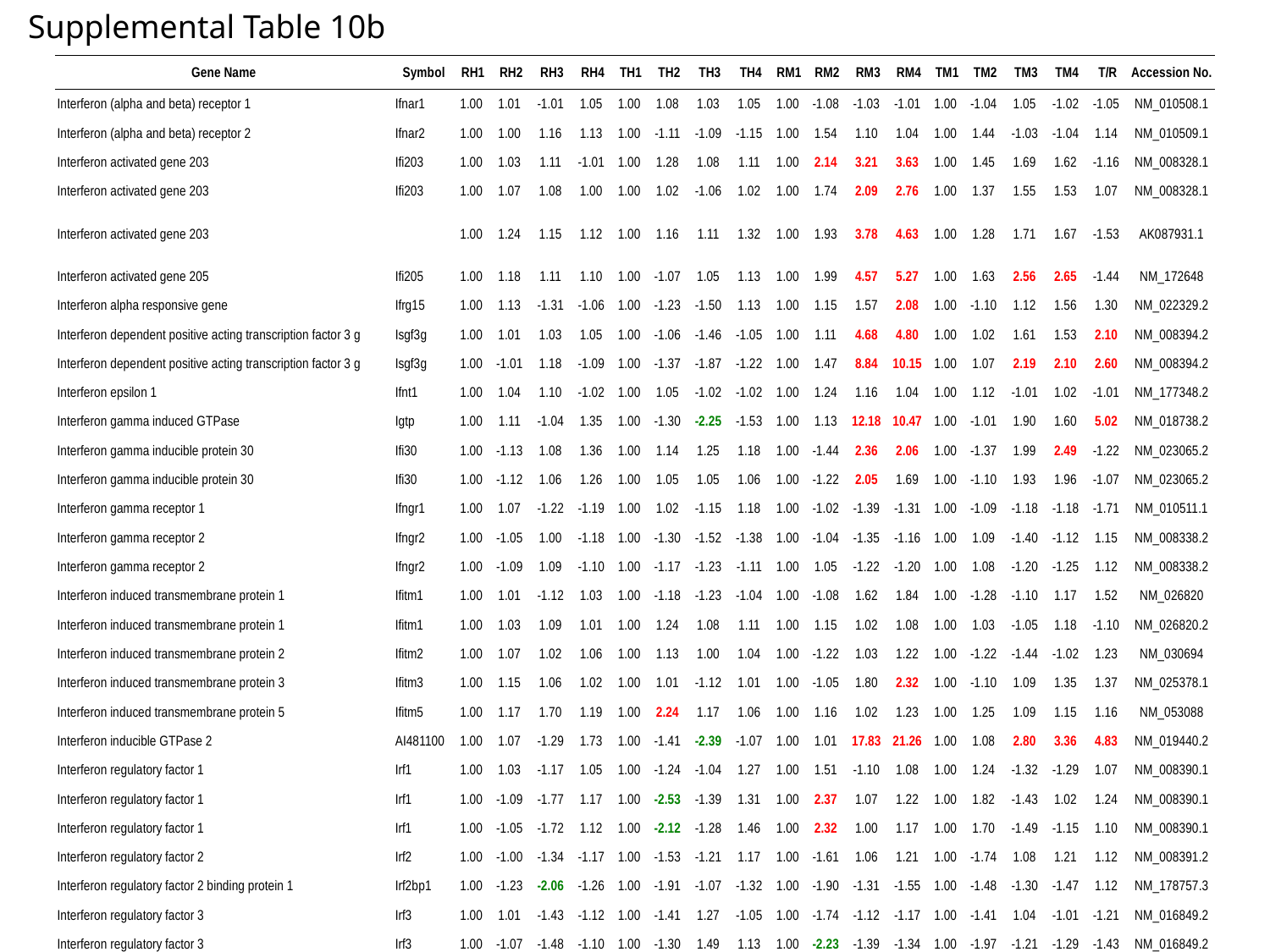

Supplemental Table 10b
| Gene Name | Symbol | RH1 | RH2 | RH3 | RH4 | TH1 | TH2 | TH3 | TH4 | RM1 | RM2 | RM3 | RM4 | TM1 | TM2 | TM3 | TM4 | T/R | Accession No. |
| --- | --- | --- | --- | --- | --- | --- | --- | --- | --- | --- | --- | --- | --- | --- | --- | --- | --- | --- | --- |
| Interferon (alpha and beta) receptor 1 | Ifnar1 | 1.00 | 1.01 | -1.01 | 1.05 | 1.00 | 1.08 | 1.03 | 1.05 | 1.00 | -1.08 | -1.03 | -1.01 | 1.00 | -1.04 | 1.05 | -1.02 | -1.05 | NM\_010508.1 |
| Interferon (alpha and beta) receptor 2 | Ifnar2 | 1.00 | 1.00 | 1.16 | 1.13 | 1.00 | -1.11 | -1.09 | -1.15 | 1.00 | 1.54 | 1.10 | 1.04 | 1.00 | 1.44 | -1.03 | -1.04 | 1.14 | NM\_010509.1 |
| Interferon activated gene 203 | Ifi203 | 1.00 | 1.03 | 1.11 | -1.01 | 1.00 | 1.28 | 1.08 | 1.11 | 1.00 | 2.14 | 3.21 | 3.63 | 1.00 | 1.45 | 1.69 | 1.62 | -1.16 | NM\_008328.1 |
| Interferon activated gene 203 | Ifi203 | 1.00 | 1.07 | 1.08 | 1.00 | 1.00 | 1.02 | -1.06 | 1.02 | 1.00 | 1.74 | 2.09 | 2.76 | 1.00 | 1.37 | 1.55 | 1.53 | 1.07 | NM\_008328.1 |
| Interferon activated gene 203 | | 1.00 | 1.24 | 1.15 | 1.12 | 1.00 | 1.16 | 1.11 | 1.32 | 1.00 | 1.93 | 3.78 | 4.63 | 1.00 | 1.28 | 1.71 | 1.67 | -1.53 | AK087931.1 |
| Interferon activated gene 205 | Ifi205 | 1.00 | 1.18 | 1.11 | 1.10 | 1.00 | -1.07 | 1.05 | 1.13 | 1.00 | 1.99 | 4.57 | 5.27 | 1.00 | 1.63 | 2.56 | 2.65 | -1.44 | NM\_172648 |
| Interferon alpha responsive gene | Ifrg15 | 1.00 | 1.13 | -1.31 | -1.06 | 1.00 | -1.23 | -1.50 | 1.13 | 1.00 | 1.15 | 1.57 | 2.08 | 1.00 | -1.10 | 1.12 | 1.56 | 1.30 | NM\_022329.2 |
| Interferon dependent positive acting transcription factor 3 g | Isgf3g | 1.00 | 1.01 | 1.03 | 1.05 | 1.00 | -1.06 | -1.46 | -1.05 | 1.00 | 1.11 | 4.68 | 4.80 | 1.00 | 1.02 | 1.61 | 1.53 | 2.10 | NM\_008394.2 |
| Interferon dependent positive acting transcription factor 3 g | Isgf3g | 1.00 | -1.01 | 1.18 | -1.09 | 1.00 | -1.37 | -1.87 | -1.22 | 1.00 | 1.47 | 8.84 | 10.15 | 1.00 | 1.07 | 2.19 | 2.10 | 2.60 | NM\_008394.2 |
| Interferon epsilon 1 | Ifnt1 | 1.00 | 1.04 | 1.10 | -1.02 | 1.00 | 1.05 | -1.02 | -1.02 | 1.00 | 1.24 | 1.16 | 1.04 | 1.00 | 1.12 | -1.01 | 1.02 | -1.01 | NM\_177348.2 |
| Interferon gamma induced GTPase | Igtp | 1.00 | 1.11 | -1.04 | 1.35 | 1.00 | -1.30 | -2.25 | -1.53 | 1.00 | 1.13 | 12.18 | 10.47 | 1.00 | -1.01 | 1.90 | 1.60 | 5.02 | NM\_018738.2 |
| Interferon gamma inducible protein 30 | Ifi30 | 1.00 | -1.13 | 1.08 | 1.36 | 1.00 | 1.14 | 1.25 | 1.18 | 1.00 | -1.44 | 2.36 | 2.06 | 1.00 | -1.37 | 1.99 | 2.49 | -1.22 | NM\_023065.2 |
| Interferon gamma inducible protein 30 | Ifi30 | 1.00 | -1.12 | 1.06 | 1.26 | 1.00 | 1.05 | 1.05 | 1.06 | 1.00 | -1.22 | 2.05 | 1.69 | 1.00 | -1.10 | 1.93 | 1.96 | -1.07 | NM\_023065.2 |
| Interferon gamma receptor 1 | Ifngr1 | 1.00 | 1.07 | -1.22 | -1.19 | 1.00 | 1.02 | -1.15 | 1.18 | 1.00 | -1.02 | -1.39 | -1.31 | 1.00 | -1.09 | -1.18 | -1.18 | -1.71 | NM\_010511.1 |
| Interferon gamma receptor 2 | Ifngr2 | 1.00 | -1.05 | 1.00 | -1.18 | 1.00 | -1.30 | -1.52 | -1.38 | 1.00 | -1.04 | -1.35 | -1.16 | 1.00 | 1.09 | -1.40 | -1.12 | 1.15 | NM\_008338.2 |
| Interferon gamma receptor 2 | Ifngr2 | 1.00 | -1.09 | 1.09 | -1.10 | 1.00 | -1.17 | -1.23 | -1.11 | 1.00 | 1.05 | -1.22 | -1.20 | 1.00 | 1.08 | -1.20 | -1.25 | 1.12 | NM\_008338.2 |
| Interferon induced transmembrane protein 1 | Ifitm1 | 1.00 | 1.01 | -1.12 | 1.03 | 1.00 | -1.18 | -1.23 | -1.04 | 1.00 | -1.08 | 1.62 | 1.84 | 1.00 | -1.28 | -1.10 | 1.17 | 1.52 | NM\_026820 |
| Interferon induced transmembrane protein 1 | Ifitm1 | 1.00 | 1.03 | 1.09 | 1.01 | 1.00 | 1.24 | 1.08 | 1.11 | 1.00 | 1.15 | 1.02 | 1.08 | 1.00 | 1.03 | -1.05 | 1.18 | -1.10 | NM\_026820.2 |
| Interferon induced transmembrane protein 2 | Ifitm2 | 1.00 | 1.07 | 1.02 | 1.06 | 1.00 | 1.13 | 1.00 | 1.04 | 1.00 | -1.22 | 1.03 | 1.22 | 1.00 | -1.22 | -1.44 | -1.02 | 1.23 | NM\_030694 |
| Interferon induced transmembrane protein 3 | Ifitm3 | 1.00 | 1.15 | 1.06 | 1.02 | 1.00 | 1.01 | -1.12 | 1.01 | 1.00 | -1.05 | 1.80 | 2.32 | 1.00 | -1.10 | 1.09 | 1.35 | 1.37 | NM\_025378.1 |
| Interferon induced transmembrane protein 5 | Ifitm5 | 1.00 | 1.17 | 1.70 | 1.19 | 1.00 | 2.24 | 1.17 | 1.06 | 1.00 | 1.16 | 1.02 | 1.23 | 1.00 | 1.25 | 1.09 | 1.15 | 1.16 | NM\_053088 |
| Interferon inducible GTPase 2 | AI481100 | 1.00 | 1.07 | -1.29 | 1.73 | 1.00 | -1.41 | -2.39 | -1.07 | 1.00 | 1.01 | 17.83 | 21.26 | 1.00 | 1.08 | 2.80 | 3.36 | 4.83 | NM\_019440.2 |
| Interferon regulatory factor 1 | Irf1 | 1.00 | 1.03 | -1.17 | 1.05 | 1.00 | -1.24 | -1.04 | 1.27 | 1.00 | 1.51 | -1.10 | 1.08 | 1.00 | 1.24 | -1.32 | -1.29 | 1.07 | NM\_008390.1 |
| Interferon regulatory factor 1 | Irf1 | 1.00 | -1.09 | -1.77 | 1.17 | 1.00 | -2.53 | -1.39 | 1.31 | 1.00 | 2.37 | 1.07 | 1.22 | 1.00 | 1.82 | -1.43 | 1.02 | 1.24 | NM\_008390.1 |
| Interferon regulatory factor 1 | Irf1 | 1.00 | -1.05 | -1.72 | 1.12 | 1.00 | -2.12 | -1.28 | 1.46 | 1.00 | 2.32 | 1.00 | 1.17 | 1.00 | 1.70 | -1.49 | -1.15 | 1.10 | NM\_008390.1 |
| Interferon regulatory factor 2 | Irf2 | 1.00 | -1.00 | -1.34 | -1.17 | 1.00 | -1.53 | -1.21 | 1.17 | 1.00 | -1.61 | 1.06 | 1.21 | 1.00 | -1.74 | 1.08 | 1.21 | 1.12 | NM\_008391.2 |
| Interferon regulatory factor 2 binding protein 1 | Irf2bp1 | 1.00 | -1.23 | -2.06 | -1.26 | 1.00 | -1.91 | -1.07 | -1.32 | 1.00 | -1.90 | -1.31 | -1.55 | 1.00 | -1.48 | -1.30 | -1.47 | 1.12 | NM\_178757.3 |
| Interferon regulatory factor 3 | Irf3 | 1.00 | 1.01 | -1.43 | -1.12 | 1.00 | -1.41 | 1.27 | -1.05 | 1.00 | -1.74 | -1.12 | -1.17 | 1.00 | -1.41 | 1.04 | -1.01 | -1.21 | NM\_016849.2 |
| Interferon regulatory factor 3 | Irf3 | 1.00 | -1.07 | -1.48 | -1.10 | 1.00 | -1.30 | 1.49 | 1.13 | 1.00 | -2.23 | -1.39 | -1.34 | 1.00 | -1.97 | -1.21 | -1.29 | -1.43 | NM\_016849.2 |
| Interferon regulatory factor 3 | Irf3 | 1.00 | -1.12 | -1.81 | -1.26 | 1.00 | -1.78 | 1.18 | -1.03 | 1.00 | -2.78 | -1.19 | -1.33 | 1.00 | -2.64 | -1.28 | -1.23 | -1.21 | NM\_016849.2 |
| Interferon regulatory factor 5 | Irf5 | 1.00 | 1.23 | -1.21 | 1.05 | 1.00 | -1.19 | -1.16 | 1.16 | 1.00 | 1.04 | 1.39 | 1.16 | 1.00 | 1.43 | -1.02 | -1.01 | -1.36 | NM\_012057.1 |
| Interferon regulatory factor 7 | Irf7 | 1.00 | 1.02 | -1.05 | 1.02 | 1.00 | -1.10 | -1.40 | 1.00 | 1.00 | -1.08 | 5.59 | 9.61 | 1.00 | -1.06 | 2.17 | 2.44 | 1.57 | NM\_016850.1 |
| Interferon stimulated exonuclease gene 20-like 1 | Isg20l1 | 1.00 | -1.23 | -1.26 | -1.39 | 1.00 | 1.07 | -1.26 | -1.29 | 1.00 | 1.66 | 1.02 | -1.09 | 1.00 | 1.36 | 1.10 | -1.09 | -1.00 | NM\_026531.2 |
| Interferon stimulated exonuclease gene 20-like 1 | Isg20l1 | 1.00 | -1.09 | 1.04 | -1.21 | 1.00 | 1.06 | 1.08 | -1.22 | 1.00 | 1.90 | 1.29 | -1.14 | 1.00 | 1.35 | 1.24 | -1.21 | -1.06 | NM\_026531.2 |
| Interferon-induced protein 35 | Ifi35 | 1.00 | -1.03 | -1.17 | -1.17 | 1.00 | -1.16 | -1.25 | -1.24 | 1.00 | 1.08 | 3.92 | 3.57 | 1.00 | 1.05 | 2.21 | 1.88 | 1.81 | NM\_027320.1 |
| Interferon-induced protein with tetratricopeptide repeats 2 | Ifit2 | 1.00 | 1.12 | -1.23 | 1.14 | 1.00 | -1.16 | -1.41 | -1.08 | 1.00 | 2.05 | 3.20 | 2.66 | 1.00 | -1.10 | 1.40 | 1.82 | 1.59 | NM\_008332.2 |
| Interferon-induced protein with tetratricopeptide repeats 3 | Ifit3 | 1.00 | -1.04 | 1.07 | 1.25 | 1.00 | -1.23 | -2.01 | -1.10 | 1.00 | 4.62 | 35.88 | 34.95 | 1.00 | 1.03 | 3.53 | 3.32 | 3.46 | NM\_010501.1 |
| Interferon-induced protein with tetratricopeptide repeats 3 | Ifit3 | 1.00 | 1.01 | 1.01 | 1.14 | 1.00 | -1.16 | -2.10 | -1.17 | 1.00 | 4.90 | 40.07 | 34.62 | 1.00 | -1.00 | 3.58 | 3.32 | 3.63 | NM\_010501.1 |
| Interferon-related developmental regulator 2 | Ifrd2 | 1.00 | -1.06 | -1.37 | -1.43 | 1.00 | -1.40 | -1.04 | -1.26 | 1.00 | -1.30 | -1.18 | -2.05 | 1.00 | -1.52 | -1.17 | -1.96 | 1.15 | NM\_025903.1 |
| Interferon-stimulated protein | Isg20 | 1.00 | 1.18 | 2.04 | -1.05 | 1.00 | 1.45 | -1.65 | -1.43 | 1.00 | 1.19 | 2.00 | 2.06 | 1.00 | -1.02 | 1.19 | 1.39 | 1.52 | NM\_020583.4 |
| Protein kinase, interferon inducible double stranded RNA dependent activator | Prkra | 1.00 | 1.14 | -1.08 | -1.05 | 1.00 | 1.04 | 1.26 | 1.06 | 1.00 | -1.48 | -1.07 | 1.16 | 1.00 | -1.39 | -1.23 | 1.12 | 1.11 | NM\_011871.1 |
| Protein kinase, X-linked | Prkx | 1.00 | 1.12 | 1.85 | -1.08 | 1.00 | 1.44 | -1.22 | -1.50 | 1.00 | 2.13 | 1.53 | 1.77 | 1.00 | 1.52 | 1.24 | 1.53 | 1.15 | NM\_016979.1 |
