# Supplementary material for: Systemic Analysis of Heat Shock Response Induced by Heat Shock and a Proteasome Inhibitor MG132
Source: PLoS One. 2011 Jun 30;6(6):e20252. doi: 10.1371/journal.pone.0020252 (PMC3127947; doi:10.1371/journal.pone.0020252)
Supplement: Table S11 — Predicted transcription factors possibly activated in heat shock or MG132 treated RIF-1 and TR cells. (PPT) [file pone.0020252.s018.ppt]

## Slide 1
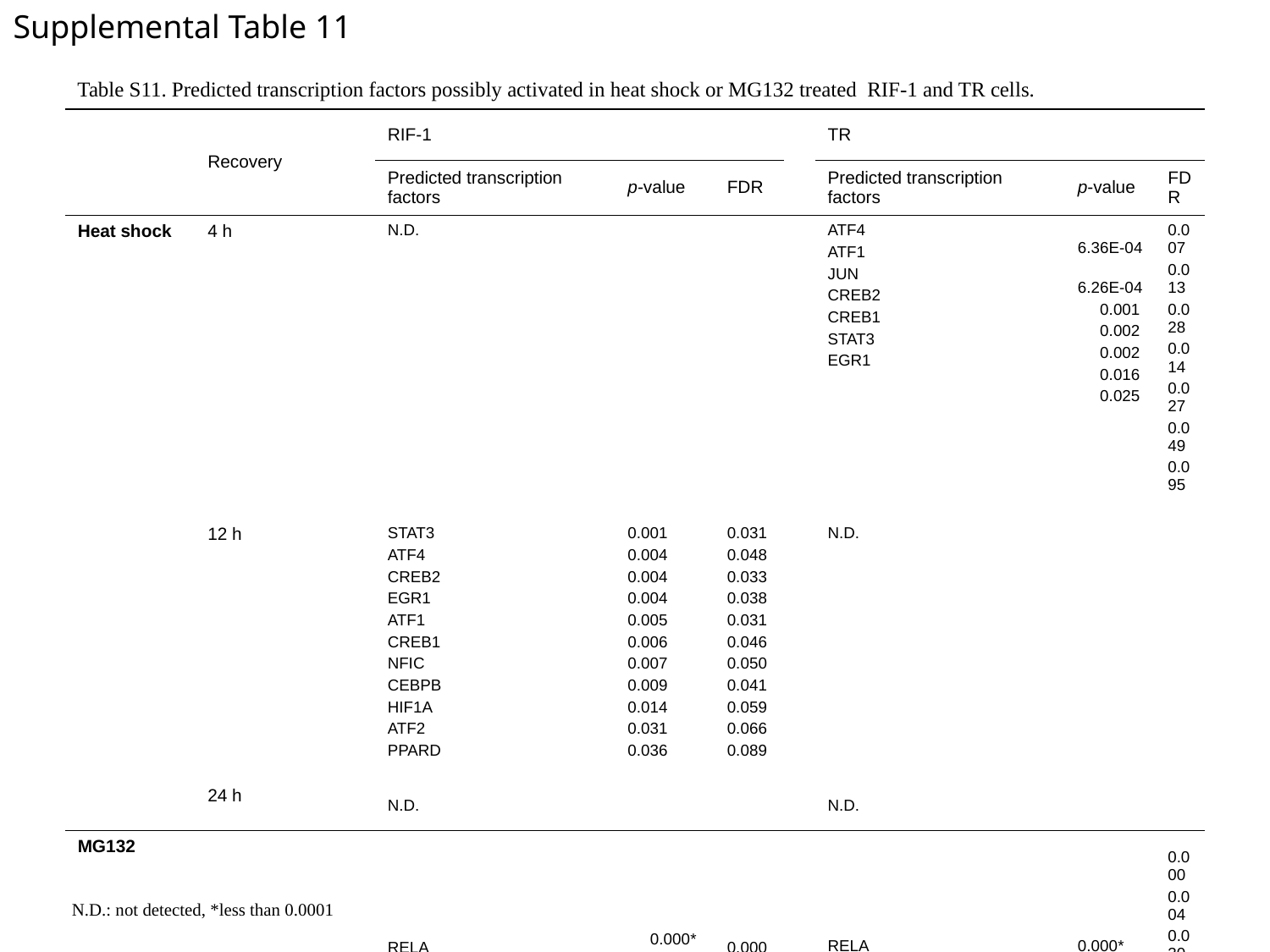

Supplemental Table 11
Table S11. Predicted transcription factors possibly activated in heat shock or MG132 treated RIF-1 and TR cells.
| | Recovery | RIF-1 | | | | TR | | |
| --- | --- | --- | --- | --- | --- | --- | --- | --- |
| | | Predicted transcription factors | p-value | FDR | | Predicted transcription factors | p-value | FDR |
| Heat shock | 4 h | N.D. | | | | ATF4 ATF1 JUN CREB2 CREB1 STAT3 EGR1 | 6.36E-04 6.26E-04 0.001 0.002 0.002 0.016 0.025 | 0.007 0.013 0.028 0.014 0.027 0.049 0.095 |
| | 12 h | STAT3 ATF4 CREB2 EGR1 ATF1 CREB1 NFIC CEBPB HIF1A ATF2 PPARD | 0.001 0.004 0.004 0.004 0.005 0.006 0.007 0.009 0.014 0.031 0.036 | 0.031 0.048 0.033 0.038 0.031 0.046 0.050 0.041 0.059 0.066 0.089 | | N.D. | | |
| | 24 h | N.D. | | | | N.D. | | |
| MG132 | 2 h | RELA STAT3 ATF4 HIF1A LEF1 SP3 EGR1 ATF1 NFKB RARB CEBPB | 0.000\* 8.02E-04 0.001 0.002 0.007 0.011 0.013 0.018 0.027 0.044 0.042 | 0.000 0.006 0.004 0.031 0.031 0.063 0.062 0.067 0.091 0.092 0.083 | | RELA ATF4 ATF1 HIF1A CREB1 SP3 CEBPB EGR1 LEF1 ETS2 | 0.000\* 3.96E-04 0.003 0.003 0.007 0.012 0.013 0.013 0.020 0.028 | 0.000 0.004 0.030 0.040 0.070 0.062 0.062 0.058 0.067 0.069 |
| | 8.5 h | N.D. | | | | RELA | 0.006 | 0.036 |
| | 18 h | STAT3 | 0.005 | 0.035 | | N.D. | | |
N.D.: not detected, *less than 0.0001
